# Investigating the metabolomic pathways in female reproductive endocrine disorders: a Mendelian randomization study

**DOI:** 10.3389/fendo.2024.1438079

**Published:** 2024-10-31

**Authors:** Fei-fan Lu, Zheng Wang, Qian-qian Yang, Feng-shang Yan, Chang Xu, Ming-tang Wang, Zhu-jing Xu, Sheng-yun Cai, Rui Guan

**Affiliations:** ^1^ Department of Obstetrics and Gynecology, Changhai Hospital, Naval Medical University, Shanghai, China; ^2^ Department of Urology, Changhai Hospital, Naval Medical University, Shanghai, China; ^3^ Department of Obstetrics and Gynecology, Shanghai Sixth People’s Hospital, Shanghai, China

**Keywords:** Mendelian randomization, metabolites, reproductive endocrine disorders, polycystic ovary syndrome, endometriosis, female infertility, genome wide association study, instrumental variables

## Abstract

**Introduction:**

Reproductive endocrine disorders (RED), including polycystic ovary syndrome (PCOS), endometriosis (EMs), and female infertility (FI), significantly affect women’s health globally, with varying prevalence across different regions. These conditions can be addressed through medication, surgical interventions, and lifestyle modifications. However, the limited understanding of RED’s etiology and the substantial economic burden of its treatment highlight the importance of investigating its pathogenesis. Metabolites play a critical role in metabolic processes and are potentially linked to the development of RED. Despite existing studies suggesting correlations between metabolites and RED, conclusive evidence remains scarce, primarily due to the observational nature of these studies, which are prone to confounding factors.

**Methods:**

This study utilized Mendelian Randomization (MR) to explore the causal relationship between metabolites and RED, leveraging genetic variants associated with metabolite levels as instrumental variables to minimize confounding and reverse causality. Data were obtained from the Metabolomics GWAS Server and the IEU OpenGWAS project. Instrumental variables were selected based on their association with the human gut microbiota composition, and the GWAS summary statistics for metabolites, PCOS, EMs, and FI were analyzed. The MR-Egger regression and random-effects inverse-variance weighted (IVW) methods were employed to validate the causal relationship. Cochran’s Q test was employed to evaluate heterogeneity, sensitivity analysis was performed using leave-one-out analysis, and for pleiotropy analysis, the intercept term of MR-Egger’s method was investigated.

**Results:**

The MR analysis revealed significant associations between various metabolites and RED conditions. For instance, a positive association was found between 1-palmitoylglycerophosphocholine and PCOS, while a negative association was noted between phenylacetate and FI. The study identified several metabolites associated with an increased risk and others with protective effects against PCOS, EMs, and FI. These findings highlight the complex interplay between metabolites and RED, suggesting potential pathways through which these conditions could be influenced or treated.

**Conclusion:**

This MR study provides valuable insights into the causal relationship between metabolites and female reproductive endocrine disorders, suggesting that metabolic alterations play a significant role in the pathogenesis of PCOS, EMs, and FI, and offering a foundation for future research and therapeutic development.

## 1 Introduction

Reproductive endocrine disorders (RED) affecting females, including polycystic ovary syndrome (PCOS), endometriosis (EMs), and female infertility (FI), have been a major health issue for women in their reproductive years (1, 2). Research studies reveal that the global prevalence of PCOS varies between 4% and 21%. In China, the reported incidence of PCOS among women aged 19-45 years is 5.6% (3). EMs affects approximately 10% of women of reproductive age worldwide, affecting 190 million people (4). While in the United States, approximately 10% to 15% of individuals are affected by FI, with around 30% of cases having an unknown etiology (5).

Early diagnosis of RED is crucial, especially of endometriosis, given its significant impact on the reproductive health and fertility potential of young patients (6). Timely identification, followed by effective treatment, is essential to reversing infertility, increasing the likelihood of a successful pregnancy, and improving overall quality of life. RED can be managed through various approaches, including medication, surgical intervention, and lifestyle adjustments, emphasizing the importance of early and appropriate therapeutic strategies (7). The major goal of medical care is to regulate the menstrual cycle, hormone secretion, and metabolism. On the other hand, surgical intervention generally aims to remove lesions, control pain, and assist with reproductive technologies. Lifestyle alterations prioritize the management of diet, exercise routines, and behavioral adjustments. Because the understanding of the etiology of RED is limited, the annual global expenditure on its treatment remains enormous, which places a significant burden on the global economy (4, 8, 9). Researching the pathogenesis of RED will not only reduce the worldwide economic burden but also aid in the advancement of more efficient treatments (1, 10, 11).

The development of the RED complex usually arises from an unhealthy lifestyle, genetic and hormonal abnormalities, inflammation, and metabolic disturbances (10–13). Metabolites are pivotal in metabolic processes since they either produce or consume resources, hence ensuring the proper functioning of physiological processes. For example, disturbances in sugar metabolism might result in the onset of chronic illnesses (14). The findings of various studies have indicated that the processes of lipid metabolism, glucose metabolism, and sphingolipid metabolism may be intricately linked to the pathogenesis of RED (15–17). Patients with PCOS exhibit a notable prevalence of common metabolic dysfunction (16). A robust association exists between inflammation in the FI signal and elevated androgen metabolism (15, 18). Supplementing vitamin C and E has been proposed as a possible treatment for reducing painful symptoms in patients with Ems (19). Hence, metabolic alterations are closely connected to the development of RED. Nevertheless, the existing clinical evidence establishing the correlation between metabolites and RED is restricted and mostly obtained from observational studies, which may be influenced by confounding factors. Therefore, gaining a deeper understanding of the connection between metabolites and RED, as well as the probable mechanisms involved, is of great therapeutic importance for accurately assessing risk and improving treatment approaches.

Mendelian randomization (MR) is a commonly employed method for inferring causality. It utilizes genetic variants that are linked to the exposure of interest as instrumental variables (IVs) to establish the causal impact of the exposure on the outcome. This approach is effective in isolating the effect of exposures from potential confounding factors, such as environmental influences and lifestyle choices (20–24). It can reduce the impact of reverse causality to ensure maximum validity (25, 26). The swift advancement of Genome Wide Association Study (GWAS) data additionally establishes a strong basis for the execution of MR Research. The relationship between metabolomics and MR research, particularly in the context of elucidating the causal pathways in human diseases, exemplifies a compelling application of this methodology (27, 28). Metabolomics, the comprehensive study of small molecule metabolites within biological systems, offers a dynamic and sensitive reflection of both genetic and environmental influences on an organism’s state. When integrated with MR, metabolomics can provide powerful insights into the causal relationships between specific metabolic profiles and disease outcomes.

At present, there is a lack of research that uses the MR approach to thoroughly examine the cause-and-effect relationship between metabolites and the start of RED. Our study aimed to examine the potential causal relationships between specific genotypes, plasma metabolite levels, and the risk of developing PCOS, EMs, and FI using MR analysis. We hypothesize that genetic variations directly influence metabolite levels and, consequently, disease outcomes, independent of confounding factors. By exploring these causal connections, the findings could help identify pathogenic metabolites with causal relationships, potentially guiding targeted therapeutic strategies and preventive measures for these REDs.

## 2 Methods

### 2.1 Study design

**Figure 1:**
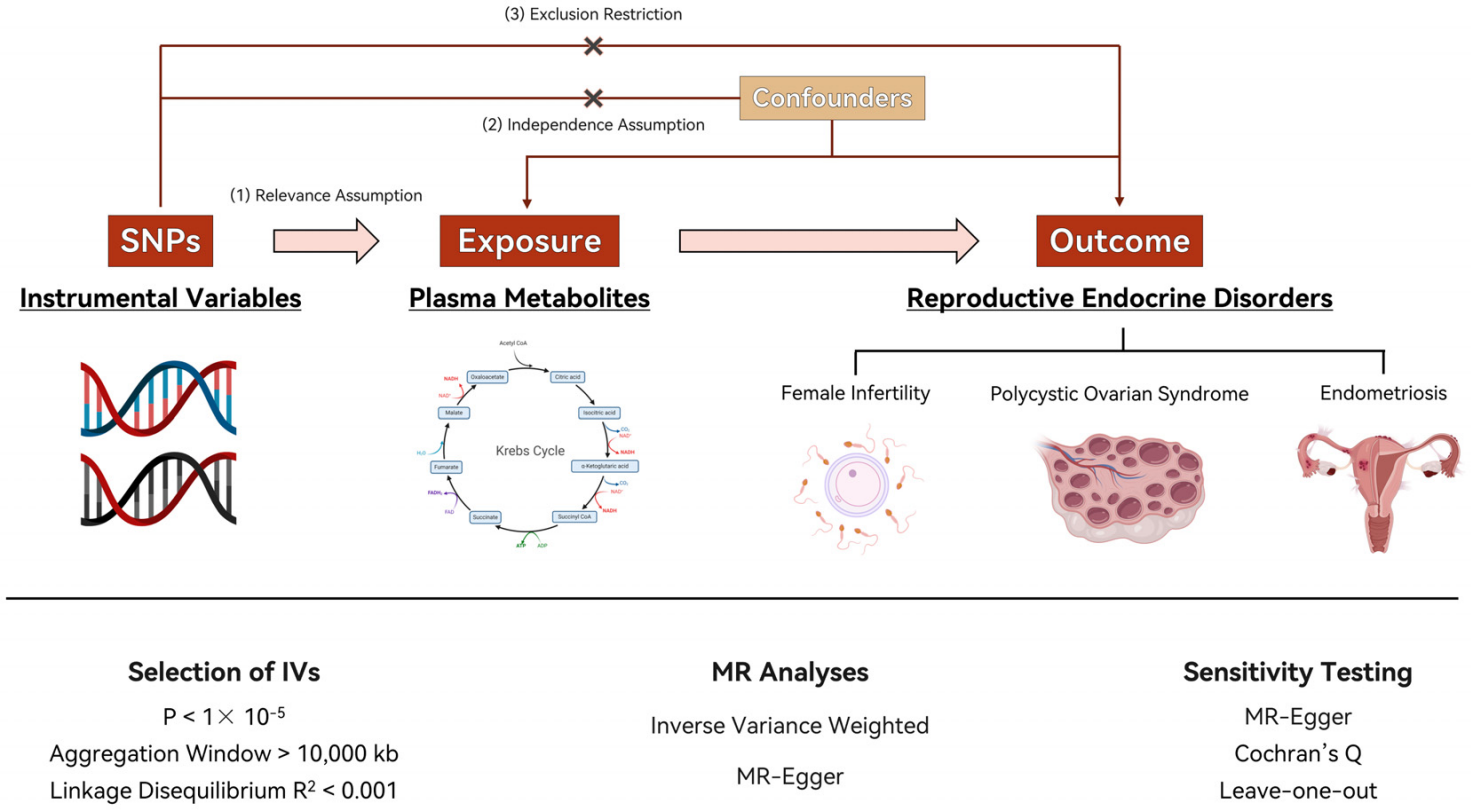
Flow chart of the study. Image illustrates the Mendelian randomization (MR) framework used to investigate the causal effects of plasma metabolite levels on reproductive endocrine disorders, including polycystic ovary syndrome (PCOS), endometriosis (EMs), and female infertility (FI). Instrumental variables (IVs) are selected based on single nucleotide polymorphisms (SNPs) fulfilling stringent criteria for relevance (P < 1 × 10^-5^), linkage disequilibrium (R^2^ < 0.001), and a sizable aggregation window (>10,000 kb). MR analyses utilize inverse variance weighted (IVW) methods and MR-Egger regression for causal inference, with additional sensitivity testing (Cochran’s Q and leave-one-out analysis) to assess the robustness of the findings.

In our study on the link between plasma metabolites and the development of PCOS, EMs and FI levels, we propose three key hypotheses. First, we expect a correlation between certain genotypes and plasma metabolite levels, suggesting a genetic influence on these metabolites that could affect disease risk. Second, these genotypes should not be influenced by confounding factors, ensuring that any observed relationship with metabolite levels is likely due to a genuine biological mechanism. Third, we assume that these genotypes directly impact the metabolites in question, thereby affecting the risk or progression of PCOS, EMs, and FI. This framework aims to clarify how genetic variations contribute to the observed outcomes, using Mendelian randomization to explore potential causal connections. **Figure 1** depicts the process of Mendelian randomization including metabolites that are linked to PCOS, EMs, and FI.

### 2.2 Data sources

The metabolite GWAS data was obtained from the Metabolomics GWAS Server website (https://metabolomips.org/gwas/), while the genetic variance data related to PCOS, EMs, and FI was obtained from the IEU OpenGWAS project website (https://gwas.mrcieu.ac.uk/) (**Table 1**). IVs were chosen as single-nucleotide polymorphisms (SNPs) that are linked to the composition of the plasma metabolites. Due to the fact that the data utilized in this study were collected from published studies or public databases, ethics approval was not necessary. The GWAS summary statistics for the metabolites were acquired from the Metabolomics GWAS Server, which may be accessed at https://metabolomips.org/gwas/. This study involves genome-wide association scans combined with high-throughput metabolic monitoring to get unique insights into the impact of genetic variation on metabolism and complex diseases. The researchers have conducted a thorough investigation into the genetic factors that affect human metabolism. They studied a total of 7,824 adult individuals from two European population studies. The study identified significant associations between 145 metabolic loci and more than 1400 metabolites in human blood, revealing their biochemical connectivity (29). The GWAS summary datasets for PCOS were obtained from the OpenGWAS database. The GWAS ID is finn-b-E4_POCS, with a total of 118,870 female participants, consisting of 642 cases and 118,228 controls. The total count of SNPs is 16,379,676. The GWAS data for individuals with endometriosis were obtained from a recent genome-wide association study. The selected trait for analysis was endometriosis, and the discovery sample consisted of 4,511 cases of European ancestry and 227,260 controls of European ancestry (30). The GWAS summary datasets for FI were obtained from the OpenGWAS database. The GWAS ID is finn-b-N14_FEMALEINFERT, with a total of 75,470 female participants, consisting of 6,481 cases and 68,969 controls. The total count of SNPs is 16,377,038.

**Table 1:** Detailed information of datasets.

	Data source	Phenotype	Sample size	Cases	Population	Adjustment
**Exposure**	The Metabolomics GWAS Server	Metabolites	7824	-	European	-
**Outcomes**	finn-b-E4_POCS	Polycystic Ovarian Syndrome	-	642	European	Males and Females
	ebi-a-GCST90018839	Endometriosis	231771	4511	European	-
	finn-b-N14_FEMALEINFERT	Female Infertility	-	6481	European	Males and Females

### 2.3 Selection of IVs

The following criteria were employed in this study for the purpose of screening instrumental variables (31). Typically, this is accomplished by utilizing data obtained from extensive GWAS. The selected SNPs should possess a strong and firmly proven correlation with the exposure. The IVs demonstrate a robust association with exposure, as evidenced by a significant criteria of P < 1× 10-5 (correlation hypothesis) (32). It is necessary to measure the degree of correlation between each SNP and the exposure. This entails utilizing statistical analysis to quantify the magnitude of the impact of each SNP on the exposure, often quantified as the beta coefficient in a regression model (33). It’s crucial to ensure that the SNPs used as IVs affect the outcome only through the exposure and not through other pathways. This is known as the assumption of no pleiotropy (34). The SNPs selected by the MR Method adhere to the genetic principle of random parental allele assignment to offspring, thereby minimizing the influence of environmental and acquired factors. Consequently, it can be theoretically assumed that instrumental variables are independent of social, economic, and cultural factors. SNPs in close proximity on the genome can be in linkage disequilibrium, meaning they are often inherited together. It’s important to ensure that the selected SNPs are independent of each other, or to adjust the analysis for LD (35). The selected SNPs should collectively explain a significant portion of the variance in the exposure. Weak instruments (those that explain only a small fraction of the variance) can lead to biased MR estimates. Moreover, an F statistic greater than 10 is employed as a criterion for assessing weak instrumental variables (36). The screening condition for selecting meaningful SNPs from the aggregated GWAS data of metabolites were set as P < 1×10-5. The coefficient of linkage disequilibrium is r2 < 0.001 and the width of the linkage disequilibrium region was 10000kb. These were applied to ensure the independence of each SNP and eliminate the impact of linkage imbalance on the results (32, 33, 35). The PhenoScanner (http://www.phenoscanner.medschl.cam.ac.uk/) was utilized to account for confounding factors and assess the outcome associated with each SNP. From the aforementioned screened SNPs, those extracted from the aggregated GWAS data of PCOS, EMs, and FI had a minimum r2 value greater than 0.8. This summarizes the information contained in this dataset.

### 2.4 Verification of causality

The MR-Egger regression and random-effects inverse-variance weighted (IVW) methods were employed to validate the causal relationship between exposure (Metabolites) and outcomes (PCOS, EMs, and FI), using SNPs as instrumental variables. IVW, which is widely recognized as the primary outcome measure, was utilized (37). In this approach, each locus’s inverse of variance (R2) served as the weight for estimating the causal effect based on multiple SNPs as instrumental variables. These weighted causal effect estimates were then summed to obtain the final estimate using IVW method. MR-Egger method essentially incorporates a weaker assumption (InSIDE) within IVW framework to perform causal effect estimation while introducing a regression intercept term to detect and correct bias caused by pleiotropic effects of instrumental variables in order to estimate the causal relationship between exposure and outcome (38). When there is horizontal pleiotropy present, MR-Egger results can be referenced. Additionally, random-effects inverse-variance weighting method was applied for analyzing the causal relationship between variables with MR-Egger regression serving as a supplementary analysis technique (39). All aforementioned methods were implemented using TwoSample MR Package in R 4.1.0 software at a significance level of α=0.05. The SNP annotation was performed using online tools available at https://biit.cs.ut.ee/gprofiler/snpense. SNPense tool facilitated the mapping of human SNP rs-codes to gene names, providing chromosomal coordinates and predicted variant effects. Mapping was restricted to variants that overlapped with protein-coding Ensembl genes (40). All essential data were retrieved from Ensembl variation.

### 2.5 Heterogeneity and horizontal pleiotropy

The heterogeneity was assessed using Cochran’s Q test, with results considered heterogeneous if P < 0.05. I² (I-squared) was also used to measure heterogeneity, indicating the proportion of variation due to heterogeneity in total variation (34). The range for I² is from 0% to 100%, with larger values indicating higher levels of heterogeneity. The formula for calculating I = 2(Q - Q_df)/Q. For pleiotropy analysis, the intercept term of MR-Egger’s method was utilized, while leave-one-out analysis was employed for sensitivity analysis. IVs were deemed non-pleiotropic when the intercept term of the MR-Egger regression model equaled zero (P > 0.05) (41). To conduct leave-one-out analysis on IVs, each SNP was gradually removed and the remaining SNPs were re-analyzed to observe their individual effects (42).

## 3 Results

### 3.1 IVs screening

After undergoing multiple screenings, the dataset of PCOS, EMs, and FI associated with metabolites ultimately comprised 13,615 SNPs. **Supplementary Table 1** presents the basic information of select SNPs, while the remaining SNPs exhibit similar characteristics. The F-statistic for each individual SNP ranged from 17.64 to 1294.286 (mean: 26.468), suggesting that weak instrumental variables were unlikely to significantly impact the causal association. See the **Supplementary Table 1** for more details.

### 3.2 The analysis of causal relationship

**Figure 2:**
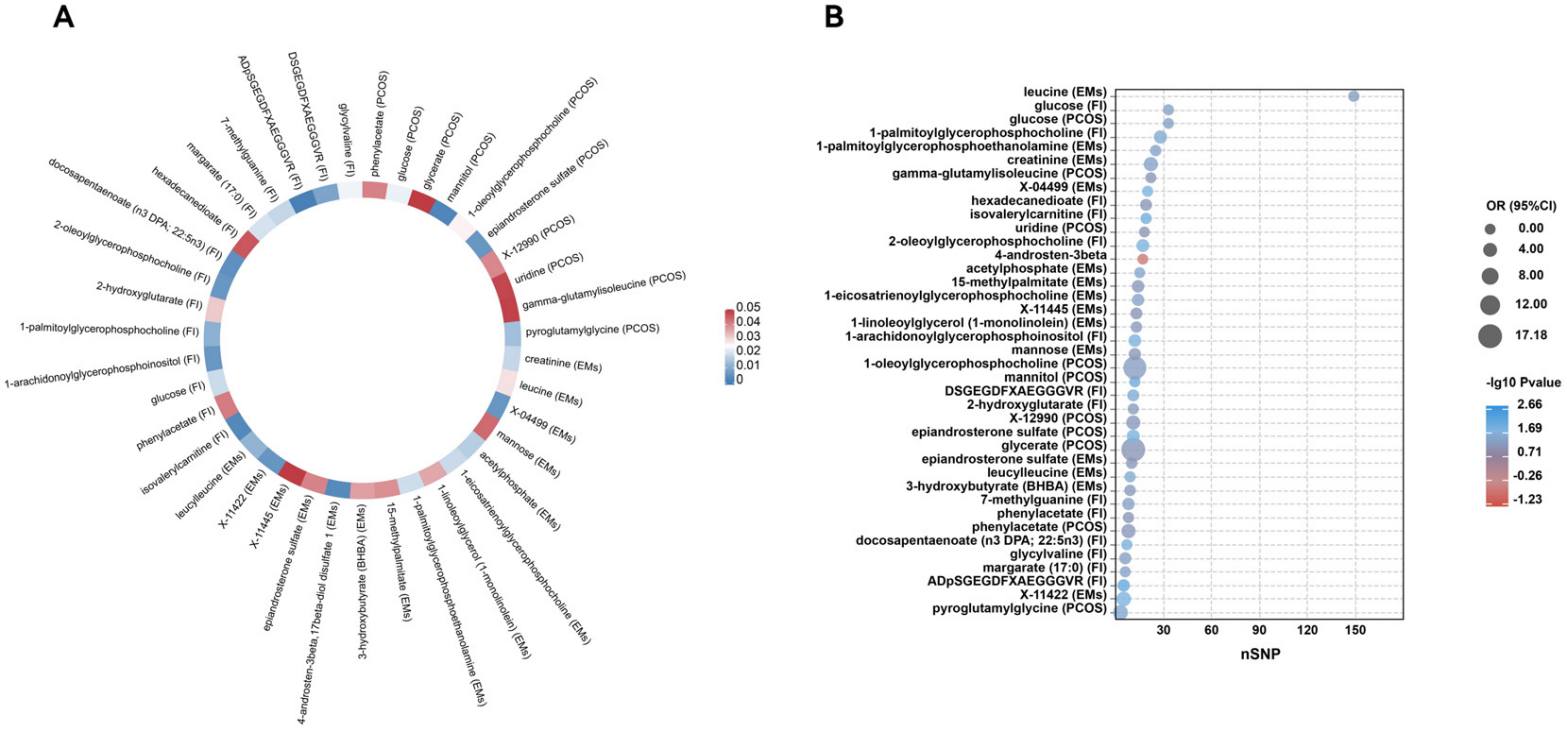
Forest plots of MR-estimated effects of plasma metabolites on PCOS, endometriosis, and female infertility risk. Image depicts a comprehensive Mendelian randomization (MR) analysis to assess the association between plasma metabolites and the risk of developing common reproductive endocrine disorders: polycystic ovary syndrome (PCOS) in **(A)**, endometriosis in **(B)**, and female infertility (FI) in **(C)**. For each disorder, forest plots illustrate the estimated effects (odds ratios with 95% confidence intervals) of the metabolite levels, with the number of single nucleotide polymorphisms (SNPs) utilized as instrumental variables (nSNP) presented alongside. A significant association is denoted by P values that breach the threshold of statistical significance. The effect size for each metabolite is visually captured through the width of the horizontal lines (confidence intervals).

**Figure 3:**
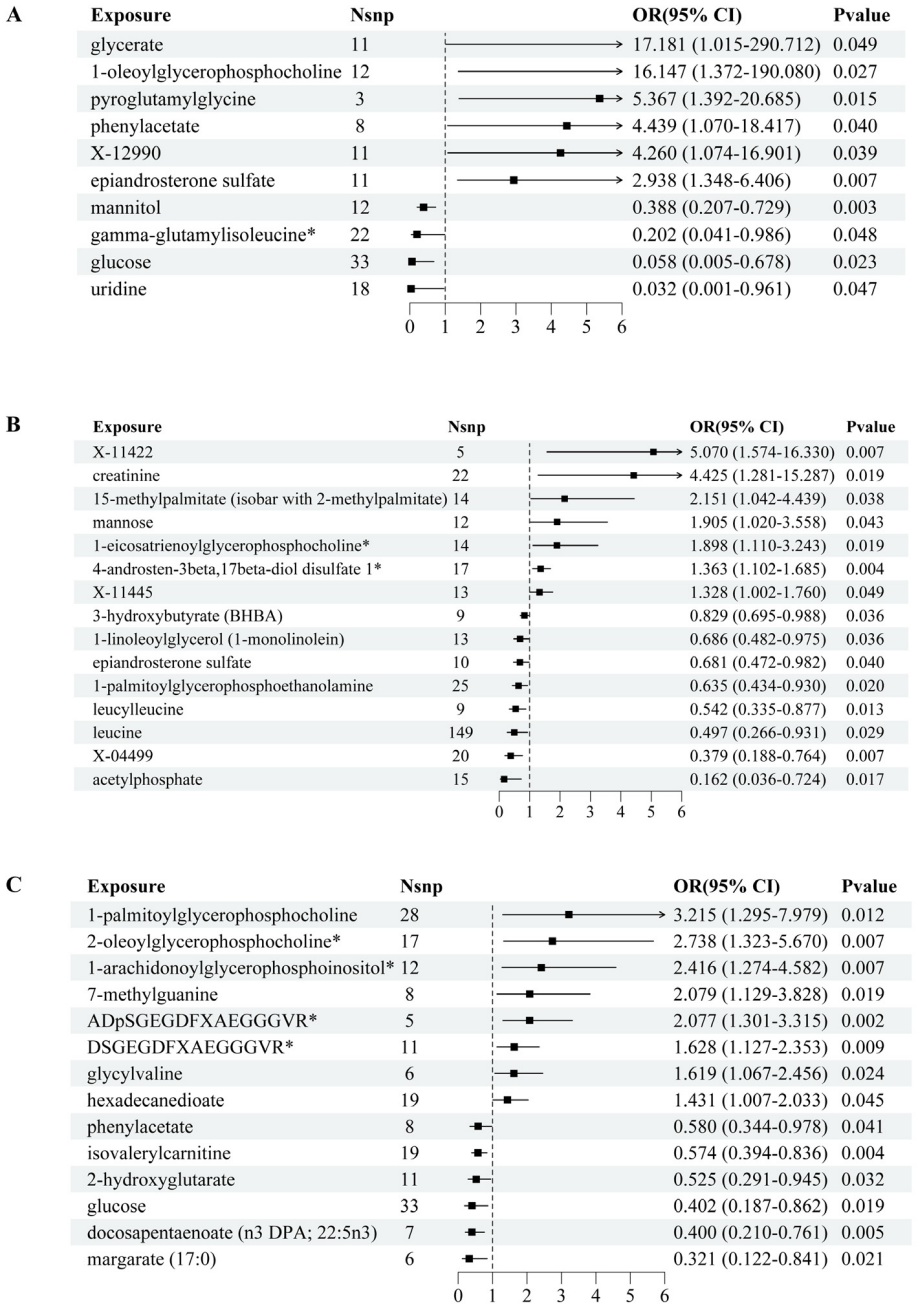
Circular heatmap and bubble plot of MR results for metabolites in reproductive endocrine disorders. **(A)** is a circular heatmap that encapsulates the MR findings for a spectrum of metabolites, with color intensities reflecting the magnitude of -log10 P values, indicating the strength of the associations. Each segment is annotated with the metabolite’s name and the specific condition it’s associated with (PCOS for polycystic ovary syndrome, EMs for endometriosis, and FI for female infertility), facilitating a comparative overview of metabolites across different outcomes. **(B)** is a bubble plot, showcasing the odds ratios (OR) and the corresponding P values (-log10 scale) for each significant metabolite’s influence on the disease states. The size of each bubble represents the number of single nucleotide polymorphisms (nSNP) used as instrumental variables, thus reflecting the genetic weight of the analysis. The color gradient represents the significance levels, with cooler blue tones denoting stronger associations and warmer red tones suggesting weaker statistical significance.

We conducted a comprehensive MR analysis to assess the causal relationships between each screened metabolite and conditions with PCOS, EMs, and FI (**Figure 2**, **Supplementary Figure 1**). The IVW analysis results demonstrated that substances with confidence intervals that do not cross the line of no effect and have a p-value less than 0.05 are considered to have a statistically significant association with the outcome (**Figure 3**).

In PCOS (**Figure 2A**), the analysis revealed significant associations across a range of metabolite classes, including amino acids, carbohydrates, lipids, nucleotides, and peptides. Phenylacetate, an amino acid, exhibited a notable association with an increased risk of PCOS (OR = 4.439, 95% CI: 1.070-18.417, p = 0.0401). In the carbohydrate category, glucose showed a protective effect (OR = 0.058, 95% CI: 0.005-0.678, p = 0.0232), while glycerate and mannitol were linked to varying effects on PCOS risk. Lipid metabolites, particularly 1-oleoylglycerophosphocholine and epiandrosterone sulfate, were significantly associated with PCOS, underscoring their potential roles in its pathogenesis. Additionally, nucleotide uridine and peptides like pyroglutamylglycine also showed significant associations, suggesting their involvement in PCOS development.

For Endometriosis (**Figure 2B**), our findings indicated significant associations with amino acids such as creatinine and leucine, pointing to a potential influence on EMs risk (OR = 4.425, 95% CI: 1.281-15.287, p = 0.0187 for creatinine). Carbohydrates like mannose and energy metabolites such as acetylphosphate also demonstrated significant links to EMs. Among lipids, several metabolites including 1-eicosatrienoylglycerophosphocholine and 4-androsten-3beta,17beta-diol disulfate 1 showed associations with EMs risk, highlighting the complexity of lipid metabolism in EMs etiology. The analysis further identified significant associations with other lipid metabolites, emphasizing their potential regulatory roles in EMs.

In the context of FI (**Figure 2C**), isovalerylcarnitine, an amino acid, was inversely associated with FI (OR = 0.574, 95% CI: 0.394-0.836, p = 0.0038), suggesting a protective role against insulin resistance. Carbohydrates and lipids also displayed significant associations with FI levels. Specifically, glucose showed an inverse relationship, while various lipid metabolites such as 1-arachidonoylglycerophosphoinositol and docosapentaenoate were linked to FI alterations, indicating their importance in insulin metabolism and sensitivity. Additionally, nucleotides and peptides demonstrated significant associations, further underscoring the multifaceted nature of genetic influences on FI levels.

We summarized a comprehensive information of the metabolite classifications (classified as amino acids, carbohydrates, lipids, nucleotides, and peptides) associated with three diseases, as well as the number of SNPs and their causality (**Table 2**).

**Table 2:** MR results of causal links.

Data source	Classification	Classification	Nsnp	Methods	OR (95%CI)	P-value
**PCOS**	Amino acid	phenylacetate	8	IVW	4.439(1.070-18.417)	0.0401
	Carbohydrate	glucose	33		0.058(0.005-0.678)	0.0232
	Carbohydrate	glycerate	11		17.181(1.015-290.712)	0.0488
	Carbohydrate	mannitol	12		0.388(0.207-0.729)	0.0032
	Lipid	1-oleoylglycerophosphocholine	12		16.147(1.372-190.080)	0.0270
	Lipid	epiandrosterone sulfate	11		2.938(1.348-6.406)	0.0067
	Lipid	X-12990	11		4.260(1.074-16.901)	0.0393
	Nucleotide	uridine	18		0.032(0.001-0.961)	0.0474
	Peptide	gamma-glutamylisoleucine	22		0.202(0.041-0.986)	0.0480
	Peptide	pyroglutamylglycine	3		5.367(1.392-20.685)	0.0146
**EMs**	Amino acid	creatinine	22	IVW	4.425(1.281-15.287)	0.0187
	Amino acid	leucine	149		0.497(0.266-0.931)	0.0289
	Amino acid	X-04499	20		0.379(0.188-0.764)	0.0066
	Carbohydrate	mannose	12		1.905(1.020-3.558)	0.0430
	Energy	acetylphosphate	15		0.162(0.036-0.724)	0.0171
	Lipid	1-eicosatrienoylglycerophosphocholine	14		1.898(1.110-3.243)	0.0191
	Lipid	1-linoleoylglycerol (1-monolinolein)	13		0.686(0.482-0.975)	0.0356
	Lipid	1-palmitoylglycerophosphoethanolamine	25		0.635(0.434-0.930)	0.0197
	Lipid	15-methylpalmitate (isobar with 2-methylpalmitate)	14		2.151(1.042-4.439)	0.0383
	Lipid	3-hydroxybutyrate (BHBA)	9		0.829(0.695-0.988)	0.0365
	Lipid	4-androsten-3beta,17beta-diol disulfate 1	17		1.363(1.102-1.685)	0.0042
	Lipid	epiandrosterone sulfate	10		0.681(0.472-0.982)	0.0398
	Lipid	X-11445	13		1.328(1.002-1.760)	0.0487
	Nucleotide	X-11422	5		5.070(1.574-16.330)	0.0065
	Peptide	leucylleucine	9		0.542(0.335-0.877)	0.0126
**FI**	Amino acid	isovalerylcarnitine	19	IVW	0.574(0.394-0.836)	0.0038
	Amino acid	phenylacetate	8		0.580(0.344-0.978)	0.0408
	Carbohydrate	glucose	33		0.402(0.187-0.862)	0.0193
	Lipid	1-arachidonoylglycerophosphoinositol	12		2.416(1.274-4.582)	0.0069
	Lipid	1-palmitoylglycerophosphocholine	28		3.215(1.295-7.979)	0.0118
	Lipid	2-hydroxyglutarate	11		0.525(0.291-0.945)	0.0317
	Lipid	2-oleoylglycerophosphocholine	17		2.738(1.323-5.670)	0.0067
	Lipid	docosapentaenoate (n3 DPA; 22:5n3)	7		0.400(0.210-0.761)	0.0053
	Lipid	hexadecanedioate	19		1.431(1.007-2.033)	0.0454
	Lipid	margarate (17:0)	6		0.321(0.122-0.841)	0.0207
	Nucleotide	7-methylguanine	8		2.079(1.129-3.828)	0.0187
	Peptide	ADpSGEGDFXAEGGGVR	5		2.077(1.301-3.315)	0.0022
	Peptide	DSGEGDFXAEGGGVR	11		1.628(1.127-2.353)	0.0095
	Peptide	glycylvaline	6		1.619(1.067-2.456)	0.0236

MR, Mendelian Randomization; PCOS, polycystic ovary syndrome; EMs, endometriosis; FI, female infertility; IVW, inversed-variance weighted; OR, odds ratio; CI, confidence interval.

The detailed list provides supplementary information for SNP annotation in metabolites of imparity, and it uncovers genetic loci where metabolites exert an impact on PCOS, EMs, and FI (**Supplementary Table 2**).

### 3.3 Sensitivity testing

In our study employing MR to assess the impact of specific metabolites on the risk of PCOS, EMs, and alterations in FI levels, we evaluated outcomes across various metabolite classes. The results are organized by disease condition, including assessments of heterogeneity and horizontal pleiotropy, which are crucial for understanding the robustness and specificity of the associations identified.

We summarized the analysis of MR heterogeneity and directional pleiotropy in metabolites and outcome variables associated with three diseases (**Table 3**, **Supplementary Figure 2**, **3**). The identified SNPs did not exhibit a statistically significant impact on the estimates of causal association.

**Table 3:** Evaluation of heterogeneity and pleiotropy.

Outcomes	Classification	Metabolites	Heterogeneity				Horizontal Pleiotropy	
			I^2^(%)	Cochran’s Q	P-value	Egger intercept	SE	P-value
**PCOS**	Amino acid	phenylacetate	13	8.0772	0.3258	0.0241	0.0562	0.6826
	Carbohydrate	glucose	11	36.1044	0.2826	0.0260	0.0271	0.3449
	Carbohydrate	glycerate	0	7.7892	0.6494	-0.0670	0.0638	0.3206
	Carbohydrate	mannitol	0	8.1490	0.6999	-0.0945	0.0628	0.1634
	Lipid	1-oleoylglycerophosphocholine	7	11.8449	0.3754	0.0737	0.0470	0.1477
	Lipid	epiandrosterone sulfate	25	13.3746	0.2035	0.0335	0.0436	0.4625
	Lipid	X-12990	0	7.8790	0.6407	-0.0140	0.0709	0.8479
	Nucleotide	uridine	0	14.9945	0.5959	0.0195	0.0584	0.7431
	Peptide	gamma-glutamylisoleucine	0	18.5263	0.6155	-0.0287	0.0345	0.4160
	Peptide	pyroglutamylglycine	0	0.9812	0.6122	0.1157	0.1383	0.5563
**EMs**	Amino acid	creatinine	15	24.6618	0.2621	-0.0053	0.0123	0.6733
	Amino acid	leucine	9	163.4983	0.1815	0.0050	0.0036	0.1682
	Amino acid	X-04499	6	20.2751	0.3782	0.0188	0.0211	0.3838
	Carbohydrate	mannose	0	5.5446	0.9020	0.0197	0.0144	0.2024
	Energy	acetylphosphate	18	17.1042	0.2507	0.0058	0.0314	0.8567
	Lipid	1-eicosatrienoylglycerophosphocholine	17	15.7089	0.2652	-0.0284	0.0178	0.1360
	Lipid	1-linoleoylglycerol (1-monolinolein)	21	15.2416	0.2285	0.0357	0.0184	0.0781
	Lipid	1-palmitoylglycerophosphoethanolamine	0	17.0084	0.8483	0.0052	0.0068	0.4529
	Lipid	15-methylpalmitate (isobar with 2-methylpalmitate)	21	16.3602	0.2302	0.0118	0.0140	0.4175
	Lipid	3-hydroxybutyrate (BHBA)	15	9.4153	0.3085	-0.0293	0.0130	0.0585
	Lipid	4-androsten-3beta,17beta-diol disulfate 1	0	8.2044	0.9425	-0.0020	0.0101	0.8475
	Lipid	epiandrosterone sulfate	0	4.1942	0.8982	0.0016	0.0380	0.9668
	Lipid	X-11445	35	18.5627	0.0996	0.0079	0.0307	0.8013
	Nucleotide	X-11422	0	0.4459	0.9785	0.0016	0.0354	0.9658
	Peptide	leucylleucine	0	1.9940	0.9812	-0.0076	0.0201	0.7172
**FI**	Amino acid	isovalerylcarnitine	5	18.8542	0.4009	0.0005	0.0084	0.9512
	Amino acid	phenylacetate	29	9.8058	0.1998	0.0024	0.0208	0.9122
	Carbohydrate	glucose	0	25.5290	0.7841	0.0004	0.0084	0.9602
	Lipid	1-arachidonoylglycerophosphoinositol	0	10.7537	0.4641	-0.0144	0.0140	0.3279
	Lipid	1-palmitoylglycerophosphocholine	0	24.6588	0.5936	0.0149	0.0192	0.4440
	Lipid	2-hydroxyglutarate	0	7.8895	0.6396	-0.0163	0.0151	0.3083
	Lipid	2-oleoylglycerophosphocholine	0	15.3490	0.4992	0.0311	0.0193	0.1269
	Lipid	docosapentaenoate (n3 DPA; 22:5n3)	0	2.4955	0.8690	-0.0034	0.0326	0.9204
	Lipid	hexadecanedioate	27	24.7590	0.1317	-0.0025	0.0129	0.8468
	Lipid	margarate (17:0)	0	3.9381	0.5584	0.0052	0.0440	0.9108
	Nucleotide	7-methylguanine	0	6.5614	0.4759	-0.0228	0.0196	0.2897
	Peptide	ADpSGEGDFXAEGGGVR	9	4.3992	0.3547	0.0106	0.0317	0.7596
	Peptide	DSGEGDFXAEGGGVR	0	9.7625	0.4616	0.0535	0.0295	0.1025
	Peptide	glycylvaline	0	4.4344	0.4887	0.0322	0.0518	0.5683

MR, Mendelian Randomization; PCOS, polycystic ovary syndrome; EMs, endometriosis; FI, female infertility.

For PCOS, heterogeneity, as measured by I^2^(%) and Cochran’s Q, varied across metabolites, with phenylacetate (amino acid) showing relatively low heterogeneity (I^2^ = 13%) but a significant horizontal pleiotropy p-value (Egger intercept p-value = 0.6826). Other notable findings include glucose (carbohydrate) with an I^2^ of 11% and no significant pleiotropy (Egger intercept p-value = 0.3449), and 1-oleoylglycerophosphocholine (lipid) with I^2^ = 7% and a marginally non-significant pleiotropy p-value (Egger intercept p-value = 0.1477). These results suggest a varied landscape of genetic instruments’ effects on PCOS risk, with some metabolites showing more stable and specific associations than others.

In EMs, creatinine (amino acid) exhibited moderate heterogeneity (I^2^ = 15%) and no significant pleiotropy (Egger intercept p-value = 0.6733). A high level of heterogeneity was noted for leucine (amino acid) with an I^2^ of 9% and a borderline significant pleiotropy (Egger intercept p-value = 0.1682), suggesting the need for cautious interpretation. The lipid metabolite 1-linoleoylglycerol (1-monolinolein) showed a notable I^2^ of 21% and a pleiotropy p-value nearing significance (Egger intercept p-value = 0.0781), indicating potential pleiotropic effects influencing its association with EMs.

For alterations in FI, isovalerylcarnitine (amino acid) had a low heterogeneity (I^2^ = 5%) and showed no evidence of significant horizontal pleiotropy (Egger intercept p-value = 0.9512), suggesting a more direct association with FI. Conversely, phenylacetate (amino acid) and glucose (carbohydrate) displayed higher heterogeneity but also no significant pleiotropy, highlighting the complexity of these associations.

Furthermore, the robustness of our findings was further validated by a leave-one-out sensitivity analysis, as illustrated in **Supplementary Figure 4**.

## 4 Discussion

This study represents a pioneering endeavor to unveil the intricate connections between metabolites and REDs from a metabolomics perspective. By employing MR analysis, we have identified a combined total of 39 metabolites (10 for PCOS, 15 for EMs, and 14 for FI) across various classes that are significantly associated with conditions. Through the process of gene annotation, it has been shown that there are potentially 442 different types of genes that could be linked to female REDs (**Supplementary Table 2**). This work is the first to use MR analysis to establish a causal link between metabolites and RED. These findings not only enrich our understanding of the metabolic underpinnings of RED but also pave the way for novel diagnostic and therapeutic approaches.

The Global Burden of Disease Study 2021 highlights infertility as an escalating global health challenge marked by rising prevalence and notable regional disparities (2). Identifying the causes of infertility is essential for devising effective strategies to mitigate its impact worldwide. Exploring these causes from an omics and metabolic perspective could provide valuable insights into the underlying mechanisms and potential therapeutic targets (43, 44). Many recent studies have focused on the significance of metabolites in female reproductive endocrine diseases. Li et al. discovered that mono-(2-ethylhexyl) phthalate (MEHP), a byproduct of DEHP, can disrupt ovarian function by causing irregularities in the 17β-hydroxysteroid dehydrogenase (17β-HSD) signaling pathway. This study sought to validate this idea in living organisms by conducting experiments on adult female Wistar rats (45). Li et al. conducted a study on the developmental abnormalities of the ovary in quail caused by di-(2-ethylhexyl) phthalate (DEHP) and its metabolite MEHP. They emphasized the receptor-mediated signaling pathway through which these metabolites hinder estradiol production and disrupt the hypothalamic-pituitary-ovarian axis (46). Additionally, Charifson et al. suggested that PCOS could be attributed to a mismatch between genetic variations that developed in physically active subsistence settings and the sedentary industrialized surroundings of modern times (47). In their study, Chu et al. examined the impact of continuous exposure to light on the disruption of circadian rhythm and its implications on reproductive, metabolic, and gut microbiome abnormalities resembling those seen in PCOS in a rat model. The study highlights the significance of circadian rhythm in the development of PCOS (48). In addition, Mukhopadhyay et al. conducted a review on the relationship between bisphenol A (BPA) and the risk of PCOS. Their focus was on how BPA can change the expression of genes that play a role in regulating hormones, which are often linked to the symptoms of PCOS (49). Ding et al. emphasized the importance of comprehending the impacts of endocrine disrupting chemicals (EDCs) on female reproductive health, particularly in relation to ovarian aging (50). To summarize, the data indicates that metabolites, namely those originating from phthalates and BPA, have a substantial impact on female reproductive endocrine disorders such as PCOS. Gaining a comprehensive understanding of the processes by which these metabolites interfere with endocrine pathways and affect ovarian function is essential for the development of successful treatments and interventions for these disorders (51). Additional investigation is necessary to enhance our comprehension of the intricate problems related to female reproductive health, including the impacts of EDCs, circadian rhythm disruption, and toxicological data from biomonitoring investigations (50, 52).

From the perspective of pathophysiological mechanisms, the growth of endometriotic deposits is dependent on the presence of estradiol, which is supplied by both systemic hormones and enhanced expression of aromatase and steroidogenic acute regulatory protein locally. Additionally, the expression of 17β-hydroxysteroid dehydrogenase 2 is reduced by endometriotic lesions, further contributing to the proliferation of these deposits (53). Furthermore, lesions exhibit heightened expression of estrogen receptor β (54). Moreover, the inhibition of progesterone receptor B in both normal and abnormal endometrial tissue is intensified in abnormal endometrial stromal cells due to epigenetic differential methylation of PR-B, HOX, and GATA family transcription-factor genes (55). This leads to disrupted progesterone signaling, commonly referred to as “progesterone resistance (56). PCOS is associated with adrenocortical steroidogenic dysfunction, as evidenced by research (57) Around one third of women with PCOS show elevated levels of dehydroepiandrosterone sulfate, which is an androgen metabolite or prohormone primarily produced by the adrenal cortex. Insulin resistance and compensatory hyperinsulinemia are crucial factors in the development of PCOS. Excessive insulin, in combination with LH, enhances the production of androgens by ovarian theca cells (58). Additionally, it reduces the production of sex hormone-binding globulin in the liver, together with the excess of androgens (59). The cause of the reduced insulin sensitivity in PCOS is not yet fully understood. However, it is believed that the different genetic and epigenetic abnormalities contribute to impairments in the production and function of the main glucose transporter in cells, known as glucose transporter 4 (GLUT4), as well as impairments in the disposal of glucose through insulin. Patients with PCOS also exhibit abnormalities in insulin-mediated lipolysis. Furthermore, the level of insulin resistance in PCOS is exacerbated by a condition of persistent mild inflammation, partly caused by aberrant production and function of adipocytokines. On the other hand, there is stronger evidence indicating that women with PCOS have adipose tissue that shows different defects that promote an inflammatory or insulin resistant condition. These defects include dysfunction in adipocytokines, dysregulation of free fatty acid metabolism, and abnormalities in epigenetics that affect GLUT4 function (57). Further investigation is required to rule out or confirm clinical evaluation using more targeted hormonal testing. Thyroid problems, hyperprolactinemia, and nonclassic adrenal hyperplasia are the main conditions to consider. These can be ruled out by measuring thyroid-stimulating hormone, prolactin, and 17-hydroxyprogesterone, respectively. Nonclassic adrenal hyperplasia caused by abnormalities in CYP21A2 affects a percentage ranging from 1 to 10% of women with excessive hair growth, depending on their ethnic background. This condition is the most prevalent autosomal-recessive ailment in the human population. Nonclassic adrenal hyperplasia caused by abnormalities in CYP21A2 affects a percentage ranging from 1 to 10% of women with excessive hair growth, depending on their ethnic background. This condition is the most prevalent autosomal-recessive ailment in the human population. Additional evidence indicates that promptly identifying the condition and administering corticosteroid treatment may enhance the chances of achieving a successful reproductive outcome (60). Therefore, it is recommended to evaluate any woman exhibiting signs, symptoms, or complaints of hyperandrogenism, regardless of severity, for nonclassic adrenal hyperplasia. Practitioners must be aware that it is not feasible to clinically identify or even make an educated guess about the diagnosis of nonclassic adrenal hyperplasia. It is absolutely necessary to evaluate the levels of 17-hydroxyprogesterone (61).

There are multiple benefits to our study. As far as we know, this is the initial MR investigation conducted to assess the causal connection between metabolites and REDs. Our current focus is on conducting exploratory research with the specific aim of enhancing our ability to support future metabolomics studies and offering valuable insights for future endeavors. Metabolomics is a scientific discipline that seeks to understand how living systems react to genetic alterations, environmental shifts, or disease conditions by examining the entire collection of metabolites within a specific biological setting, providing a precise representation of the biochemical processes occurring at the moment of sample collection (62). By integrating advanced approaches such as extracellular vesicles, liquid biopsies, and single-cell metabolomics with our understanding of metabolites, we can further refine our analyses, potentially leading to early diagnosis and targeted early treatment of REDs (63–66). Thus, we anticipate that our study can guide the analysis of metabolites and genomes, enabling researchers to focus on fundamental experiments and develop more effective therapeutic strategies.

But there also are certain constraints that need to be addressed. First, identifying genetic variations that are strongly linked to specific metabolites and can serve as instrumental factors can be a complex task. Metabolites can be altered by a combination of genetic factors and environmental conditions. These genetic variants account for only a minor fraction of the variability in the metabolites, which may result in a slight instrument bias and diminish the statistical power of the study. The chosen genetic variants may impact the likelihood of female reproductive endocrine disorders through biological pathways that are not connected to the hypothesis, so contravening the third postulate of MR. Pleiotropy may introduce a bias in the estimated causal effect. Second, the study sample consists of individuals with varied genetic backgrounds. Failing to effectively adjust for population stratification could result in false relationships. This poses a significant challenge when the correlation between genetic variations and metabolites differs among different populations. The genetic variations utilized are predominantly derived from specific groups with European heritage, hence the findings may not be generalizable to individuals with different genetic backgrounds. Therefore, the study’s capacity to be applied to a wider population may be restricted. Third, if there is a potential for bidirectional causation between specific metabolites and female reproductive endocrine problems, relying solely on MR may not provide an accurate determination of the actual causal relationship. Furthermore, inaccuracies in detecting metabolite levels may introduce bias in the assessment of causal links, particularly if the measurements of metabolites are inaccurate or affected by batch effects. If individuals in the sample are chosen or omitted based on their disease status or other specific criteria, the study results may not be applicable to the wider population. Subsequent metabolomics investigations should include provisions to tackle these constraints.

This pioneering study marks a significant advancement in understanding the complex interplay between metabolites and RED from a metabolomics standpoint. Through meticulous MR analysis, we’ve unveiled specific associations across various metabolite classes—including amino acids, carbohydrates, lipids, nucleotides, and peptides—with conditions like PCOS, EMs, and FI. These insights not only deepen our comprehension of RED’s metabolic foundations but also herald new avenues for diagnosis and treatment. The identification of metabolites as potential biomarkers offers promising pathways for early detection and risk assessment, enabling more precise and personalized management strategies. Furthermore, elucidating these causal relationships opens up possibilities for targeted interventions aimed at correcting metabolic imbalances, thereby offering hope for improved outcomes. The diversity of implicated metabolites underscores the necessity for a personalized medicine approach, tailoring treatments to individual metabolic dysregulations to optimize care. Consequently, this research not only contributes significantly to our understanding of reproductive health but also paves the way for transformative developments in the diagnosis, management, and treatment of reproductive endocrine disorders, spotlighting the critical role of metabolomics in advancing reproductive medicine.

## 5 Conclusion

Our work employed a multivariate two-sample MR analysis using publicly accessible GWAS meta-analysis data to examine the causal connection between metabolites and several RED, such as PCOS, EMs and FI. Ultimately, we conducted a thorough assessment of the potential correlation between the metabolites and REDs. These metabolites and genes could potentially serve as biomarkers and offer valuable insights for future studies on treatment.

## Data Availability

The datasets presented in this study can be found in online repositories. The names of the repository/repositories and accession number(s) can be found in the article/**Supplementary Material**.

## Supplementary material

Supplementary Table 1 Comprehensive information of all SNPs associated with female infertility, polycystic ovary syndrome, and endometriosis.

Supplementary Table 2 Detailed annotations for all single nucleotide polymorphisms employed as instrumental variables.

Supplementary Figure 1 Scatter plot. This scatter plot depicts the correlation between the genetic associations of instrumental variables and the risk of reproductive endocrine disorders, providing a snapshot of the causal effects estimated by MR.

Supplementary Figure 2 Funnel plot. The funnel plot visualizes the distribution of effect estimates from instrumental variables against their precision, assessing potential publication bias and asymmetry in the MR analysis for metabolic markers related to reproductive disorders.

Supplementary Figure 3 Forest plot. The forest plot aggregates the effect sizes and confidence intervals for each metabolite’s impact on reproductive health outcomes, facilitating a meta-analytic view of their significance and heterogeneity.

Supplementary Figure 4 Leave-one-out plot. The leave-one-out plot examines the influence of each individual instrumental variable on the overall MR results, ensuring the robustness and reliability of the associations with female infertility, polycystic ovary syndrome, and endometriosis.
