# Supplementary material for: Investigating the metabolomic pathways in female reproductive endocrine disorders: a Mendelian randomization study
Source: Front Endocrinol (Lausanne). 2024 Oct 31;15:1438079. doi: 10.3389/fendo.2024.1438079 (PMC11560792; doi:10.3389/fendo.2024.1438079)
Supplement: Supplementary Figure 1 — Scatter plot. This scatter plot depicts the correlation between the genetic associations of instrumental variables and the risk of reproductive endocrine disorders, providing a snapshot of the causal effects estimated by MR. [file Image1.pdf]

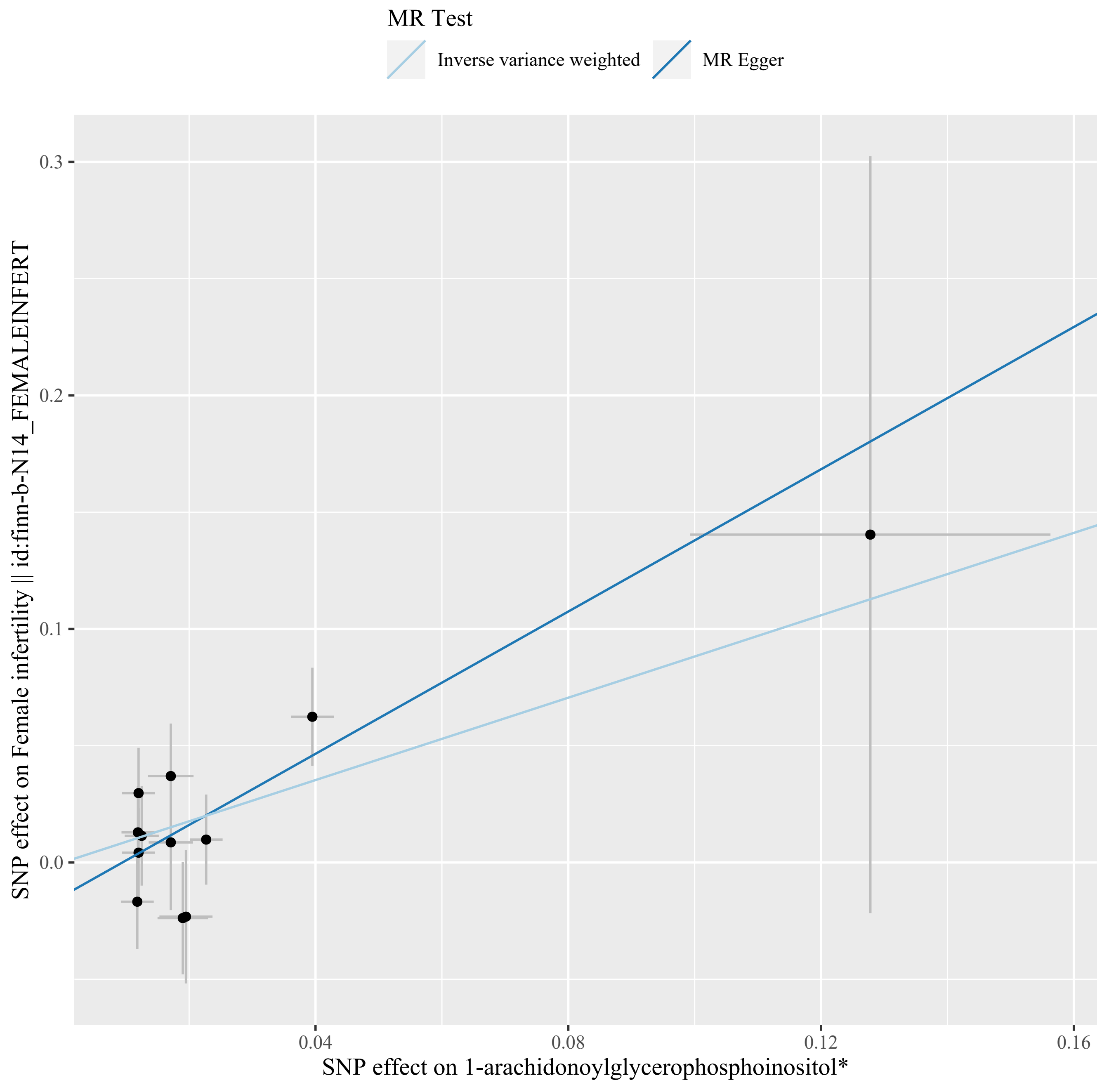
MR Test
Inverse variance weighted
MR Egger
SNP effect on Female infertility || id:finn-b-N14_FEMALEINFERT
0.3
0.2
0.1
0.0
0.04
0.08
0.12
0.16
SNP effect on 1-arachidonoylglycerophosphoinositol*

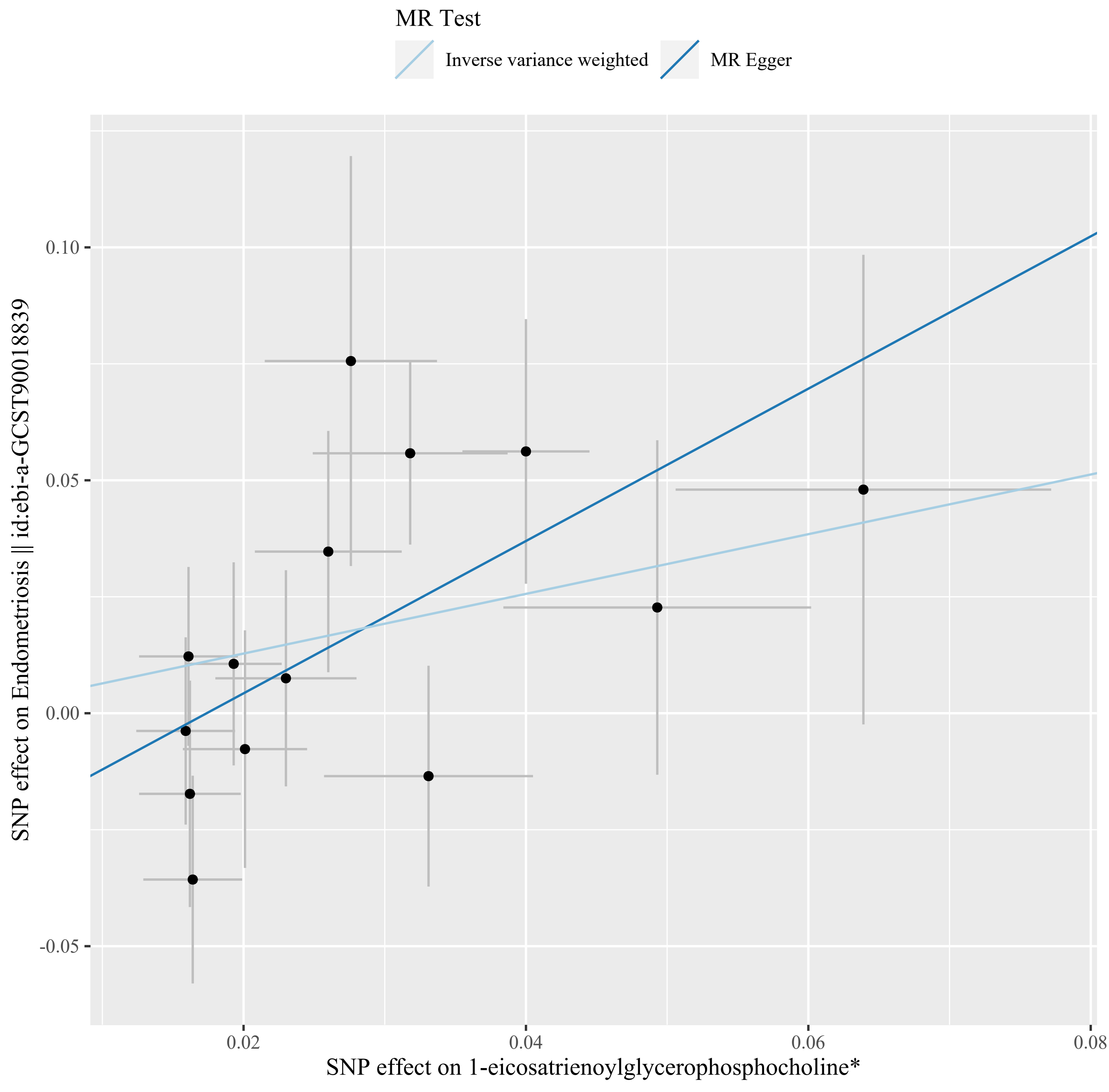
MR Test
Inverse variance weighted
MR Egger
0.10
0.05
0.00
-0.05
0.02
0.04
0.06
0.08
SNP effect on Endometriosis || id:ebi-a-GCST90018839
SNP effect on 1-eicosatrienoylglycerophosphocholine*

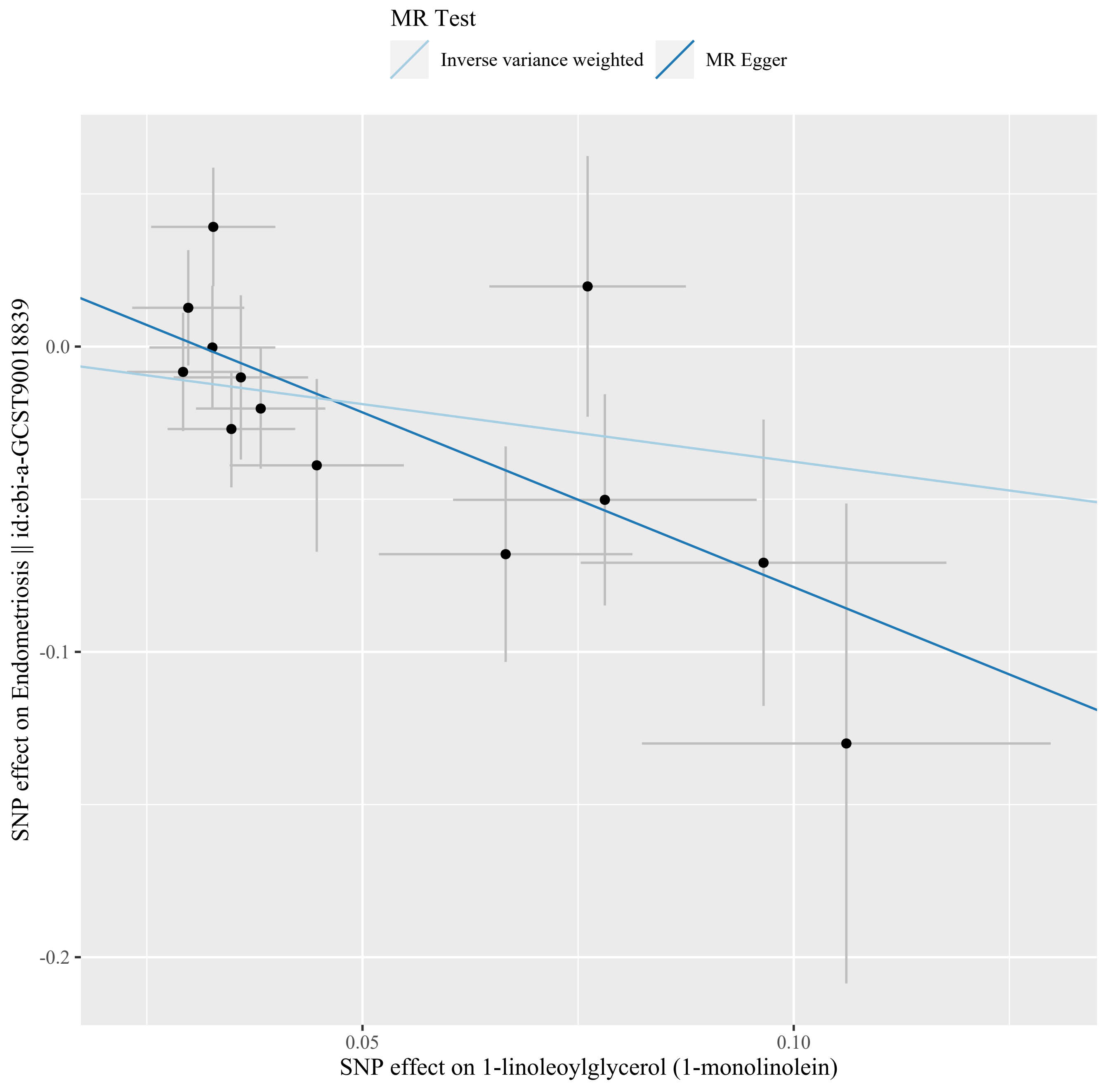
MR Test
Inverse variance weighted
MR Egger
SNP effect on Endometriosis || id:ebi-a-GCST90018839
0.0
-0.1
-0.2
0.05
0.10
SNP effect on 1-linoleoylglycerol (1-monolinolein)

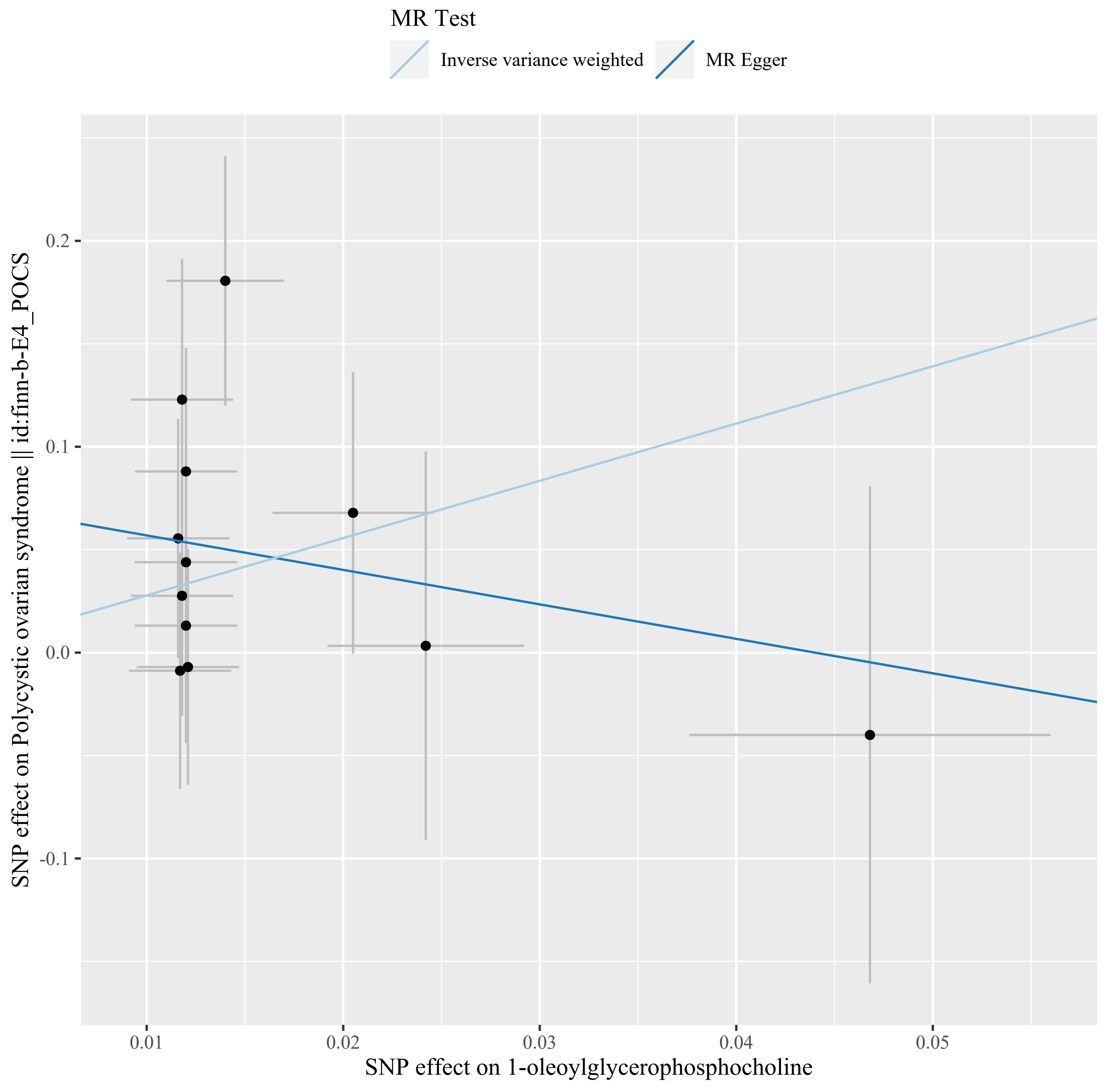
MR Test
Inverse variance weighted
MR Egger
SNP effect on Polycystic ovarian syndrome || id:finn-b-E4_POCS
SNP effect on 1-oleoylglycerophosphocholine
0.2
0.1
0.0
-0.1
0.01
0.02
0.03
0.04
0.05

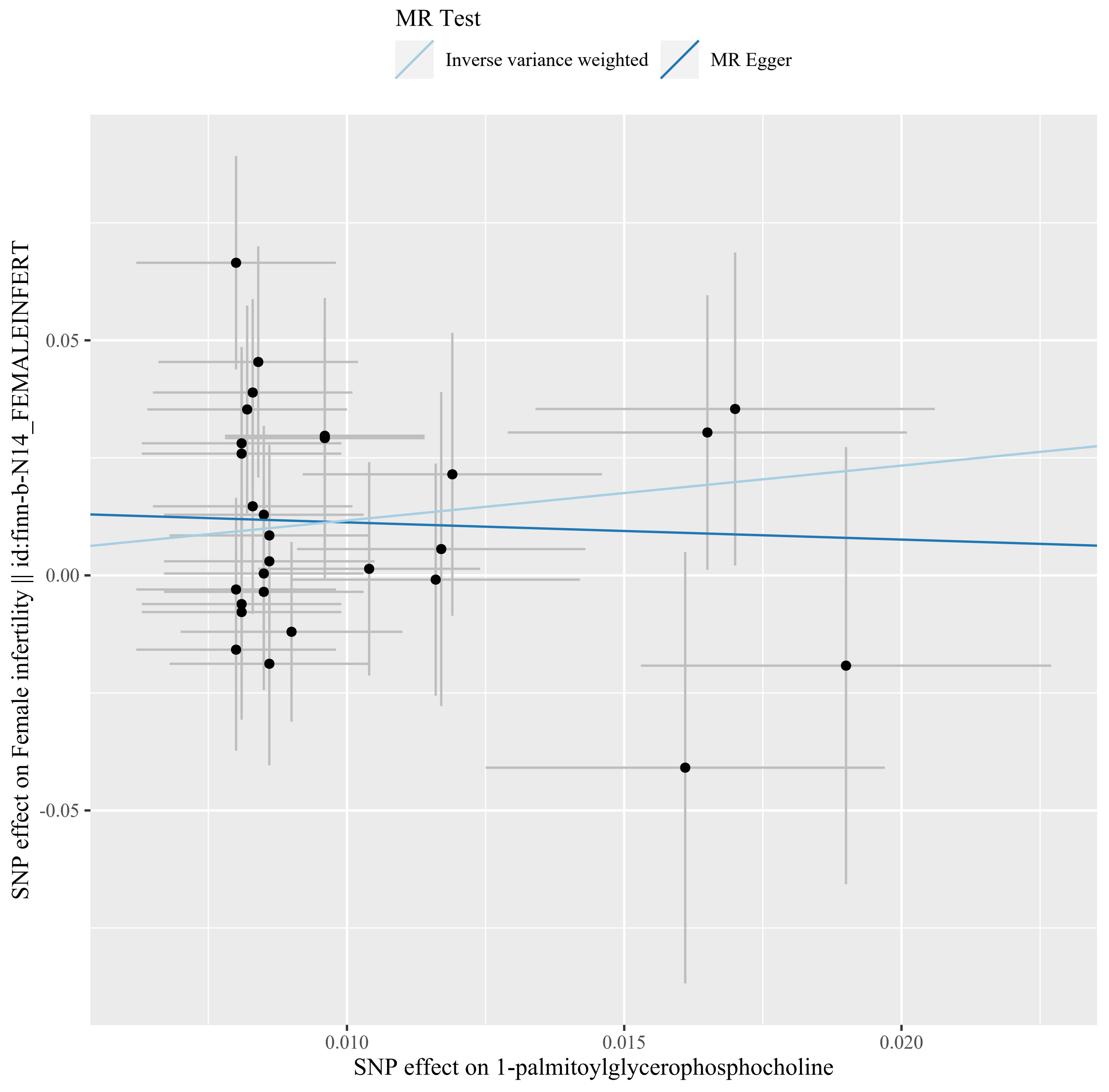

MR Test
Inverse variance weighted
MR Egger
0.05
0.00
-0.05
0.010
0.015
0.020
SNP effect on Female infertility || id:finn-b-N14_FEMALEINFERT
SNP effect on 1-palmitoylglycerophosphocholine

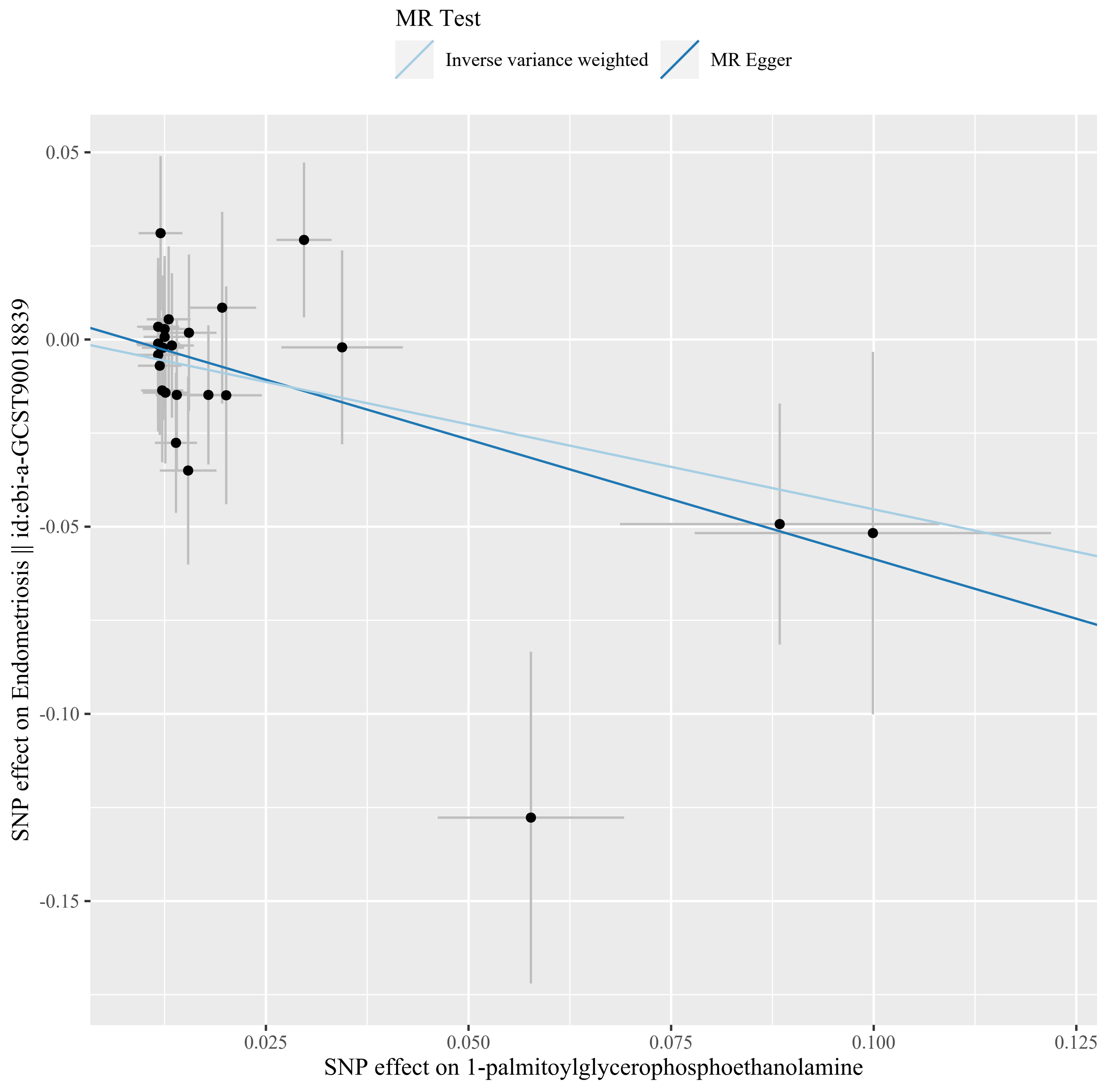
MR Test
Inverse variance weighted
MR Egger
0.05
0.00
-0.05
-0.10
-0.15
0.025
0.050
0.075
0.100
0.125
SNP effect on Endometriosis || id:ebi-a-GCST90018839
SNP effect on 1-palmitoylglycerophosphoethanolamine

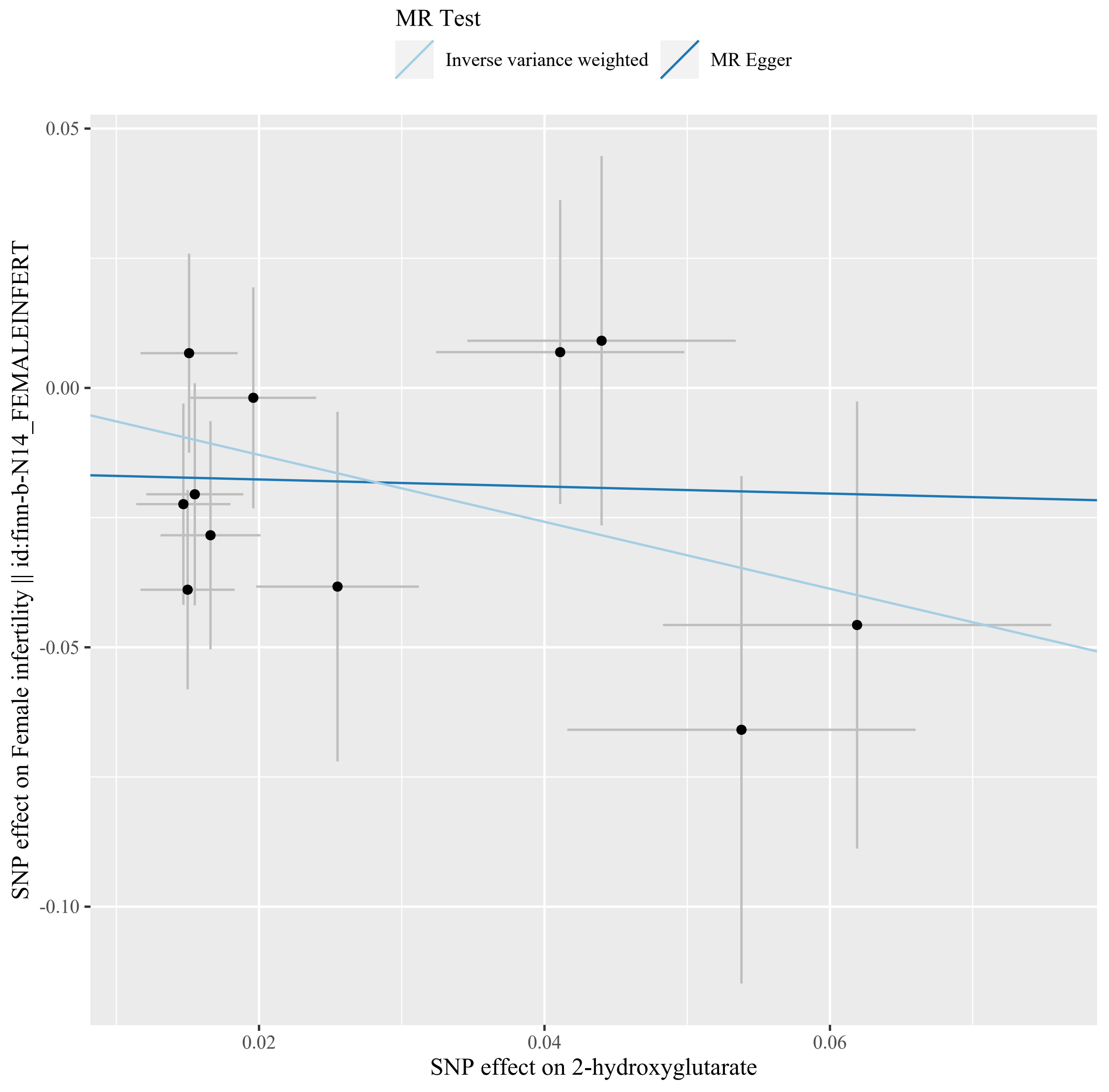

MR Test
Inverse variance weighted
MR Egger
0.05
0.00
-0.05
-0.10
SNP effect on Female infertility || id:finn-b-N14_FEMALEINFERT
0.02
0.04
0.06
SNP effect on 2-hydroxyglutarate

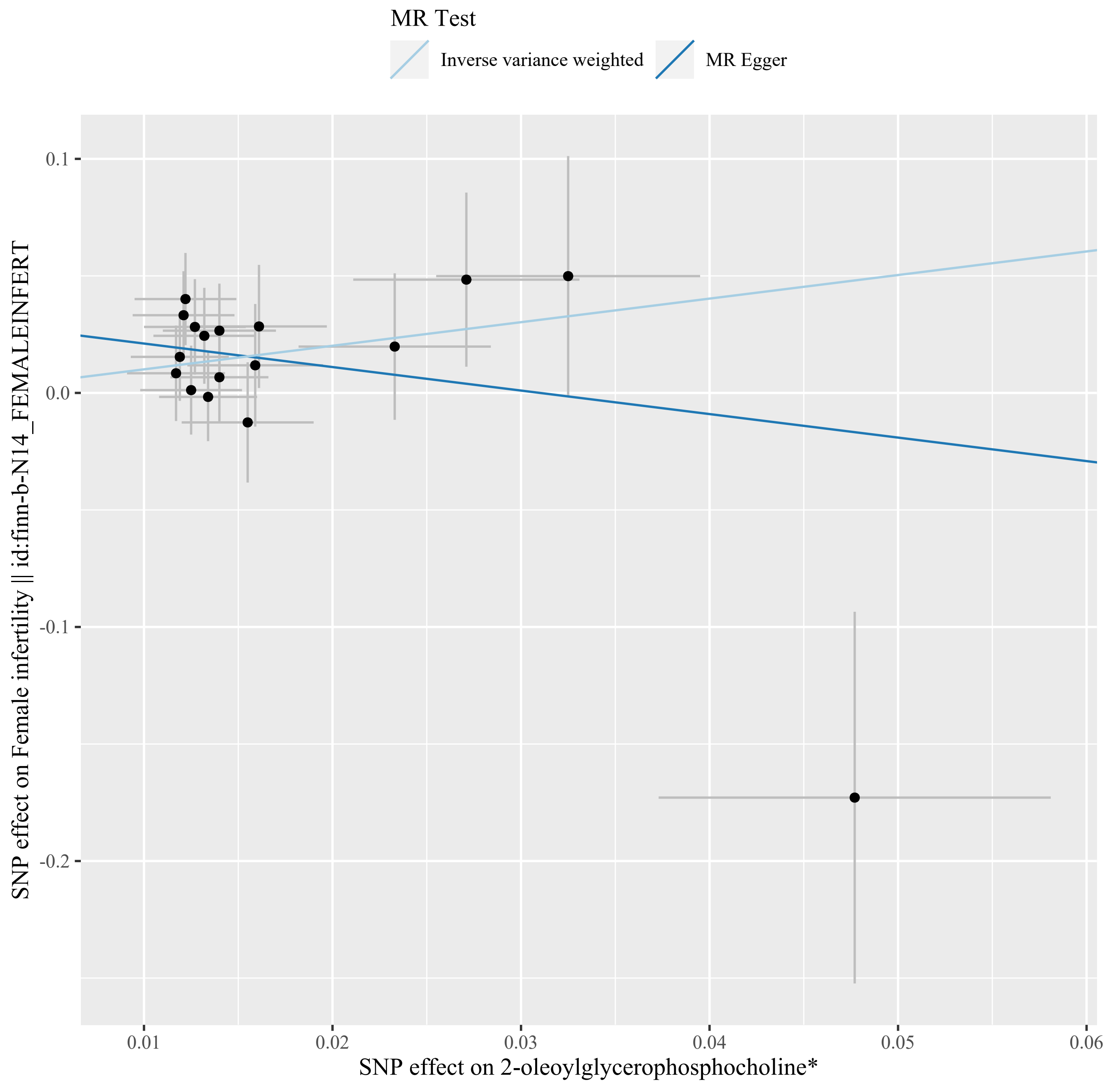
MR Test
Inverse variance weighted
MR Egger
0.1
0.0
-0.1
-0.2
0.01
0.02
0.03
0.04
0.05
0.06
SNP effect on Female infertility || id:finn-b-N14_FEMALEINFERT
SNP effect on 2-oleoylglycerophosphocholine*

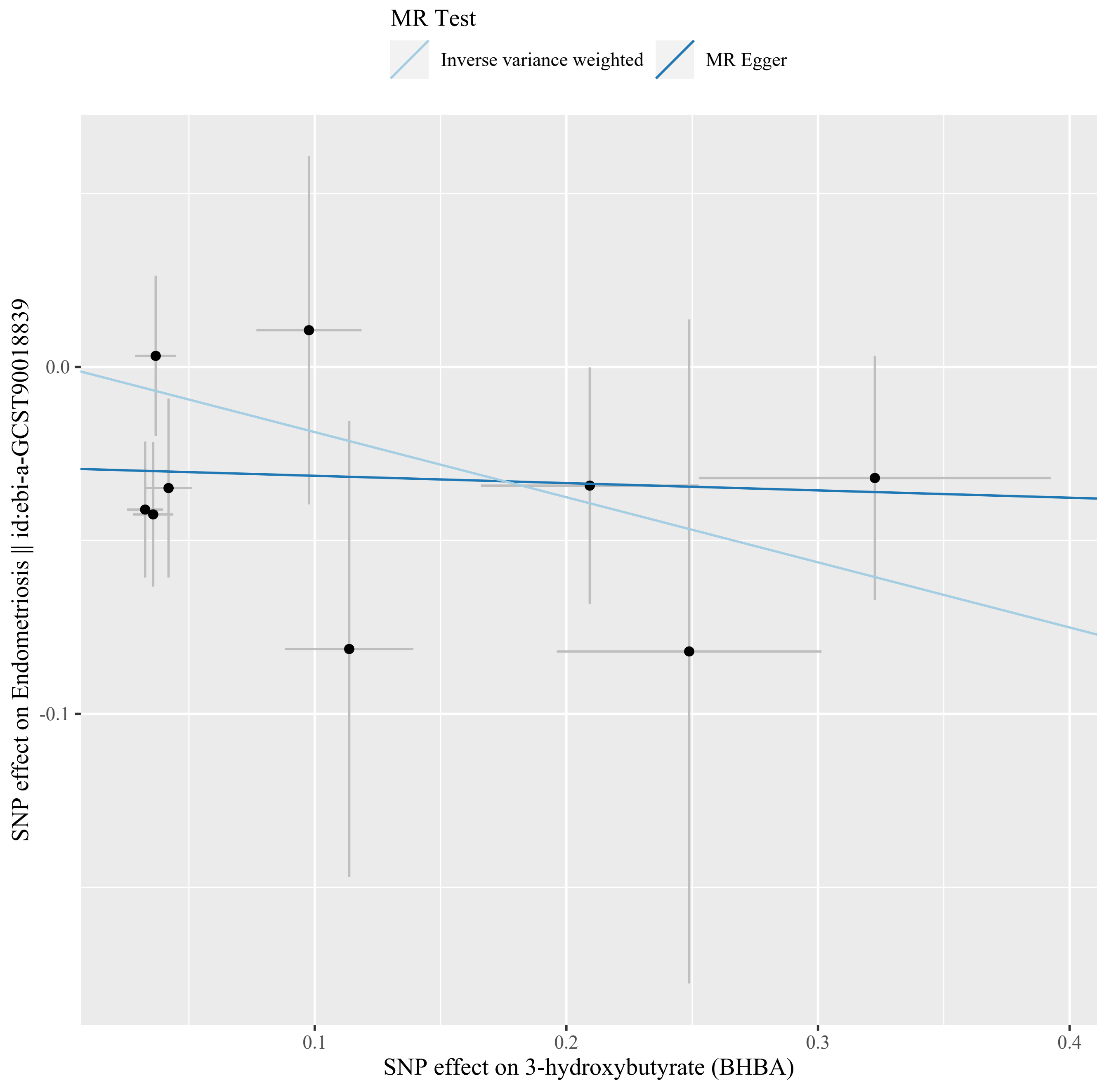

MR Test
Inverse variance weighted
MR Egger
0.0
-0.1
0.1
0.2
0.3
0.4
SNP effect on Endometriosis || id:ebi-a-GCST90018839
SNP effect on 3-hydroxybutyrate (BHBA)

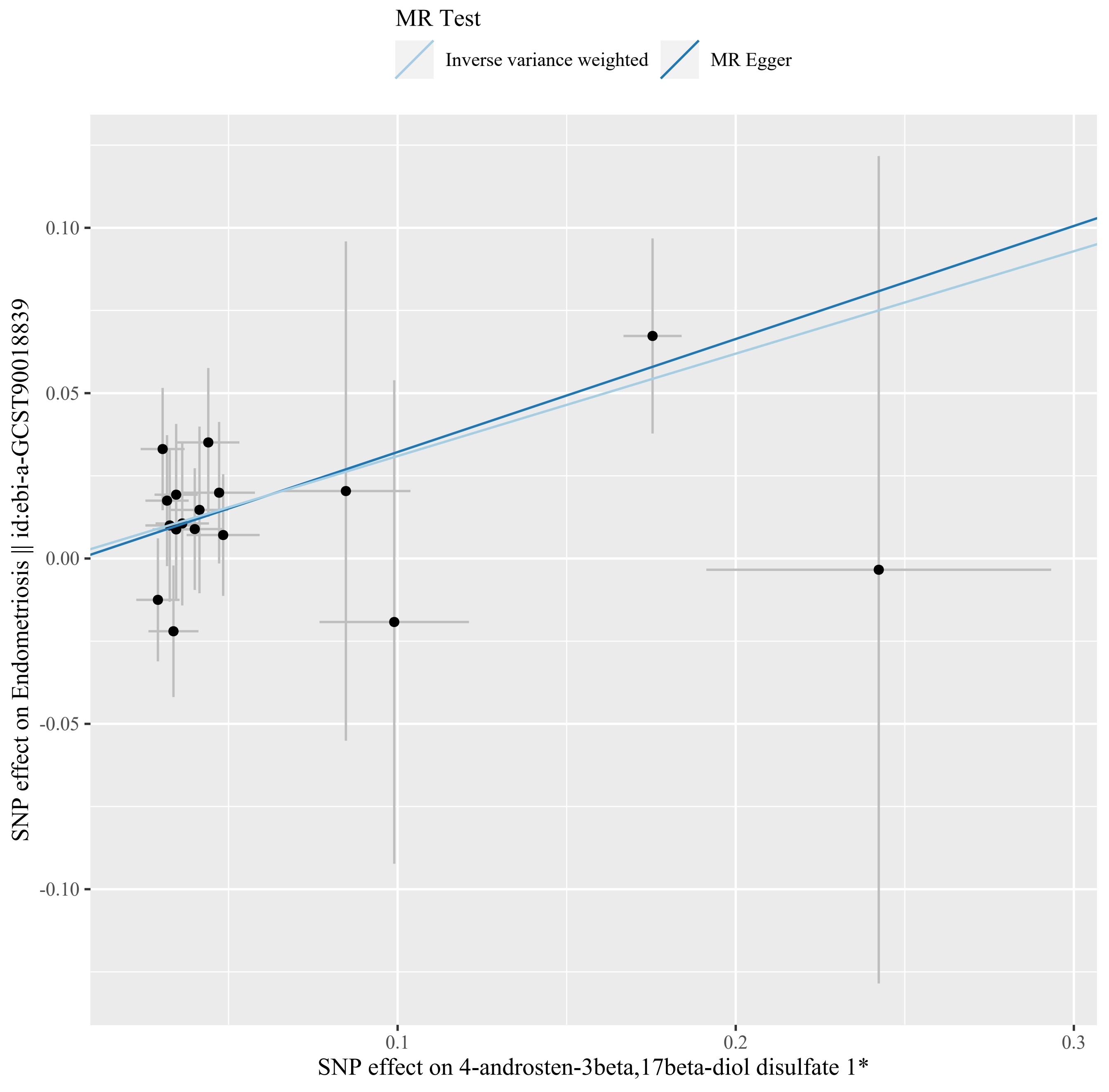
MR Test
Inverse variance weighted
MR Egger
SNP effect on Endometriosis || id:ebi-a-GCST90018839
0.10
0.05
0.00
-0.05
-0.10
0.1
0.2
0.3
SNP effect on 4-androsten-3beta,17beta-diol disulfate 1*

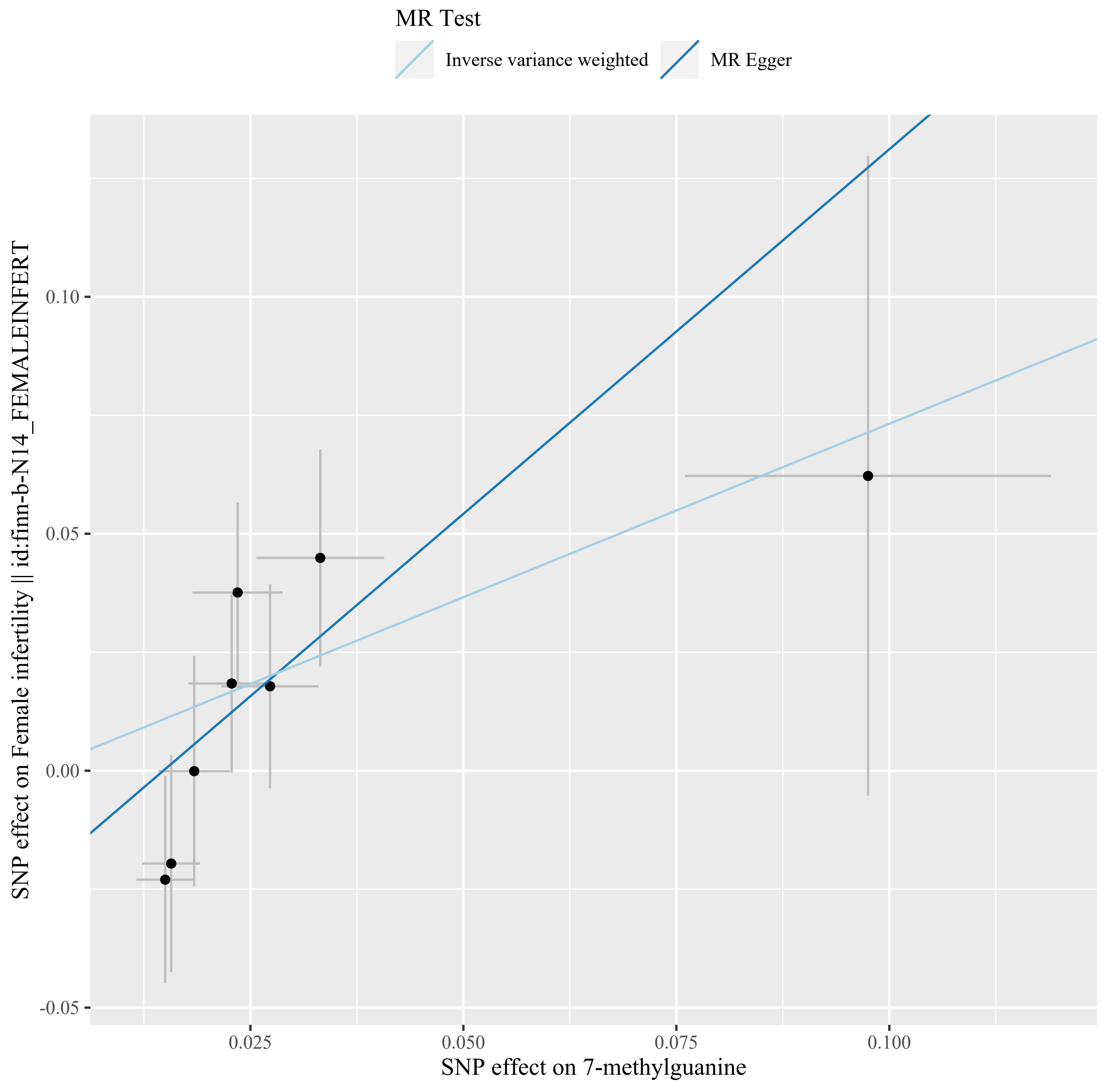

MR Test
Inverse variance weighted
MR Egger
0.10
0.05
0.00
-0.05
0.025
0.050
0.075
0.100
SNP effect on 7-methylguanine
SNP effect on Female infertility || id:finn-b-N14_FEMALEINFERT

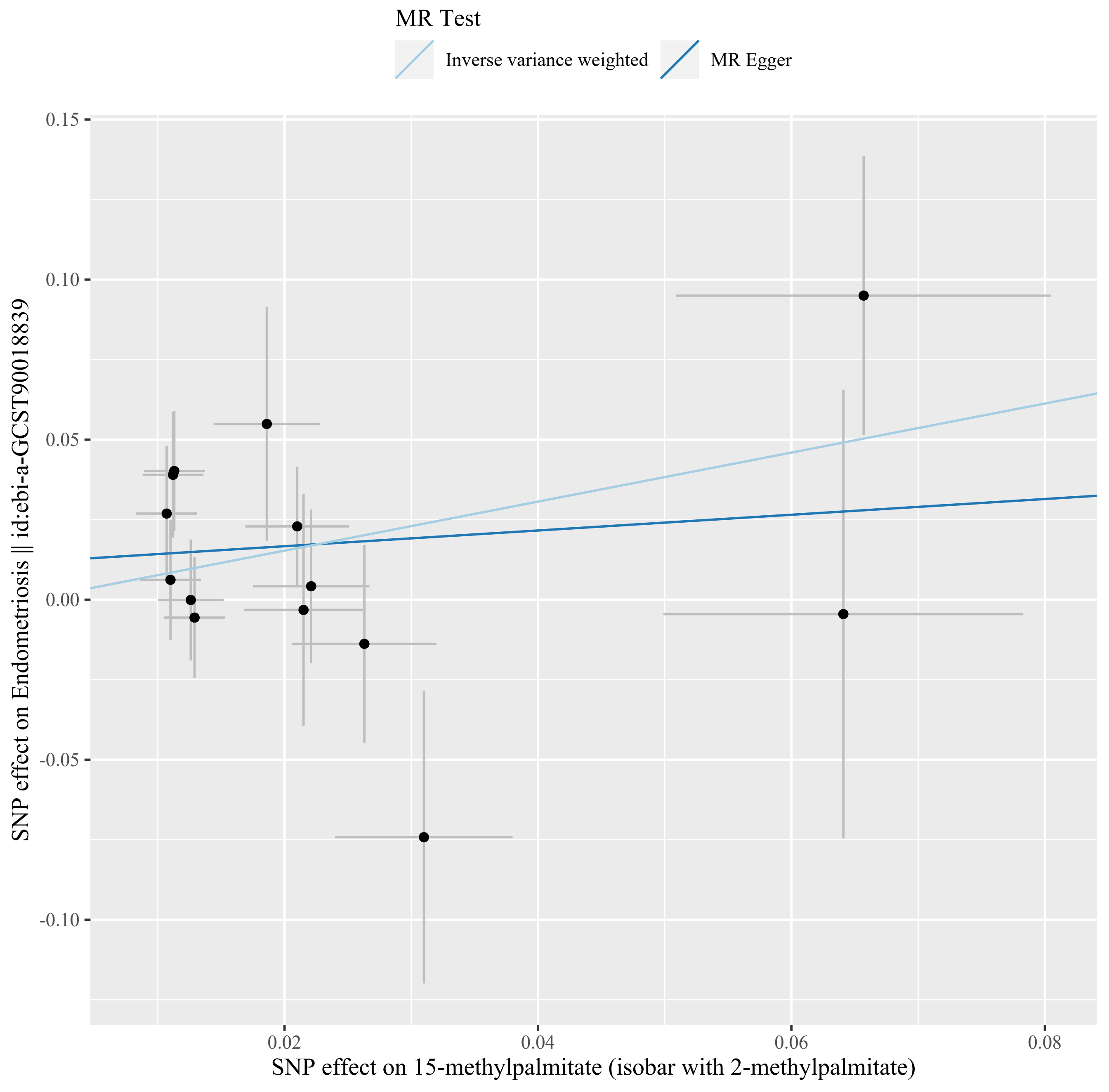
MR Test
Inverse variance weighted
MR Egger
0.15
0.10
0.05
0.00
-0.05
-0.10
SNP effect on Endometriosis || id:ebi-a-GCST90018839
0.02
0.04
0.06
0.08
SNP effect on 15-methylpalmitate (isobar with 2-methylpalmitate)

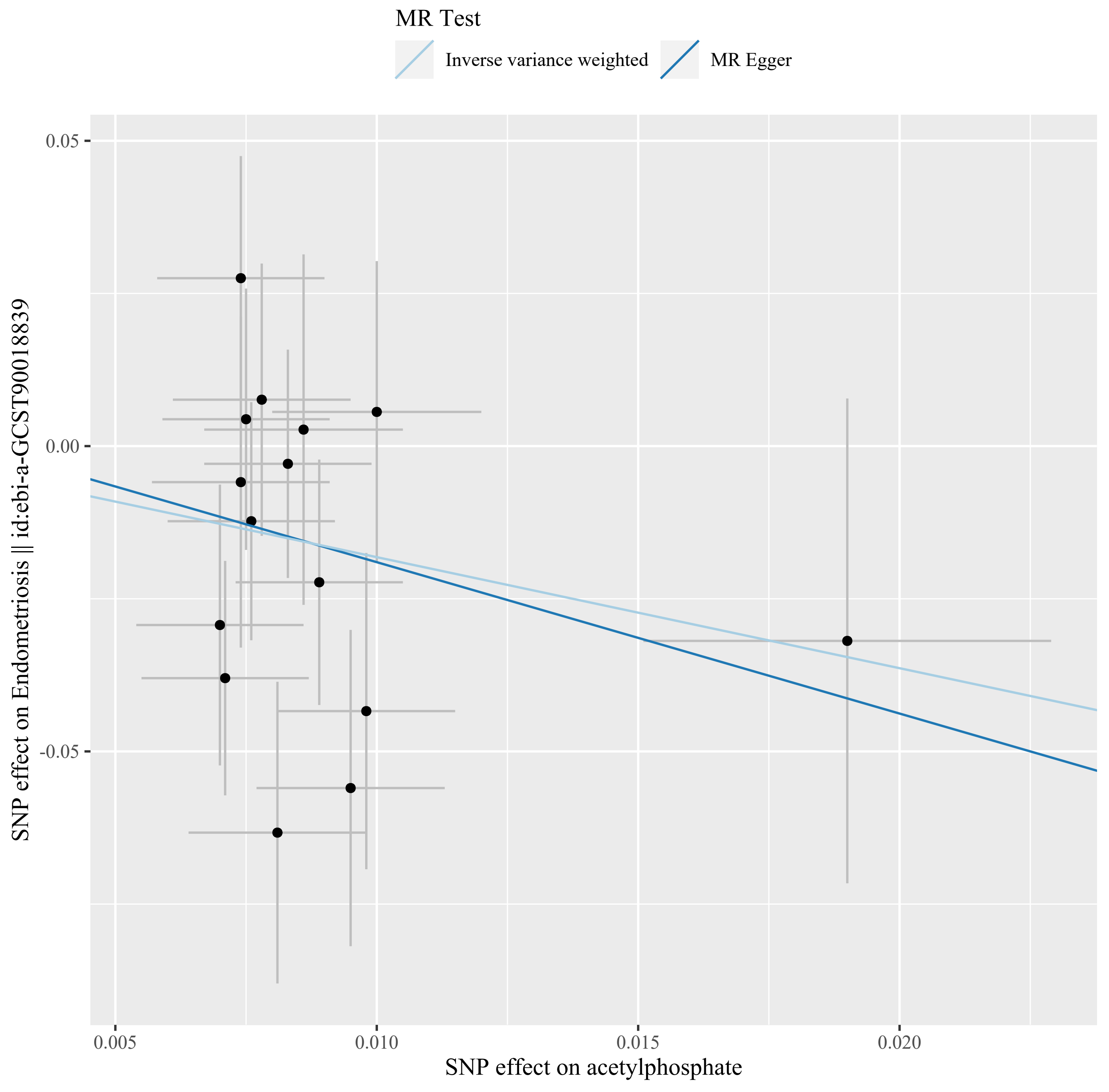
MR Test
Inverse variance weighted
MR Egger
0.05
0.00
-0.05
SNP effect on Endometriosis || id:ebi-a-GCST90018839
0.005
0.010
0.015
0.020
SNP effect on acetylphosphate

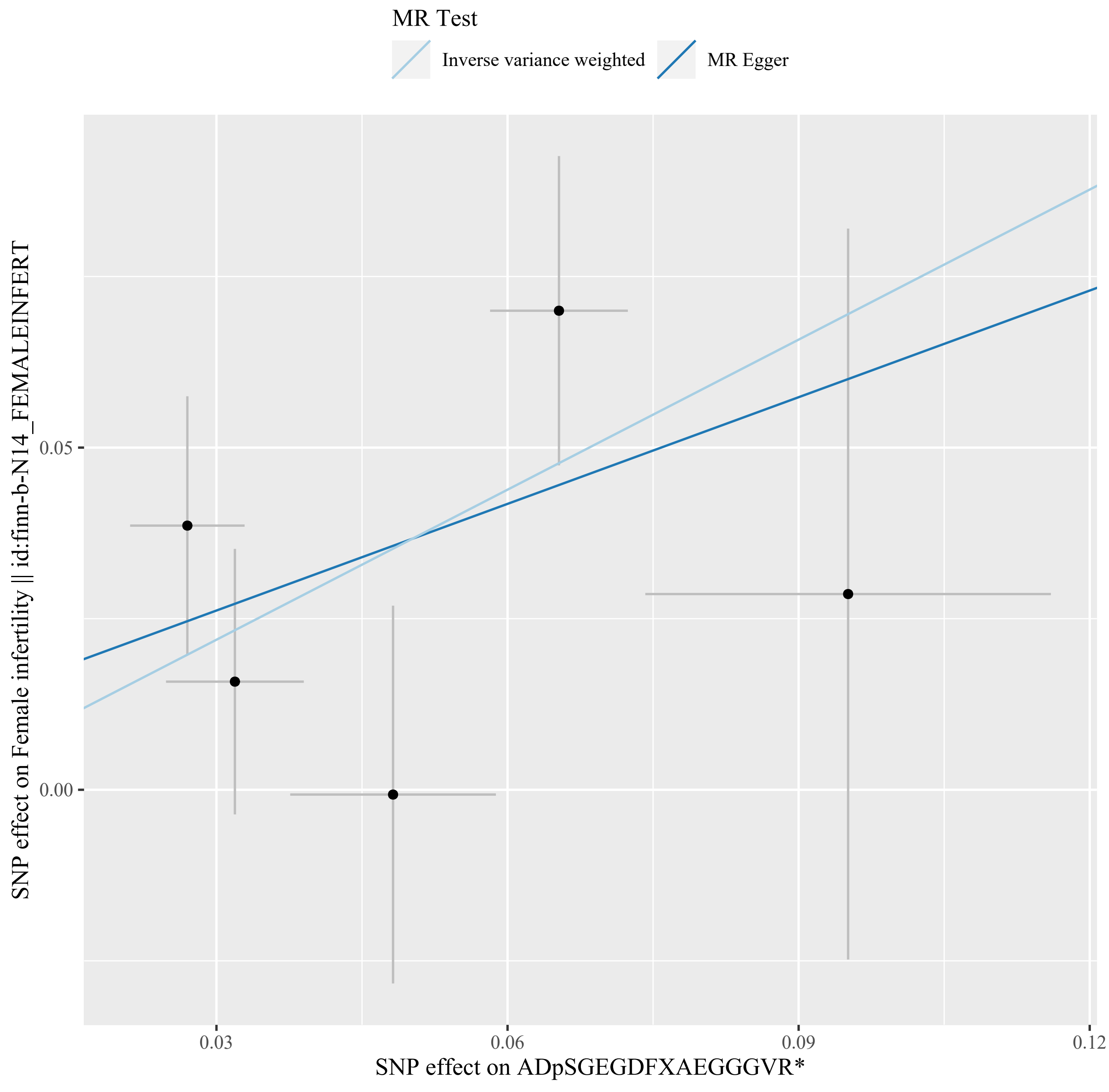

MR Test
Inverse variance weighted
MR Egger
SNP effect on Female infertility || id:finn-b-N14_FEMALEINFERT
0.05
0.00
0.03
0.06
0.09
0.12
SNP effect on ADpSGEGDFXAEGGGVR*

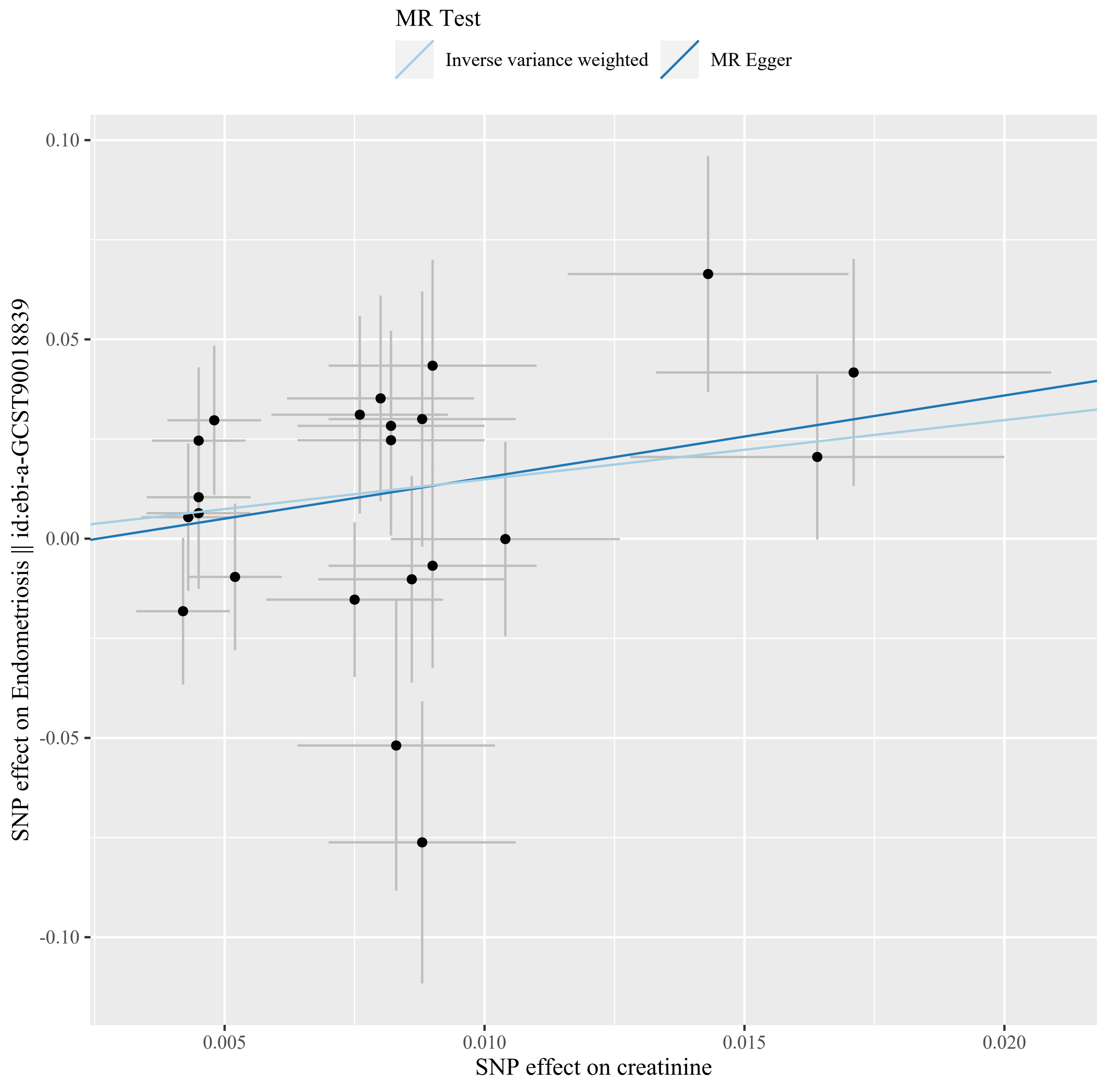
MR Test
Inverse variance weighted
MR Egger
0.10
0.05
0.00
-0.05
-0.10
SNP effect on Endometriosis || id:ebi-a-GCST90018839
0.005
0.010
0.015
0.020
SNP effect on creatinine

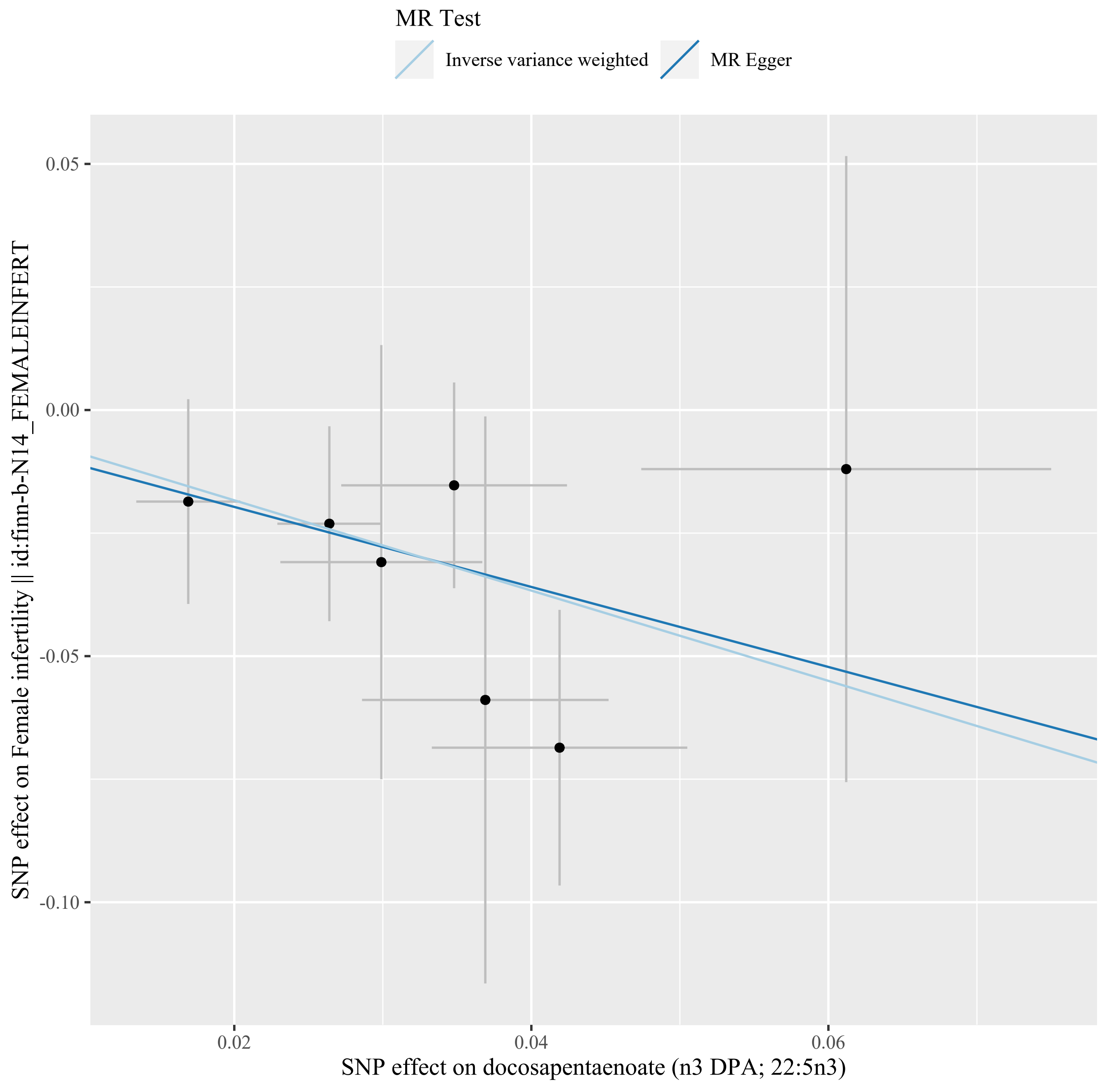
MR Test
Inverse variance weighted
MR Egger
0.05
0.00
-0.05
-0.10
SNP effect on Female infertility || id:finn-b-N14_FEMALEINFERT
0.02
0.04
0.06
SNP effect on docosapentaenoate (n3 DPA; 22:5n3)

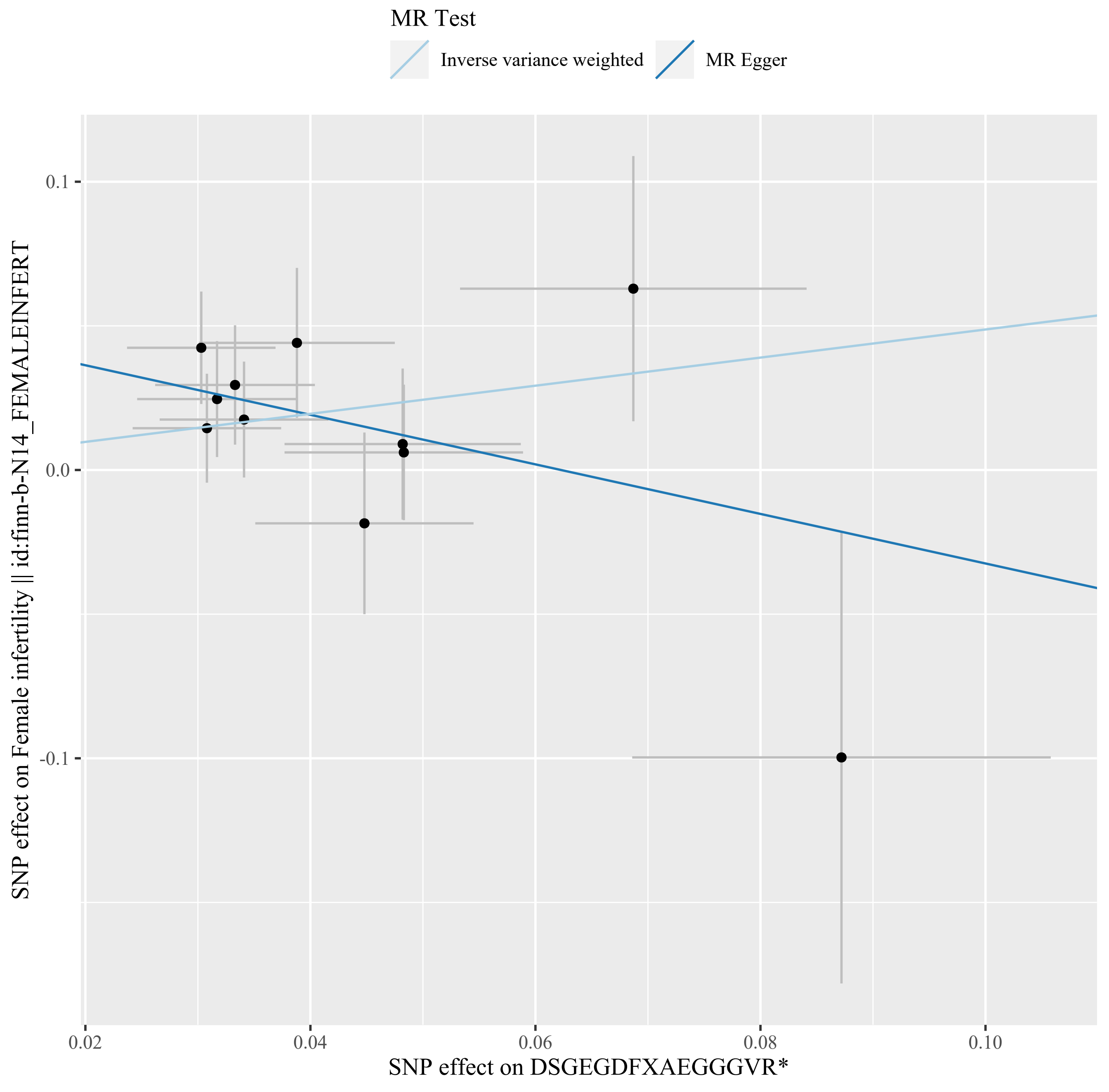
MR Test
Inverse variance weighted
MR Egger
0.1
0.0
-0.1
0.02
0.04
0.06
0.08
0.10
SNP effect on Female infertility || id:finn-b-N14_FEMALEINFERT
SNP effect on DSGEGDFXAEGGGVR*

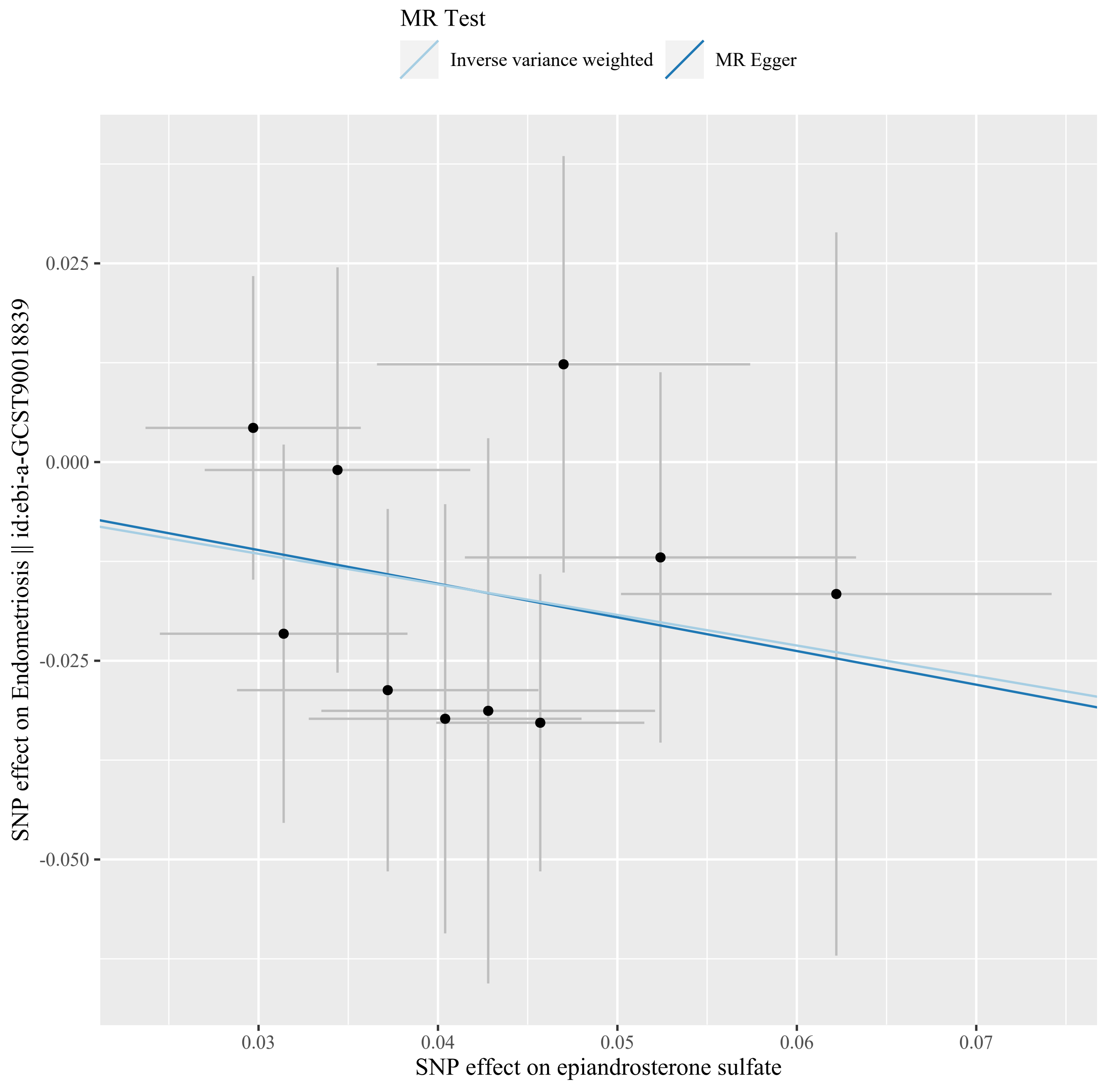
MR Test
Inverse variance weighted
MR Egger
0.025
0.000
-0.025
-0.050
SNP effect on Endometriosis || id:ebi-a-GCST90018839
0.03
0.04
0.05
0.06
0.07
SNP effect on epiandrosterone sulfate

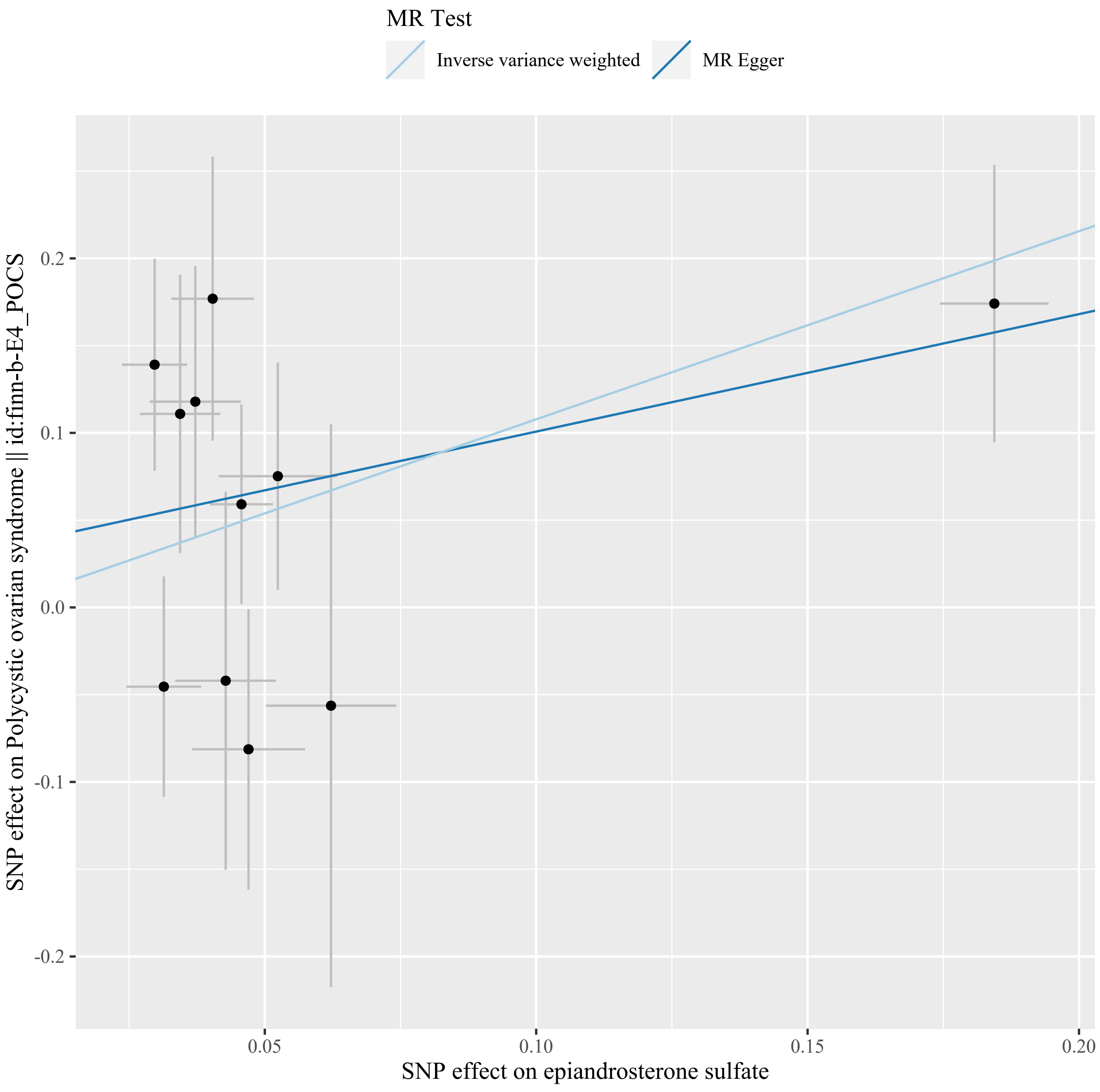
MR Test
Inverse variance weighted
MR Egger
SNP effect on Polycystic ovarian syndrome || id:finn-b-E4_POCS
0.2
0.1
0.0
-0.1
-0.2
0.05
0.10
0.15
0.20
SNP effect on epiandrosterone sulfate

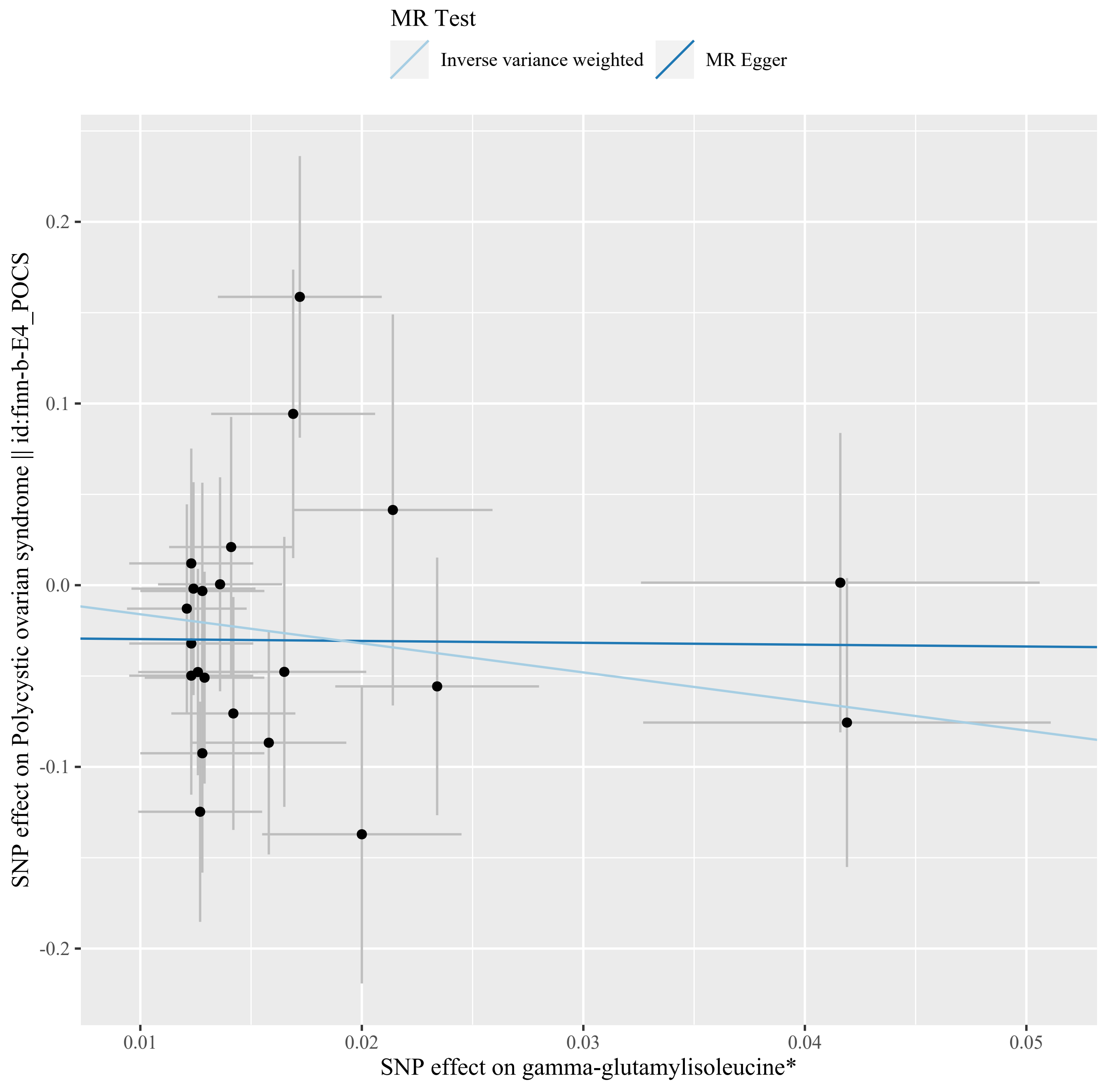

MR Test
Inverse variance weighted
MR Egger
0.2
0.1
0.0
-0.1
-0.2
0.01
0.02
0.03
0.04
0.05
SNP effect on Polycystic ovarian syndrome || id:finn-b-E4_POCS
SNP effect on gamma-glutamylisoleucine*

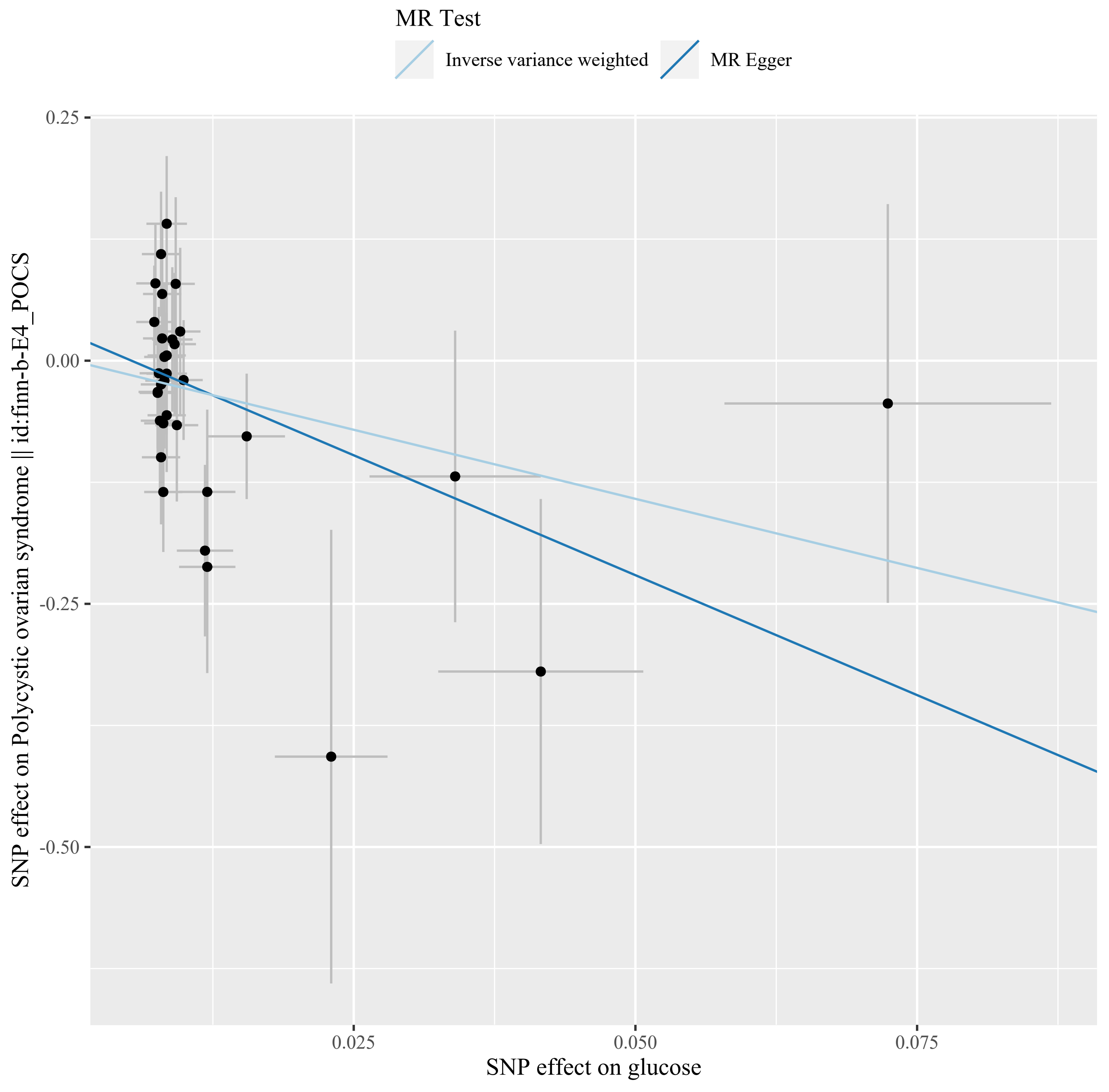
MR Test
Inverse variance weighted
MR Egger
0.25
0.00
-0.25
-0.50
0.025
0.050
0.075
SNP effect on Polycystic ovarian syndrome || id:finn-b-E4_POCS
SNP effect on glucose

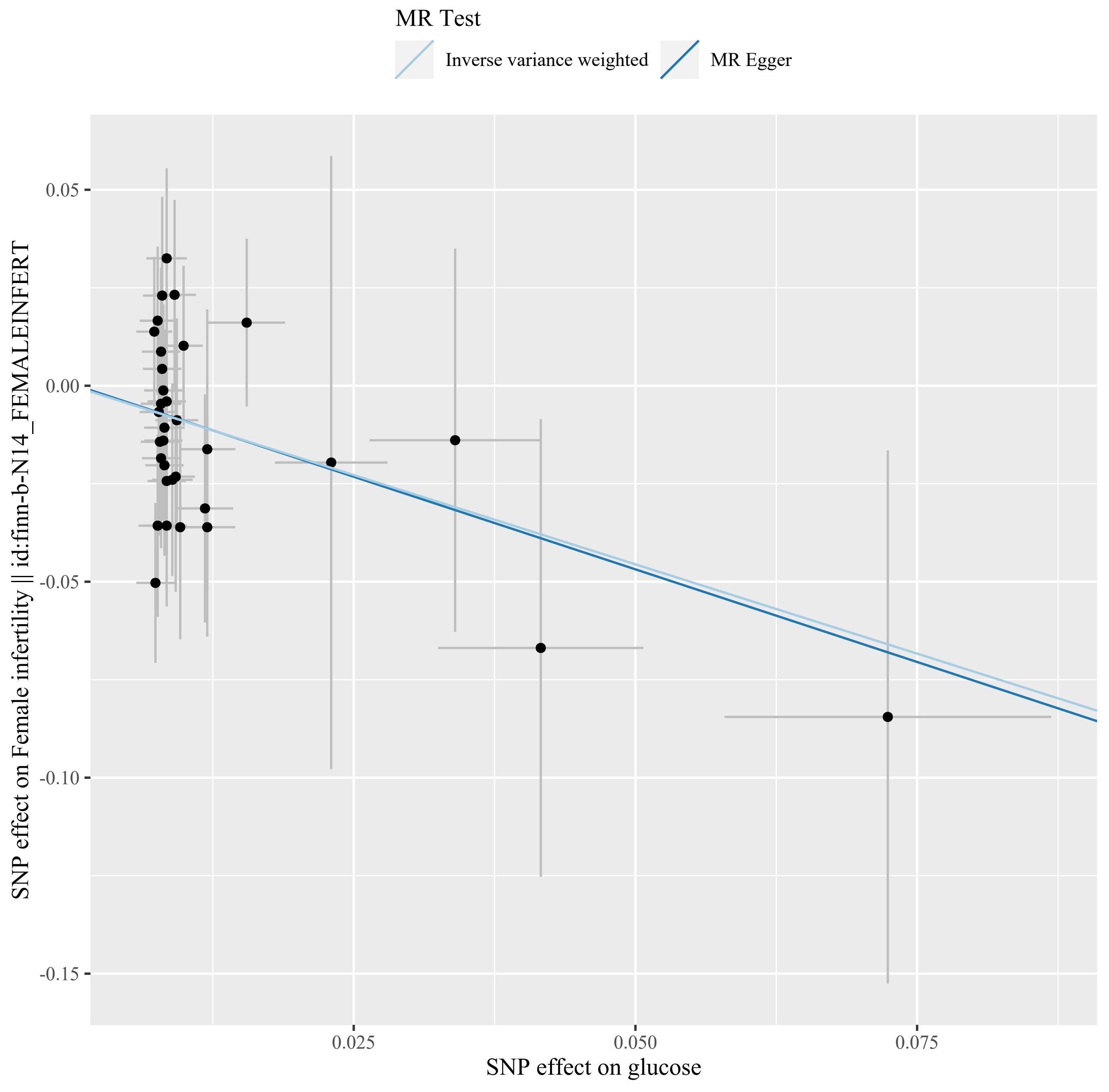

MR Test
Inverse variance weighted
MR Egger
0.05
0.00
-0.05
-0.10
-0.15
0.025
0.050
0.075
SNP effect on Female infertility || id:finn-b-N14_FEMALEINFERT
SNP effect on glucose

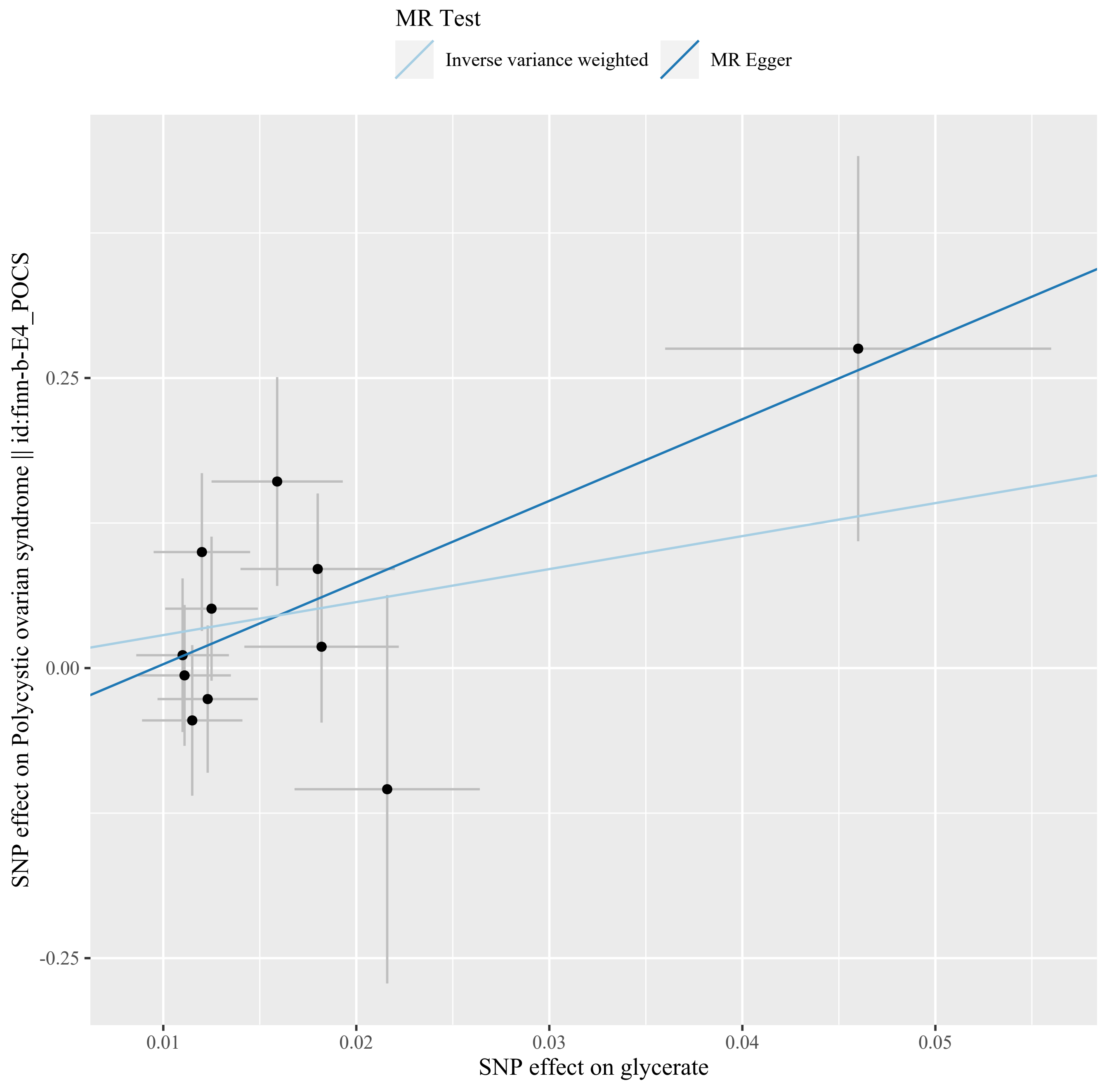
MR Test
Inverse variance weighted
MR Egger
0.25
0.00
-0.25
0.01
0.02
0.03
0.04
0.05
SNP effect on glycerate
SNP effect on Polycystic ovarian syndrome || id:finn-b-E4_POCS

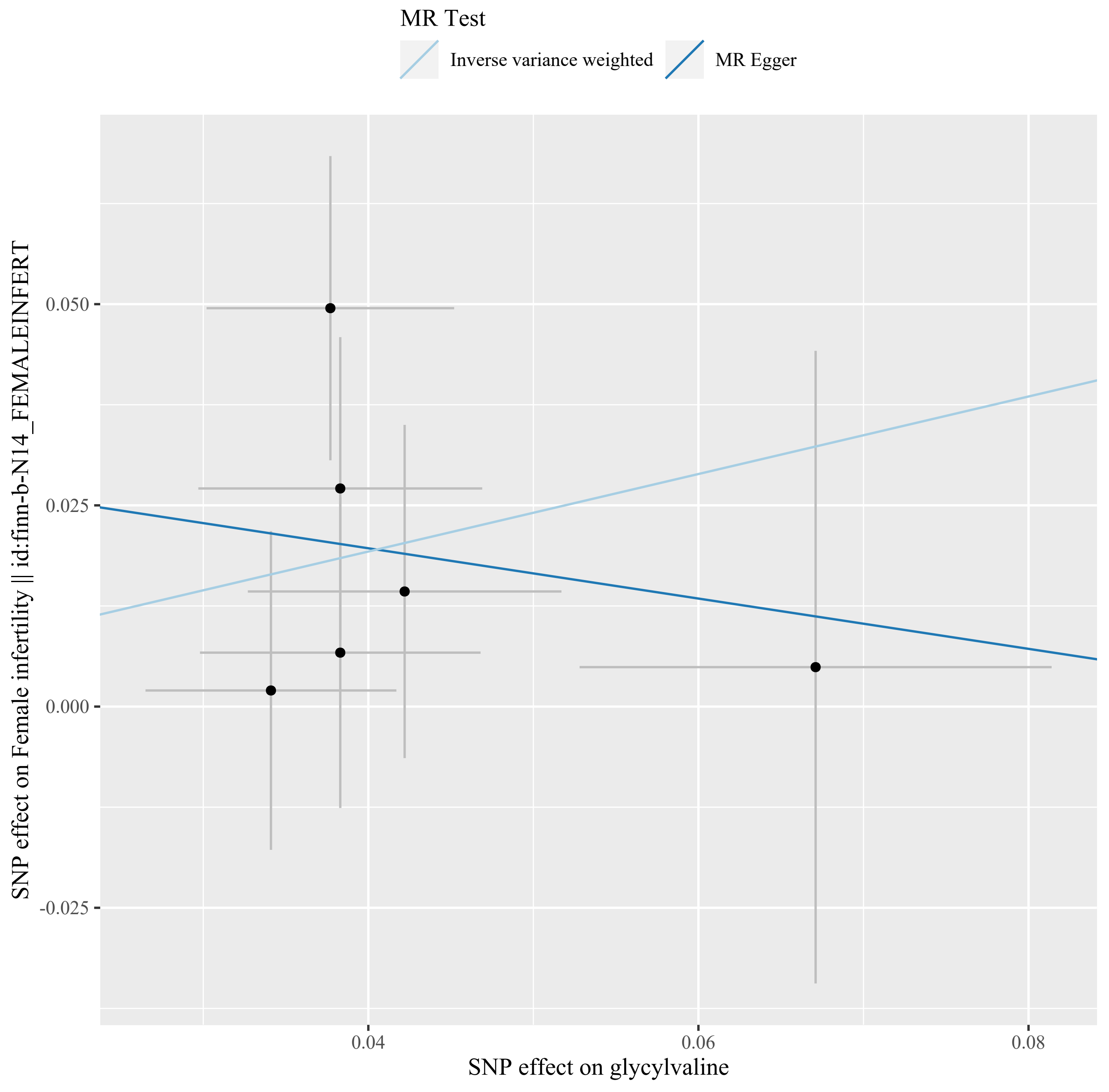
MR Test
Inverse variance weighted
MR Egger
SNP effect on Female infertility || id:finn-b-N14_FEMALEINFERT
0.050
0.025
0.000
-0.025
0.04
0.06
0.08
SNP effect on glycylvaline

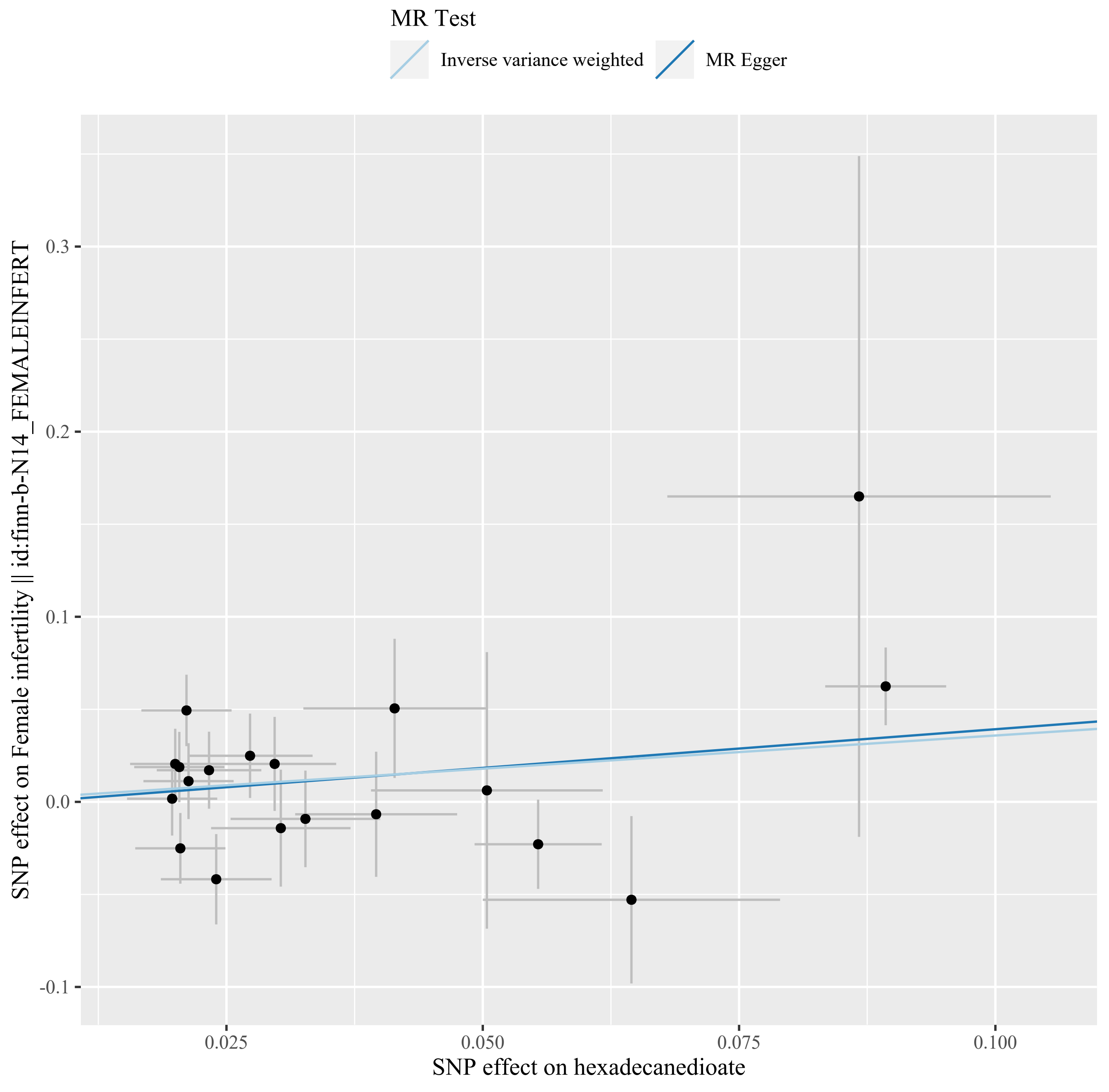
MR Test
Inverse variance weighted
MR Egger
SNP effect on Female infertility || id:finn-b-N14_FEMALEINFERT
0.3
0.2
0.1
0.0
-0.1
0.025
0.050
0.075
0.100
SNP effect on hexadecanedioate

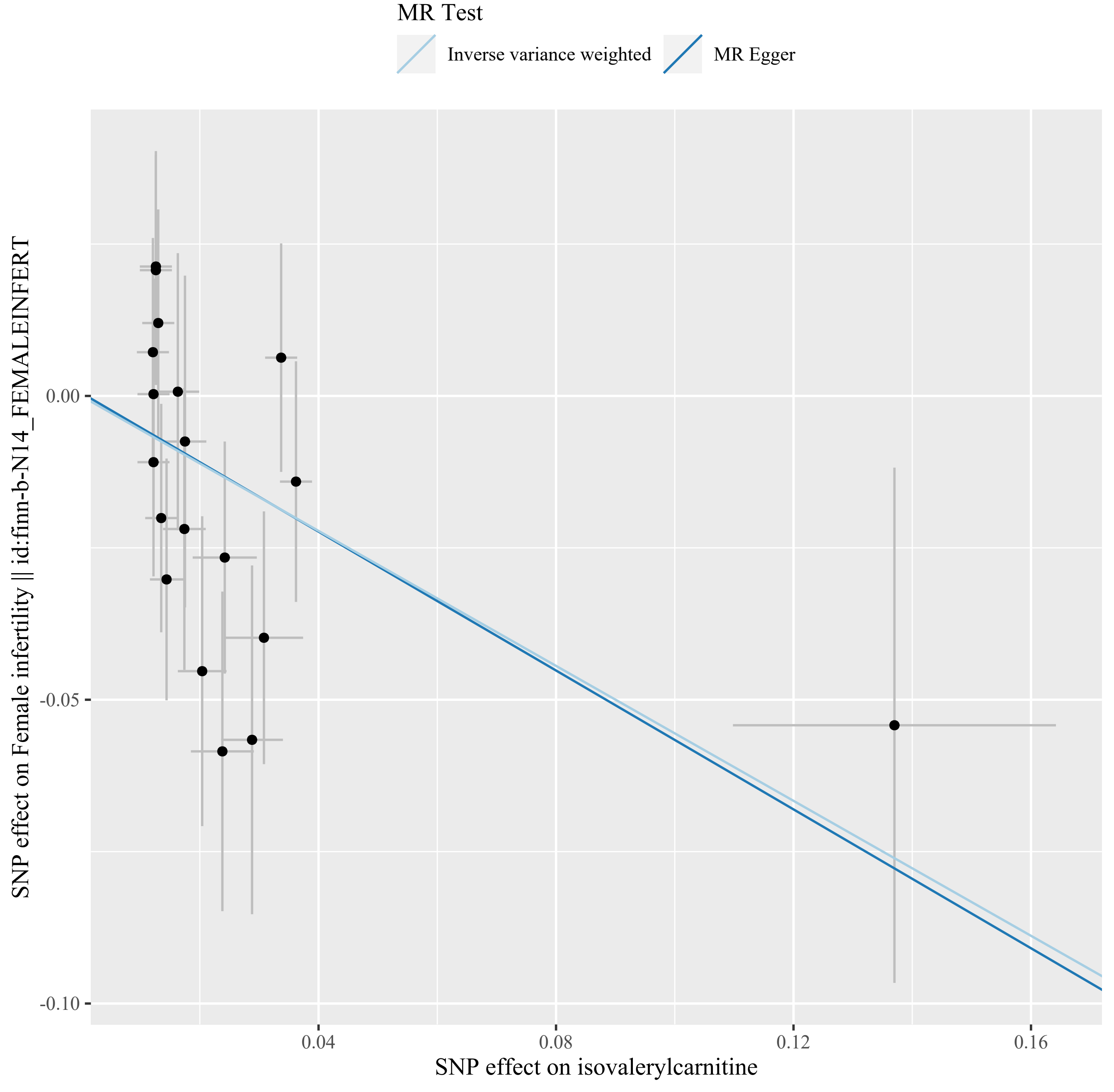
MR Test
Inverse variance weighted
MR Egger
SNP effect on Female infertility || id:finn-b-N14_FEMALEINFERT
0.00
-0.05
-0.10
0.04
0.08
0.12
0.16
SNP effect on isovalerylcarnitine

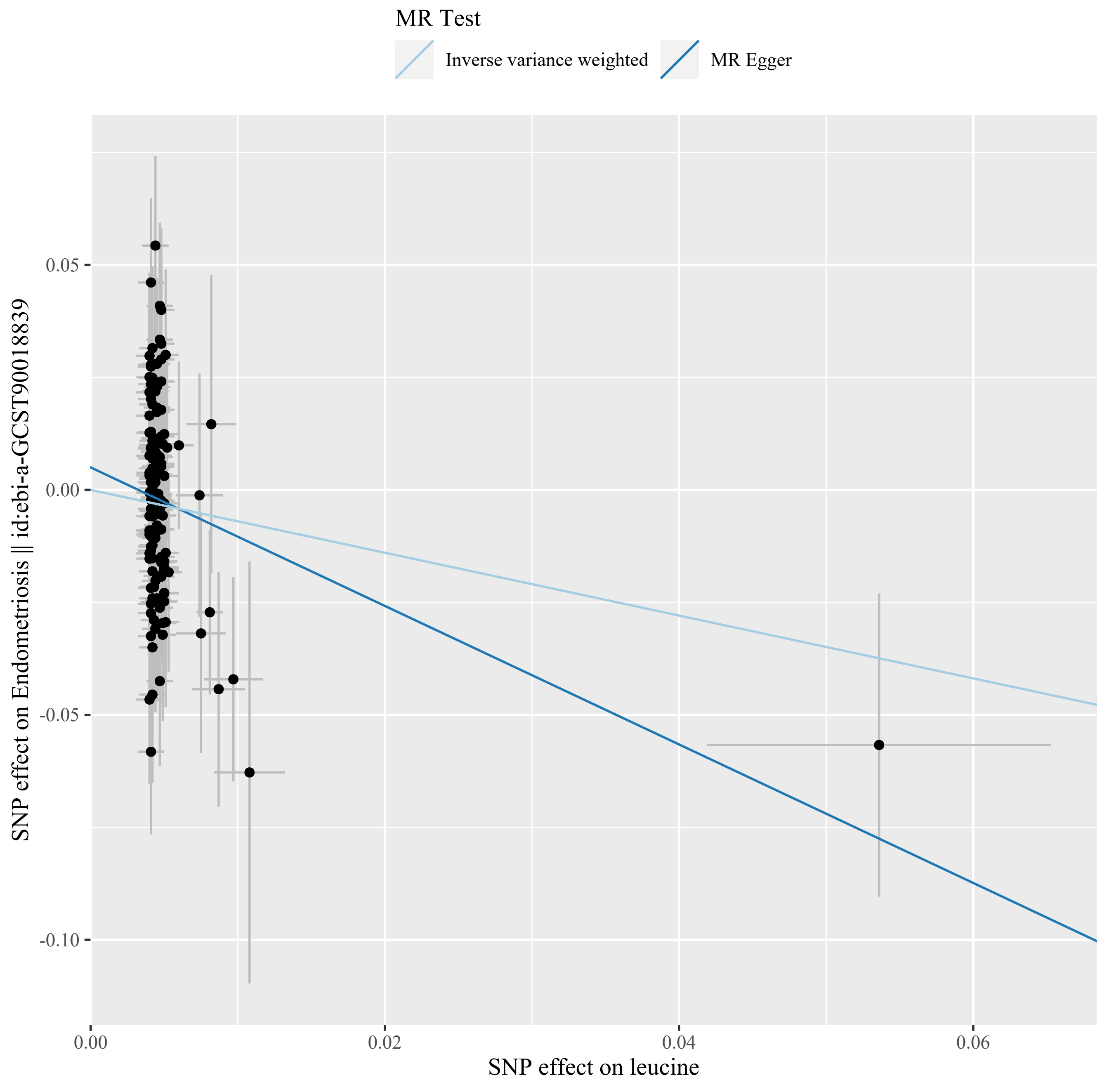

MR Test
Inverse variance weighted
MR Egger
0.05
0.00
-0.05
-0.10
0.00
0.02
0.04
0.06
SNP effect on Endometriosis || id:ebi-a-GCST90018839
SNP effect on leucine

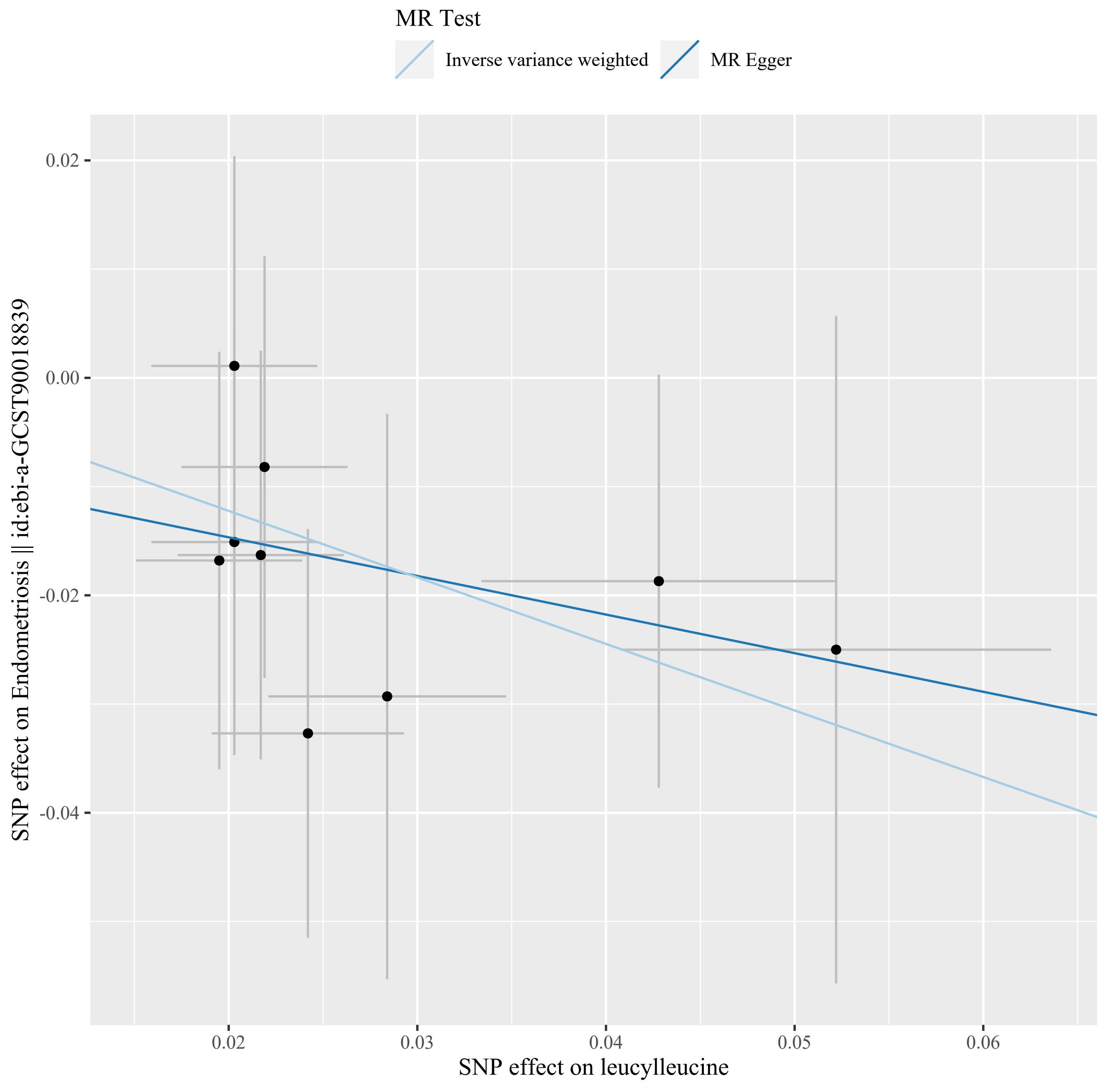
MR Test
Inverse variance weighted
MR Egger
0.02
0.00
-0.02
-0.04
0.02
0.03
0.04
0.05
0.06
SNP effect on leucylleucine
SNP effect on Endometriosis || id:ebi-a-GCST90018839

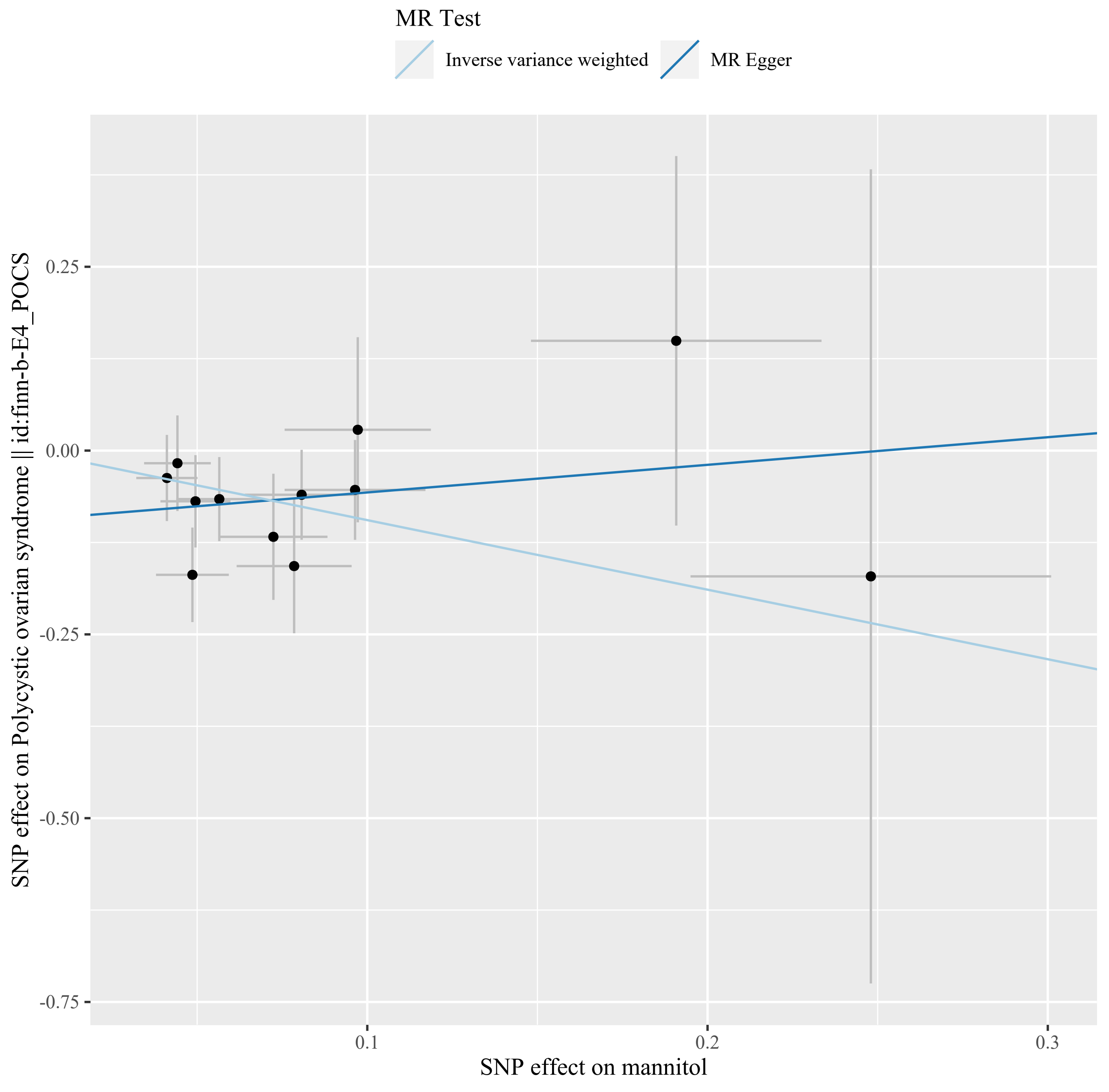

MR Test
Inverse variance weighted
MR Egger
SNP effect on Polycystic ovarian syndrome || id:finn-b-E4_POCS
0.25
0.00
-0.25
-0.50
-0.75
0.1
0.2
0.3
SNP effect on mannitol

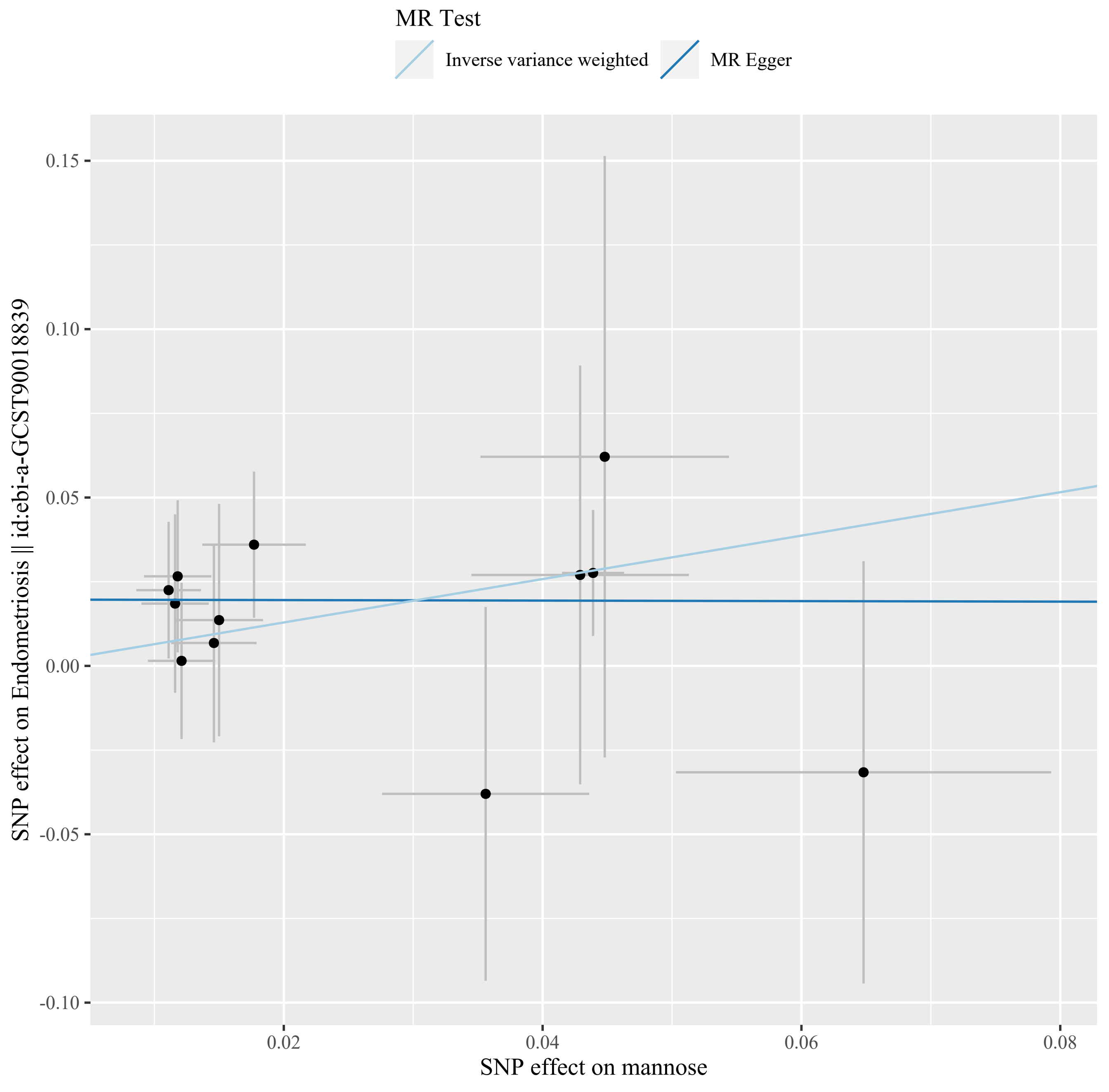
MR Test
Inverse variance weighted
MR Egger
SNP effect on Endometriosis || id:ebi-a-GCST90018839
SNP effect on mannose
0.15
0.10
0.05
0.00
-0.05
-0.10
0.02
0.04
0.06
0.08

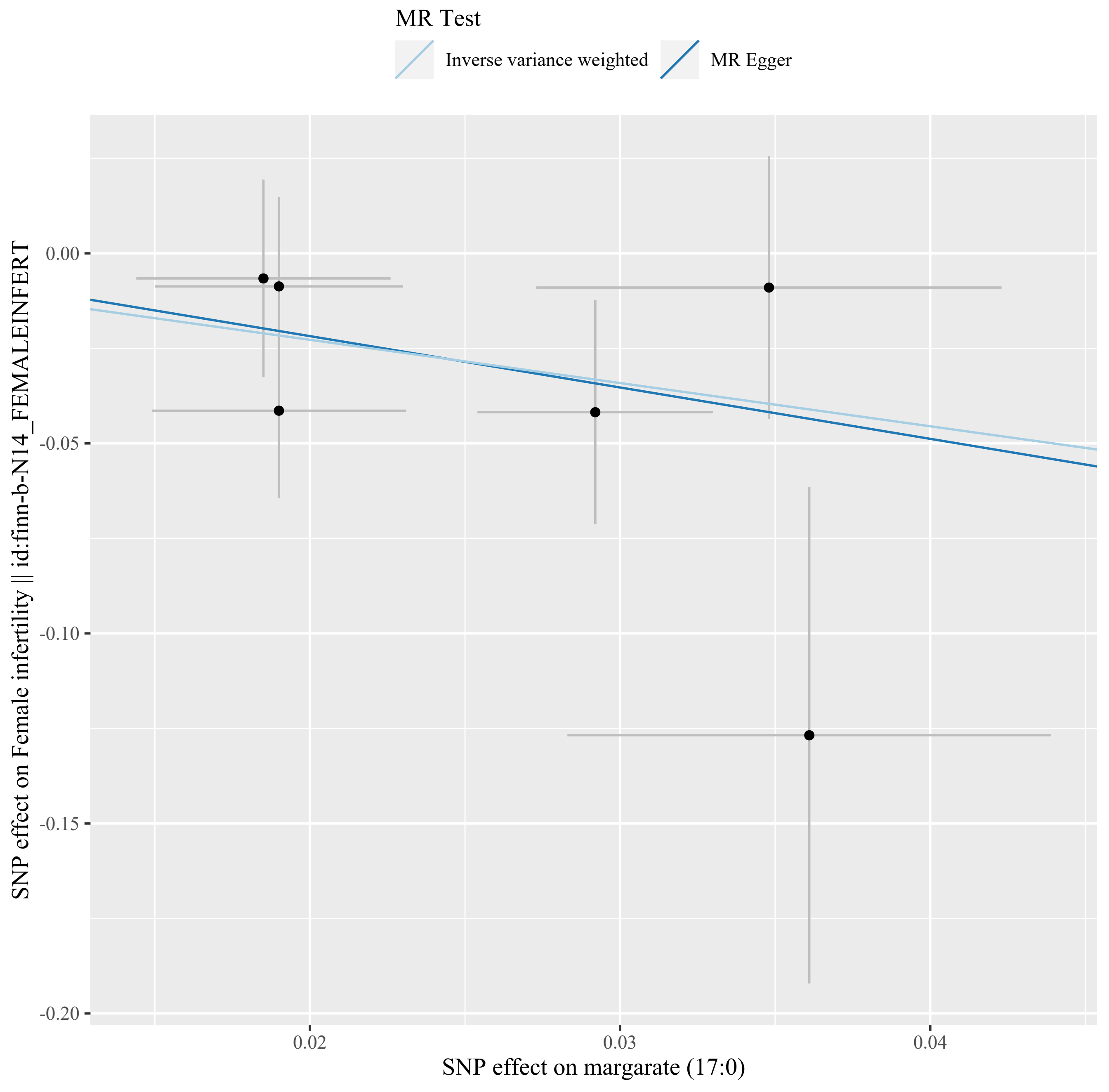
MR Test
Inverse variance weighted
MR Egger
0.00
-0.05
-0.10
-0.15
-0.20
0.02
0.03
0.04
SNP effect on margarate (17:0)
SNP effect on Female infertility || id:finn-b-N14_FEMALEINFERT

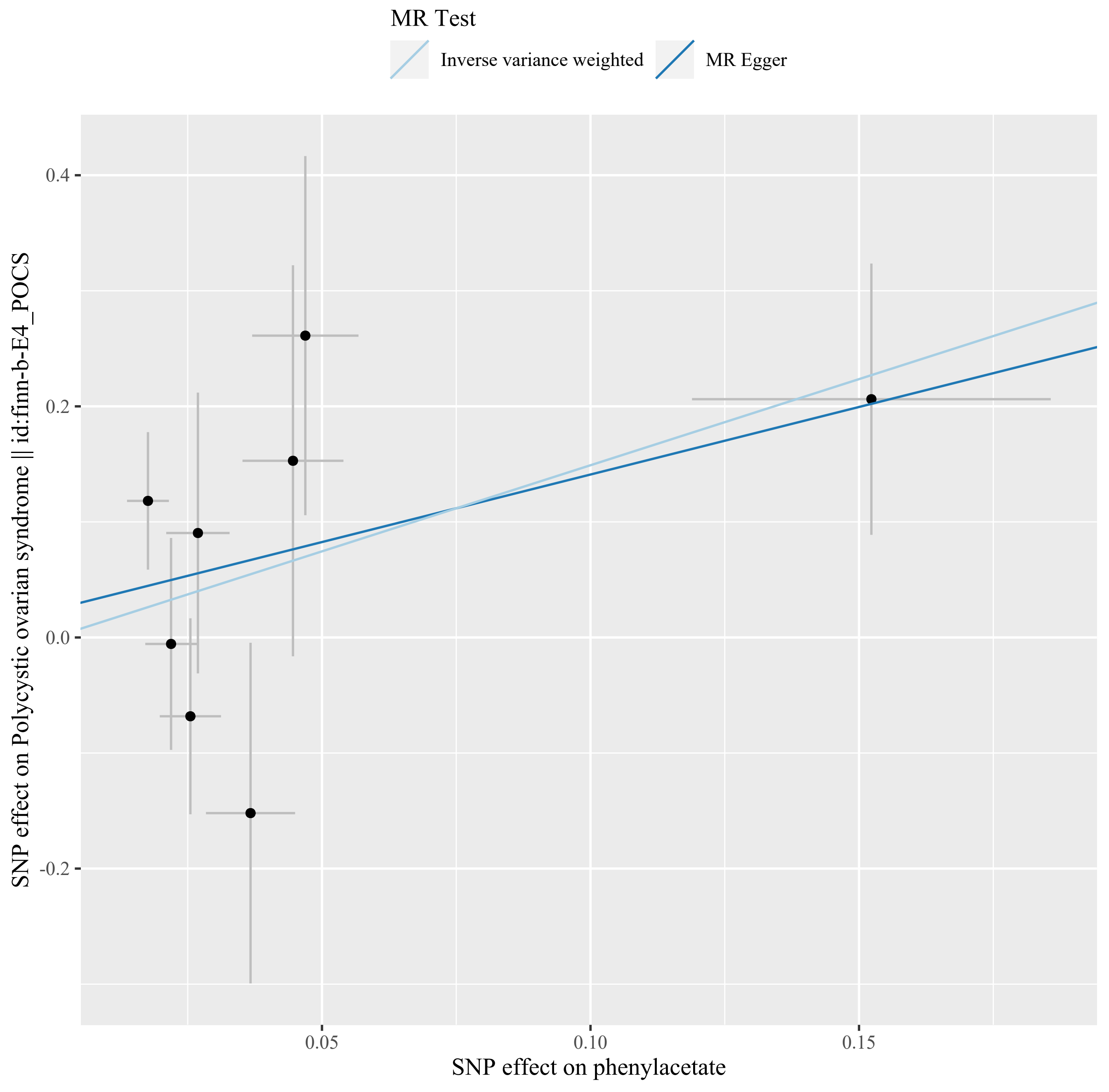
MR Test
Inverse variance weighted
MR Egger
0.4
0.2
0.0
-0.2
0.05
0.10
0.15
SNP effect on Polycystic ovarian syndrome || id:finn-b-E4_POCS
SNP effect on phenylacetate

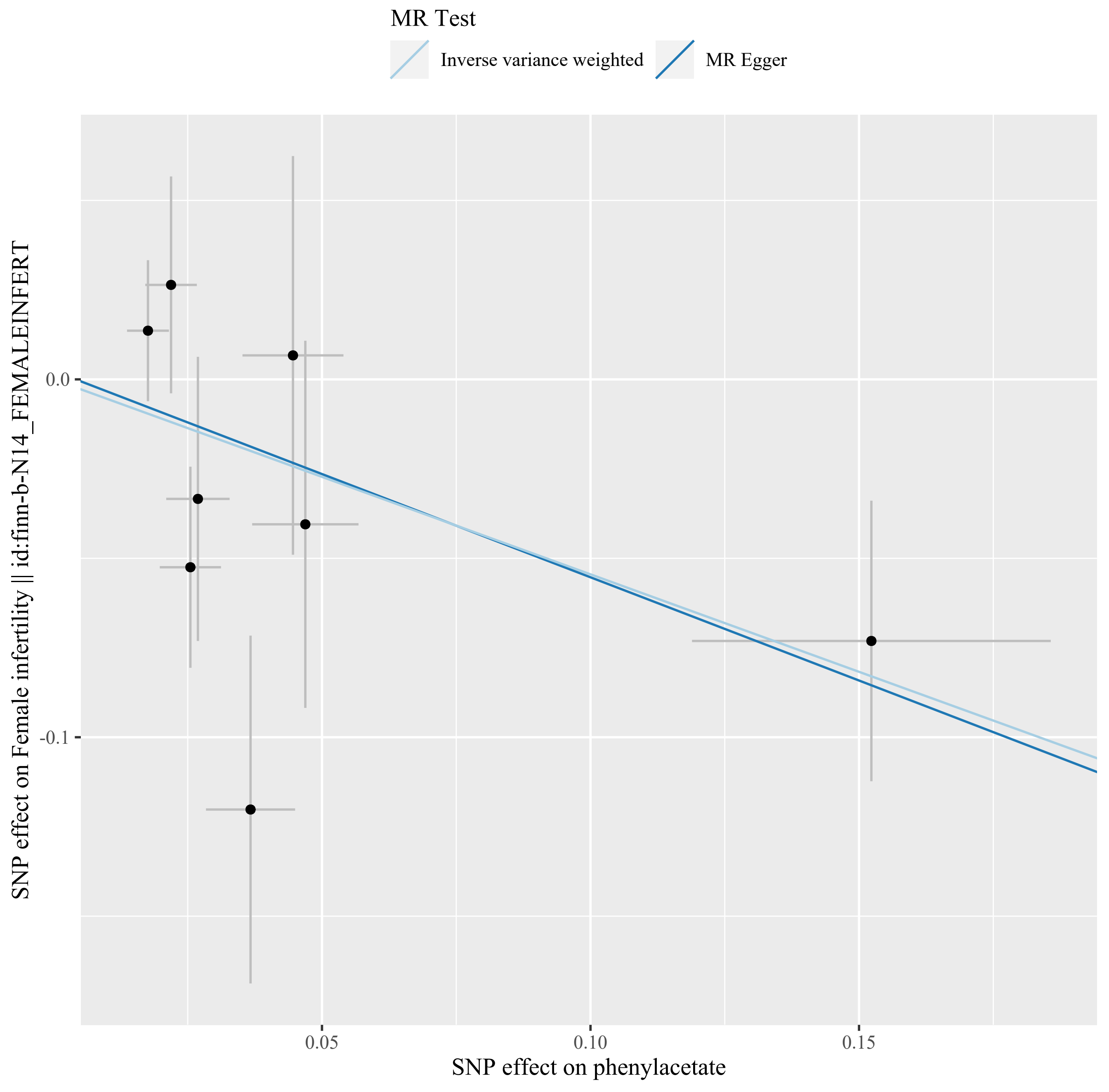
MR Test
Inverse variance weighted
MR Egger
SNP effect on Female infertility || id:finn-b-N14_FEMALEINFERT
0.0
-0.1
0.05
0.10
0.15
SNP effect on phenylacetate

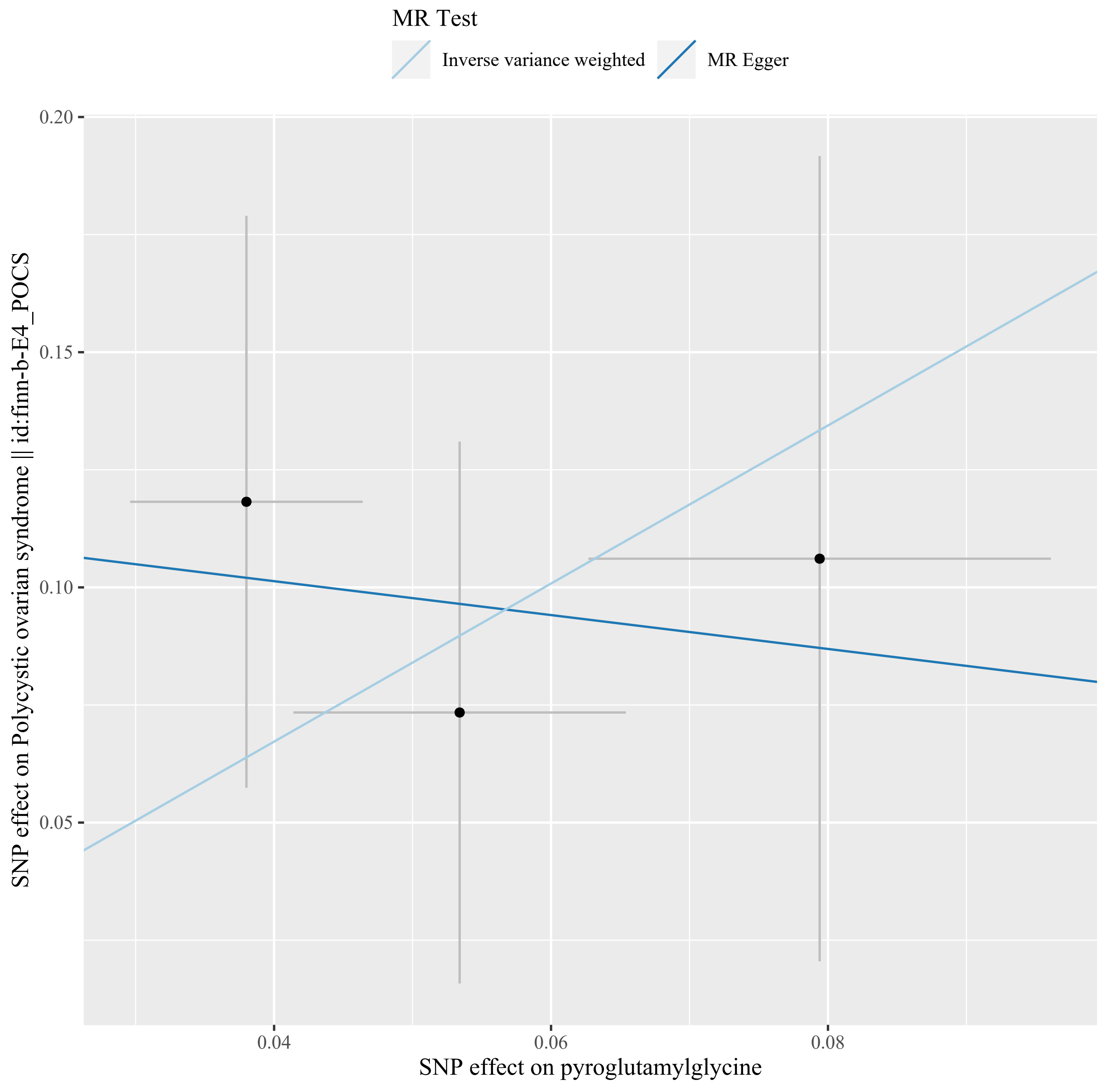

MR Test
Inverse variance weighted
MR Egger
0.20
0.15
0.10
0.05
0.04
0.06
0.08
SNP effect on Polycystic ovarian syndrome || id:finn-b-E4_POCS
SNP effect on pyroglutamylglycine

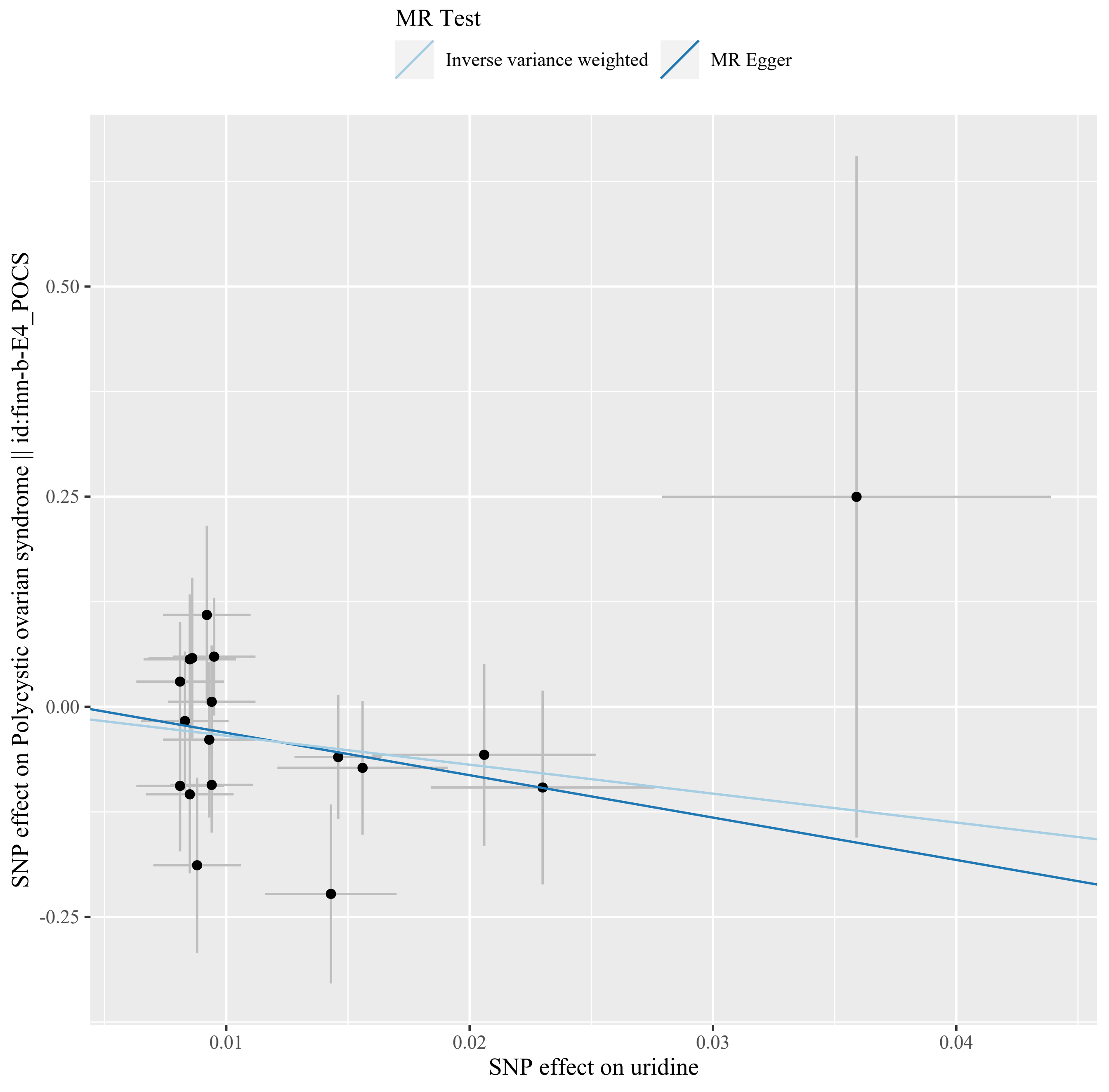
MR Test
Inverse variance weighted
MR Egger
SNP effect on Polycystic ovarian syndrome || id:finn-b-E4_POCS
0.50
0.25
0.00
-0.25
0.01
0.02
0.03
0.04
SNP effect on uridine

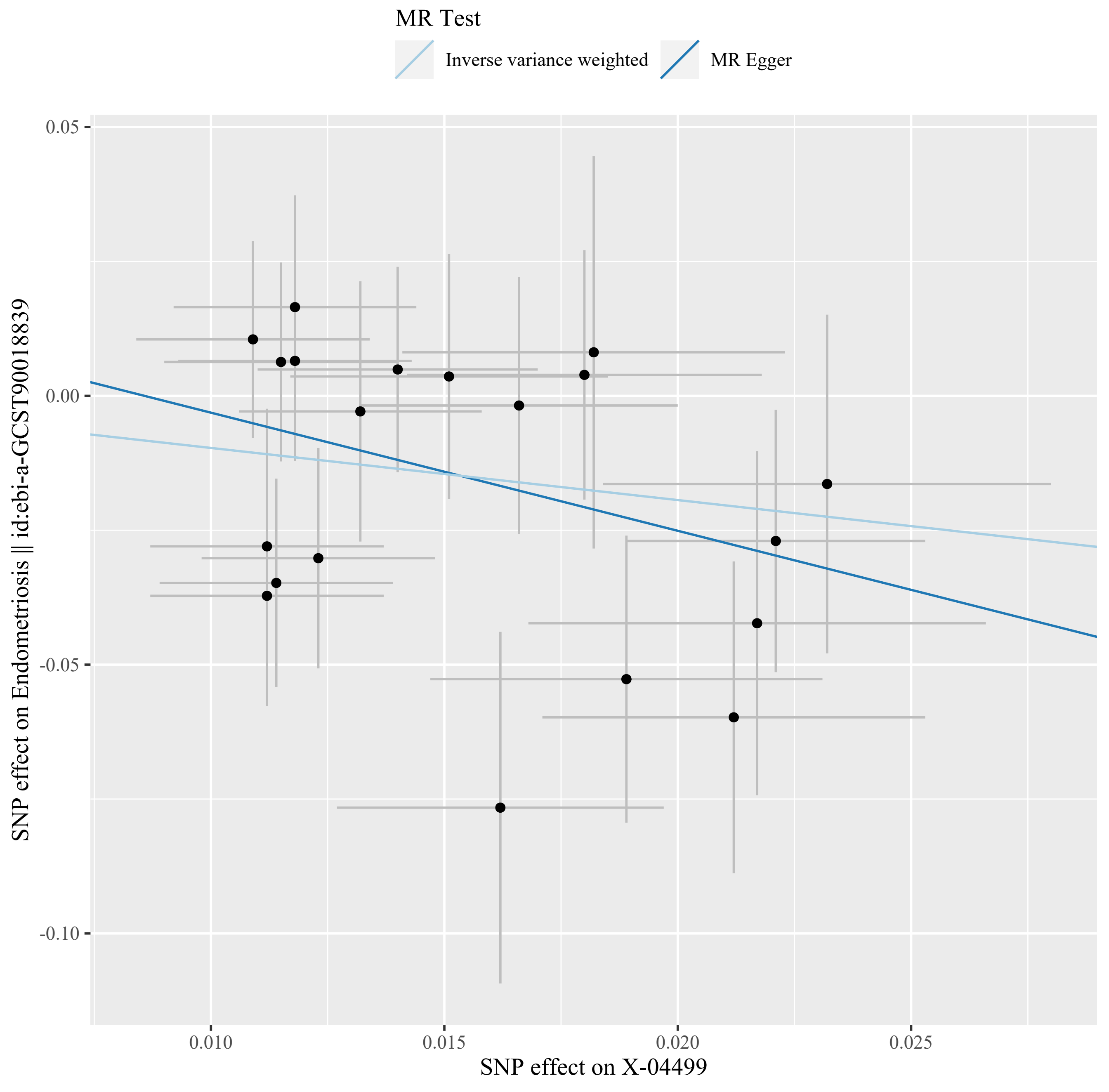
MR Test
Inverse variance weighted
MR Egger
0.05
0.00
-0.05
-0.10
0.010
0.015
0.020
0.025
SNP effect on X-04499
SNP effect on Endometriosis || id:ebi-a-GCST90018839

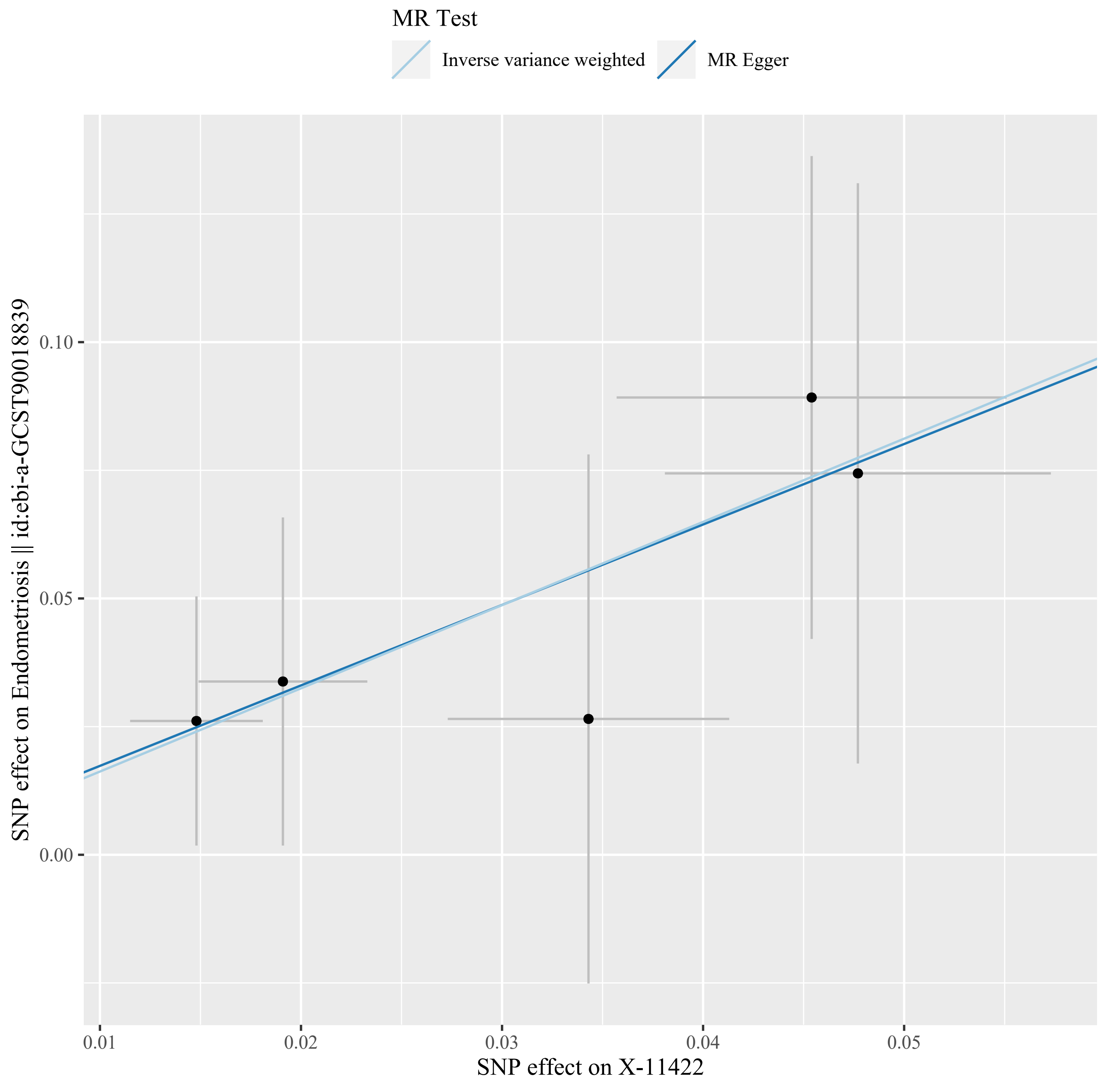
MR Test
Inverse variance weighted
MR Egger
SNP effect on Endometriosis || id:ebi-a-GCST90018839
0.10
0.05
0.00
0.01
0.02
0.03
0.04
0.05
SNP effect on X-11422

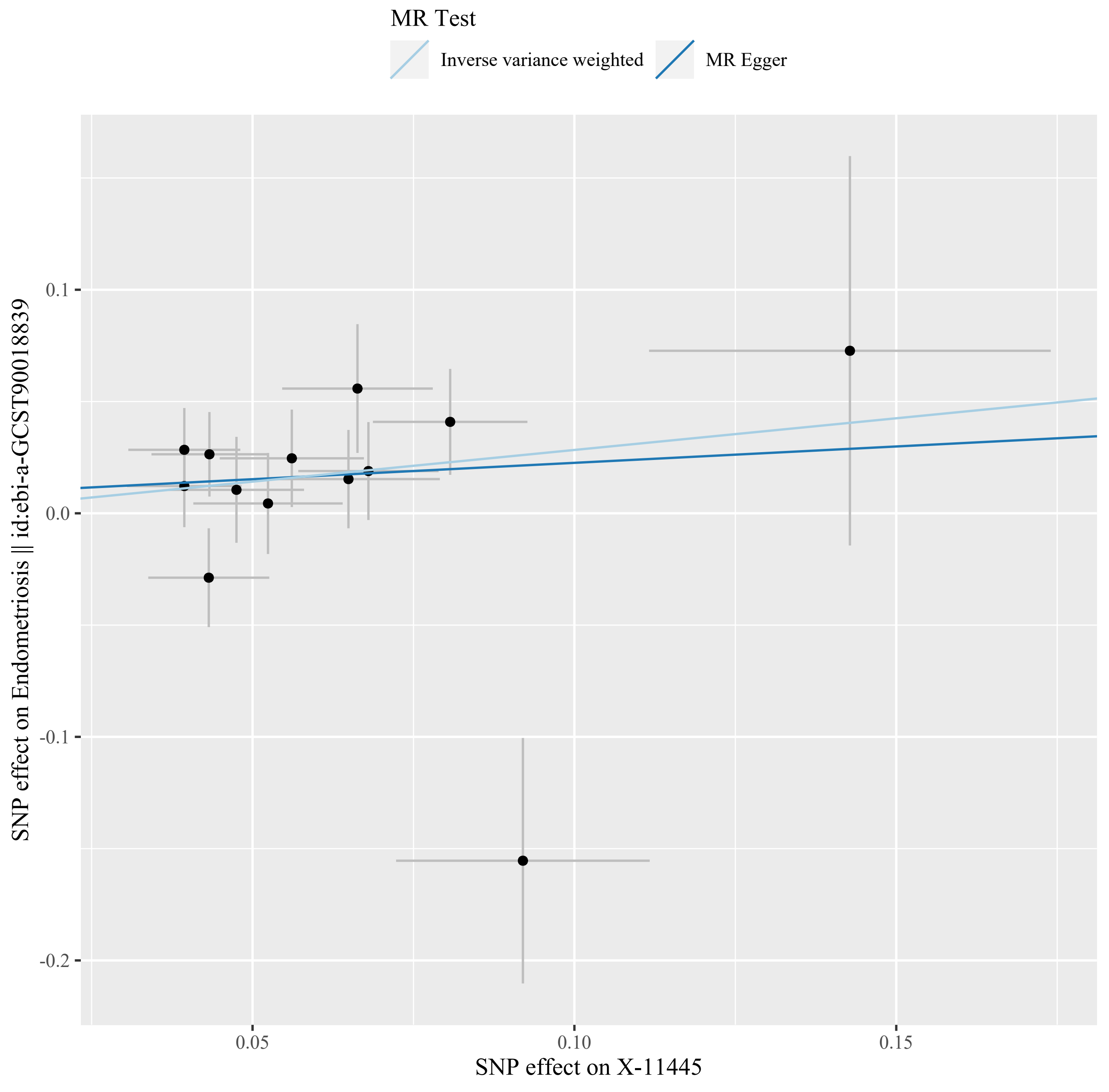
MR Test
Inverse variance weighted
MR Egger
0.1
0.0
-0.1
-0.2
0.05
0.10
0.15
SNP effect on X-11445
SNP effect on Endometriosis || id:ebi-a-GCST90018839

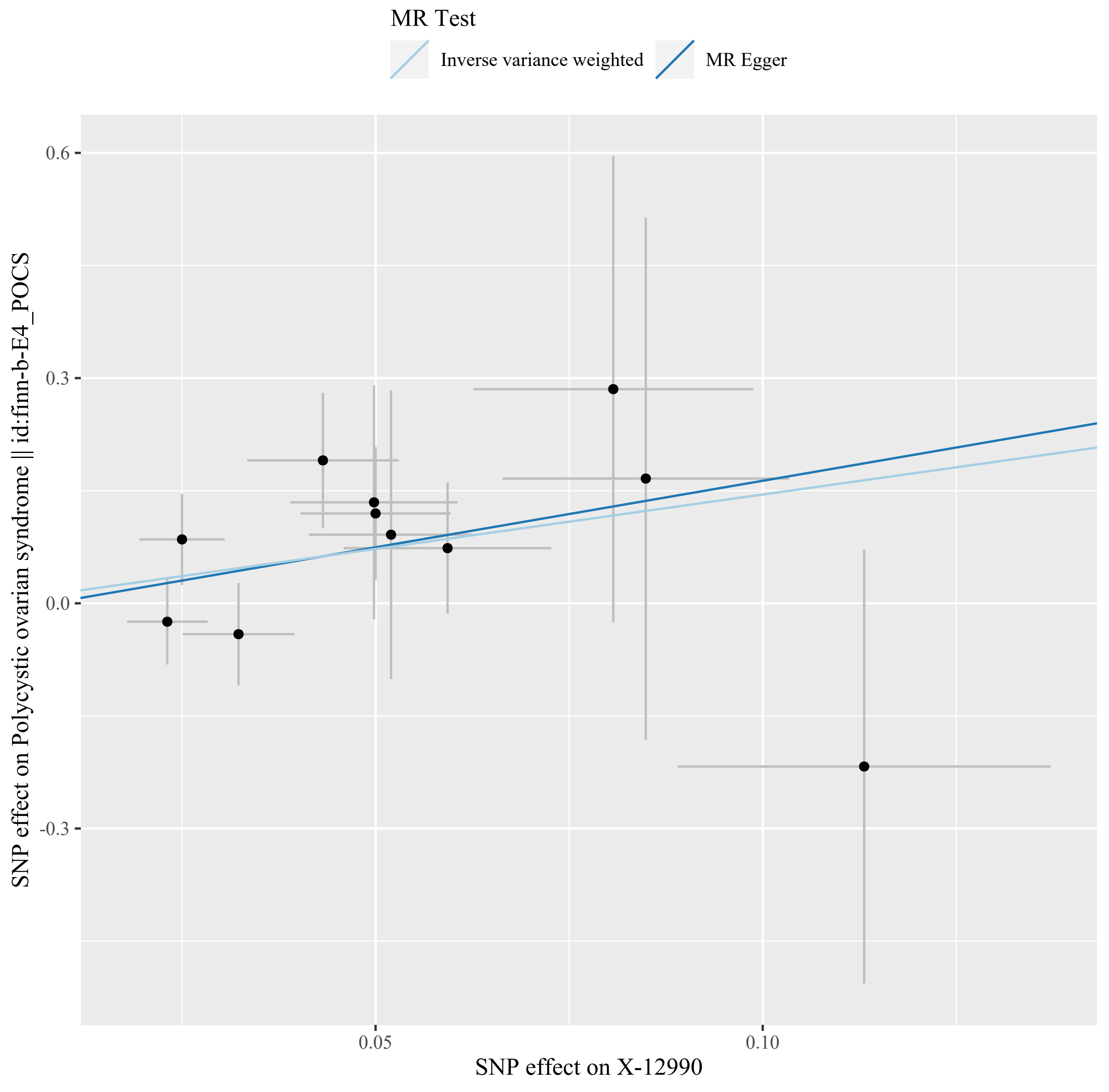
MR Test
Inverse variance weighted
MR Egger
0.6
0.3
0.0
-0.3
0.05
0.10
SNP effect on Polycystic ovarian syndrome || id:finn-b-E4_POCS
SNP effect on X-12990
