# Supplementary material for: Investigating the metabolomic pathways in female reproductive endocrine disorders: a Mendelian randomization study
Source: Front Endocrinol (Lausanne). 2024 Oct 31;15:1438079. doi: 10.3389/fendo.2024.1438079 (PMC11560792; doi:10.3389/fendo.2024.1438079)
Supplement: Supplementary Figure 2 — Funnel plot. The funnel plot visualizes the distribution of effect estimates from instrumental variables against their precision, assessing potential publication bias and asymmetry in the MR analysis for metabolic markers related to reproductive disorders. [file Image2.pdf]

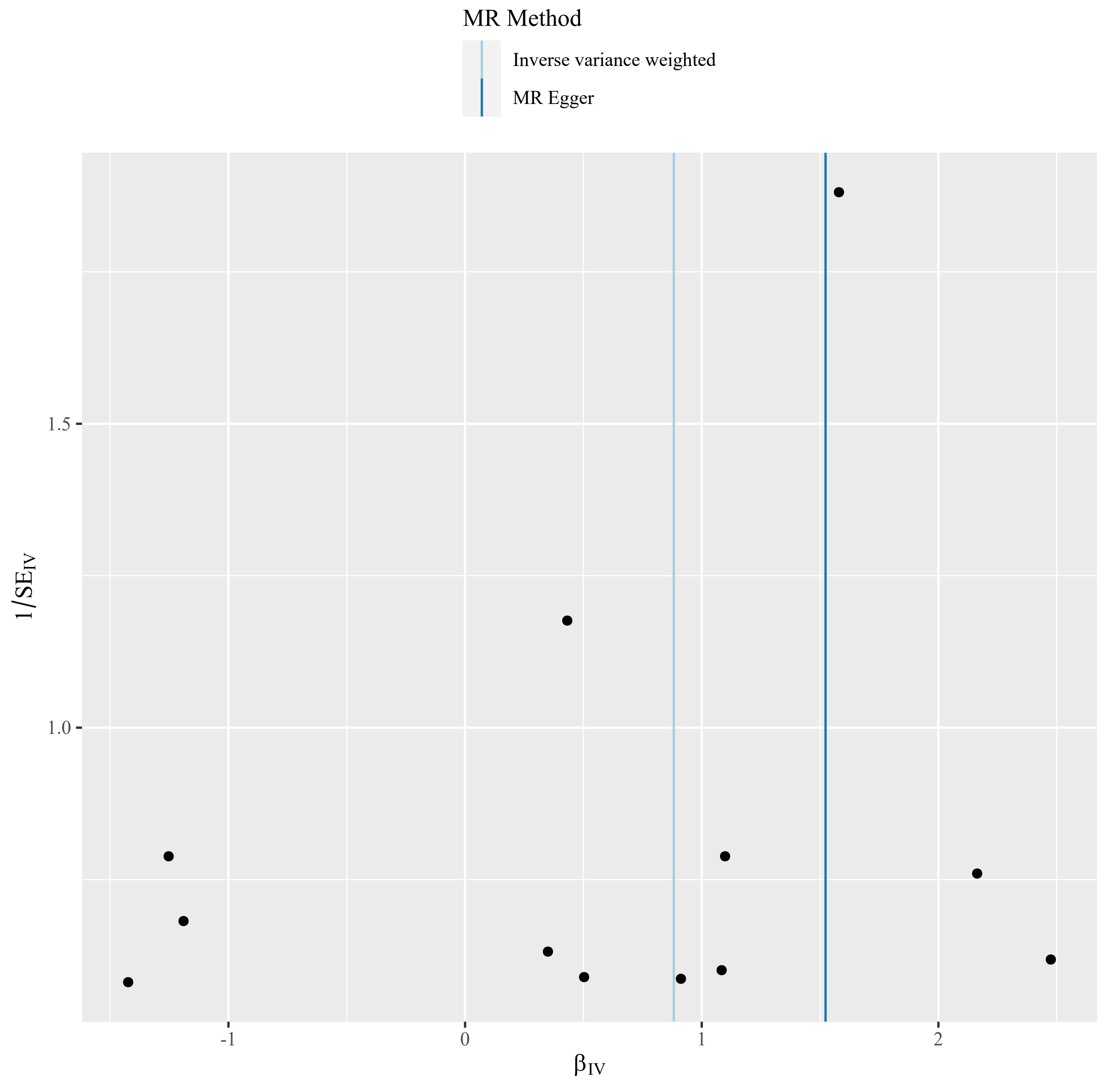

MR Method
Inverse variance weighted
MR Egger
1.5
1.0
1/SE$_{IV}$
-1
0
1
2
β$_{IV}$

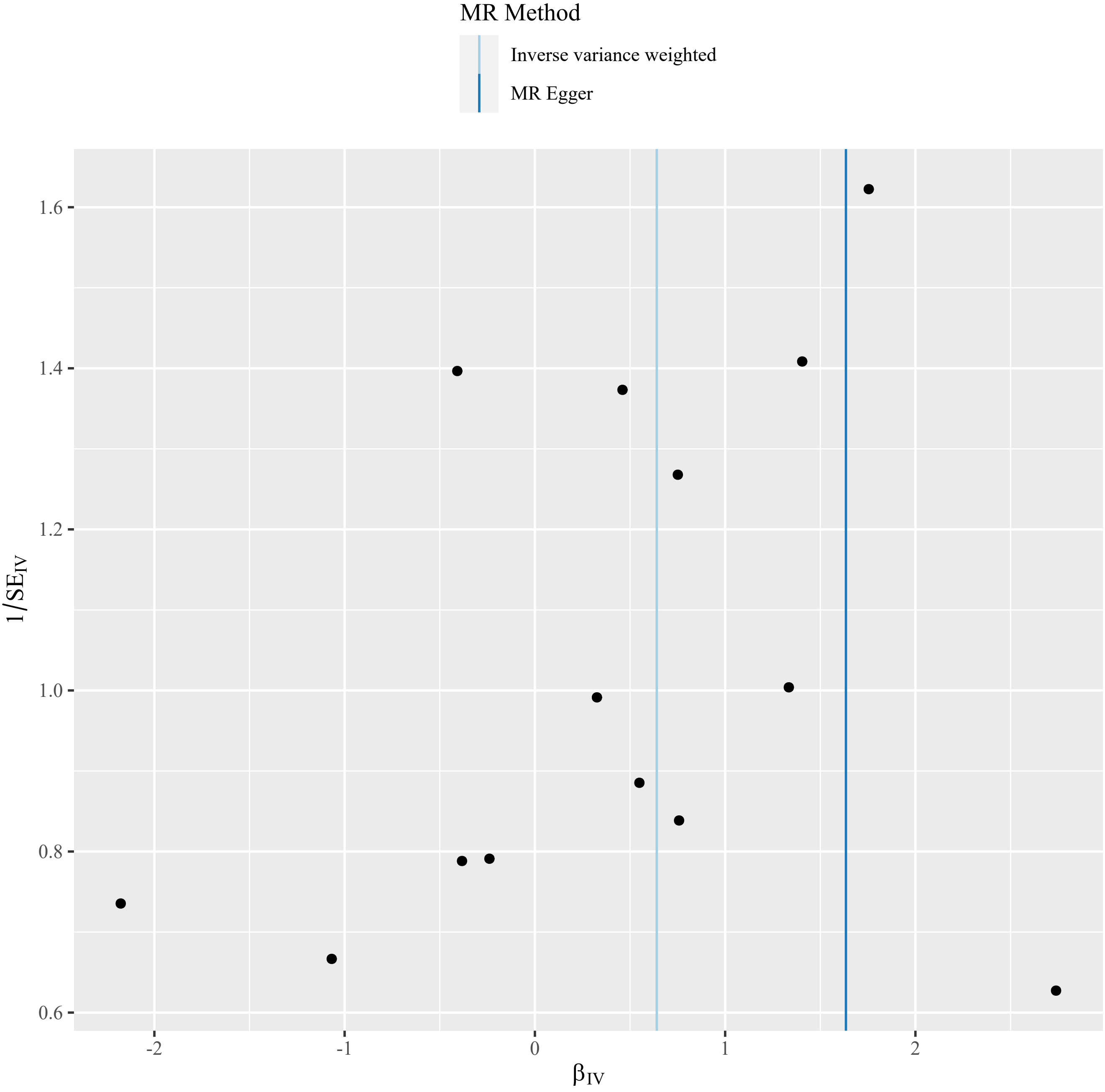

MR Method
Inverse variance weighted
MR Egger
1.6
1.4
1.2
1.0
0.8
0.6
$1/SE_{IV}$
-2
-1
0
1
2
β_{IV}

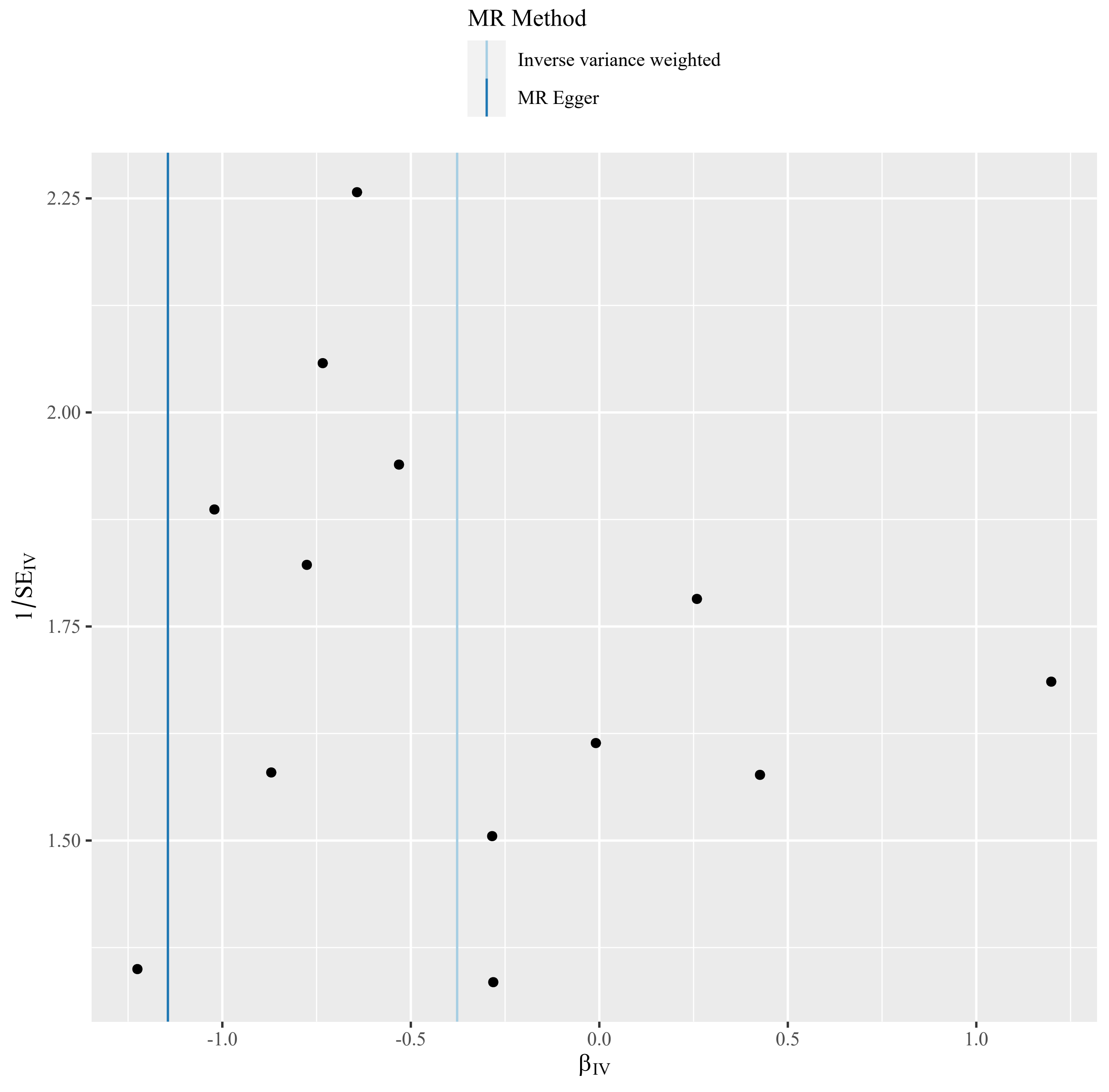

MR Method
Inverse variance weighted
MR Egger
2.25
2.00
1.75
1.50
1/SE_IV
-1.0
-0.5
0.0
0.5
1.0
β_IV

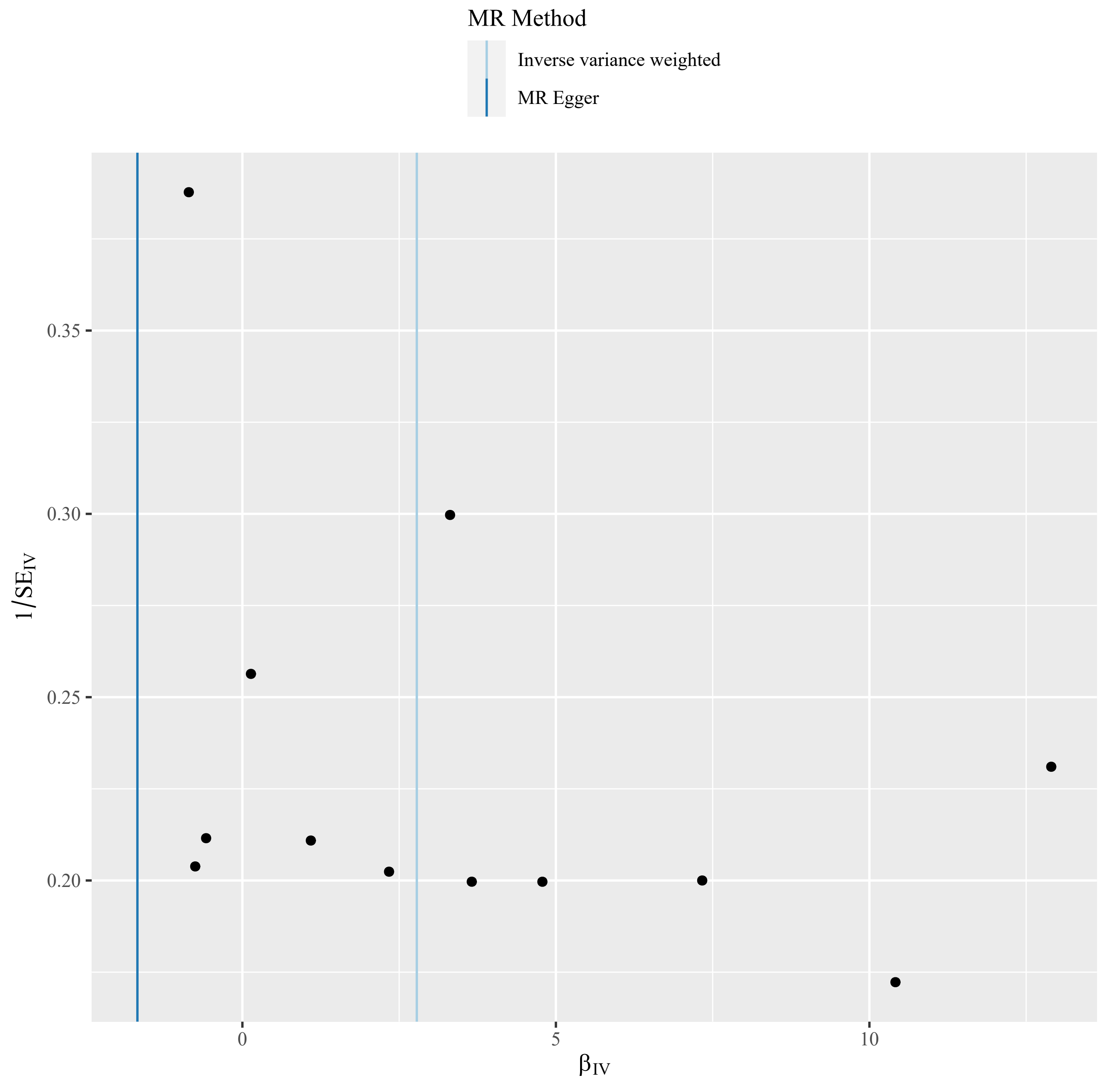

MR Method
Inverse variance weighted
MR Egger
0.35
0.30
0.25
0.20
1/SE_IV
0
5
10
β_IV

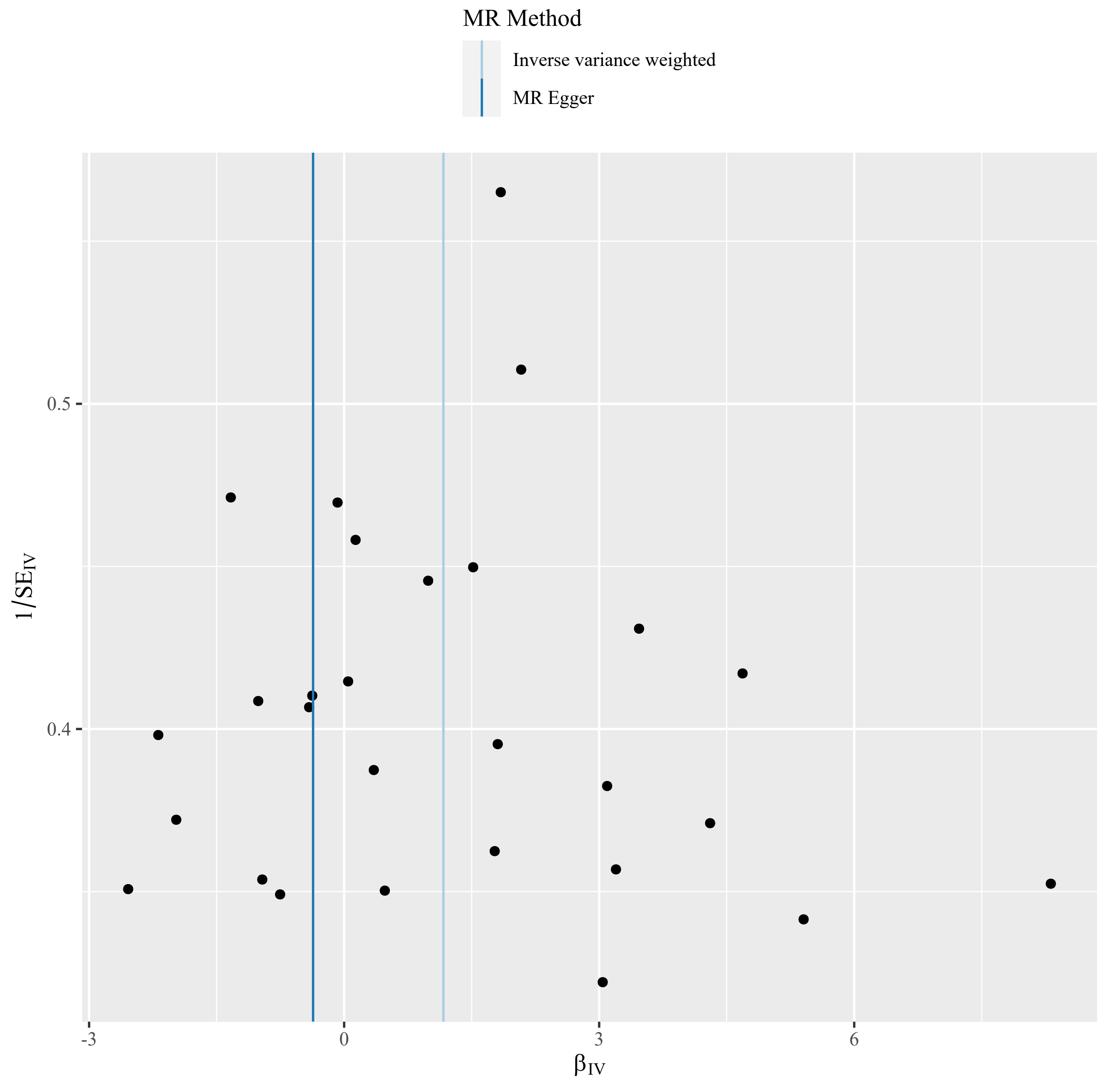

MR Method
Inverse variance weighted
MR Egger
0.5
0.4
$1/SE_{IV}$
-3
0
3
6
β_{IV}

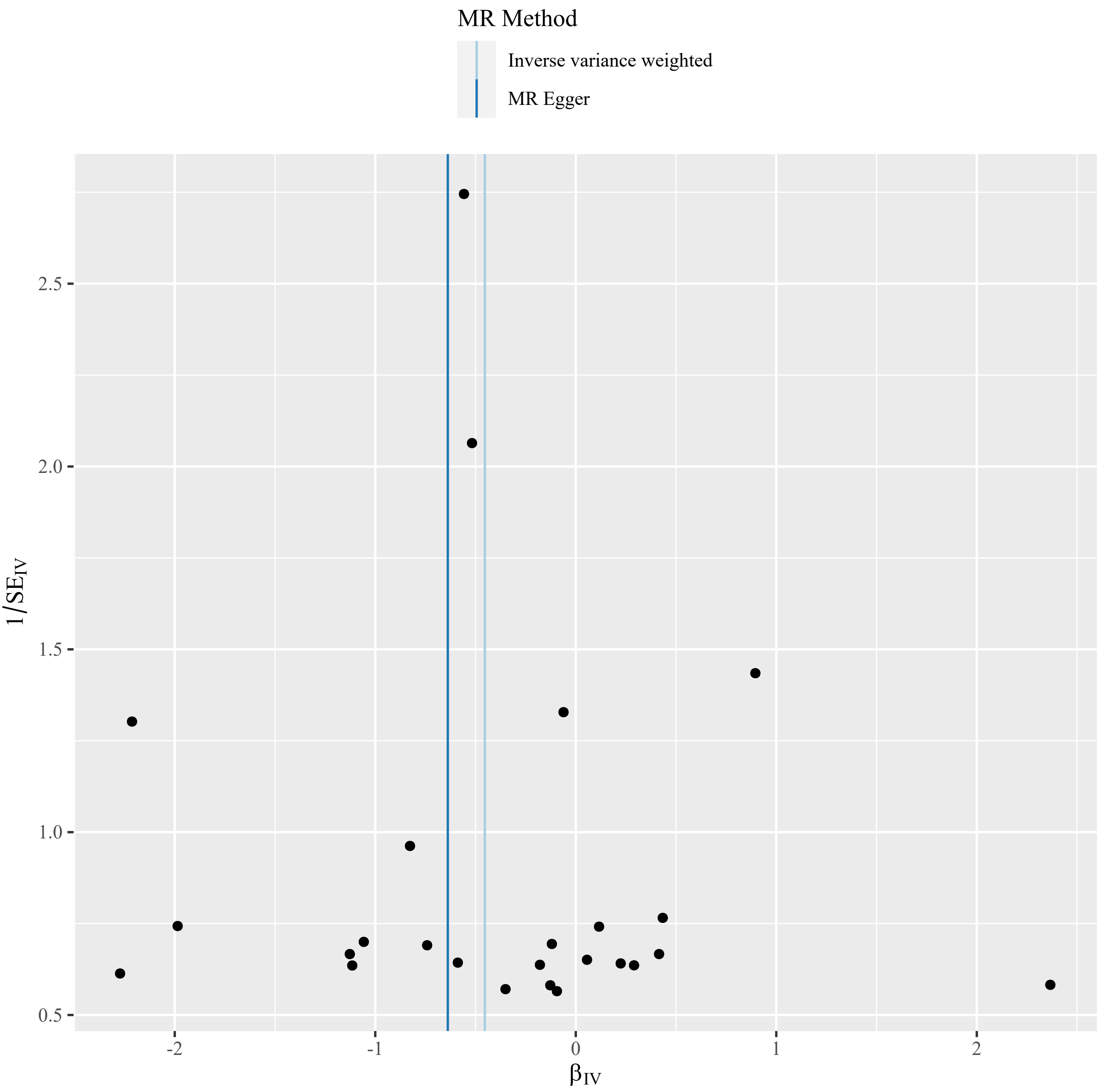

MR Method
Inverse variance weighted
MR Egger
2.5
2.0
1.5
1.0
0.5
1/SE_IV
-2
-1
0
1
2
β_IV

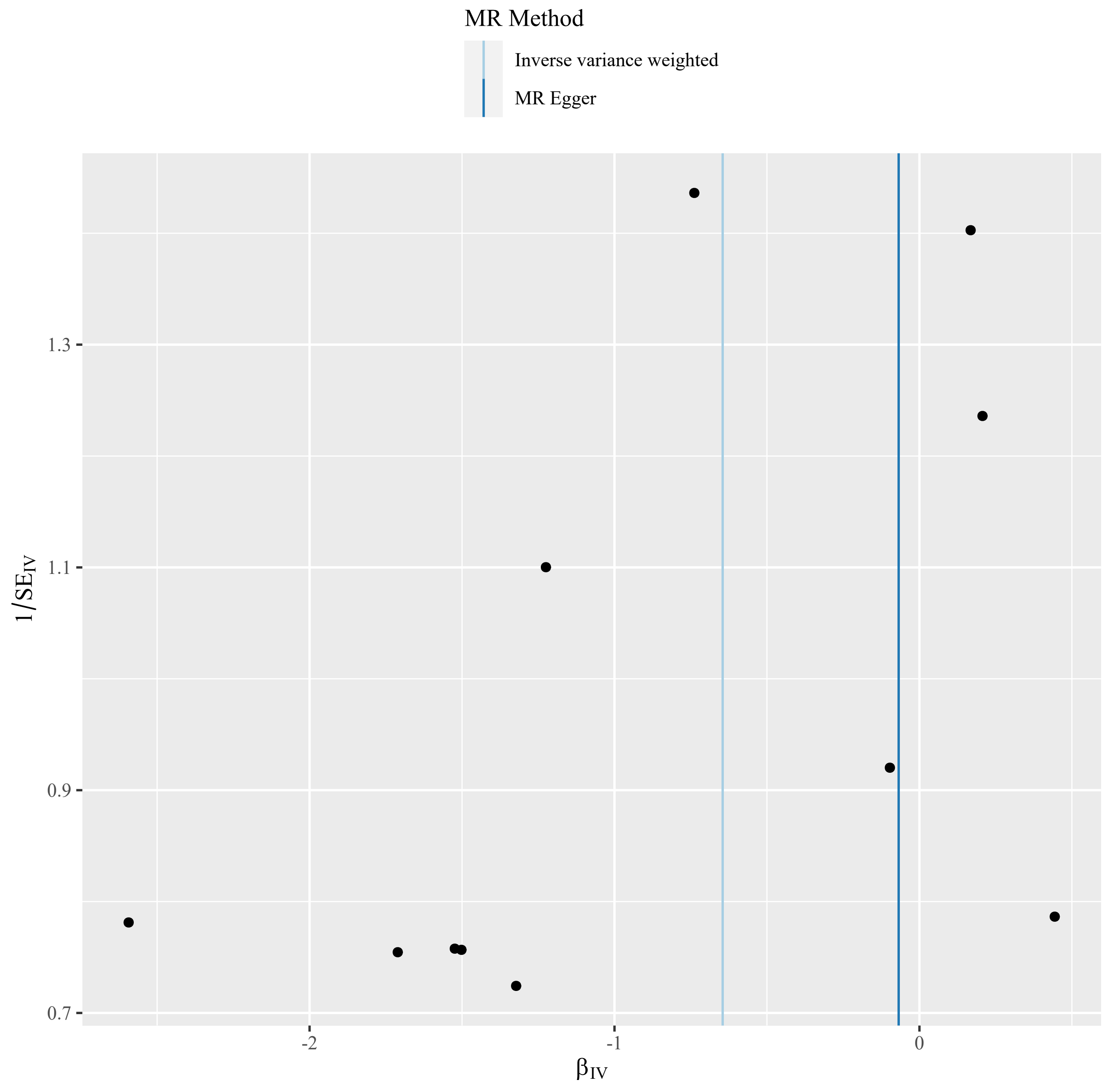
MR Method
Inverse variance weighted
MR Egger
1.3
1.1
0.9
0.7
$1/SE_{IV}$
-2
-1
0
β_{IV}

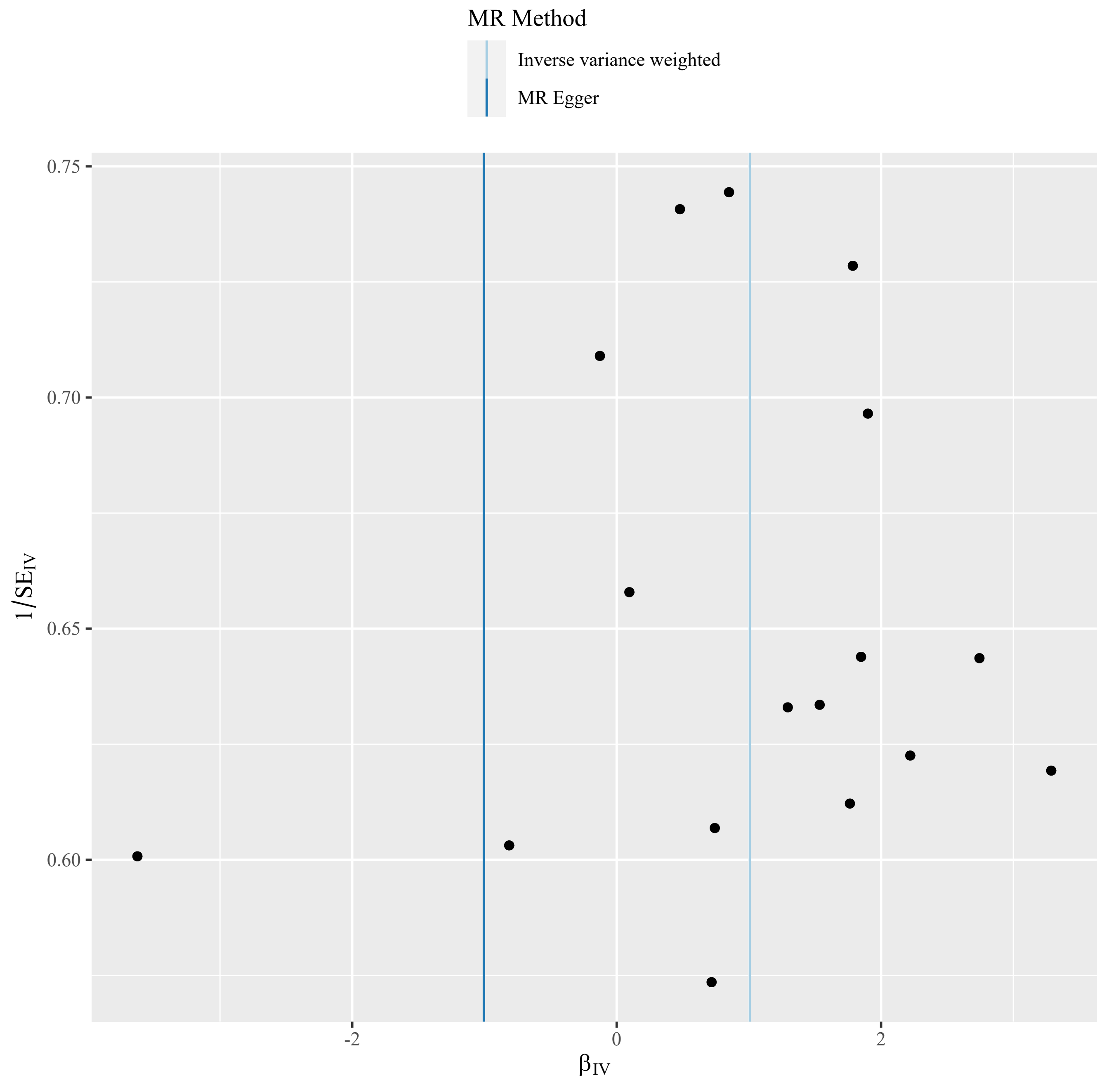
MR Method
Inverse variance weighted
MR Egger
0.75
0.70
0.65
0.60
$1/SE_{IV}$
-2
0
2
β_{IV}

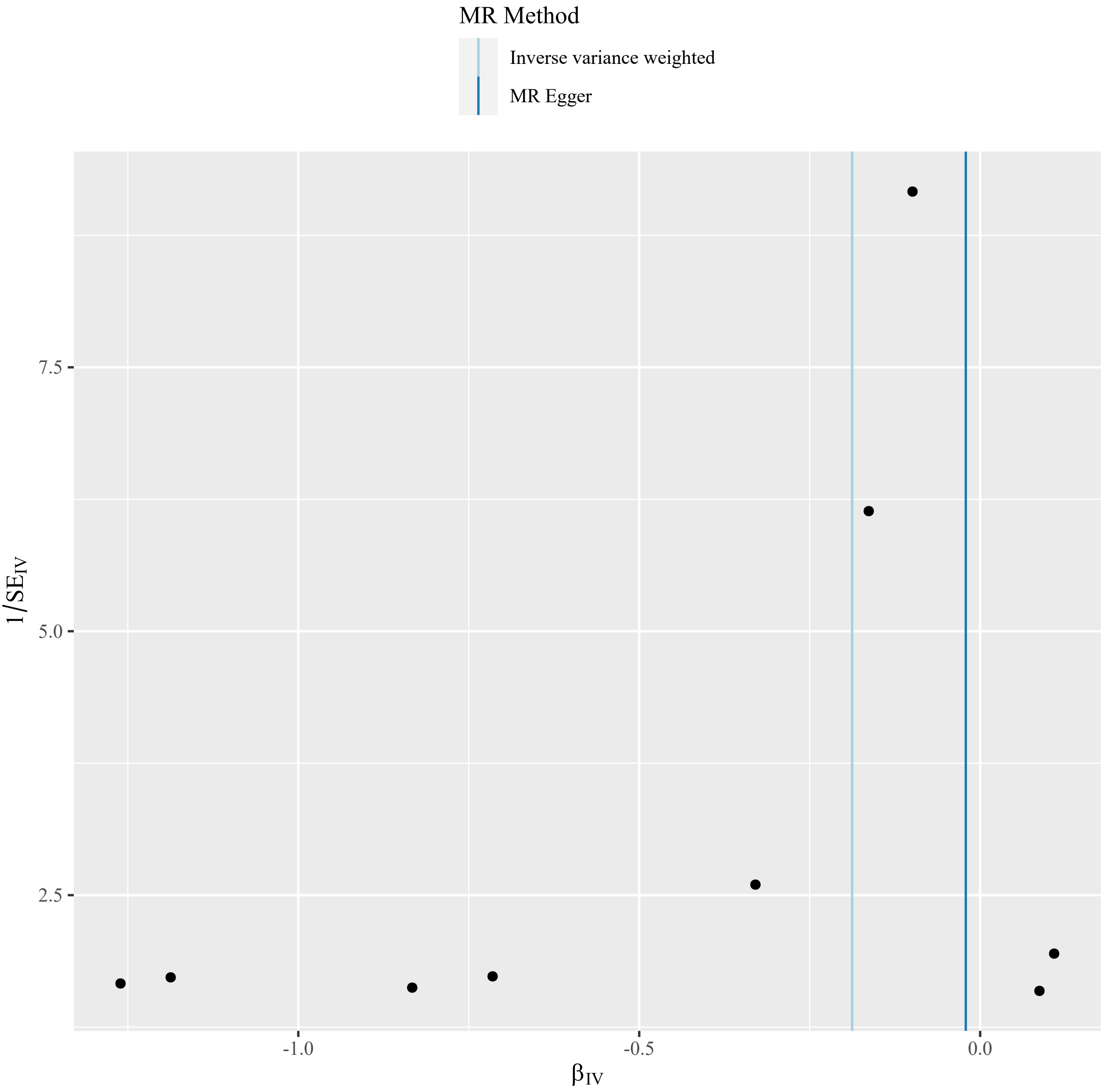
MR Method
Inverse variance weighted
MR Egger
7.5
5.0
2.5
-1.0
-0.5
0.0
$1/SE_{IV}$
β_{IV}

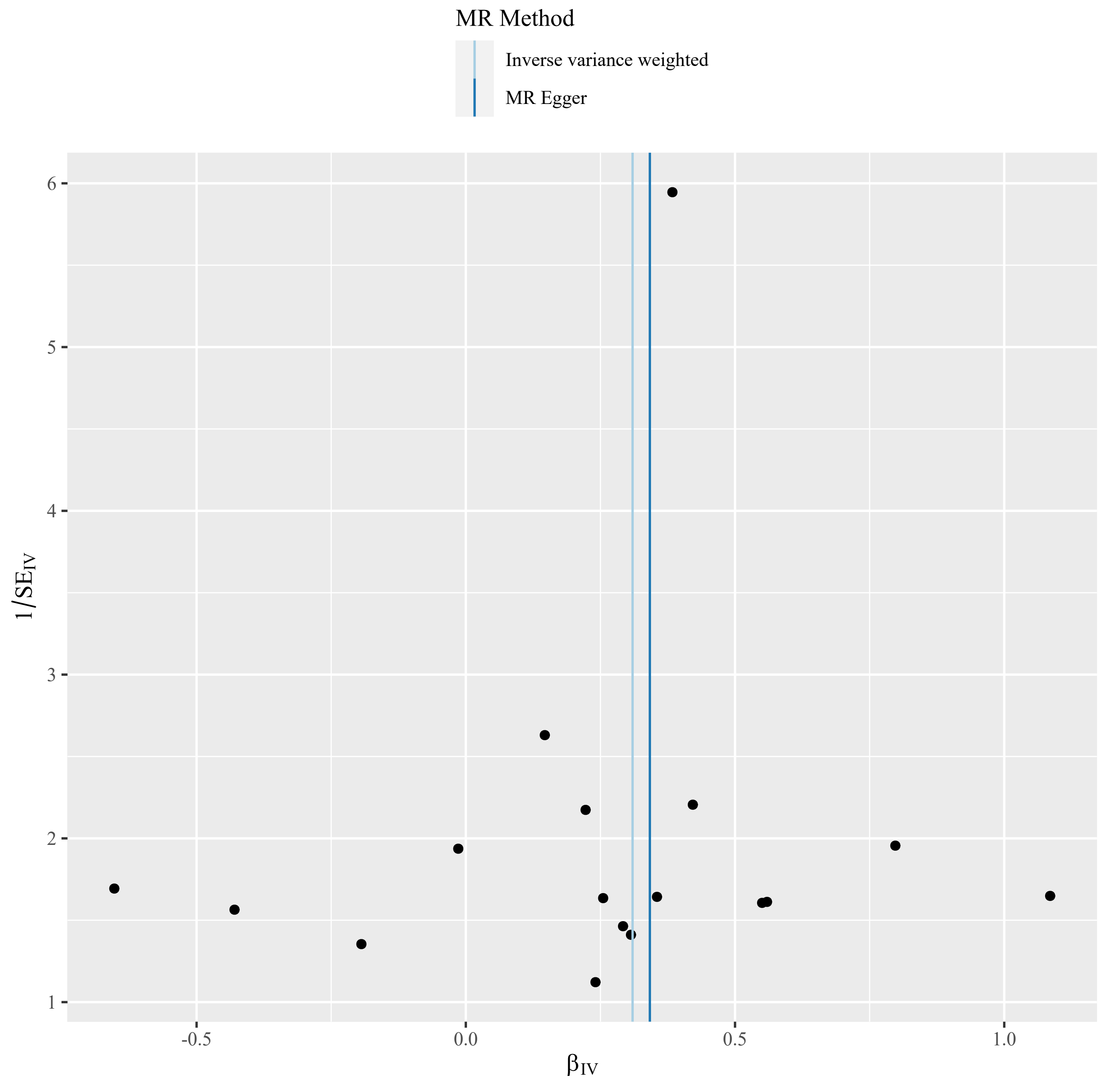

MR Method
Inverse variance weighted
MR Egger
6
5
4
3
2
1
1/SE_IV
-0.5
0.0
0.5
1.0
β_IV

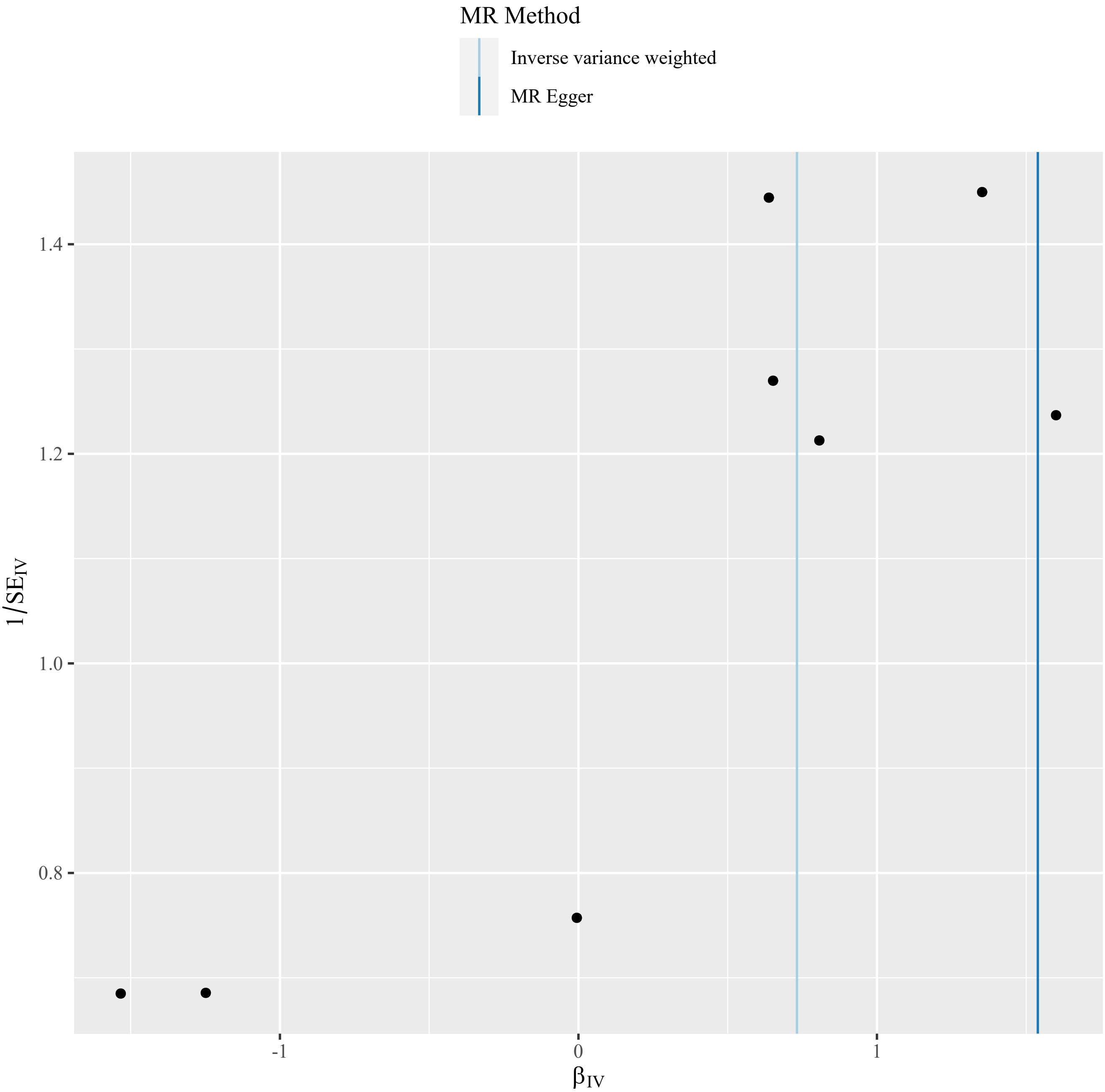

MR Method
Inverse variance weighted
MR Egger
1.4
1.2
1.0
0.8
-1
0
1
1/SE_IV
β_IV

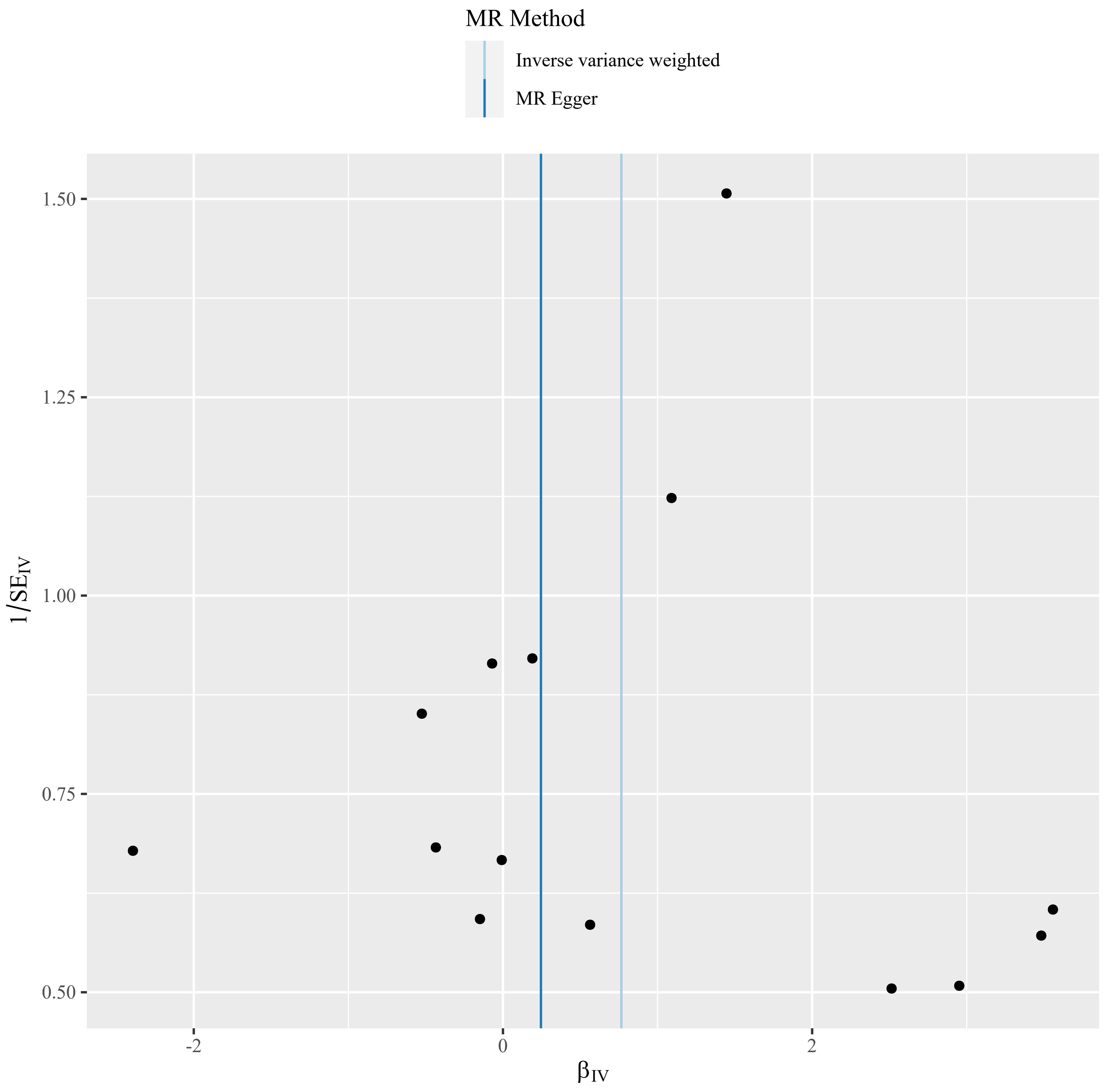
MR Method
Inverse variance weighted
MR Egger
1.50
1.25
1.00
0.75
0.50
$1/SE_{IV}$
-2
0
2
β_{IV}

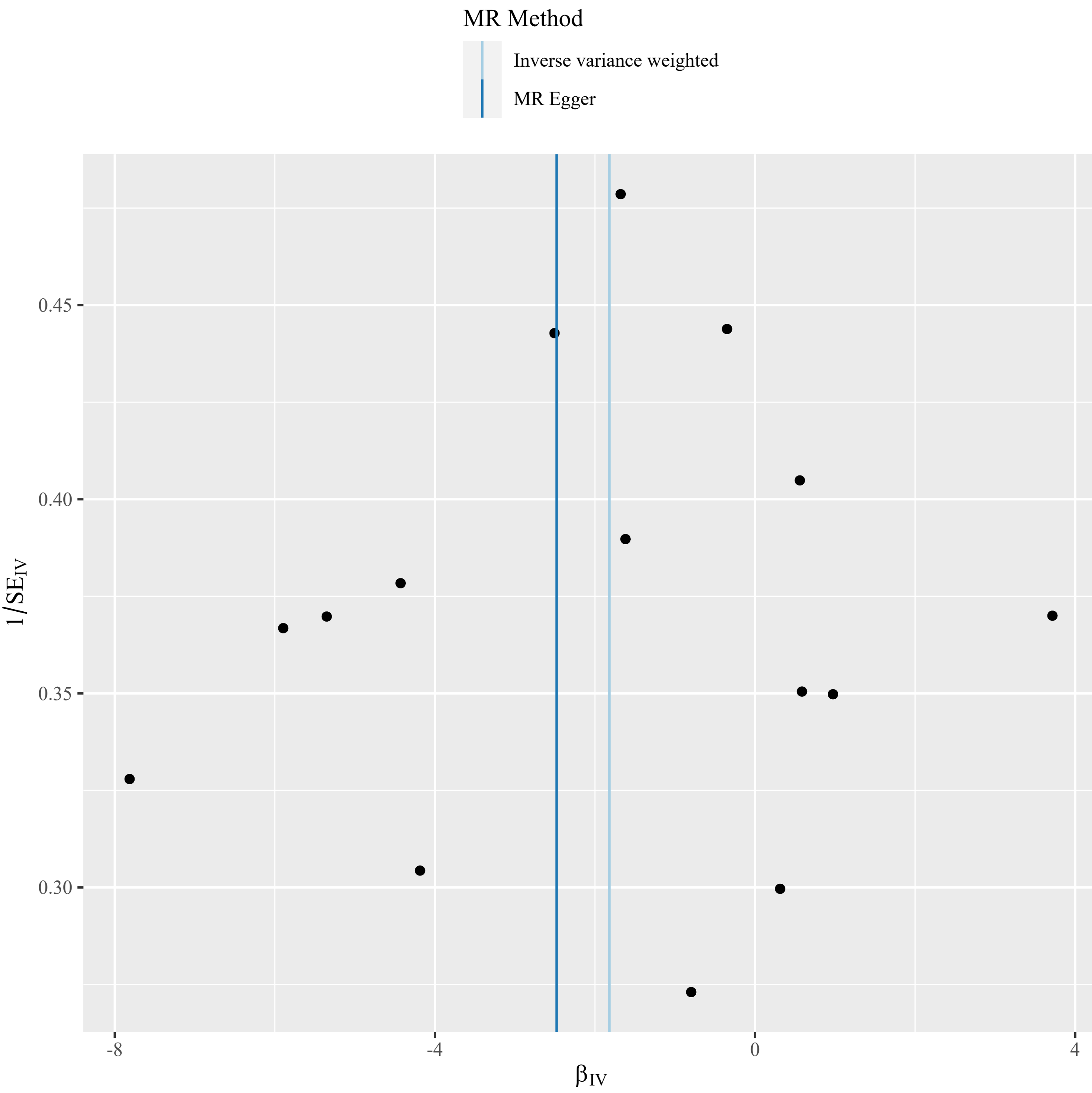

MR Method
Inverse variance weighted
MR Egger
0.45
0.40
0.35
0.30
1/SE_IV
-8
-4
0
4
β_IV

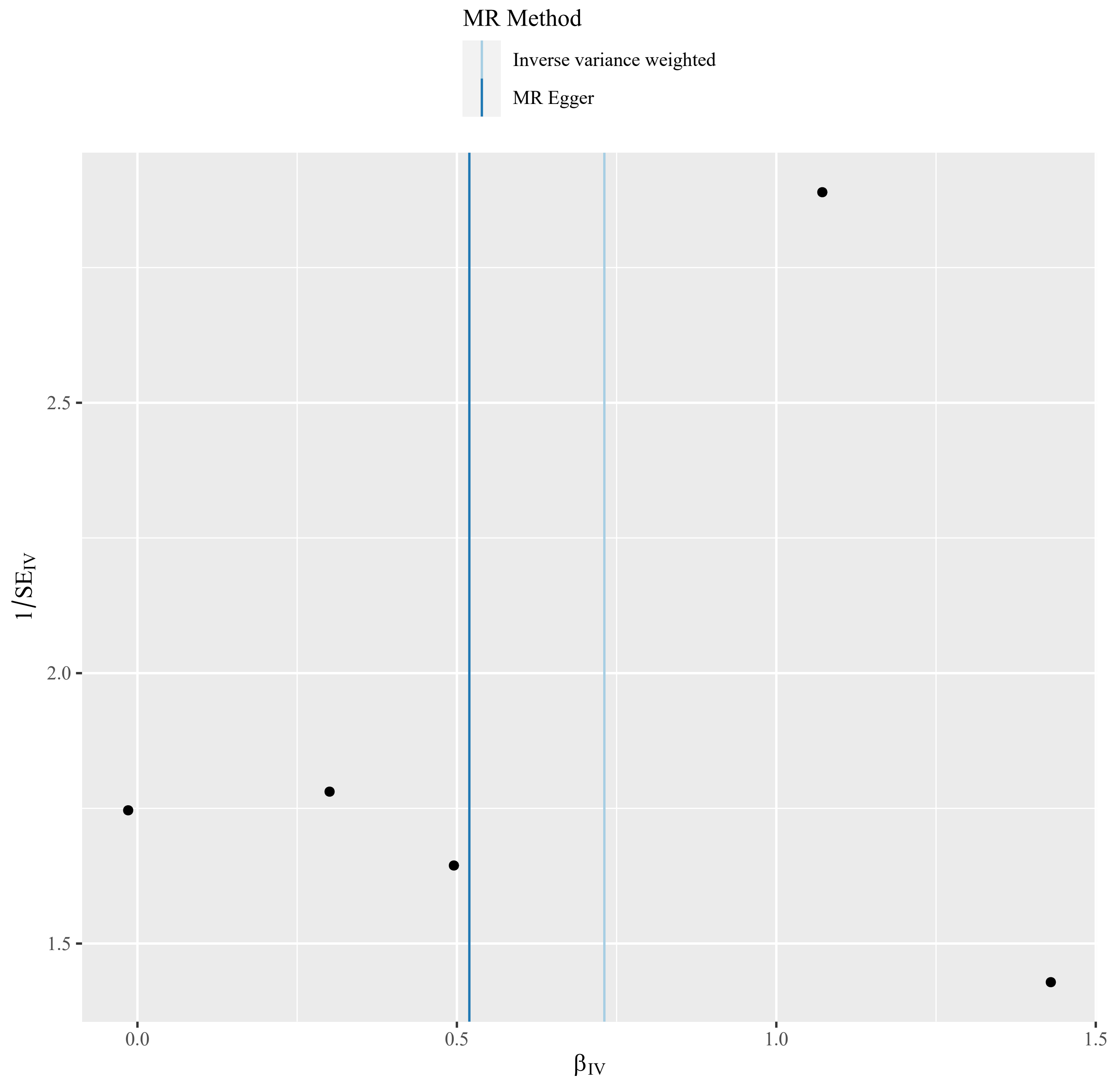

MR Method
Inverse variance weighted
MR Egger
2.5
2.0
1.5
1/SE$_{IV}$
0.0
0.5
1.0
1.5
β$_{IV}$

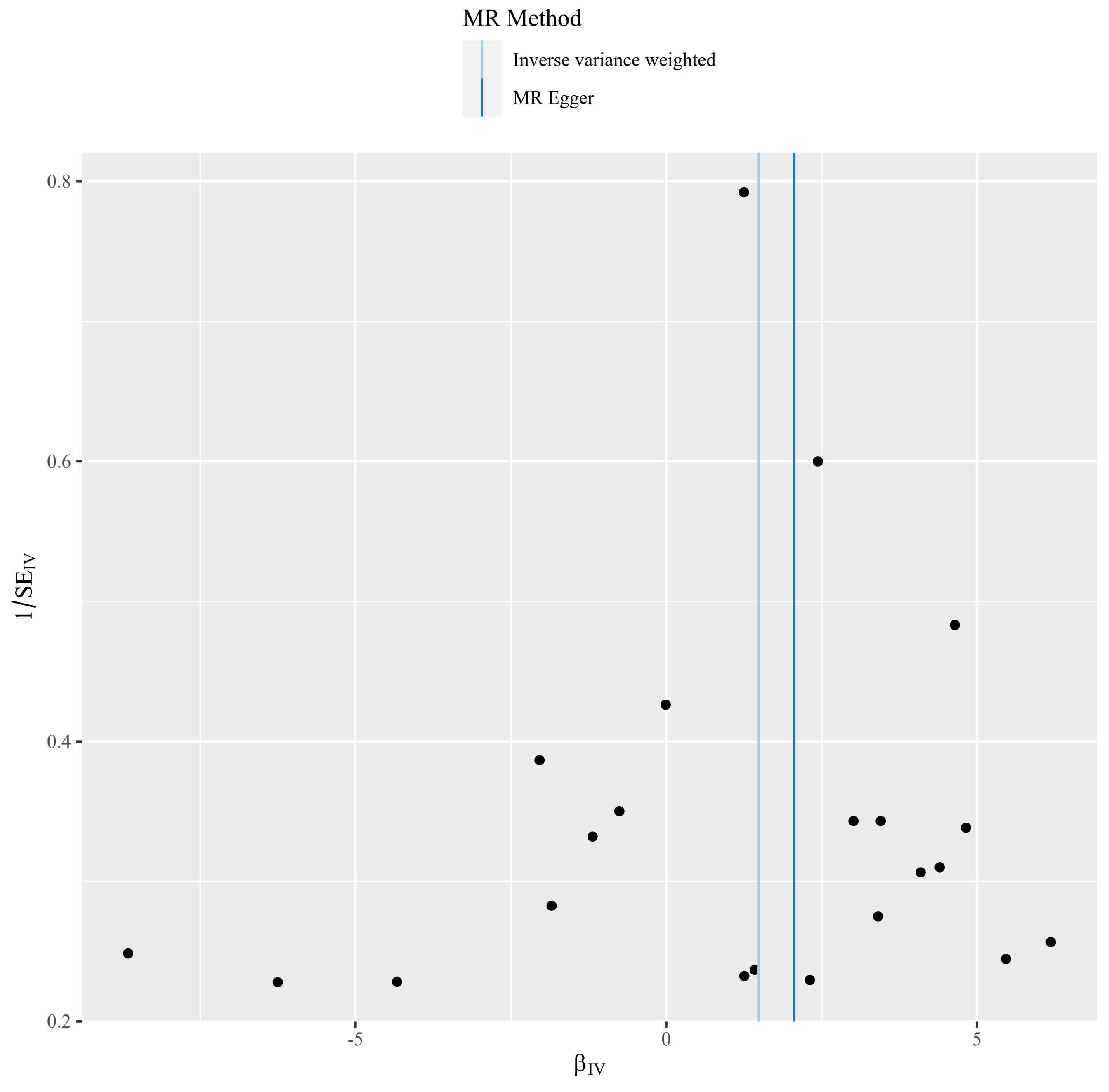

MR Method
Inverse variance weighted
MR Egger
0.8
0.6
0.4
0.2
1/SE_IV
-5
0
5
β_IV

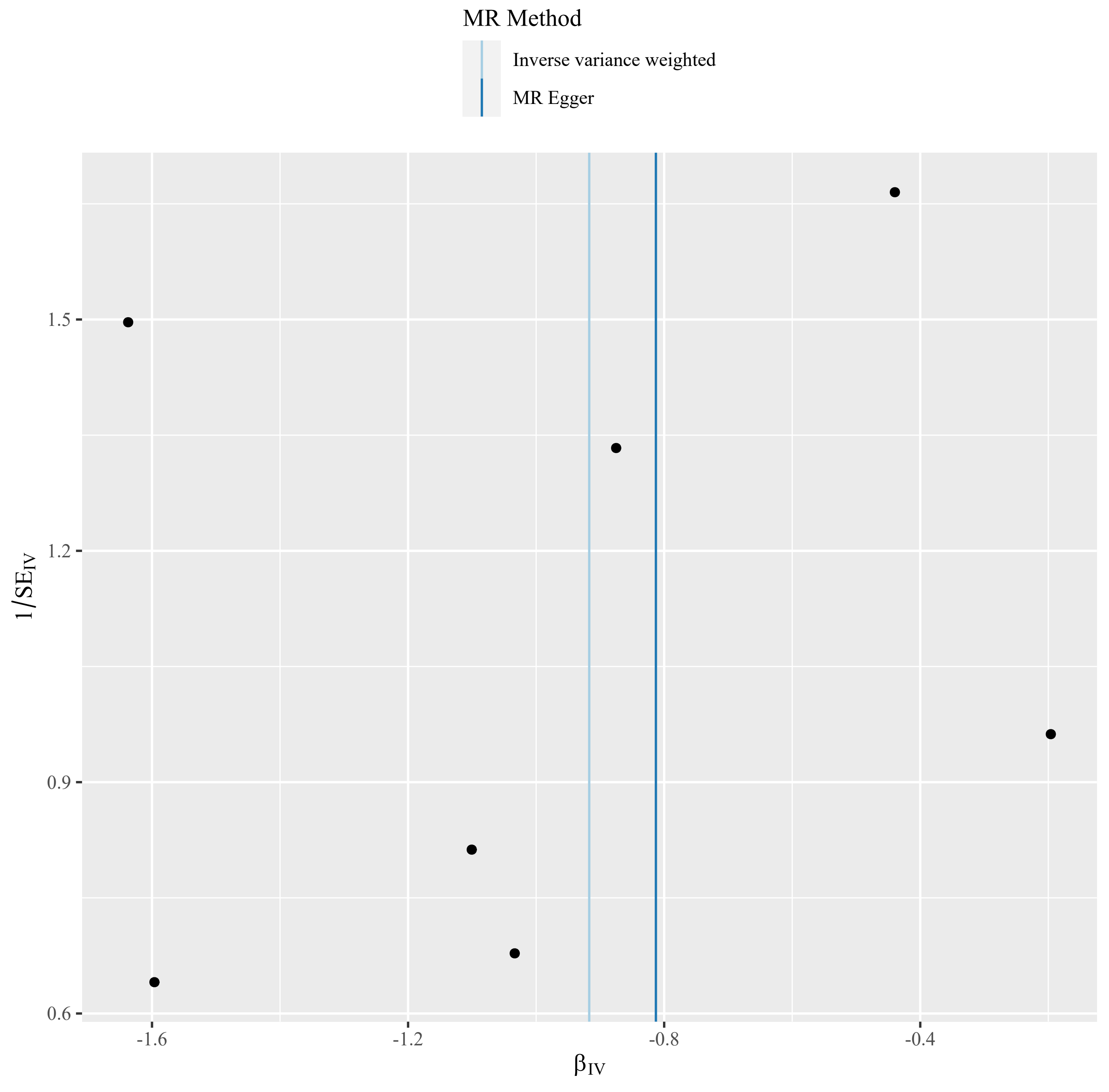
MR Method
Inverse variance weighted
MR Egger
1.5
1.2
0.9
0.6
1/SE$_{IV}$
-1.6
-1.2
-0.8
-0.4
β$_{IV}$

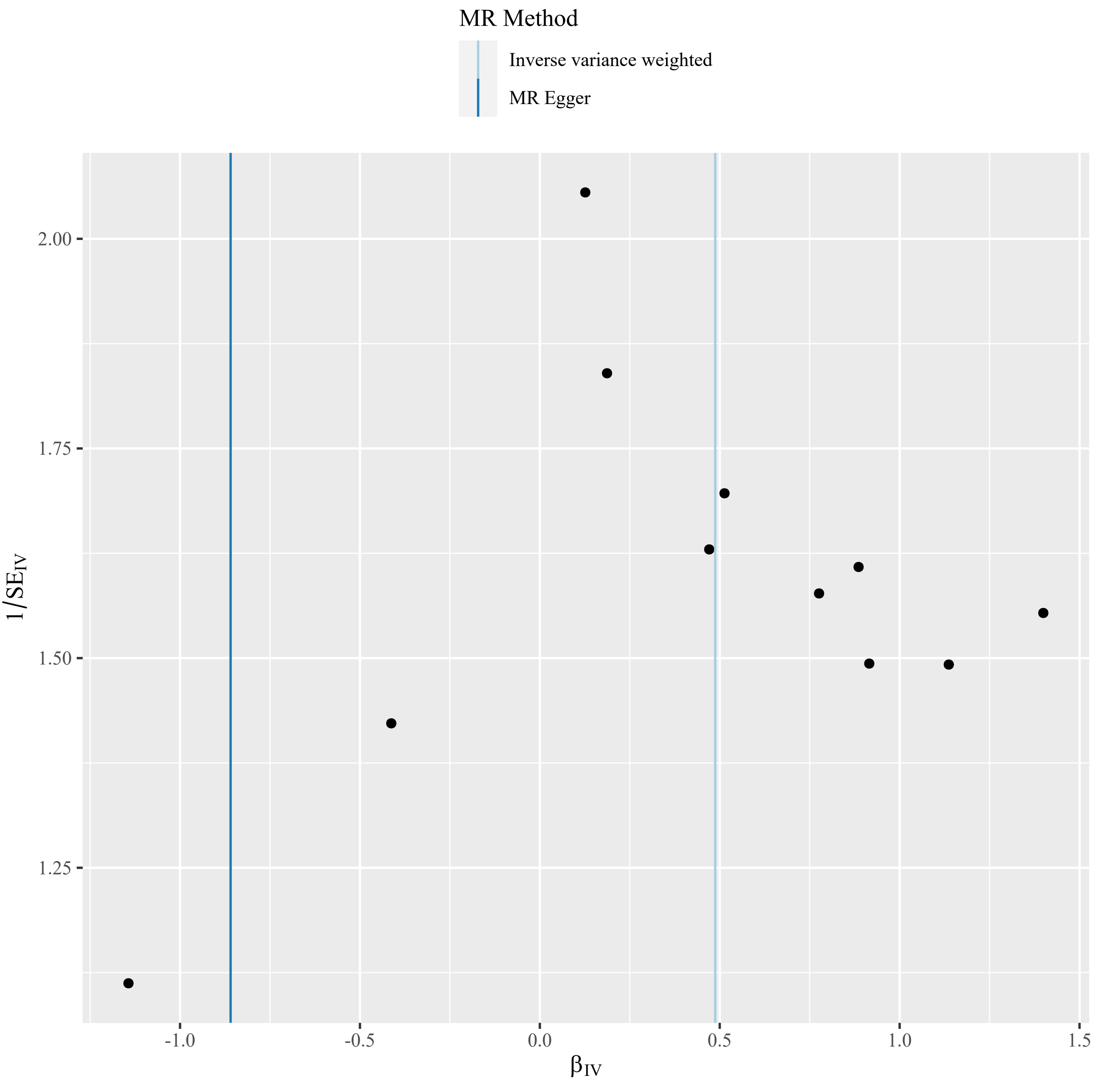

MR Method
Inverse variance weighted
MR Egger
2.00
1.75
1.50
1.25
-1.0
-0.5
0.0
0.5
1.0
1.5
$1/SE_{IV}$
β_{IV}

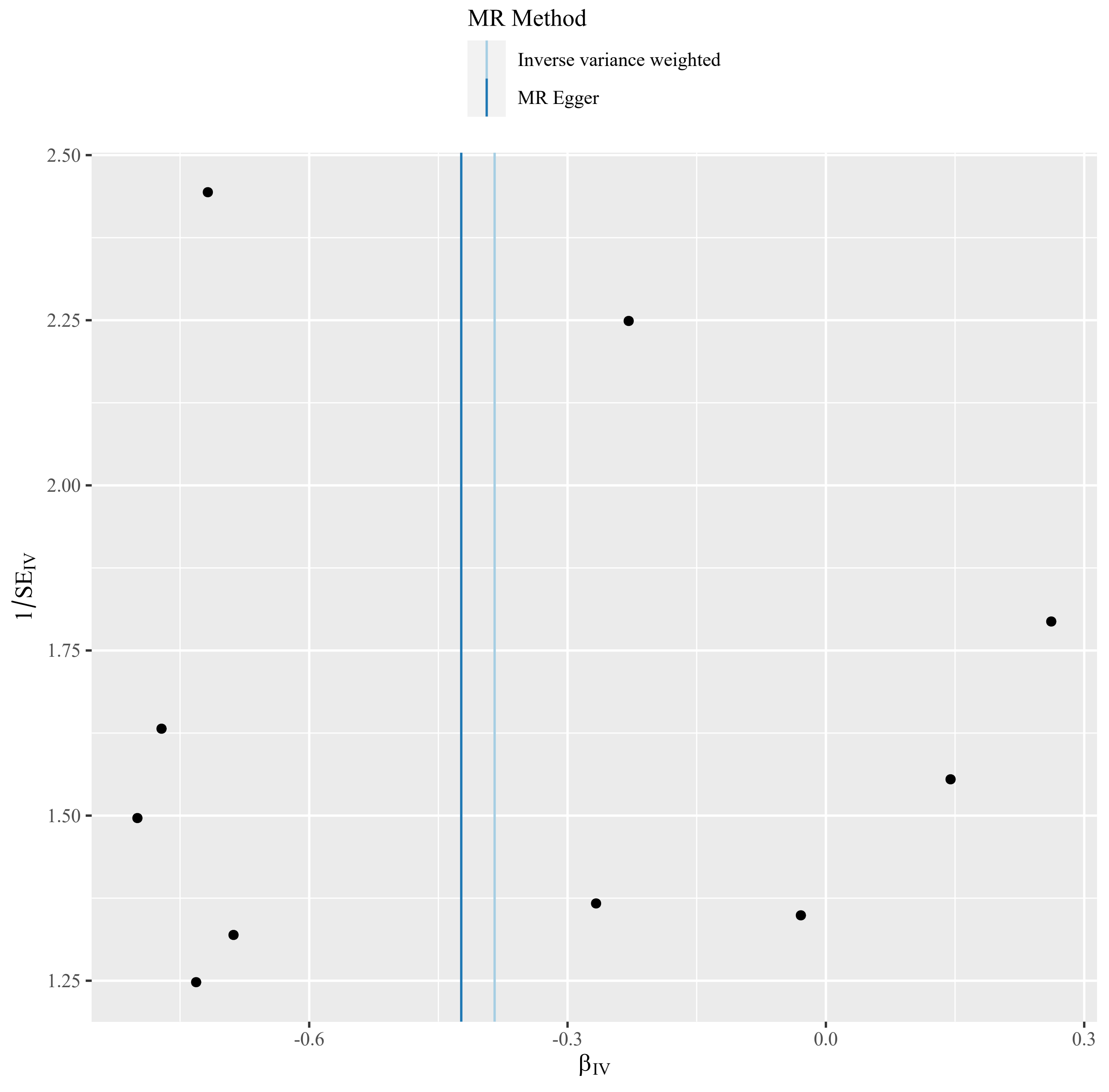
MR Method
Inverse variance weighted
MR Egger
2.50
2.25
2.00
1.75
1.50
1.25
$1/SE_{IV}$
-0.6
-0.3
0.0
0.3
β_{IV}

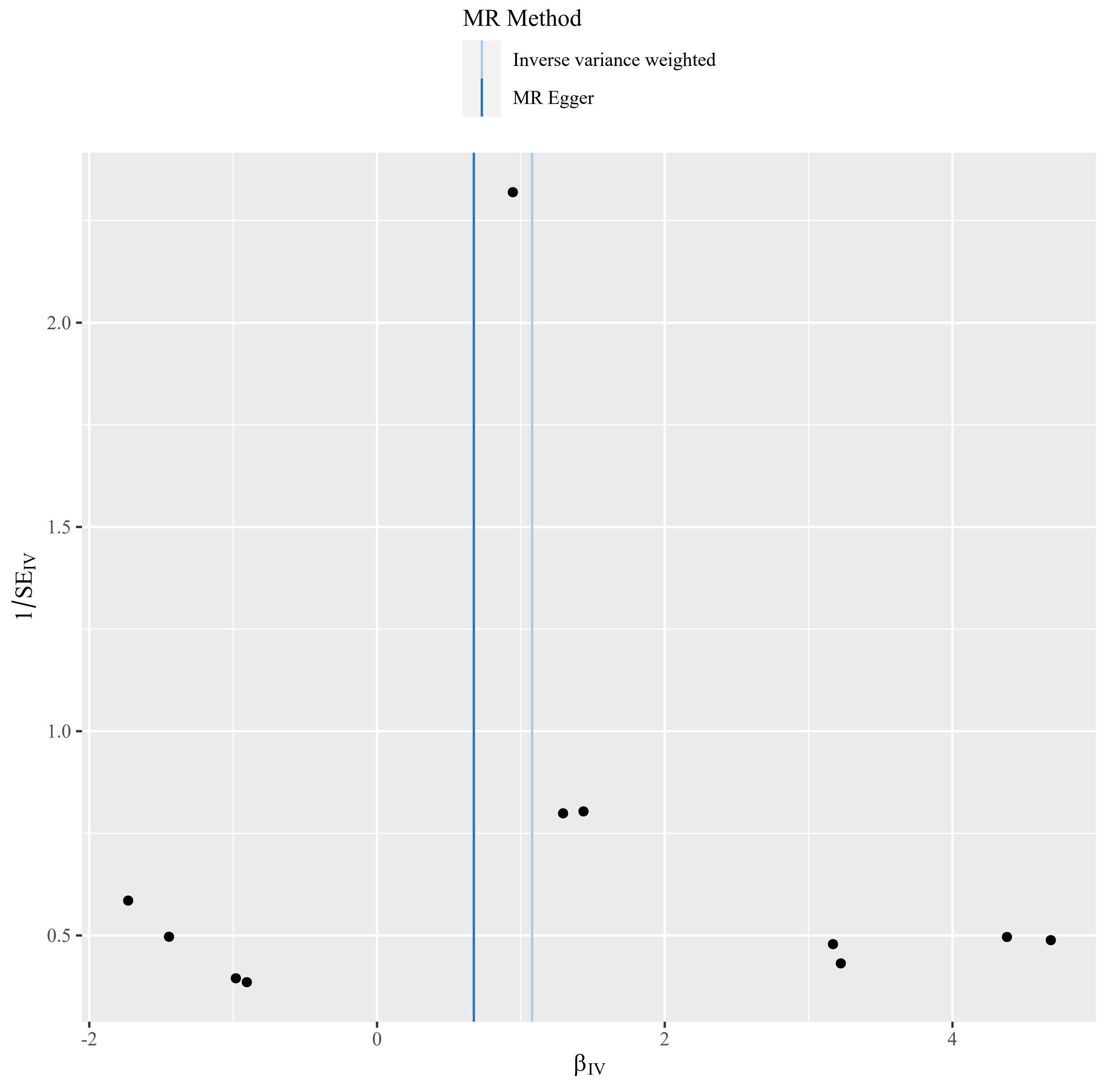

MR Method
Inverse variance weighted
MR Egger
2.0
1.5
1.0
0.5
1/SE_IV
-2
0
2
4
β_IV

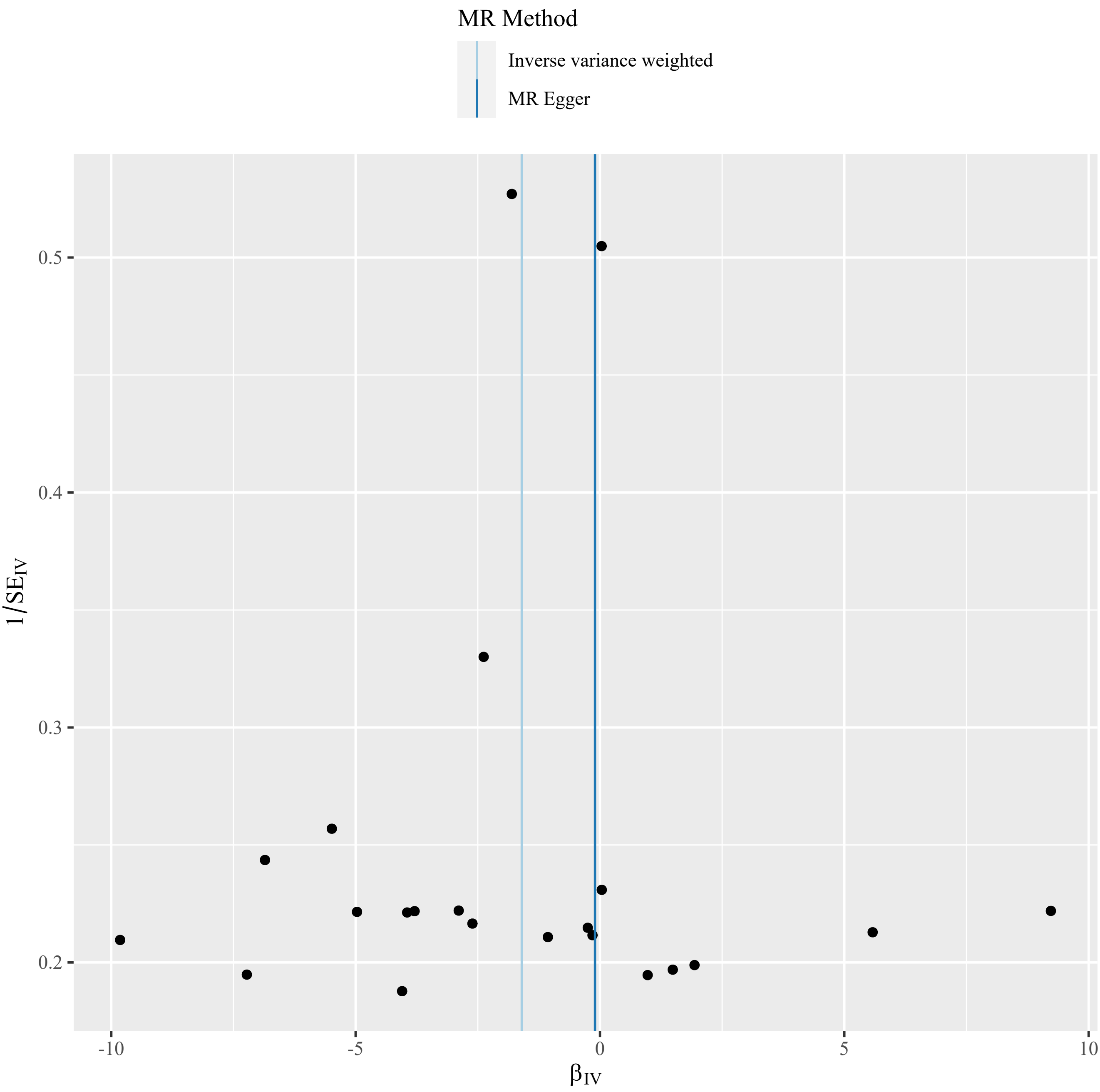
MR Method
Inverse variance weighted
MR Egger
0.5
0.4
0.3
0.2
1/SE$_{IV}$
-10
-5
0
5
10
β$_{IV}$

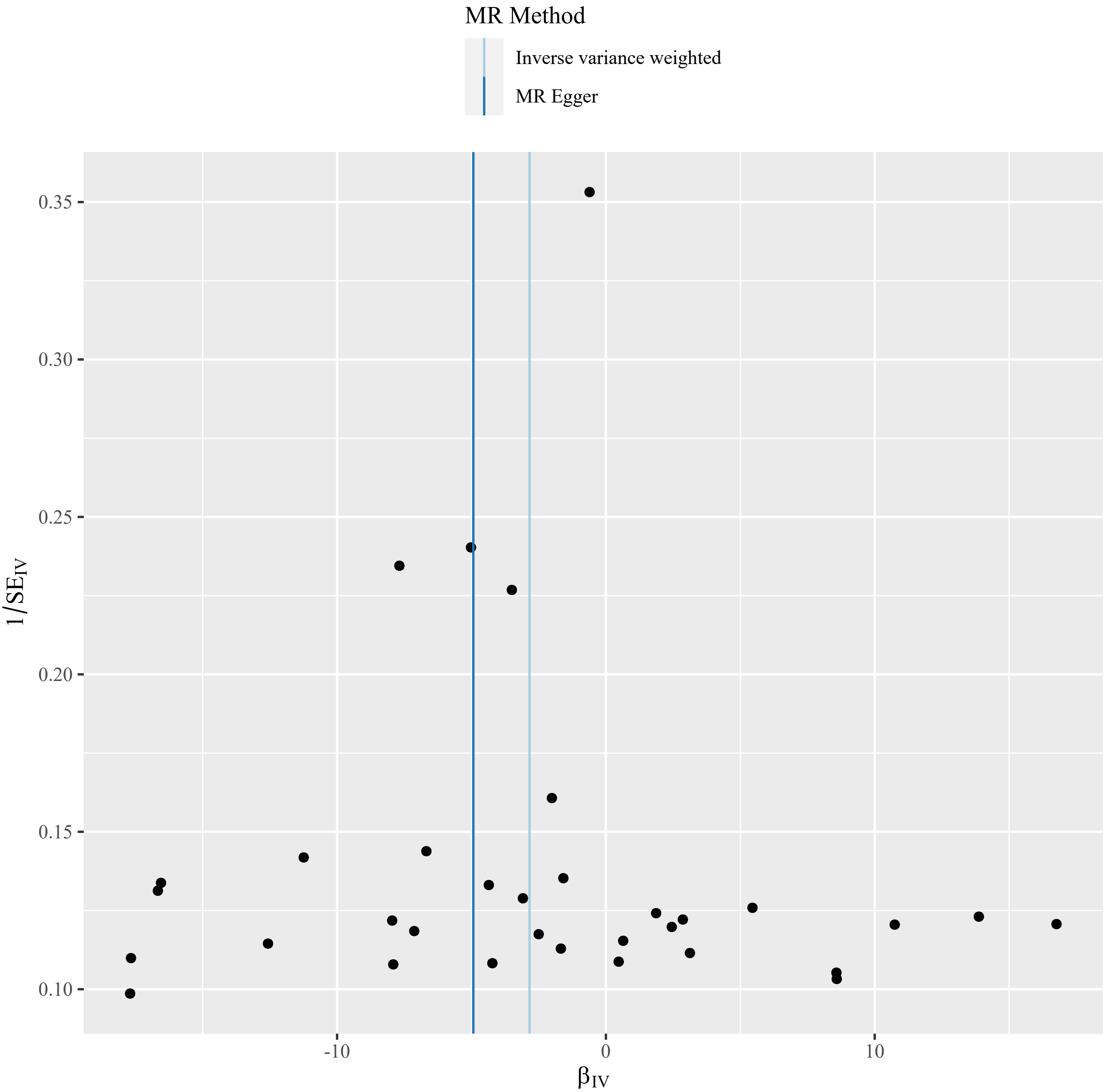
MR Method
Inverse variance weighted
MR Egger
0.35
0.30
0.25
0.20
0.15
0.10
$1/SE_{IV}$
-10
0
10
β_{IV}

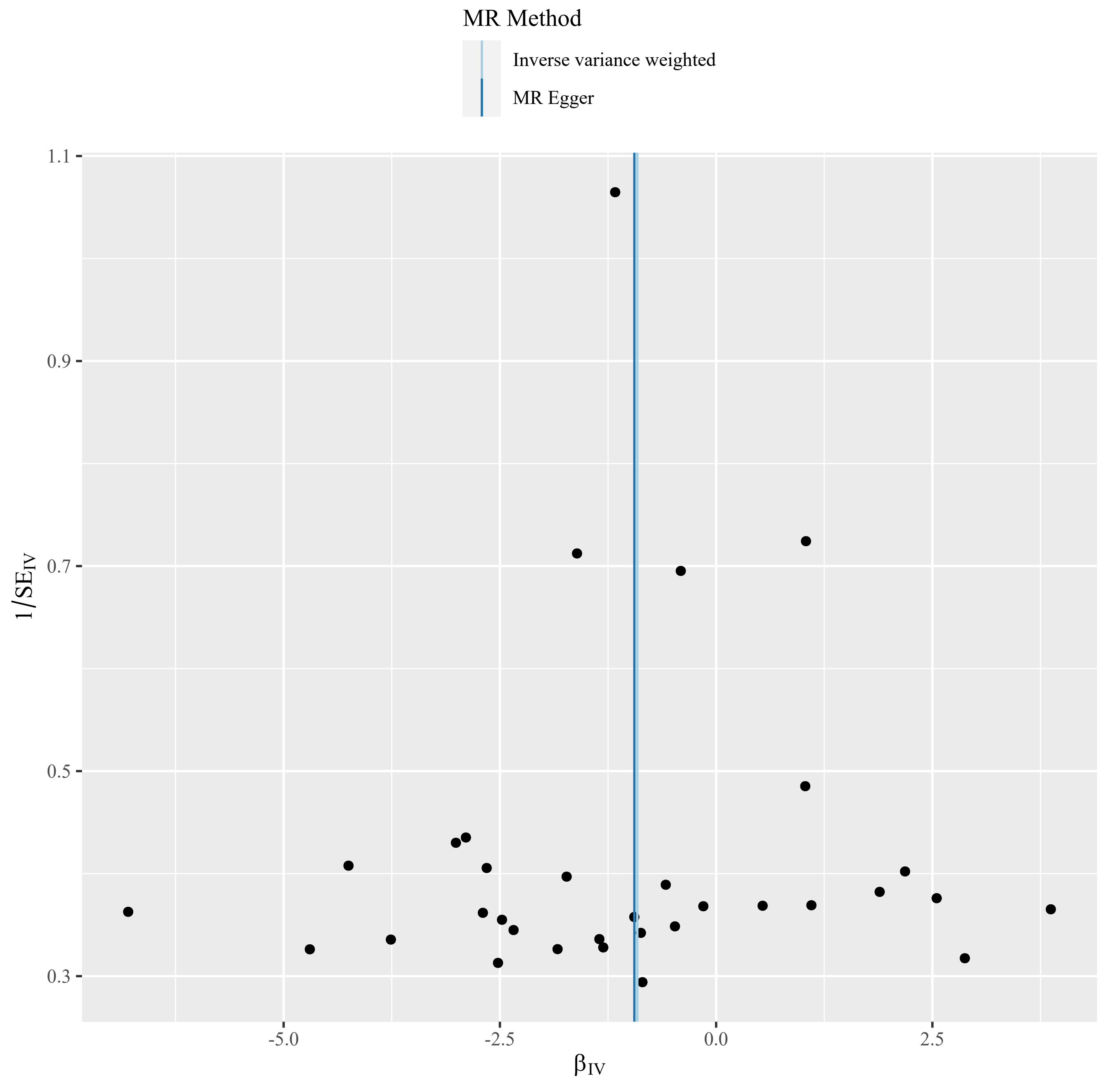

MR Method
Inverse variance weighted
MR Egger
1.1
0.9
0.7
0.5
0.3
1/SE_IV
-5.0
-2.5
0.0
2.5
β_IV

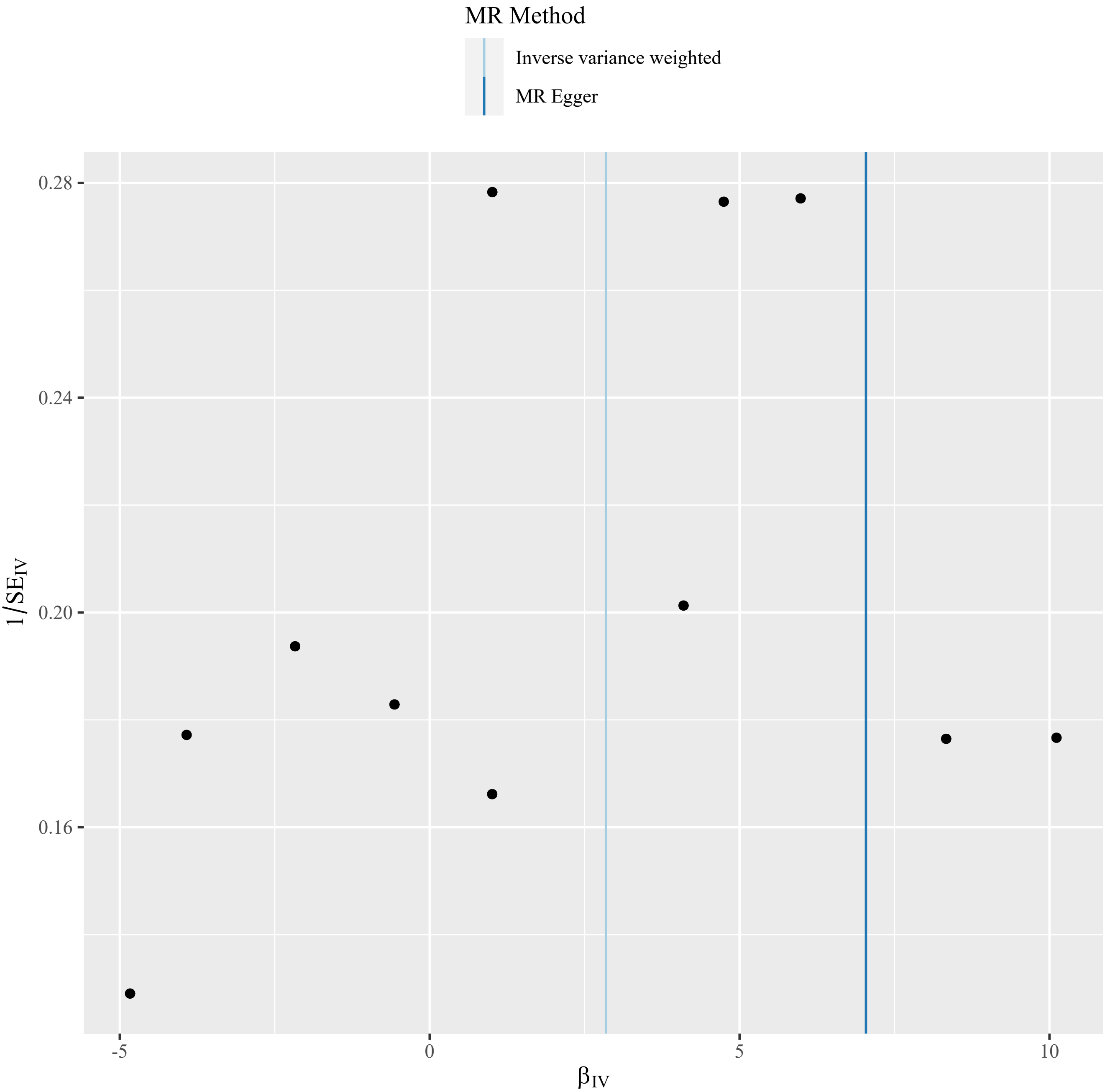
MR Method
Inverse variance weighted
MR Egger
0.28
0.24
0.20
0.16
1/SE_IV
-5
0
5
10
β_IV

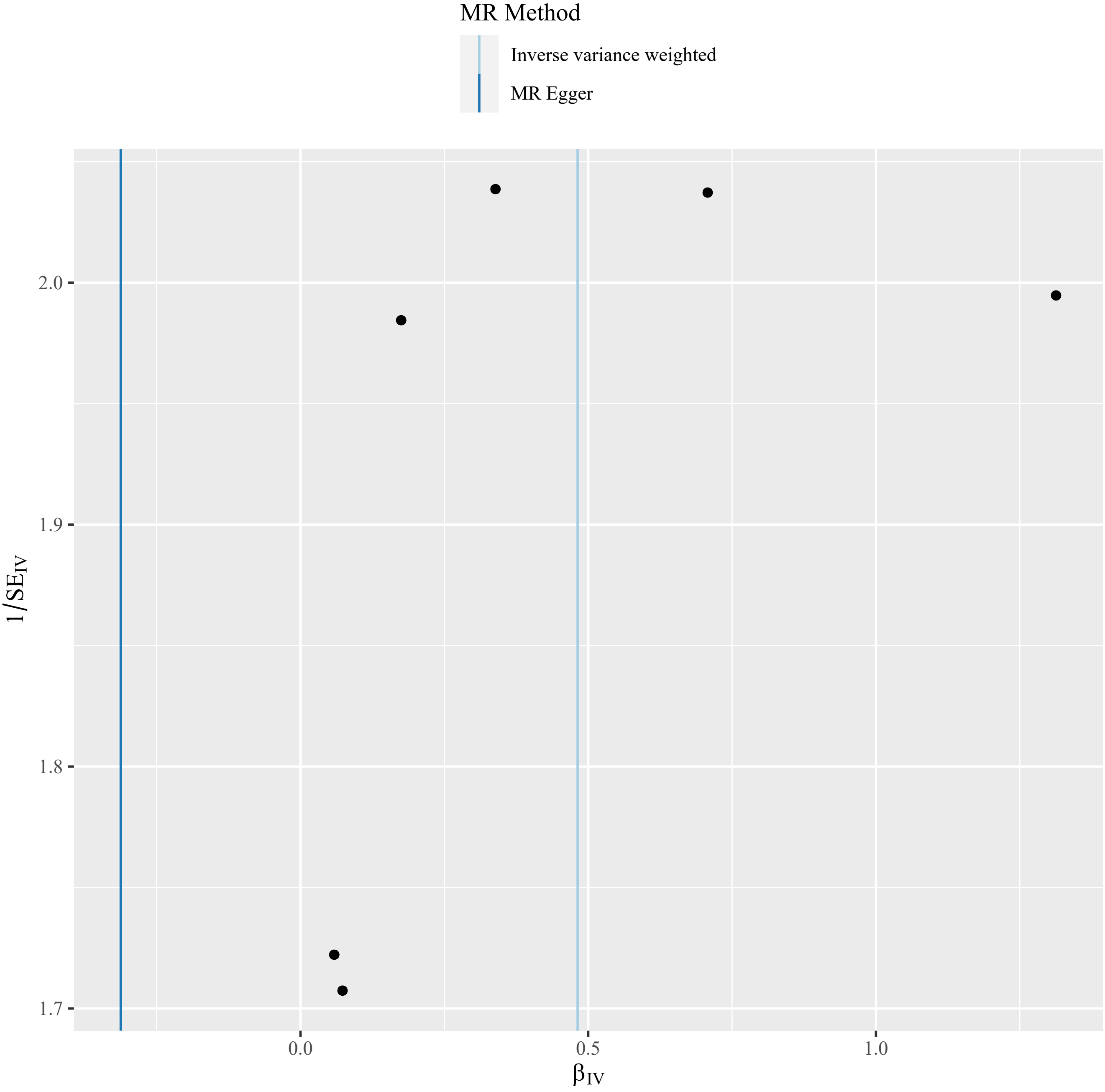
MR Method
Inverse variance weighted
MR Egger
2.0
1.9
1.8
1.7
1/SE_IV
0.0
0.5
1.0
β_IV

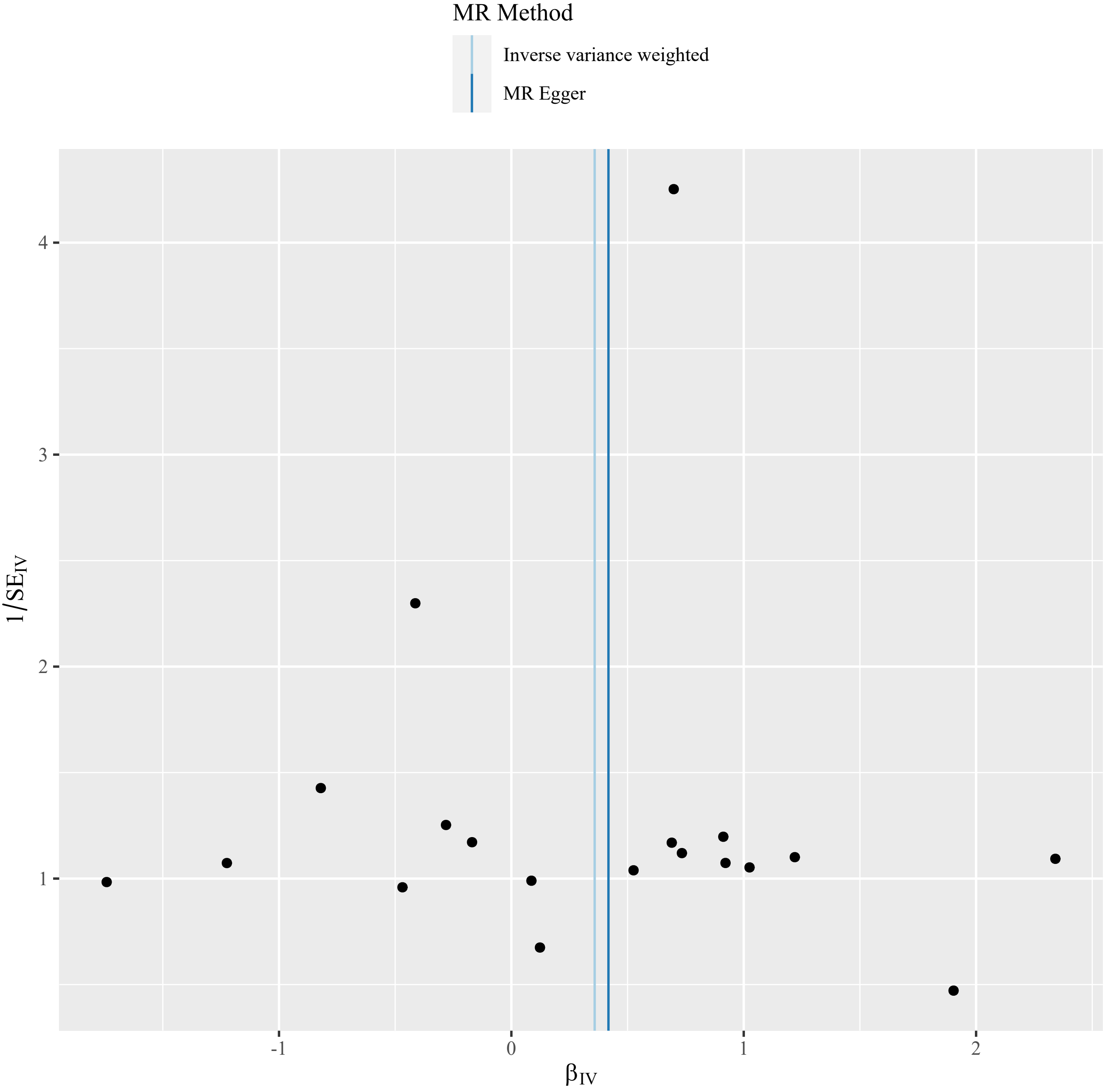

MR Method
Inverse variance weighted
MR Egger
4
3
2
1
$1/SE_{IV}$
-1
0
1
2
β_{IV}

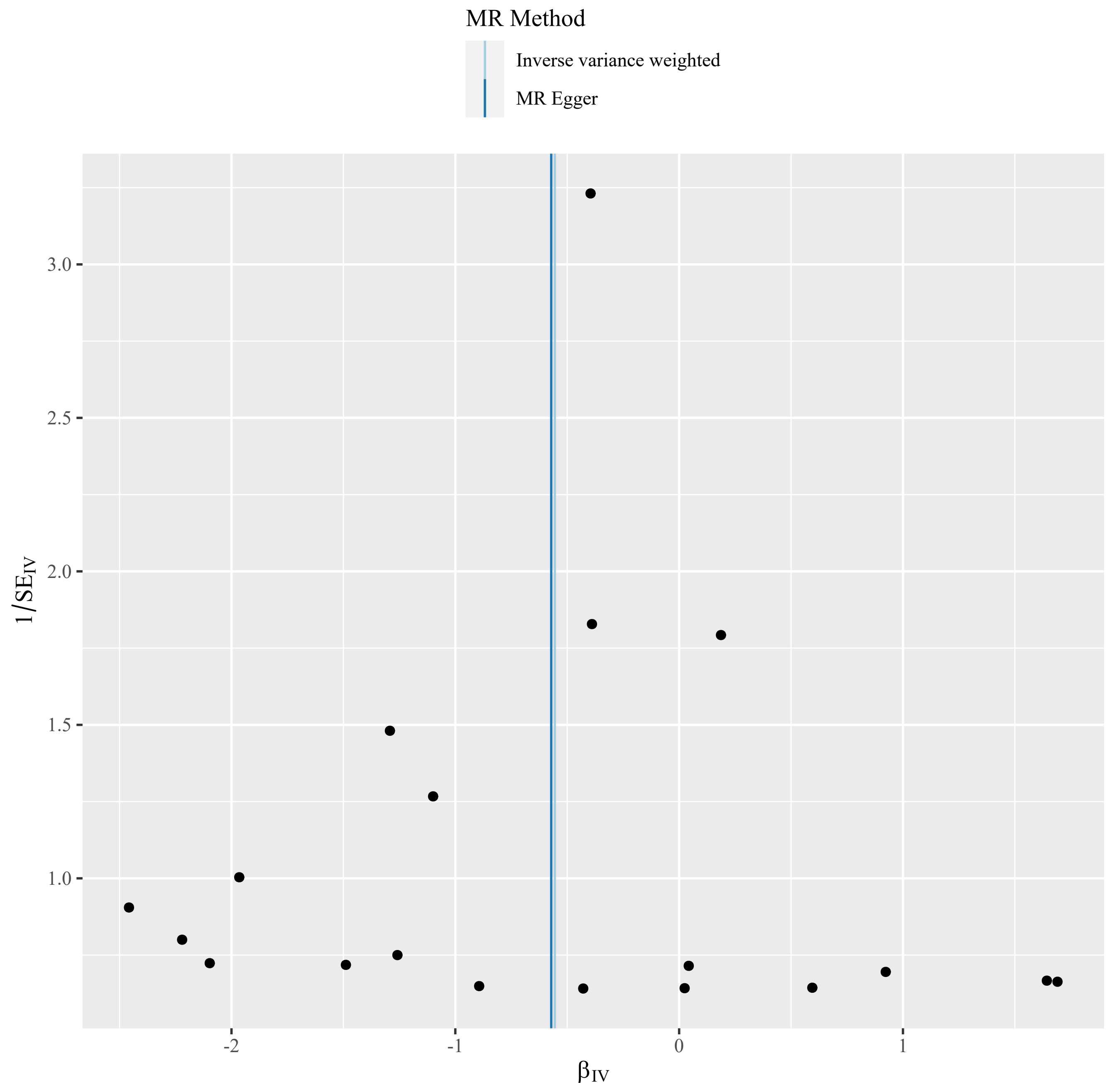
MR Method
Inverse variance weighted
MR Egger
1/SE_{IV}
3.0
2.5
2.0
1.5
1.0
-2
-1
0
1
β_{IV}

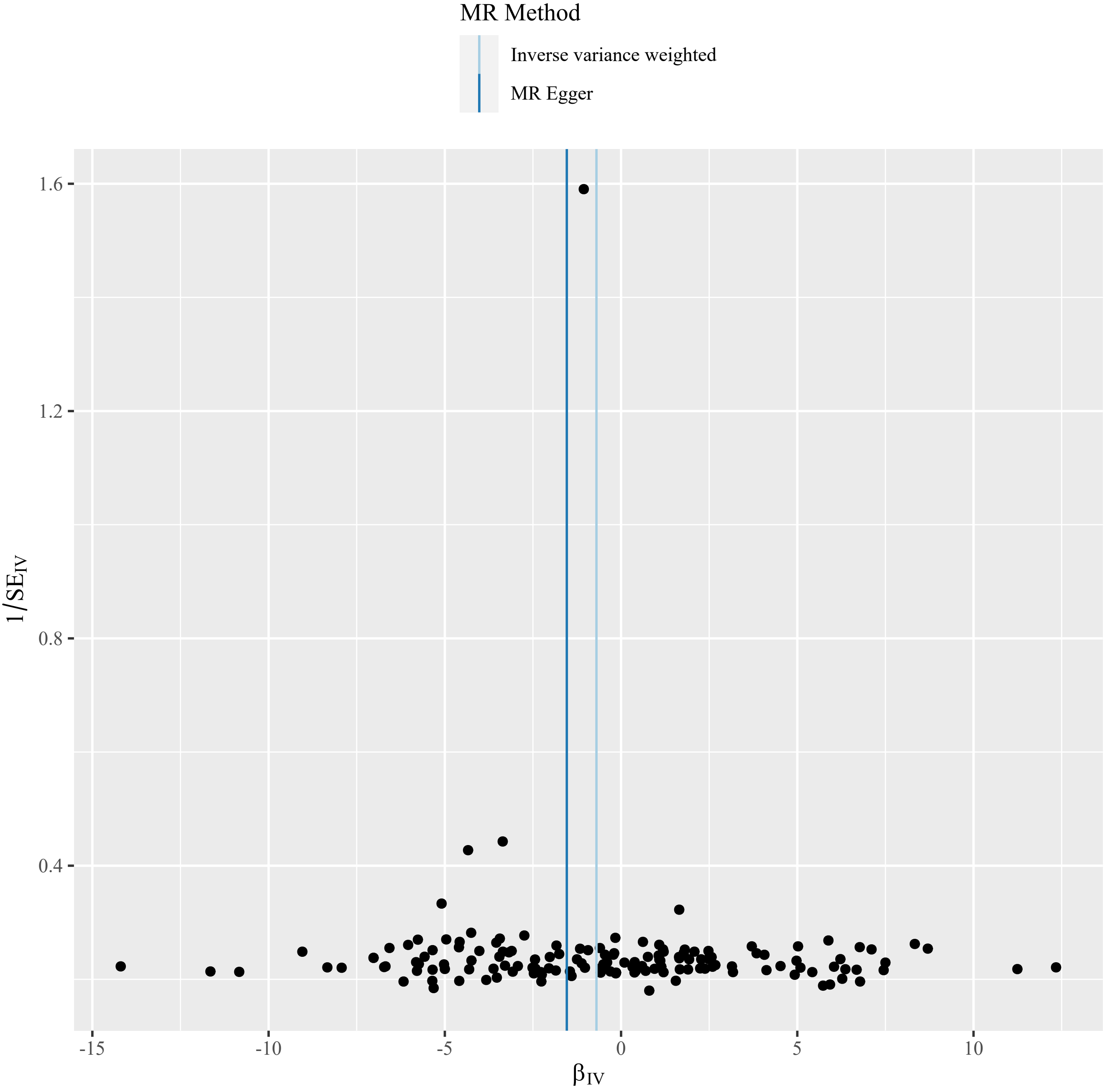
MR Method
Inverse variance weighted
MR Egger
1.6
1.2
0.8
0.4
1/SE_IV
-15
-10
-5
0
5
10
β_IV

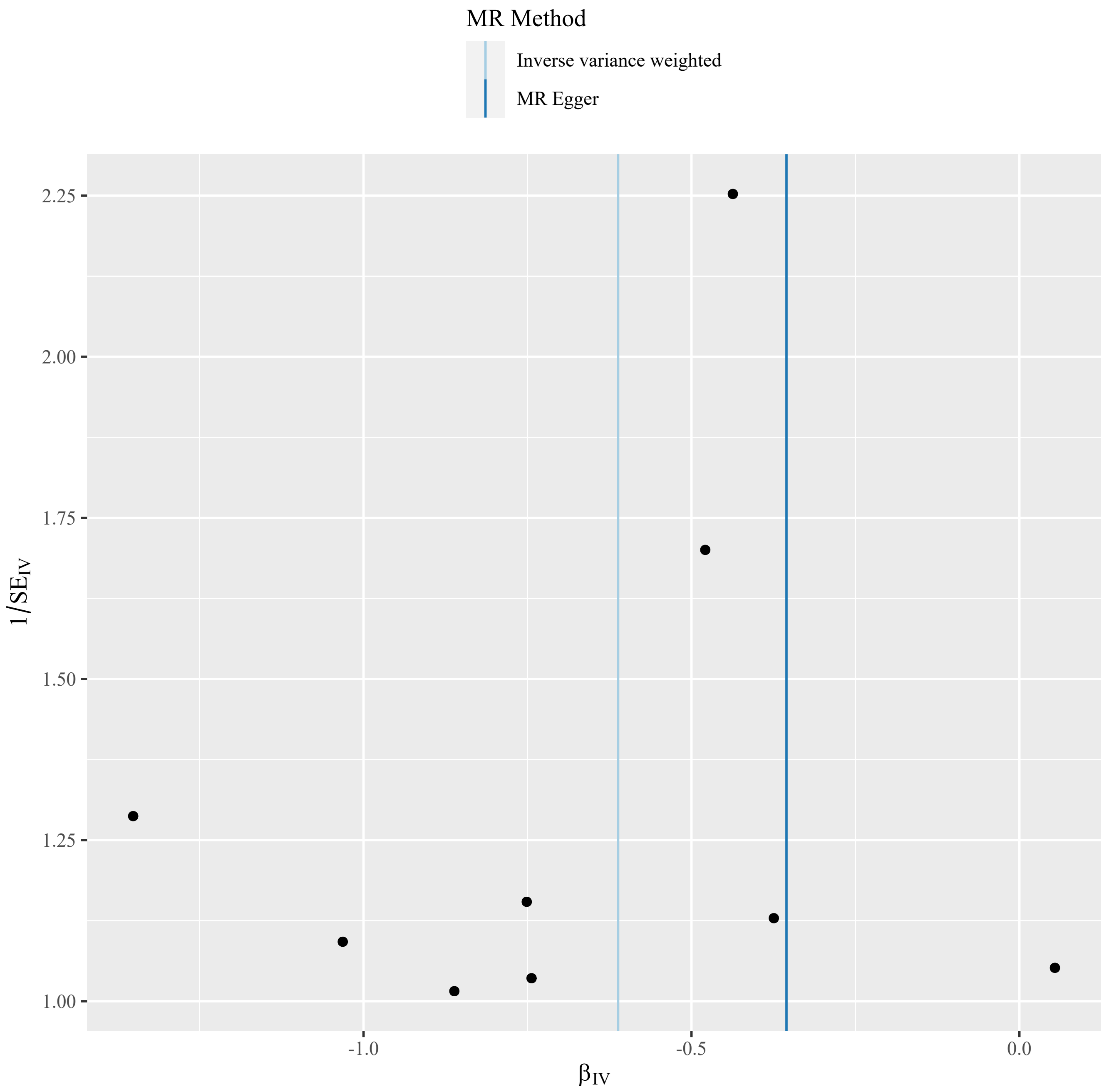

MR Method
Inverse variance weighted
MR Egger
2.25
2.00
1.75
1.50
1.25
1.00
$1/SE_{IV}$
-1.0
-0.5
0.0
β_{IV}

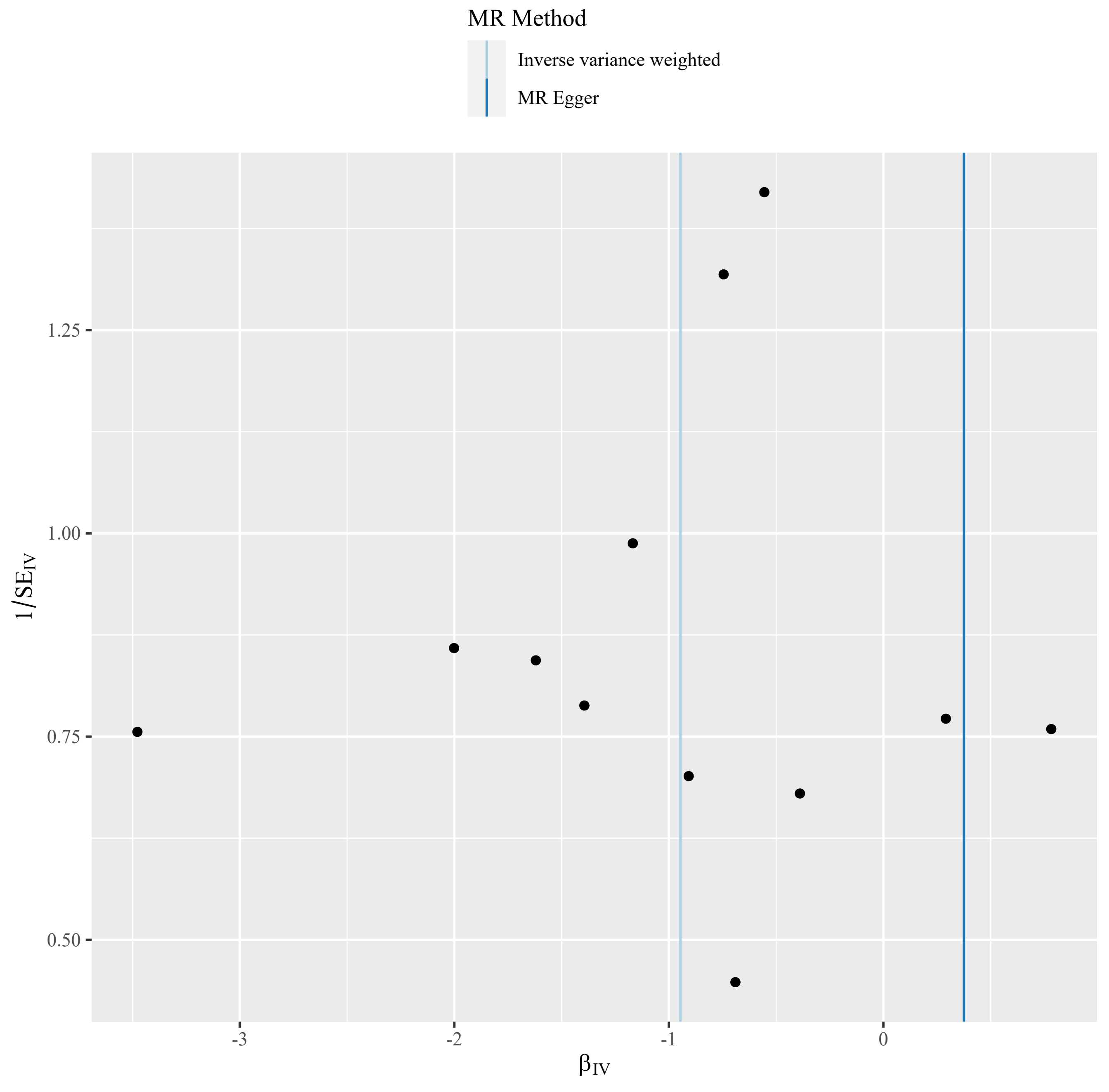
MR Method
Inverse variance weighted
MR Egger
1.25
1.00
0.75
0.50
1/SE_IV
-3
-2
-1
0
β_IV

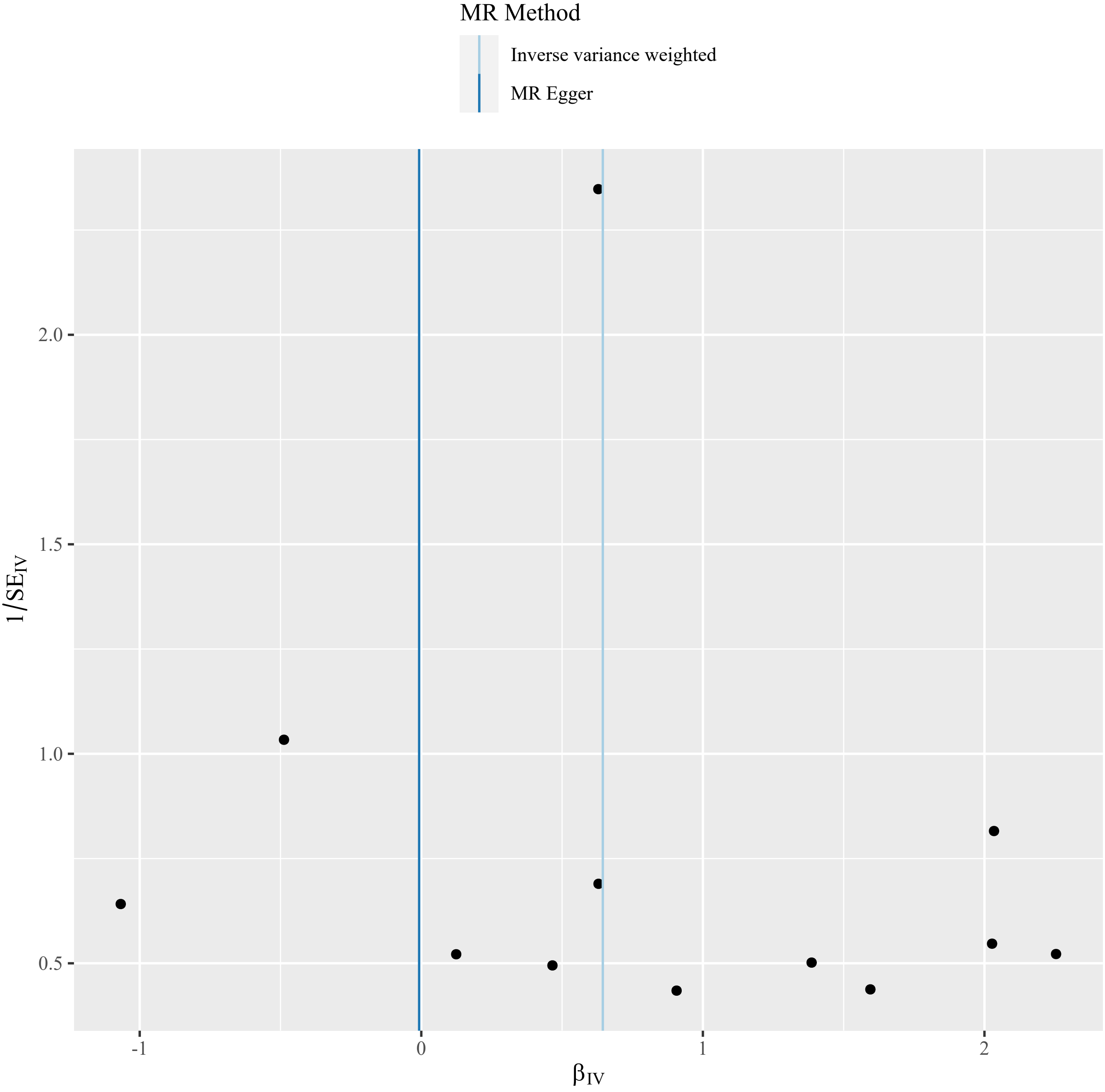
MR Method
Inverse variance weighted
MR Egger
2.0
1.5
1.0
0.5
1/SE$_{IV}$
-1
0
1
2
β$_{IV}$

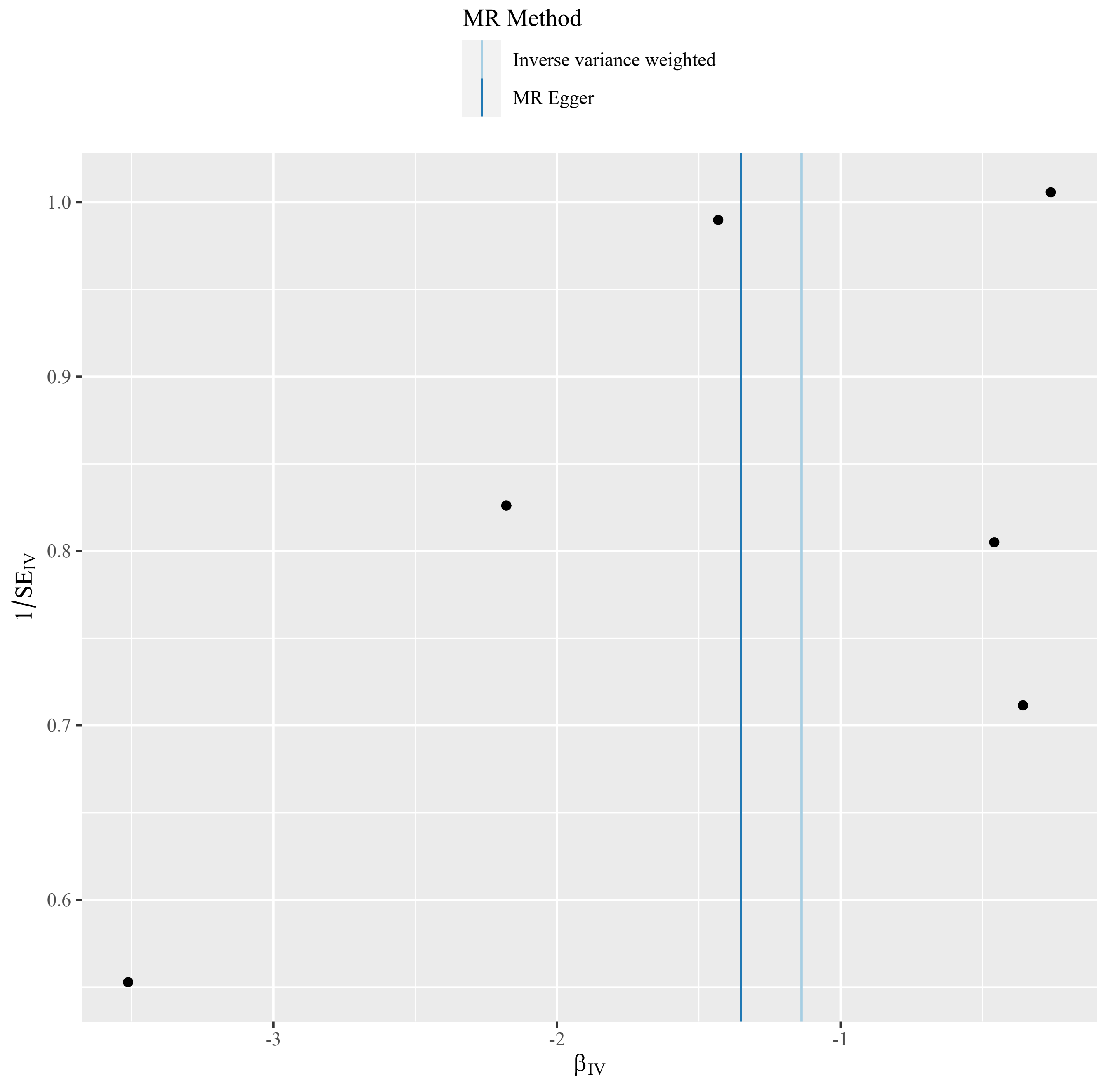
MR Method
Inverse variance weighted
MR Egger
1.0
0.9
0.8
0.7
0.6
1/SE$_{IV}$
-3
-2
-1
β$_{IV}$

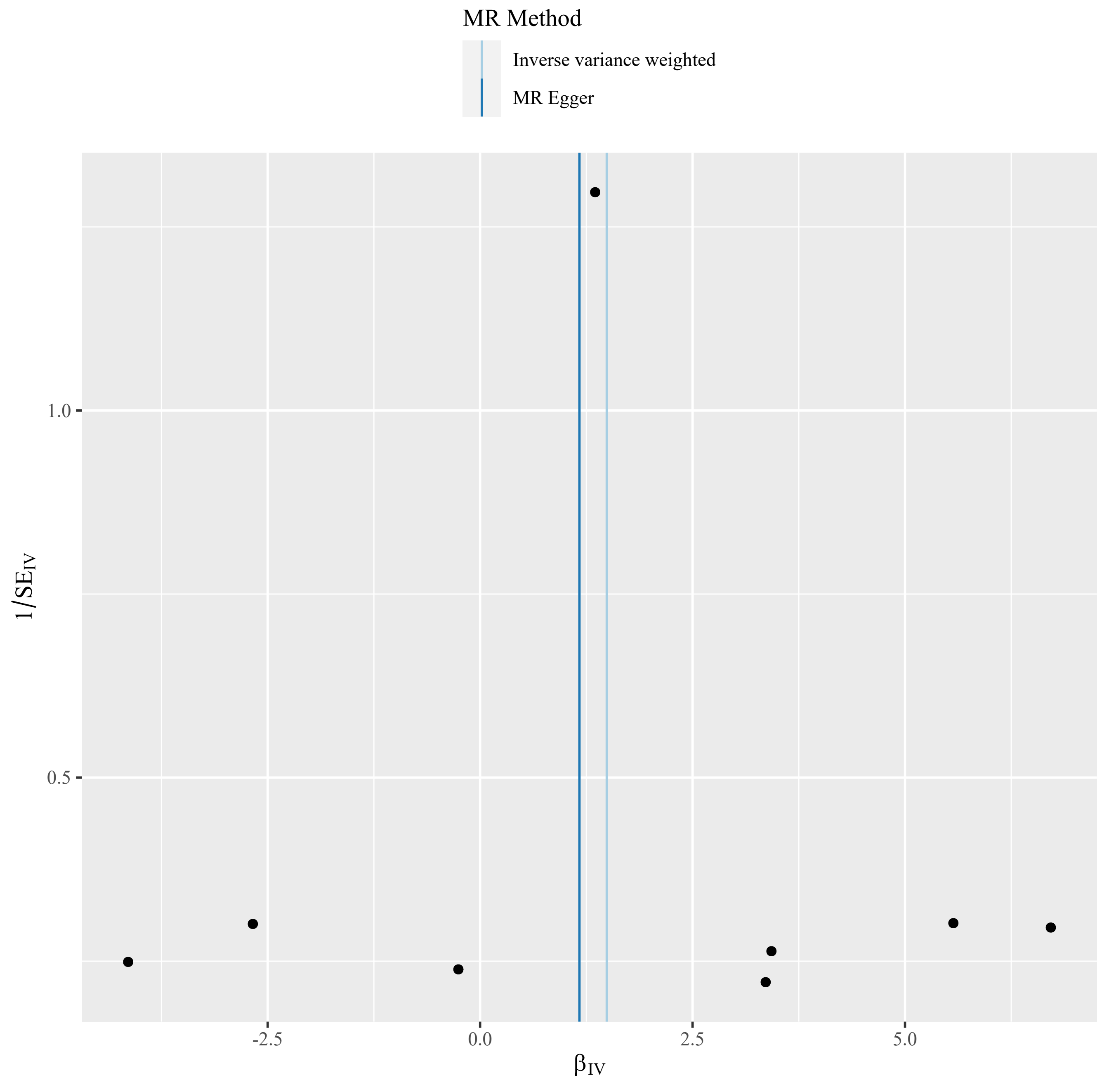
MR Method
Inverse variance weighted
MR Egger
1.0
0.5
-2.5
0.0
2.5
5.0
$1/SE_{IV}$
β_{IV}

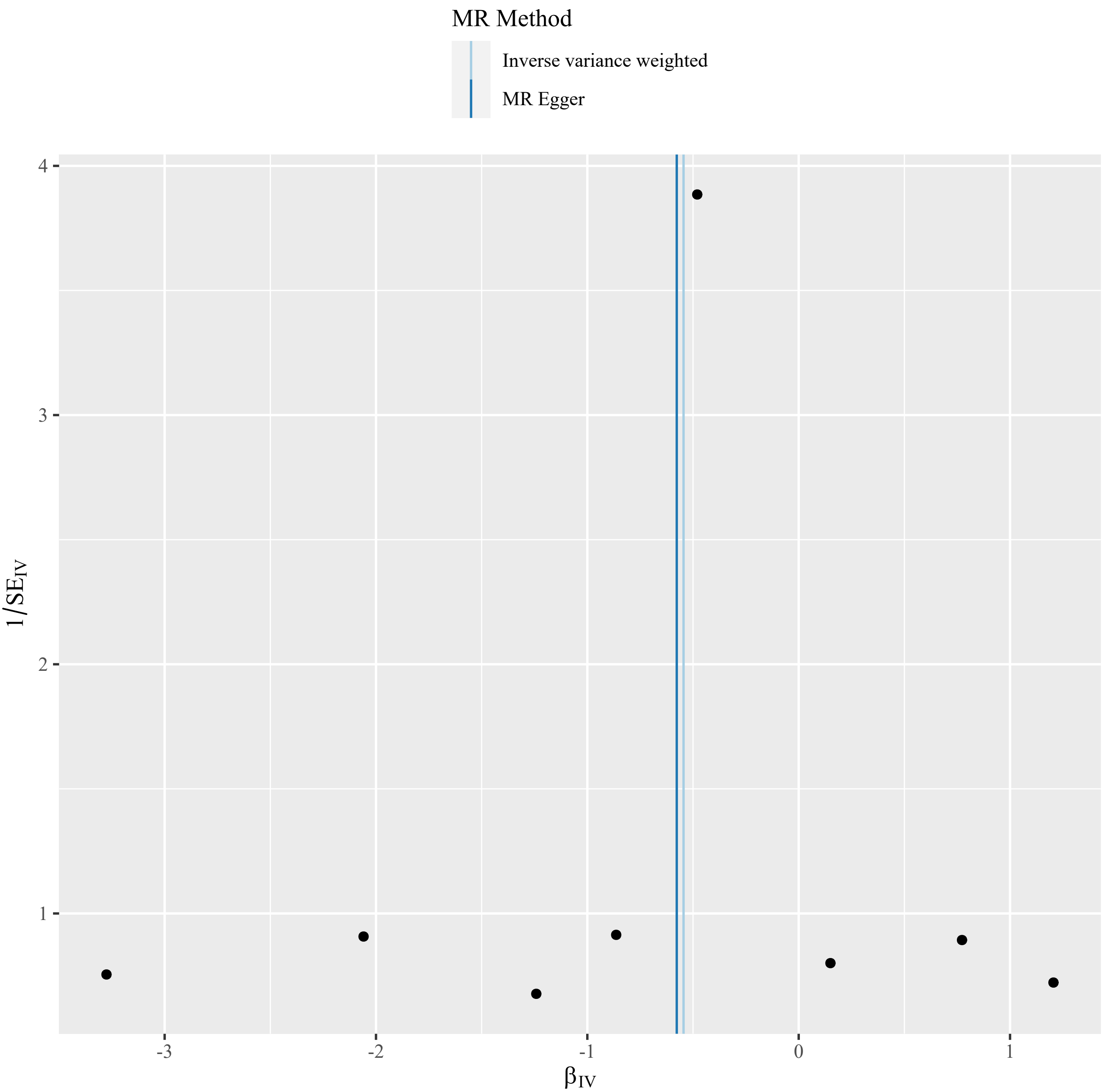
MR Method
Inverse variance weighted
MR Egger
4
3
2
1
1/SE$_{IV}$
-3
-2
-1
0
1
β$_{IV}$

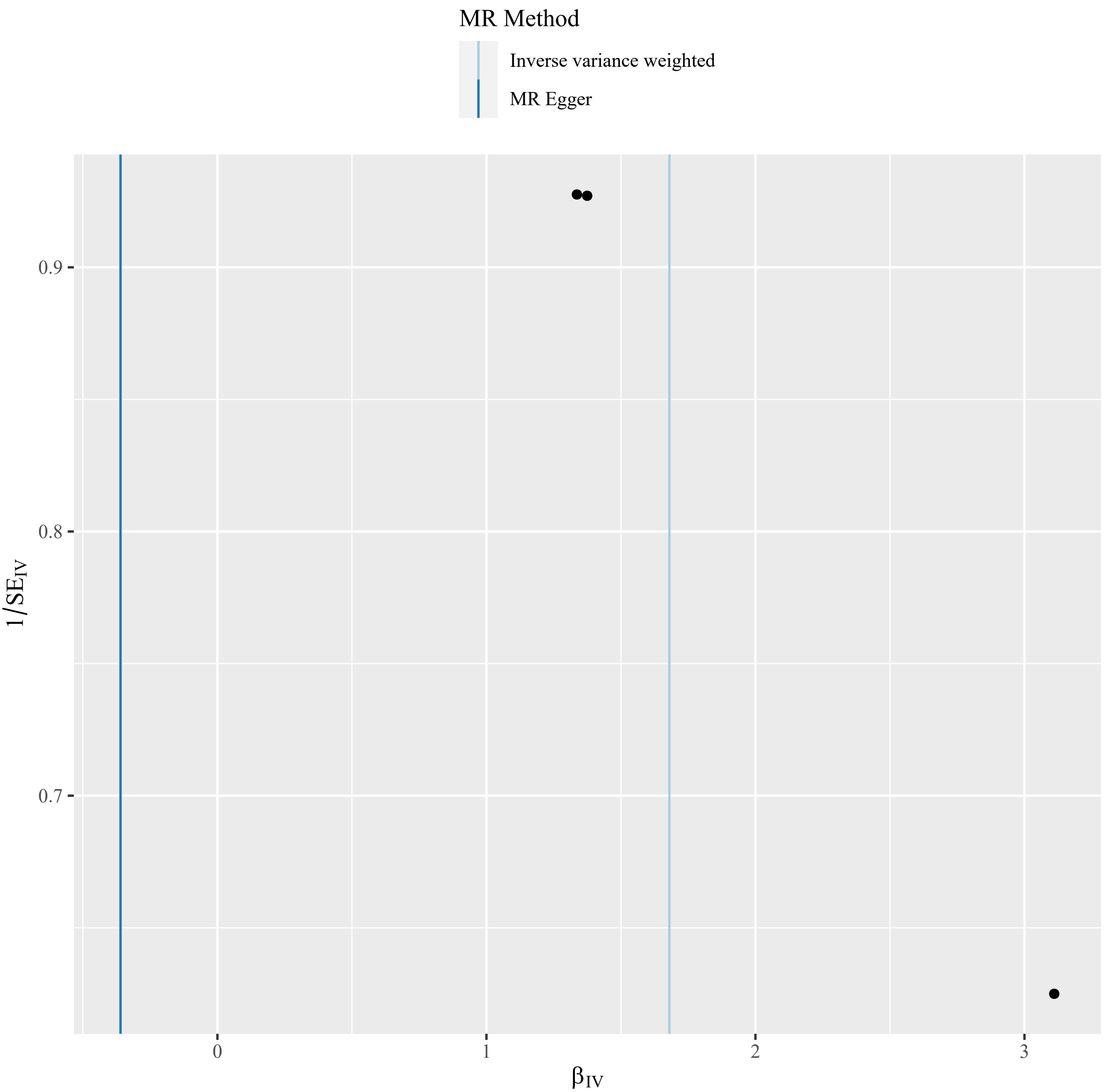
MR Method
Inverse variance weighted
MR Egger
0.9
0.8
0.7
$1/SE_{IV}$
0
1
2
3
β_{IV}

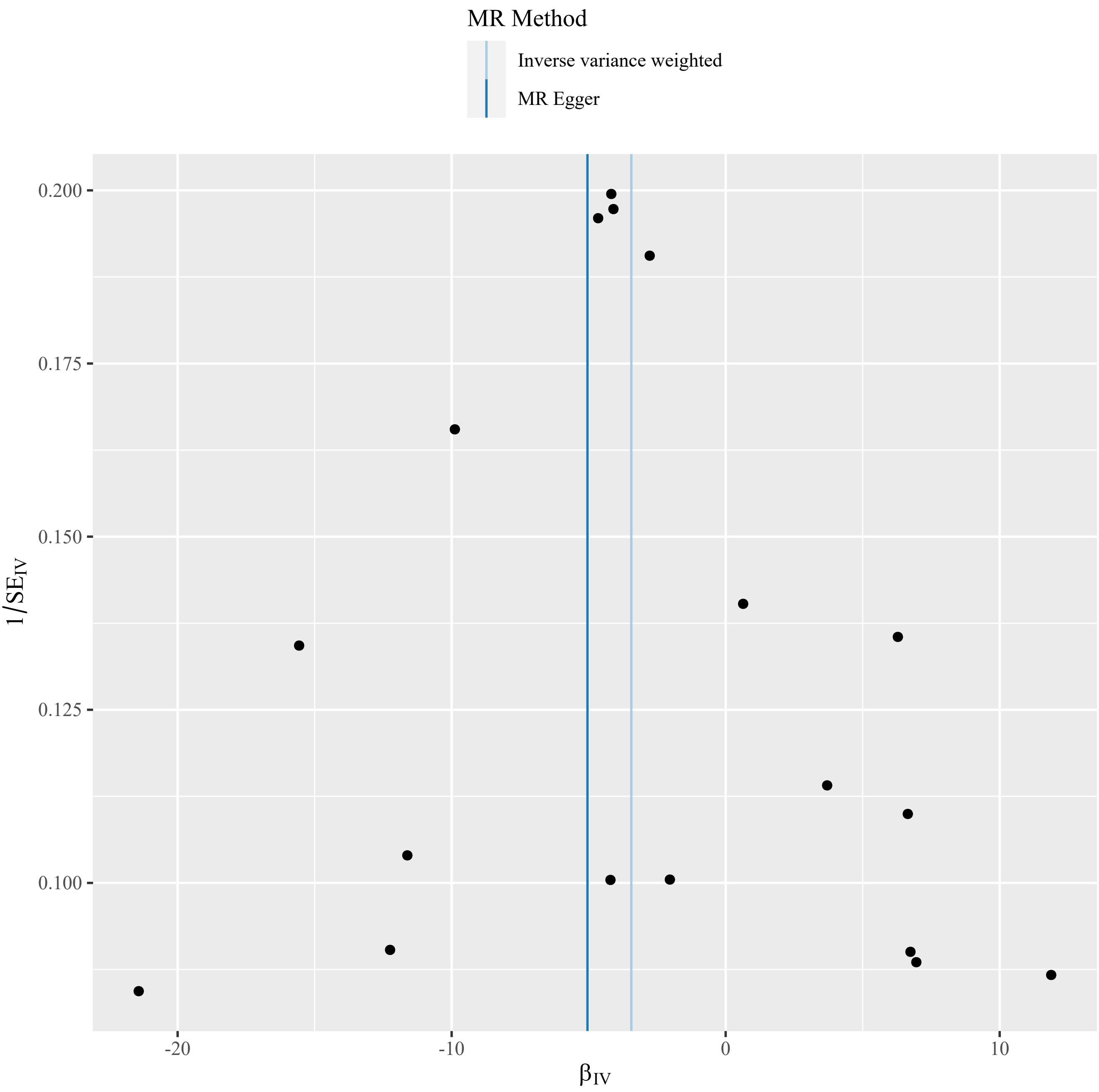
MR Method
Inverse variance weighted
MR Egger
0.200
0.175
0.150
0.125
0.100
$1/SE_{IV}$
-20
-10
0
10
β_{IV}

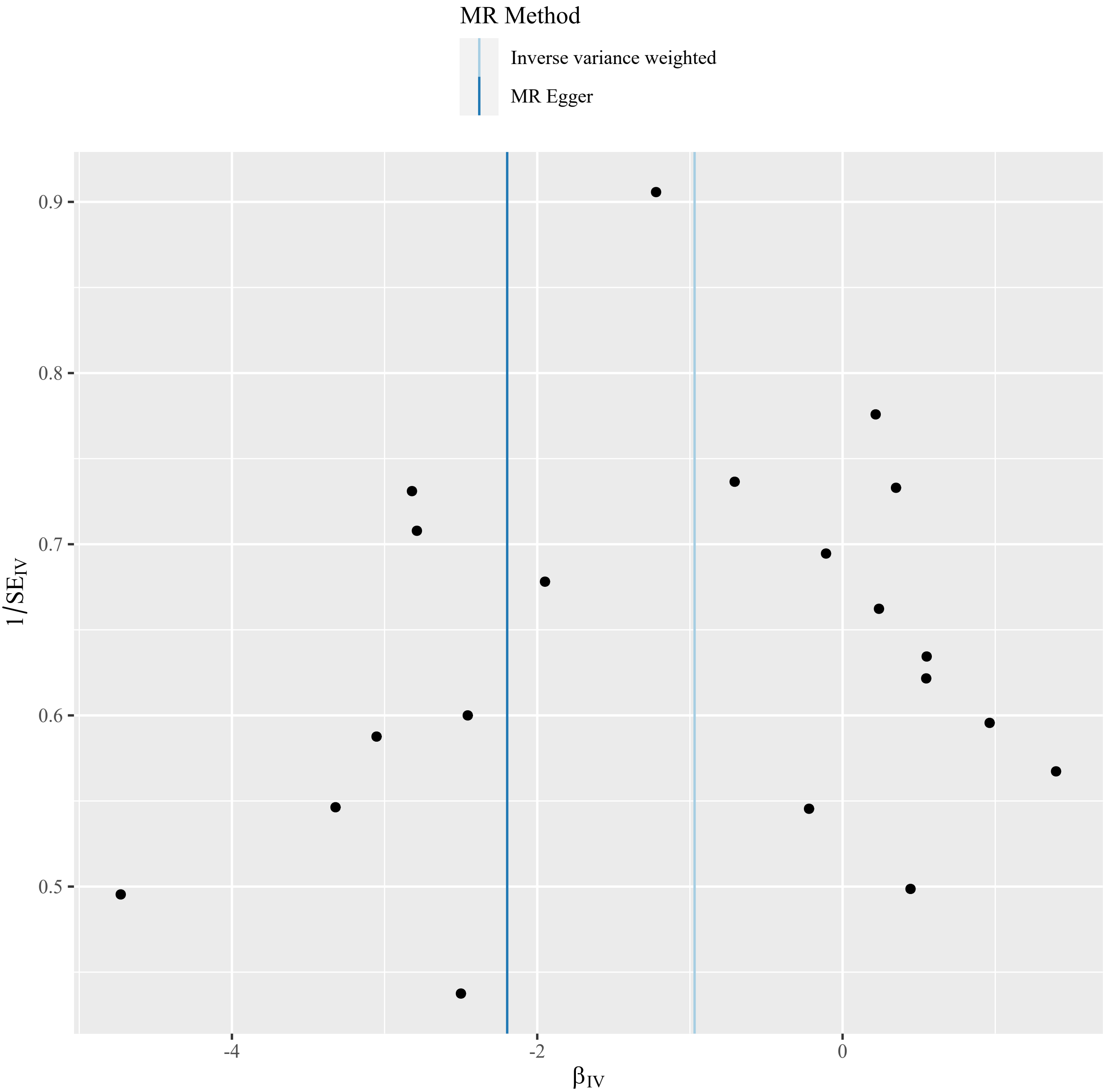

MR Method
Inverse variance weighted
MR Egger
0.9
0.8
0.7
0.6
0.5
$1/SE_{IV}$
-4
-2
0
β_{IV}

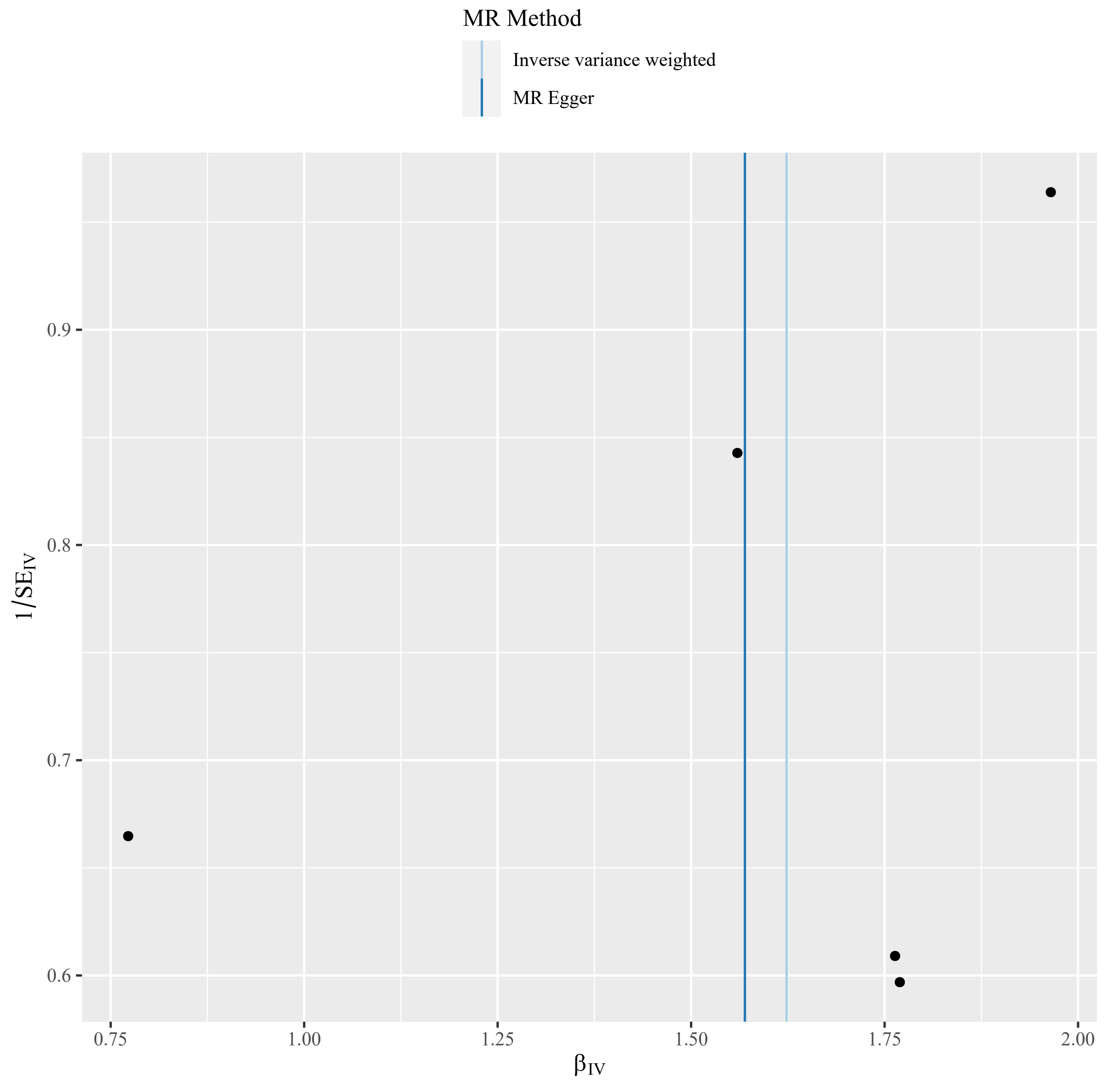

MR Method
Inverse variance weighted
MR Egger
0.9
0.8
0.7
0.6
1/SE_IV
0.75
1.00
1.25
1.50
1.75
2.00
β_IV

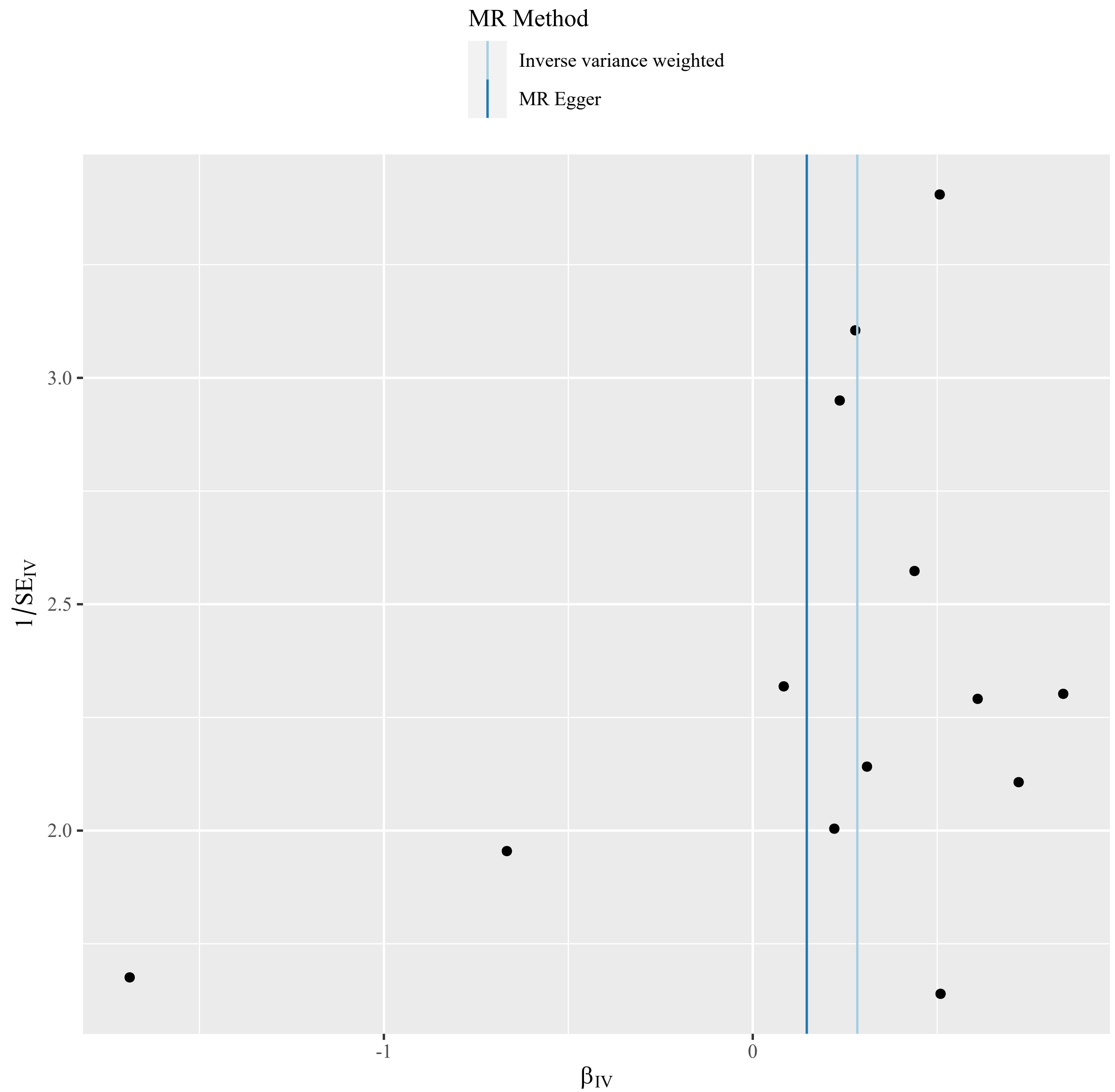
MR Method
Inverse variance weighted
MR Egger
3.0
2.5
2.0
$1/SE_{IV}$
-1
0
β_{IV}

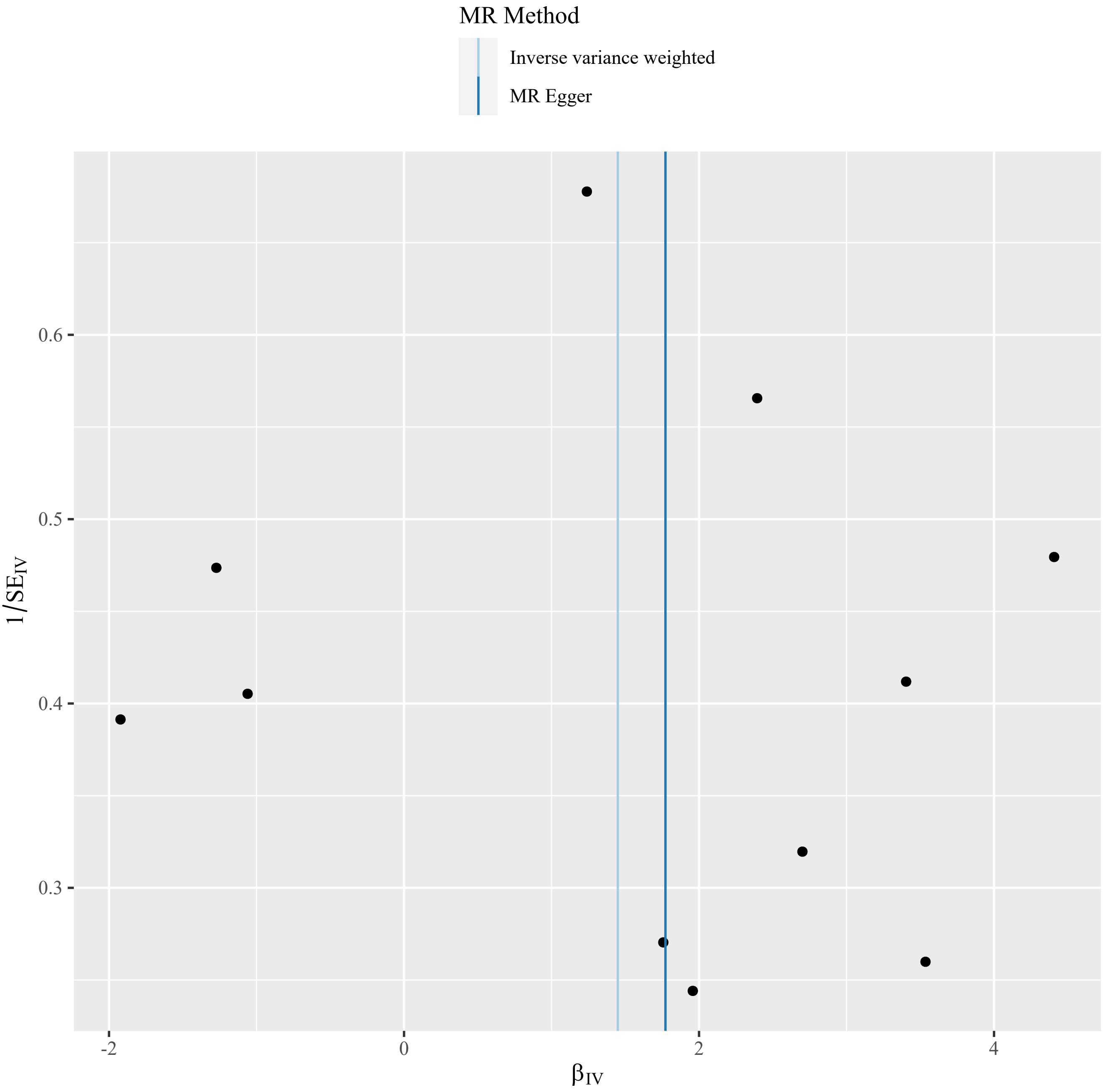

MR Method
Inverse variance weighted
MR Egger
0.6
0.5
0.4
0.3
$1/SE_{IV}$
-2
0
2
4
β_{IV}
