# Supplementary figures and images for: Investigating the metabolomic pathways in female reproductive endocrine disorders: a Mendelian randomization study
Source: Front Endocrinol (Lausanne). 2024 Oct 31;15:1438079. doi: 10.3389/fendo.2024.1438079 (PMC11560792; doi:10.3389/fendo.2024.1438079)

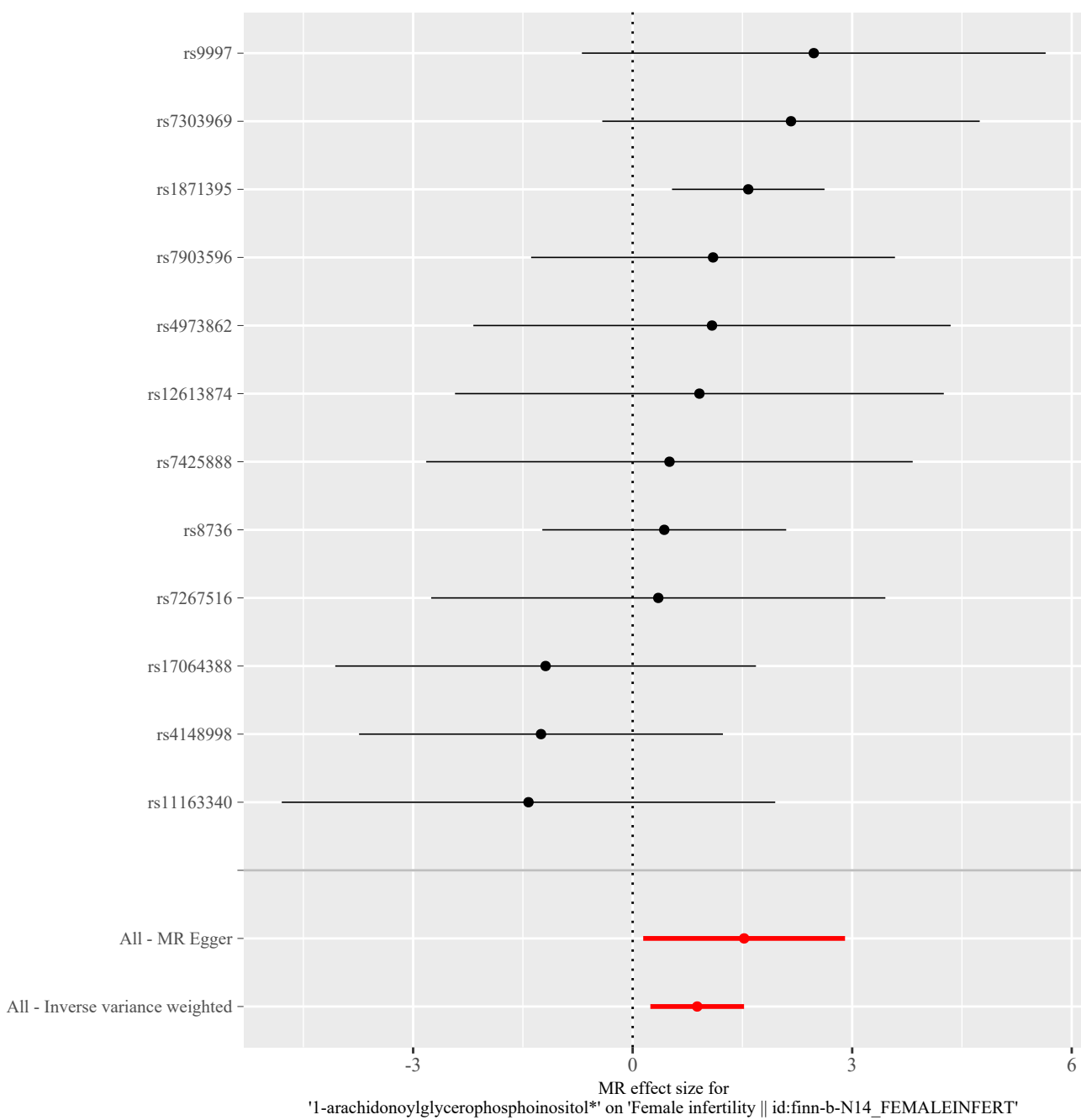

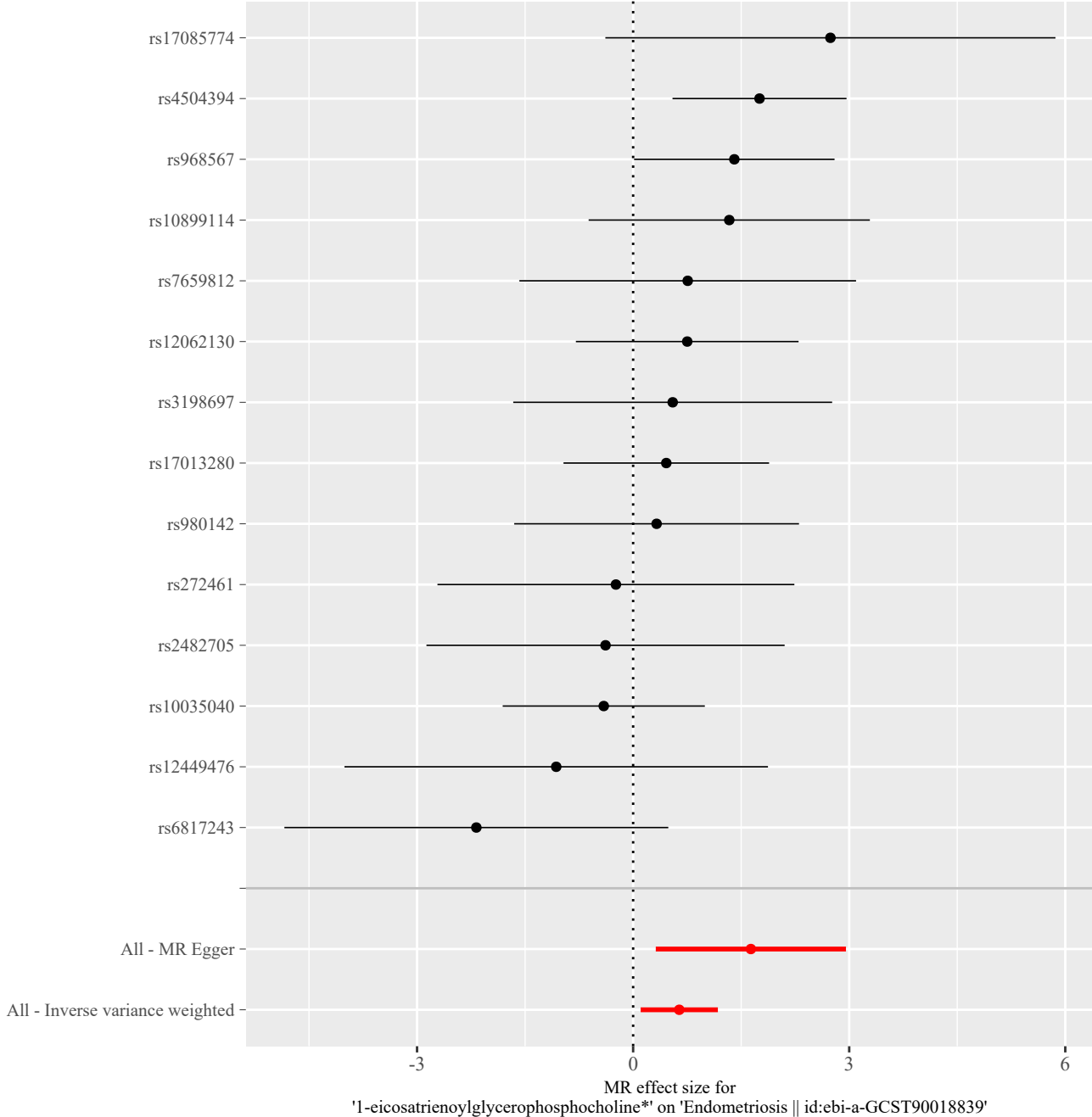

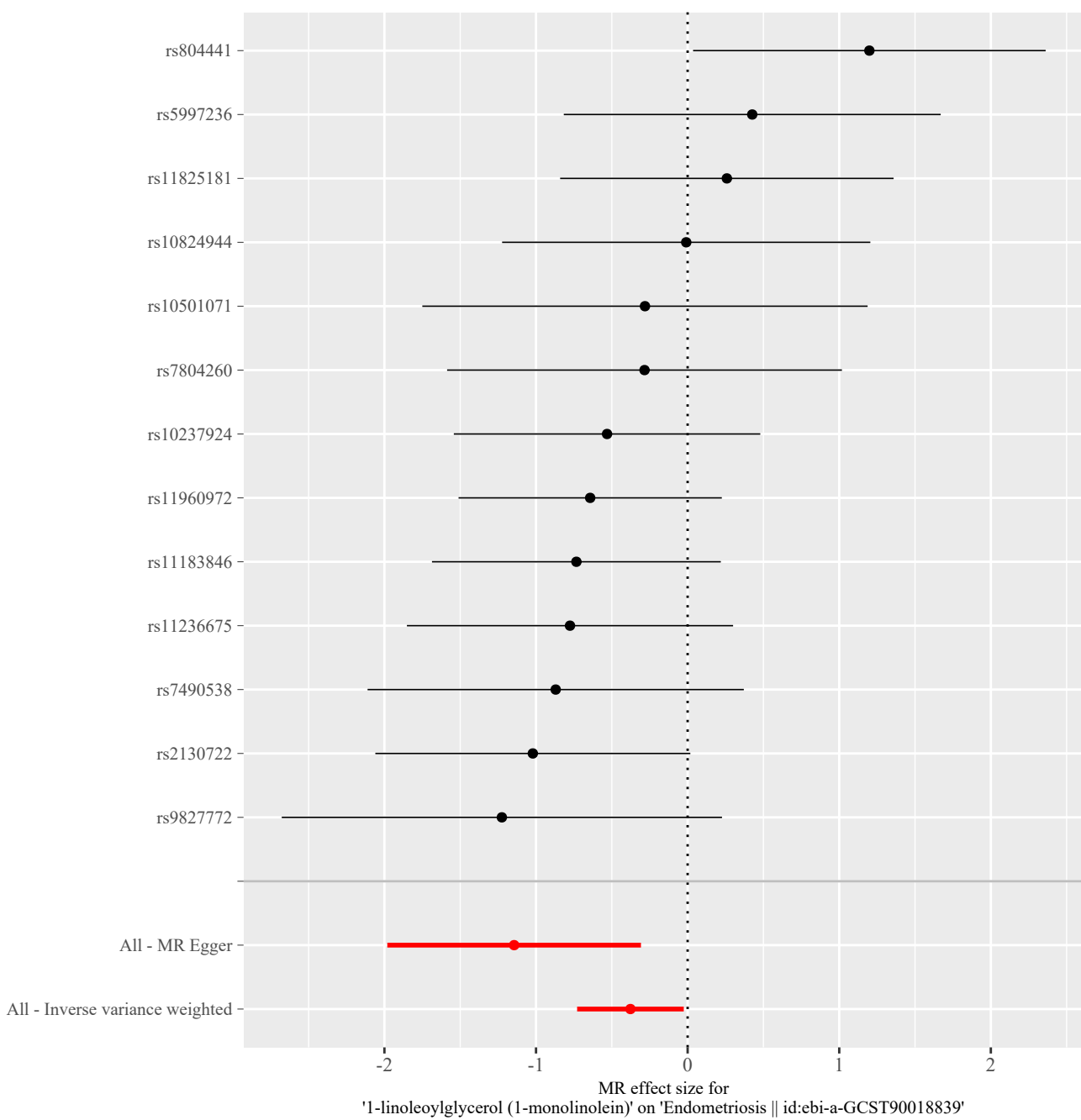

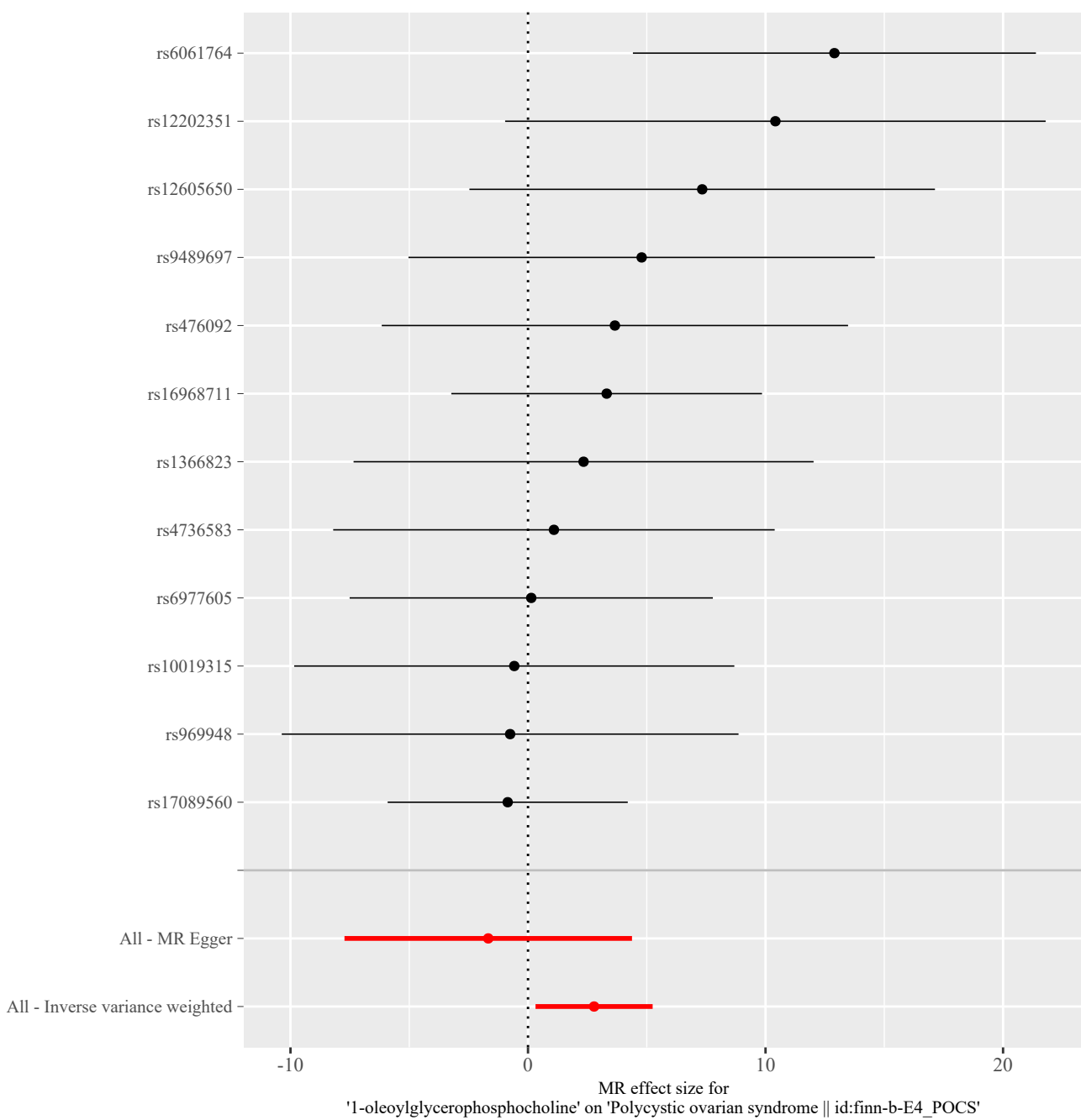

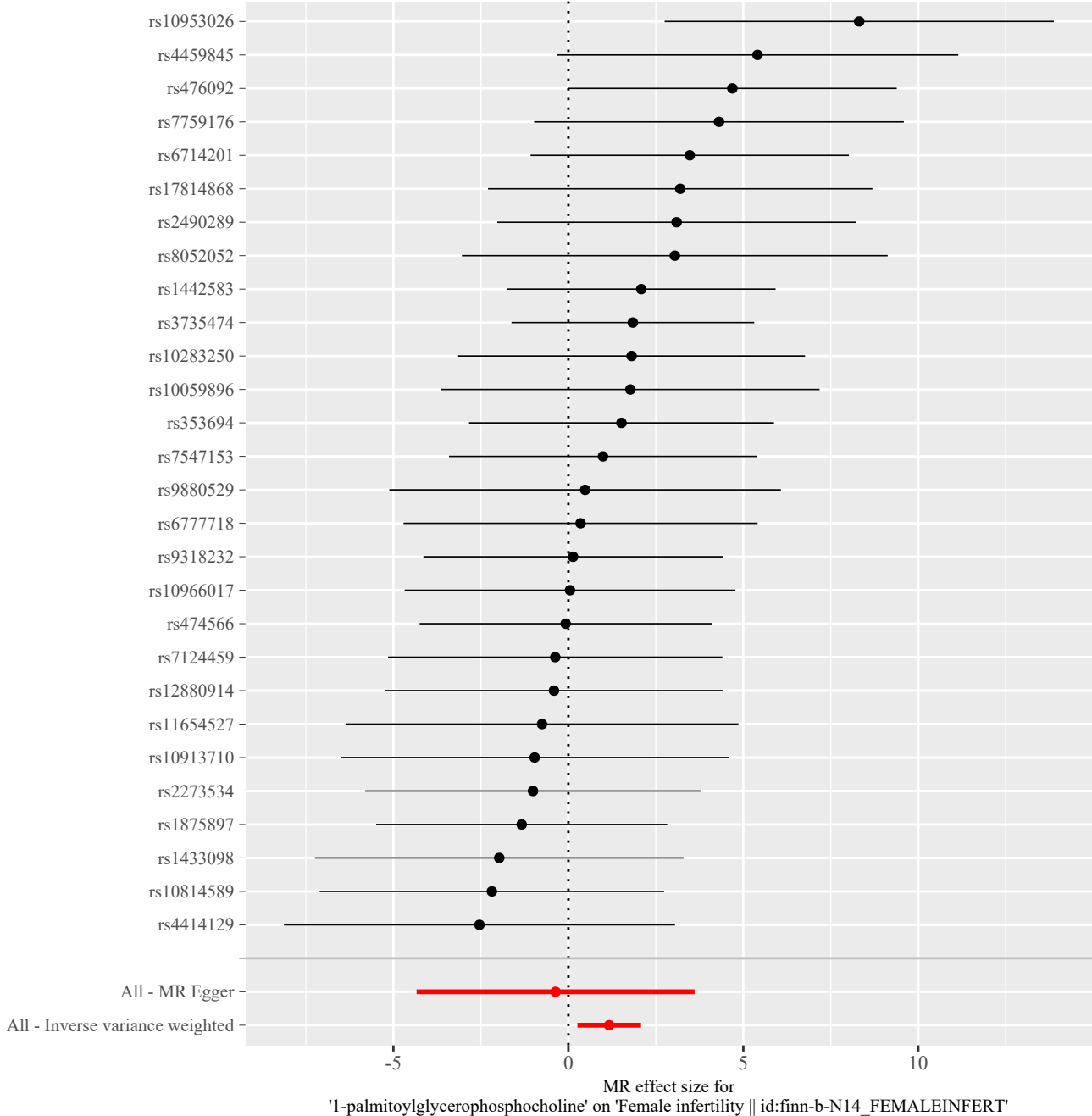

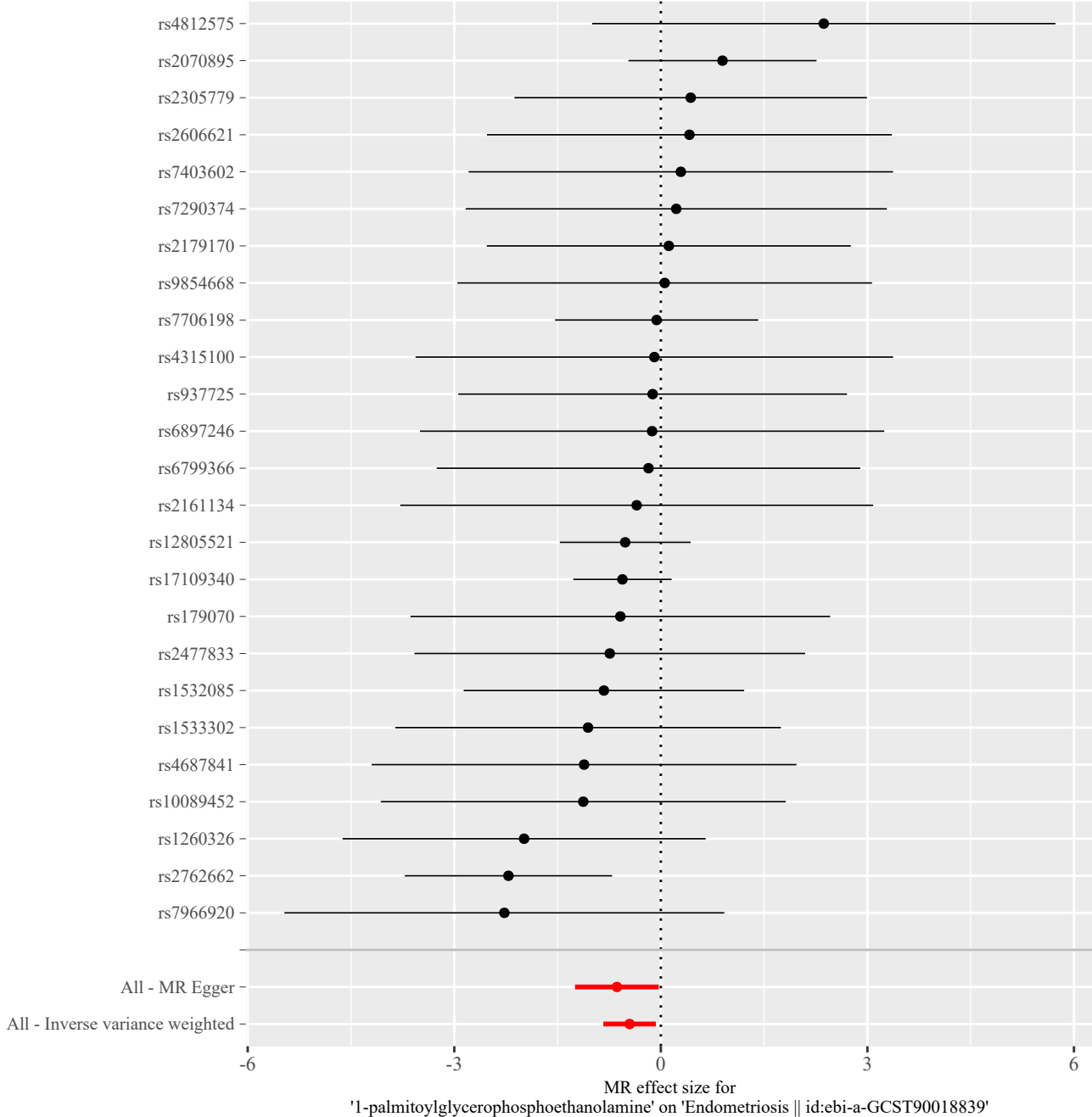

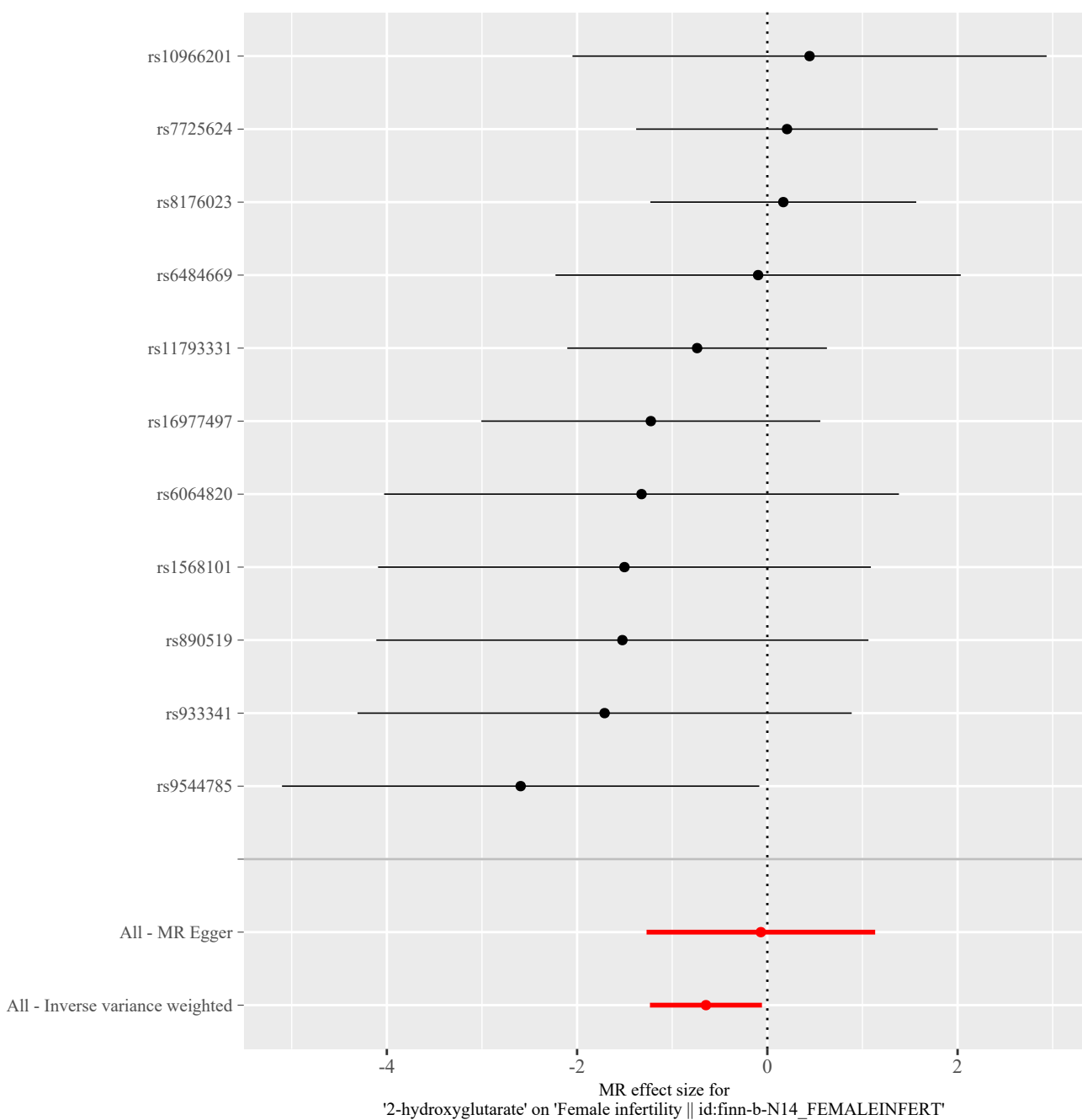

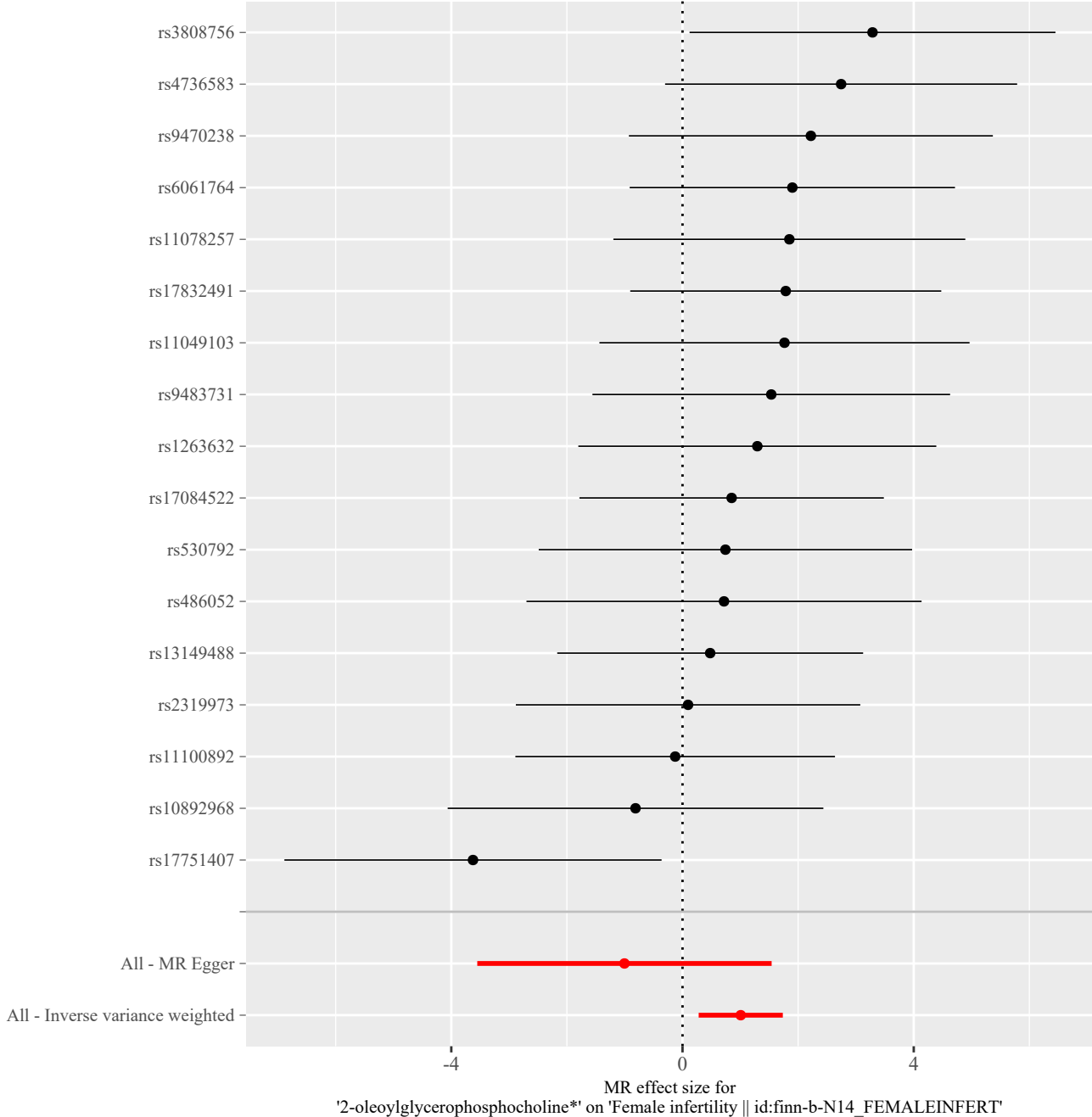

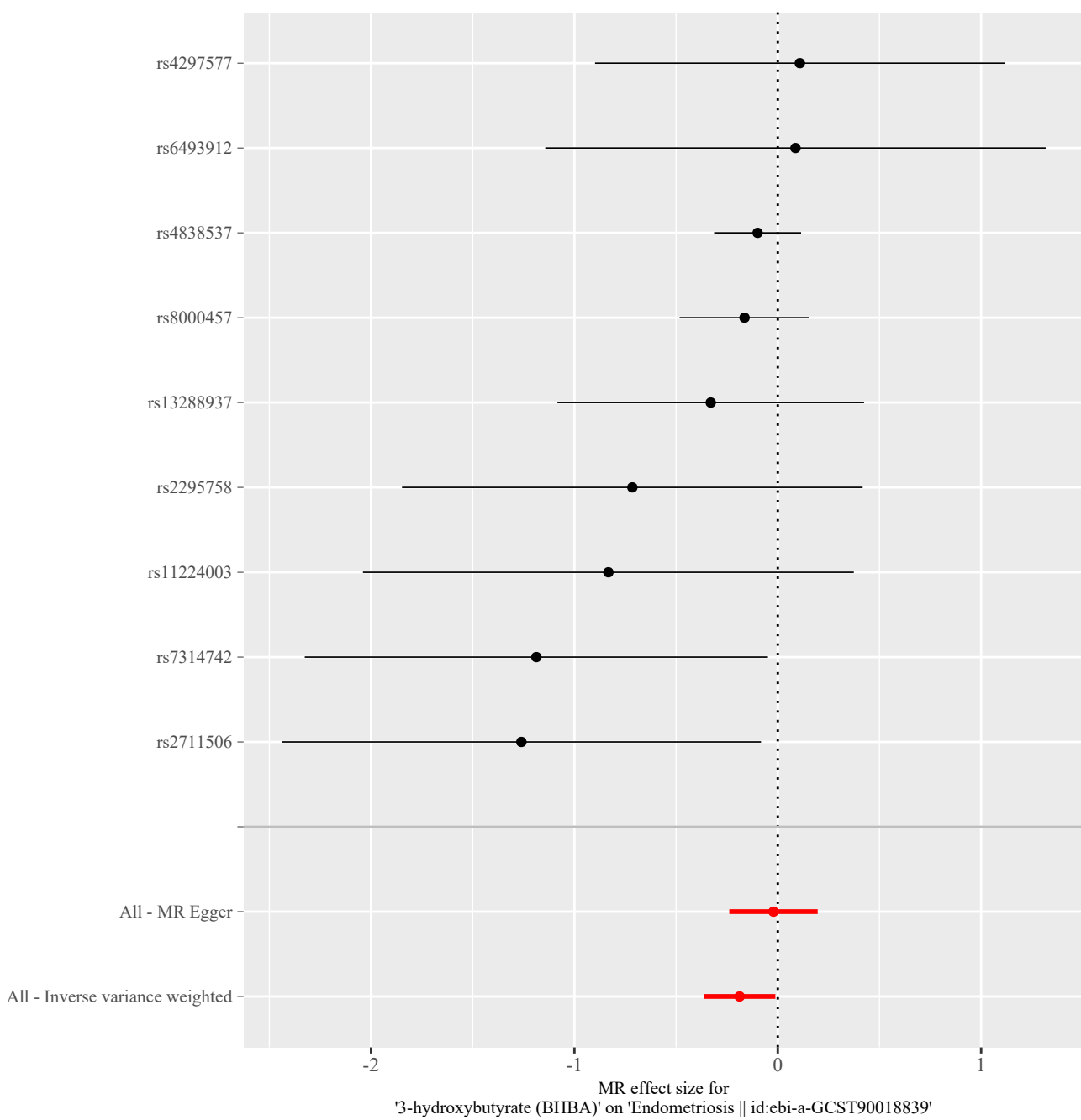

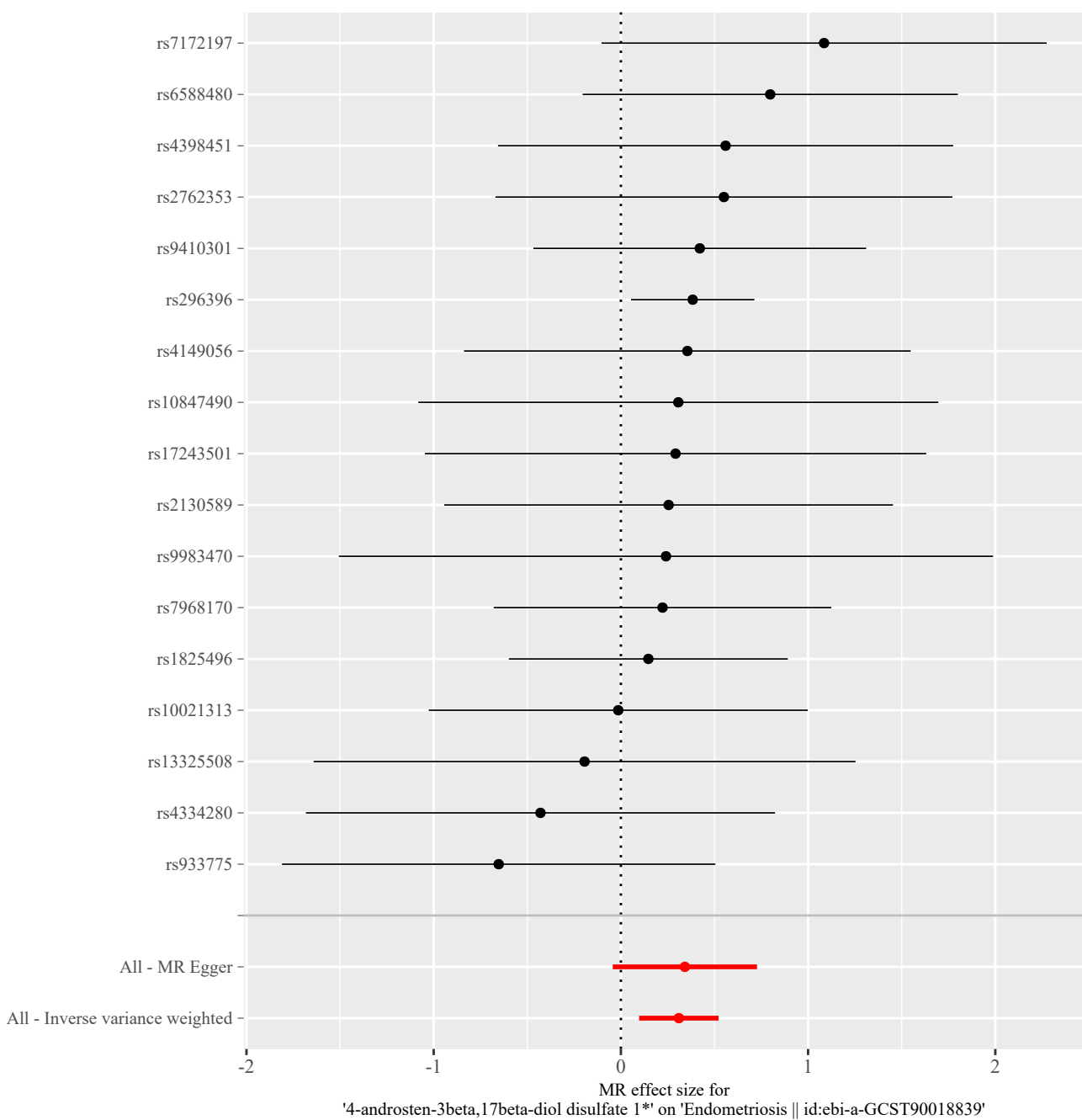

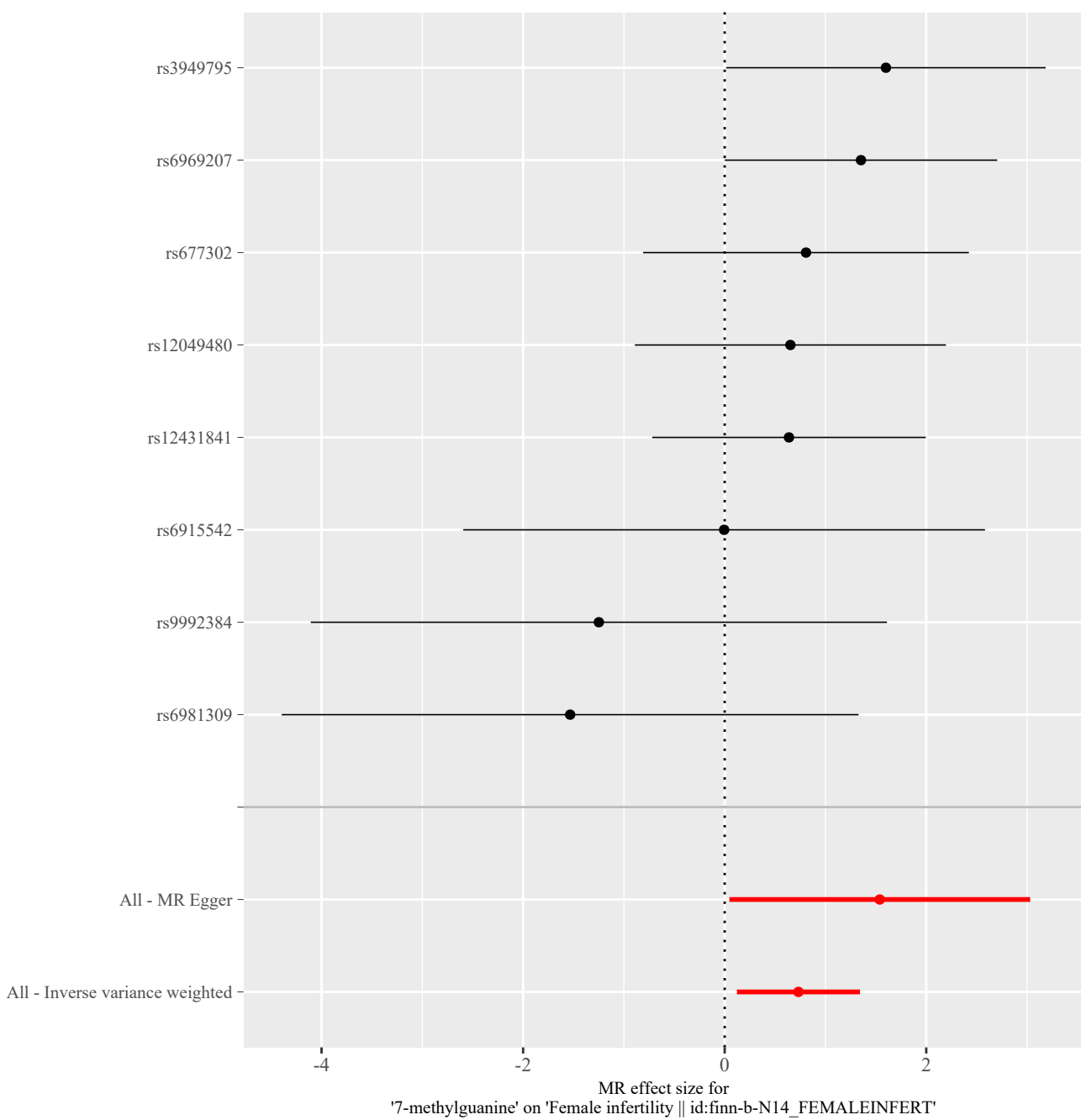

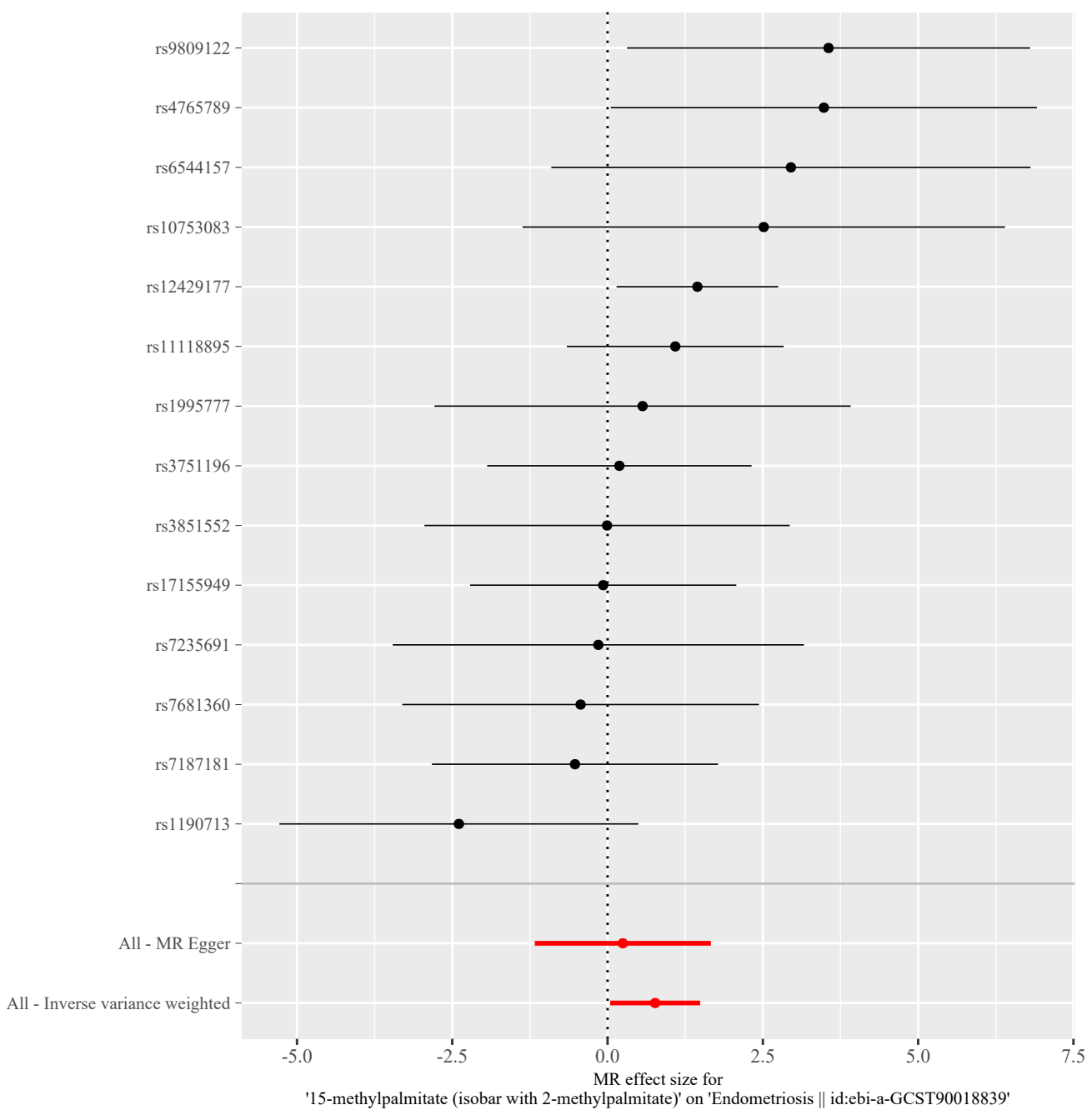

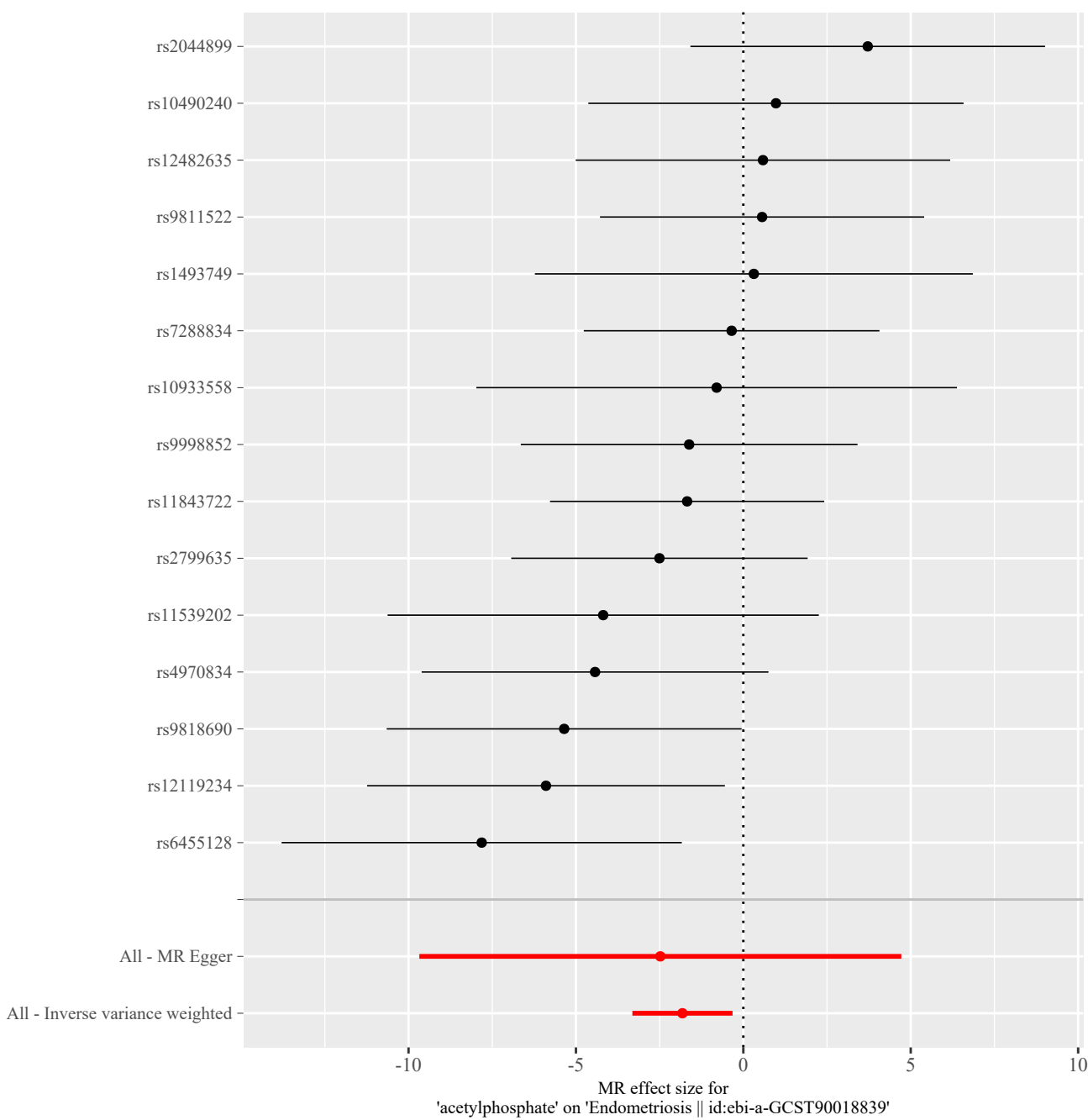

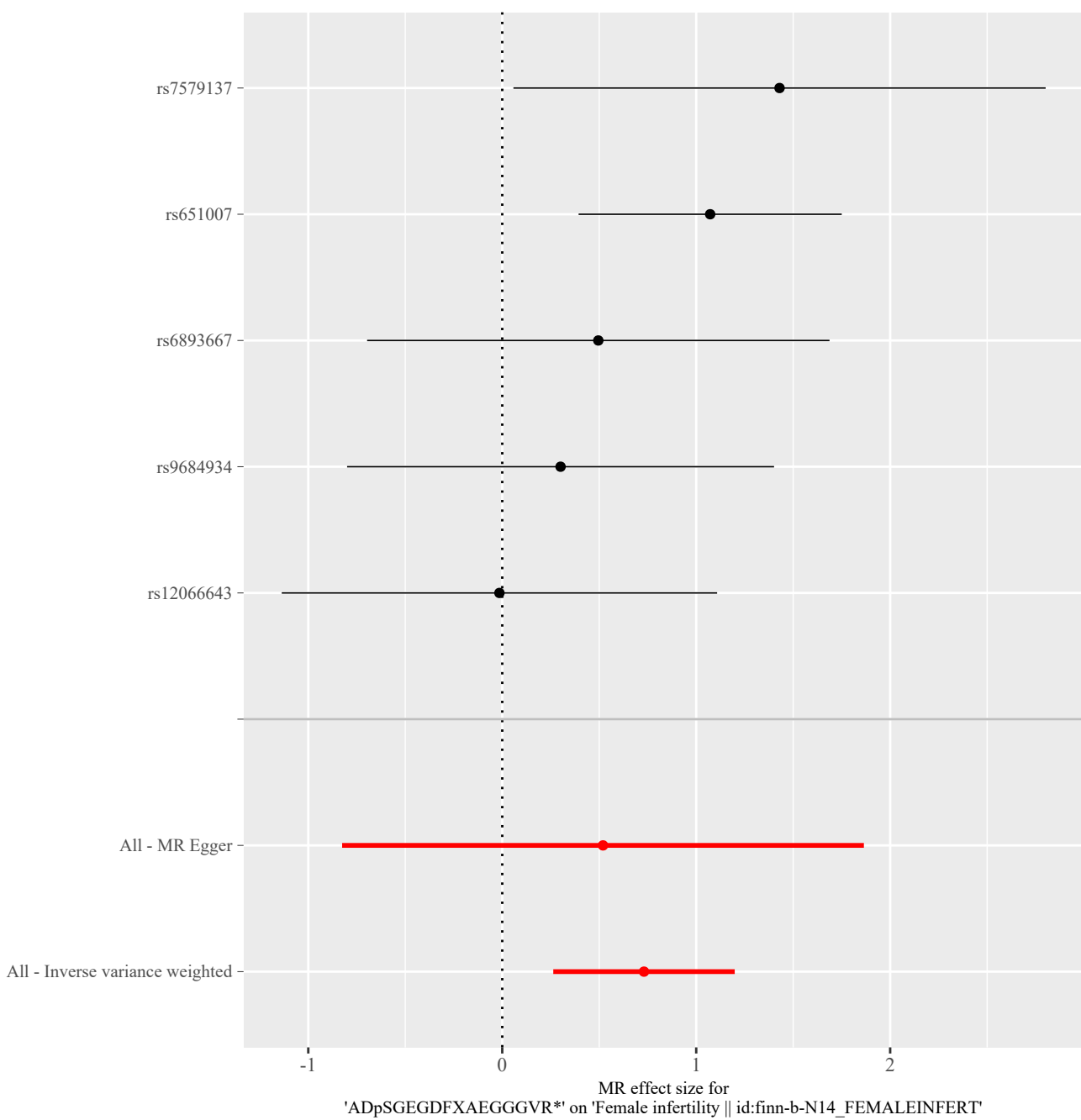

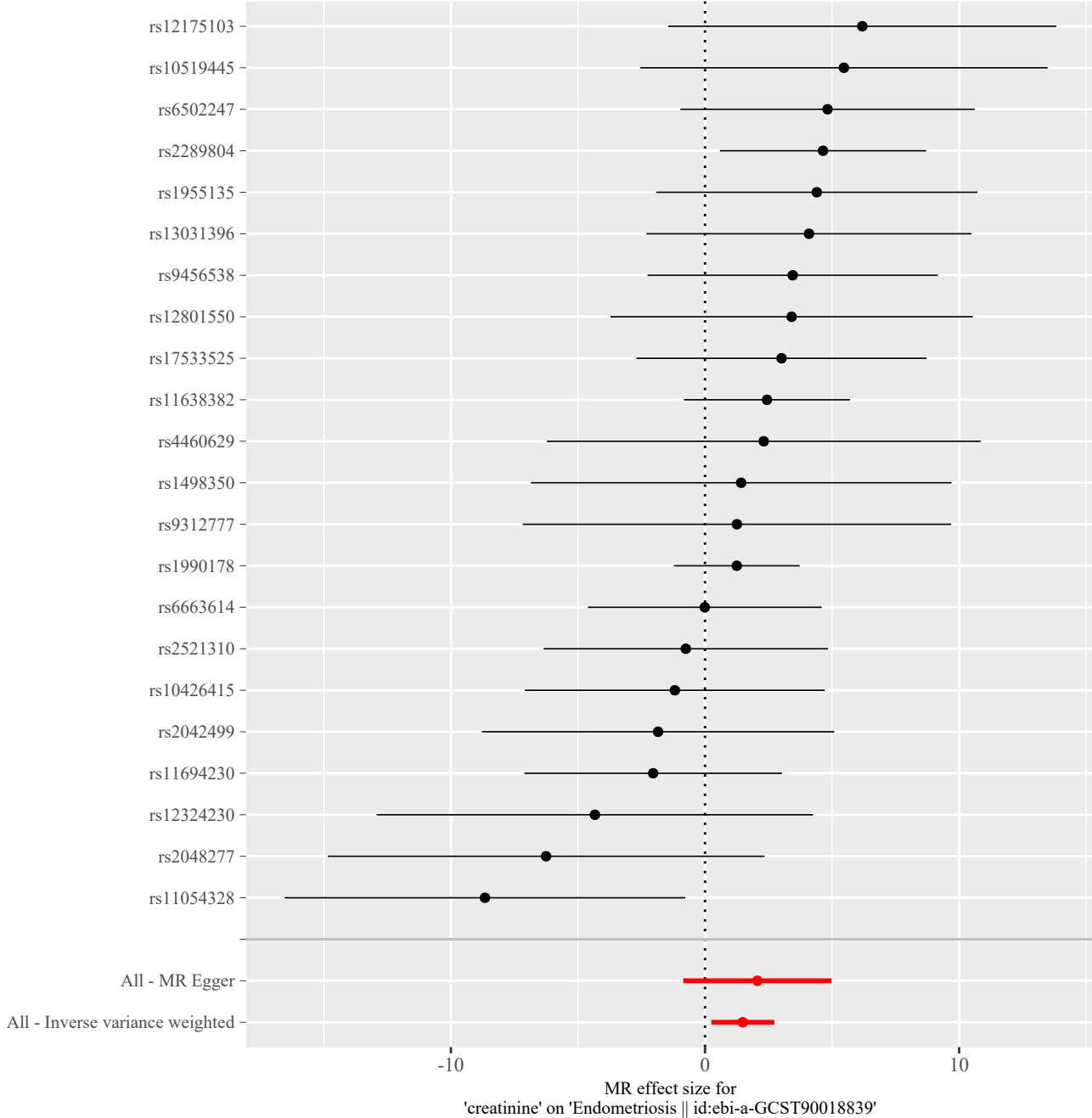

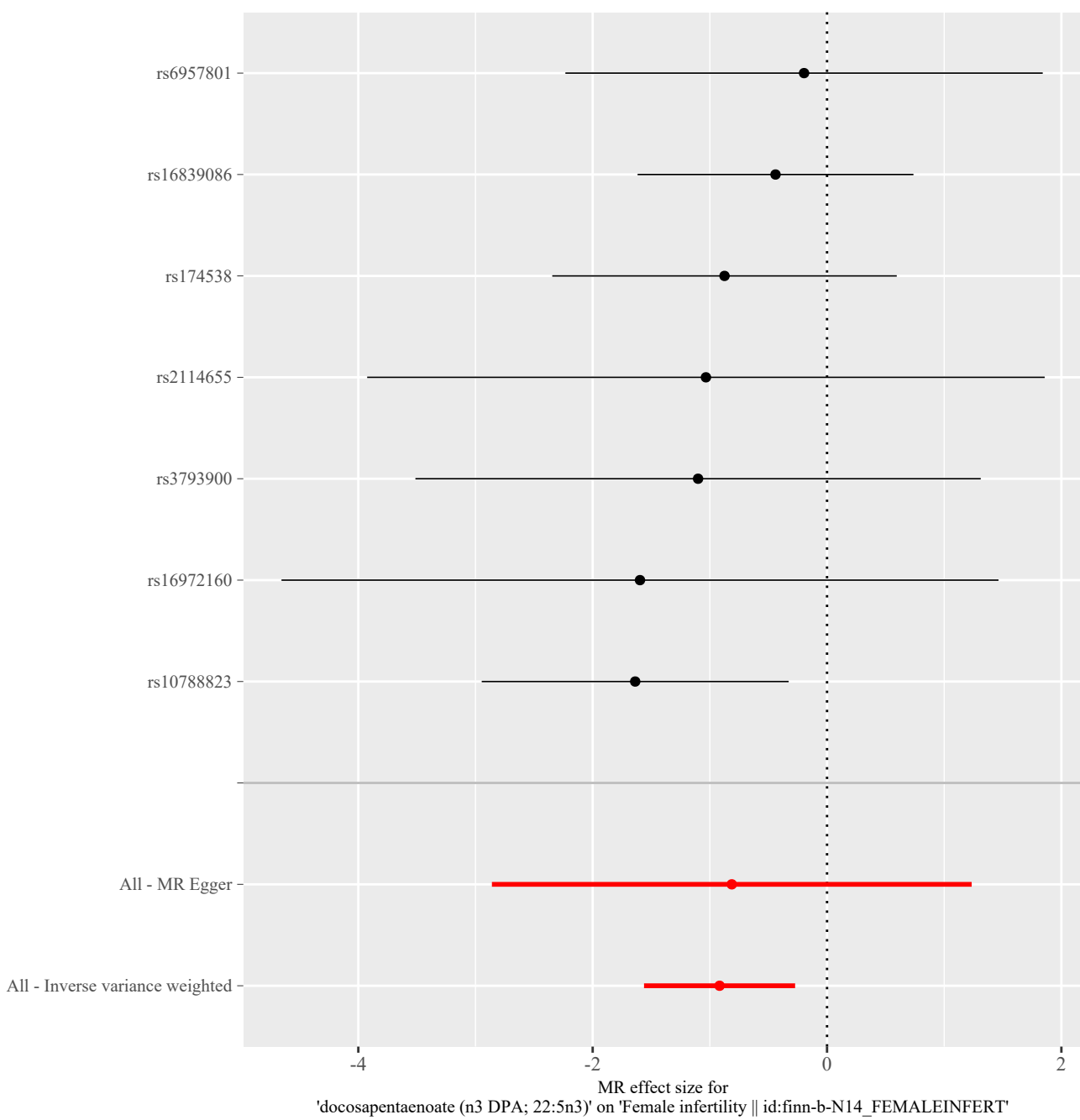

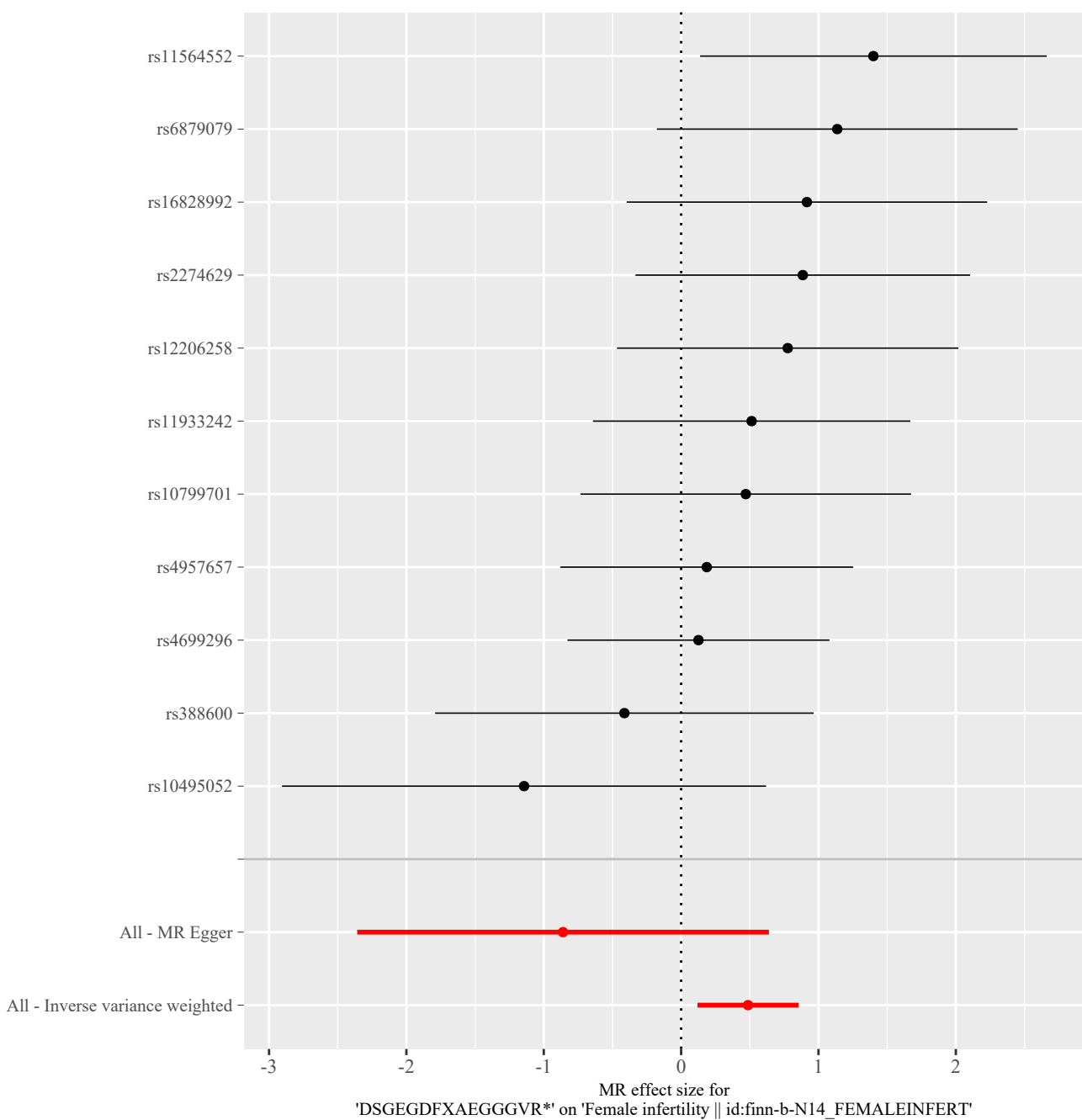

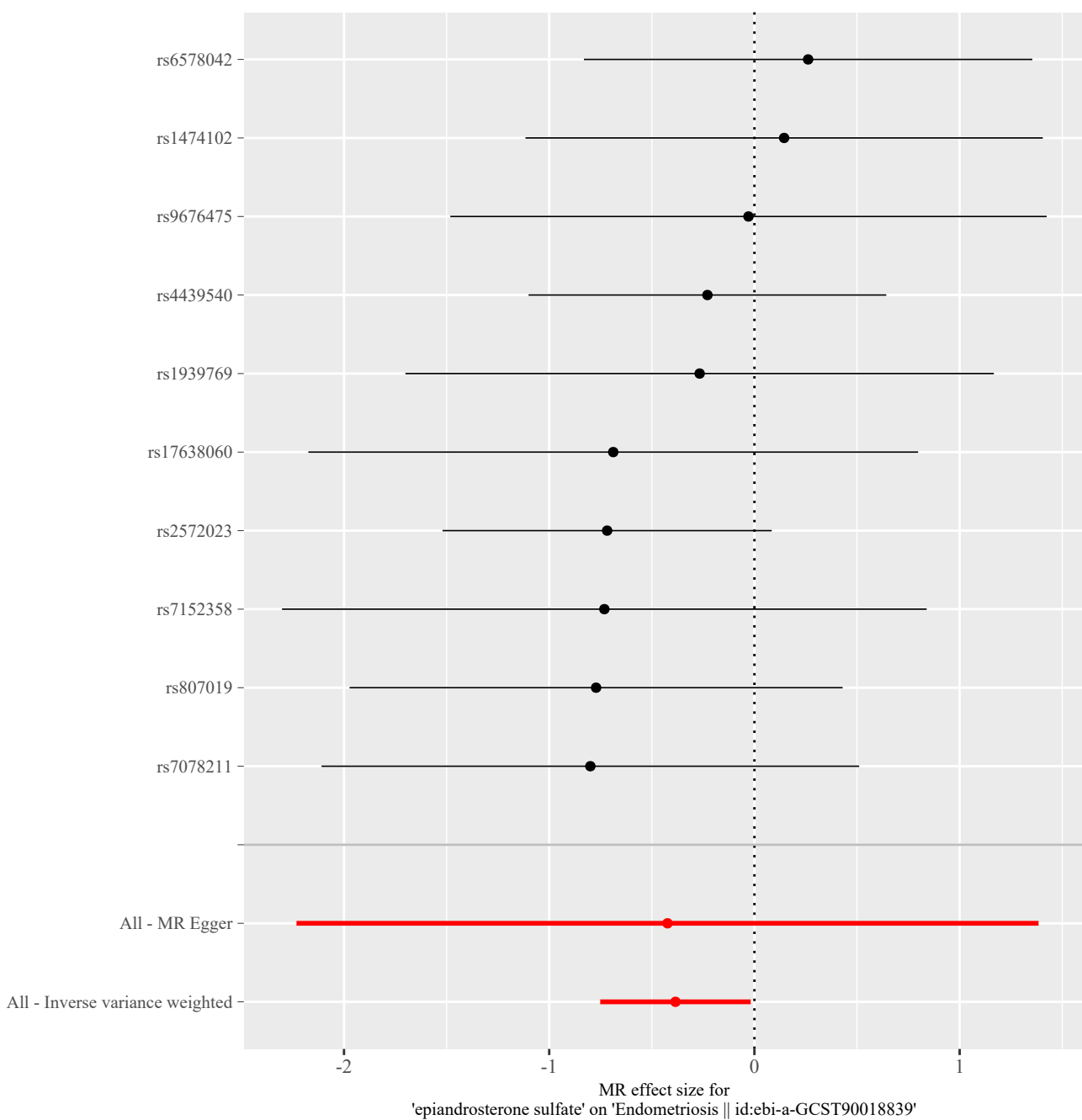

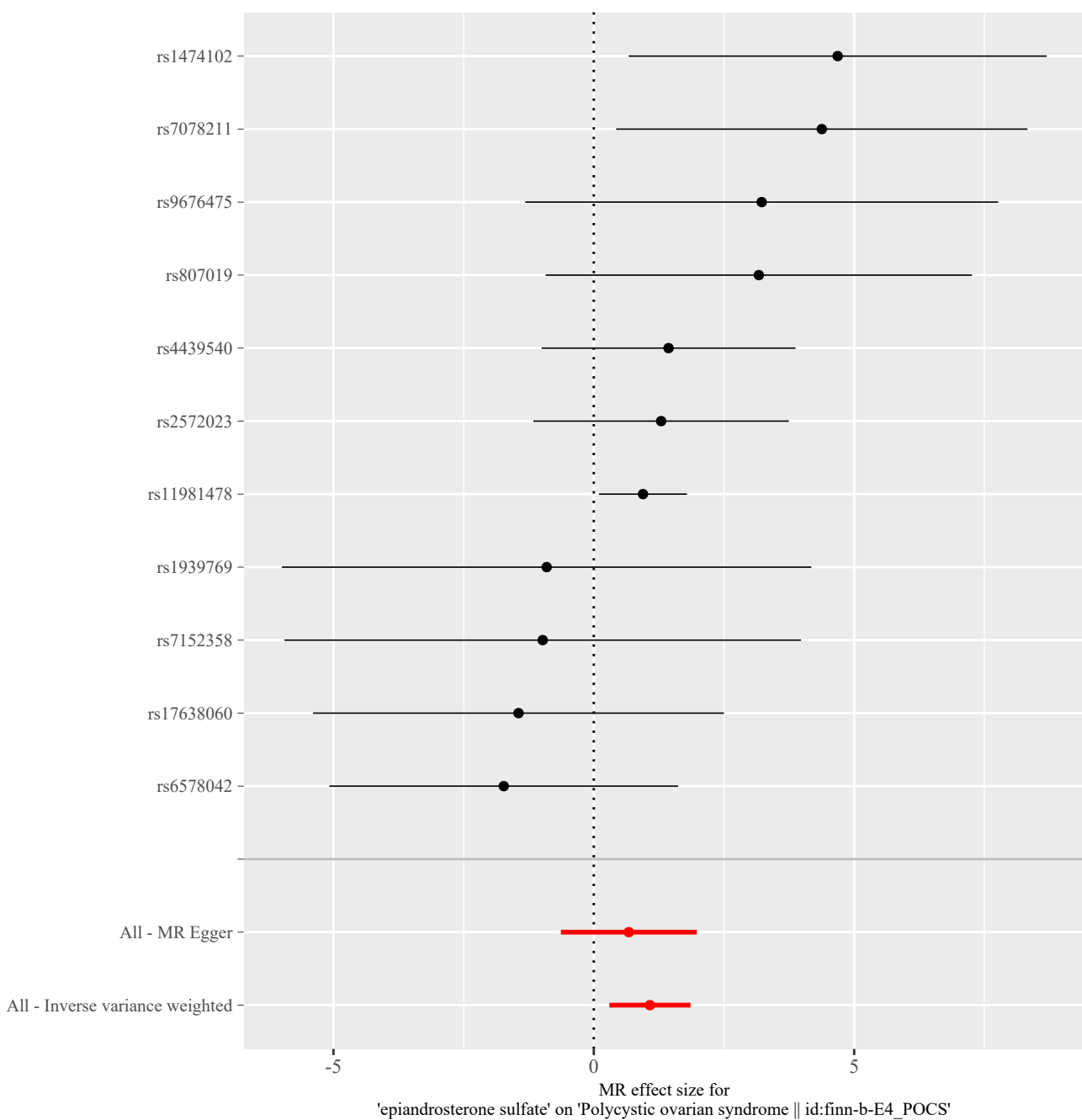

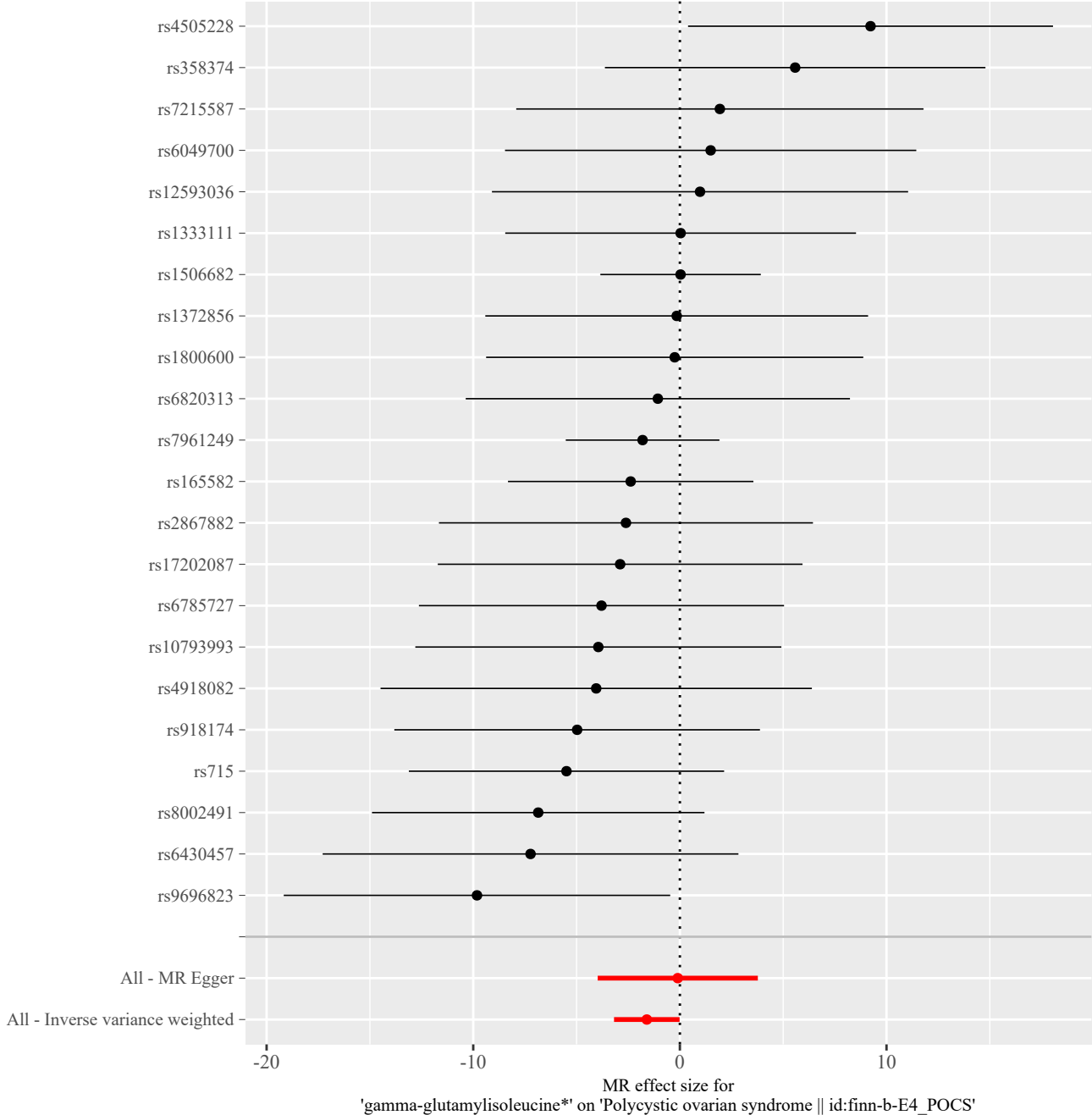

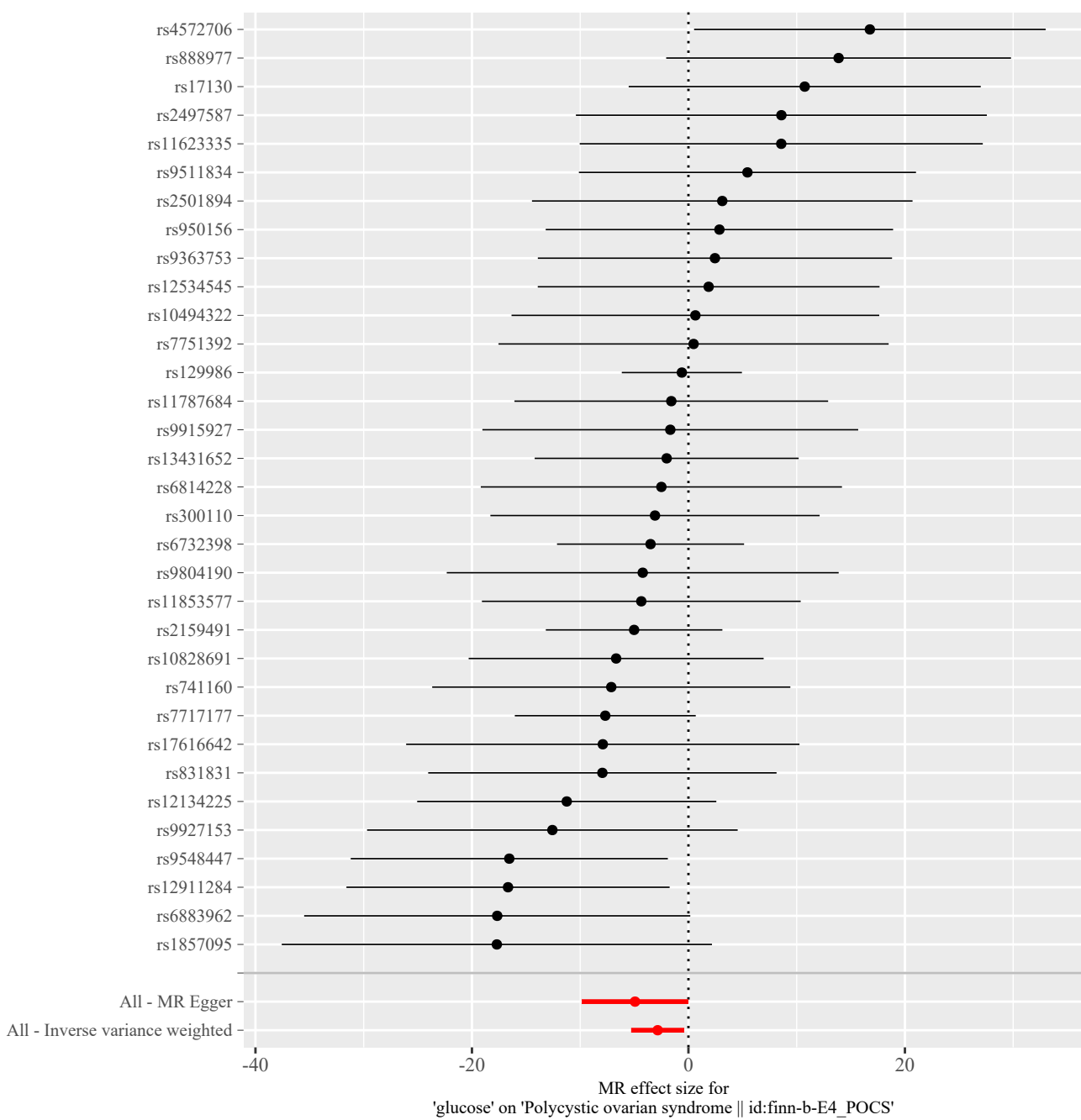

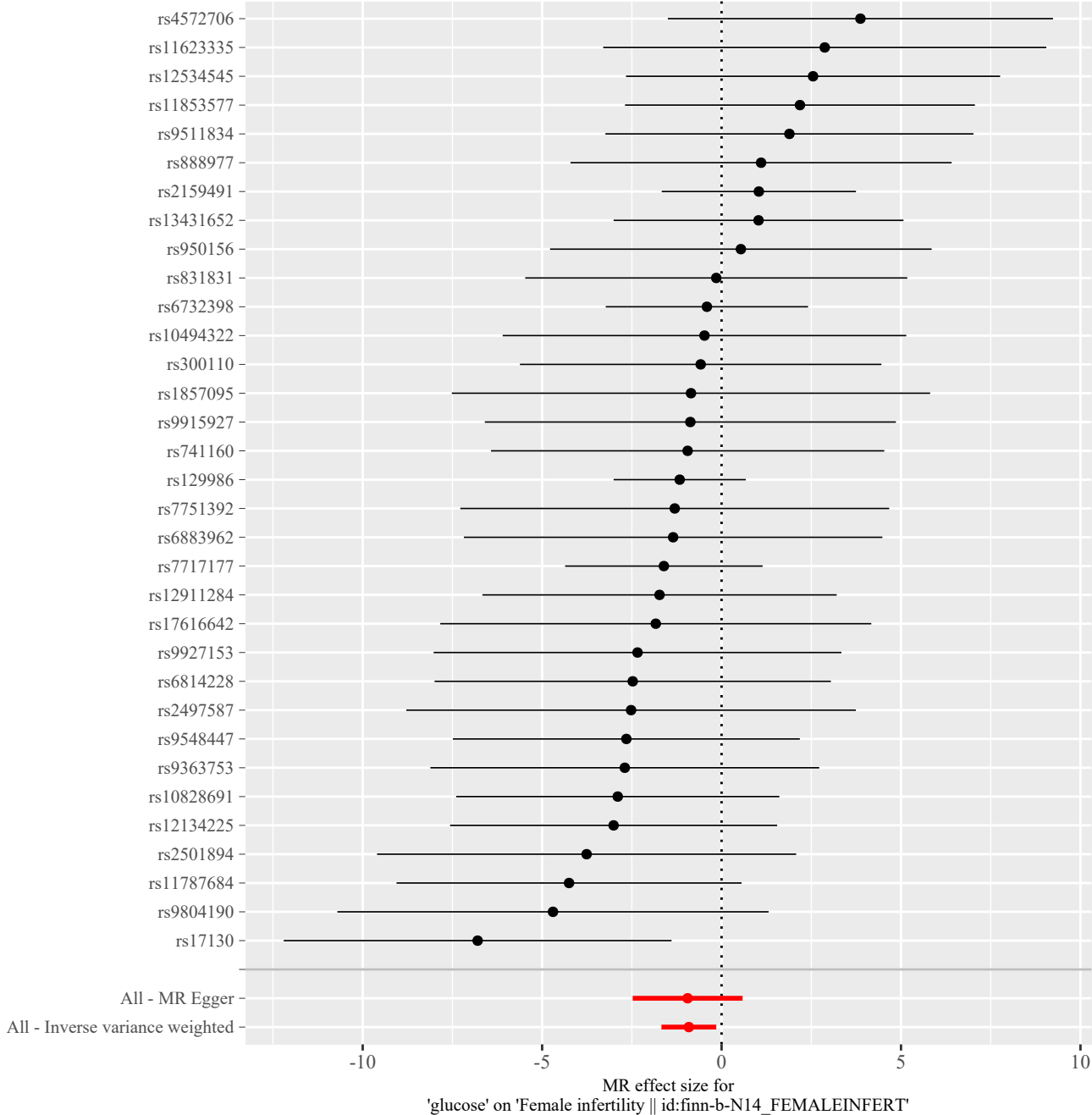

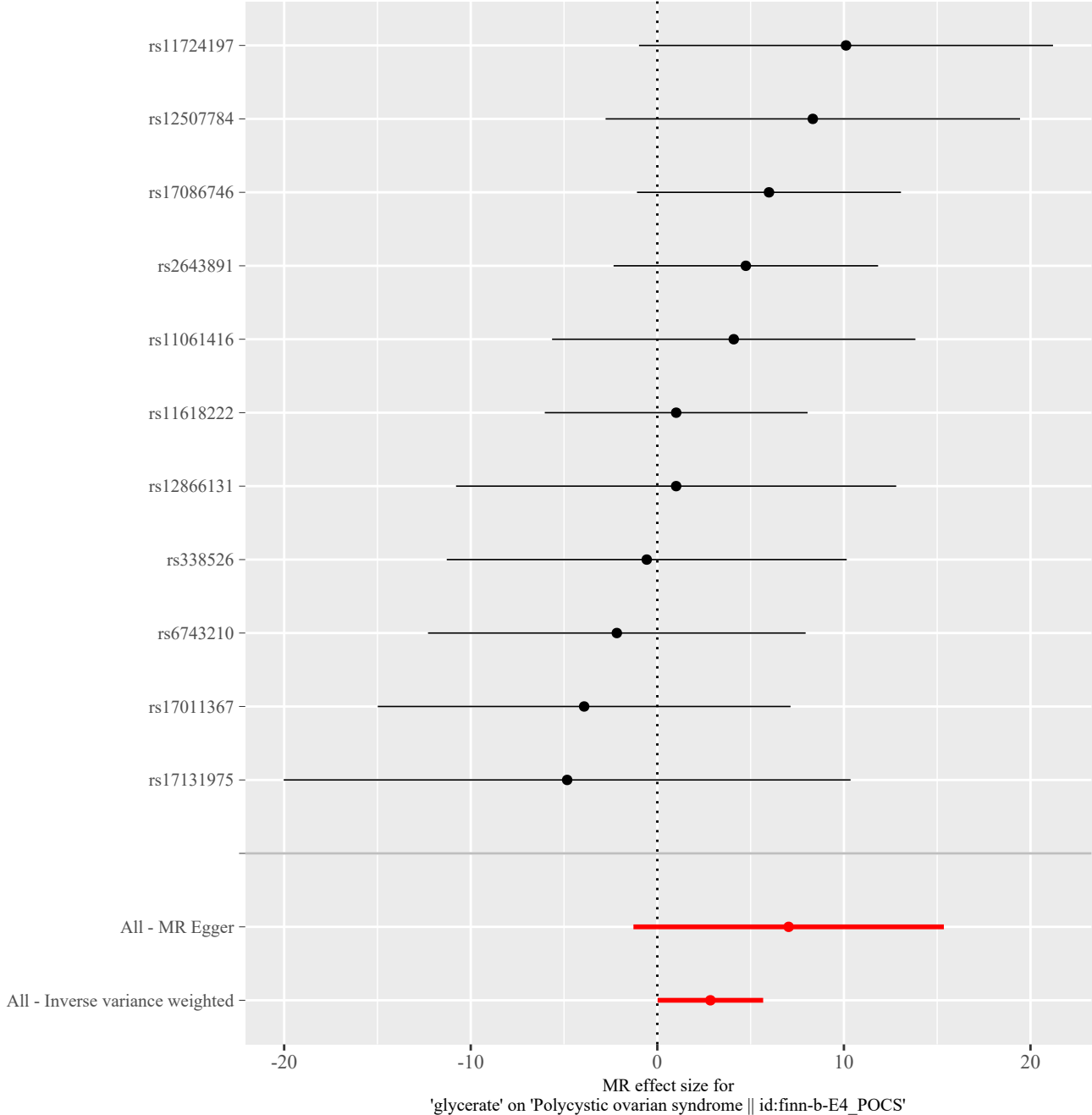

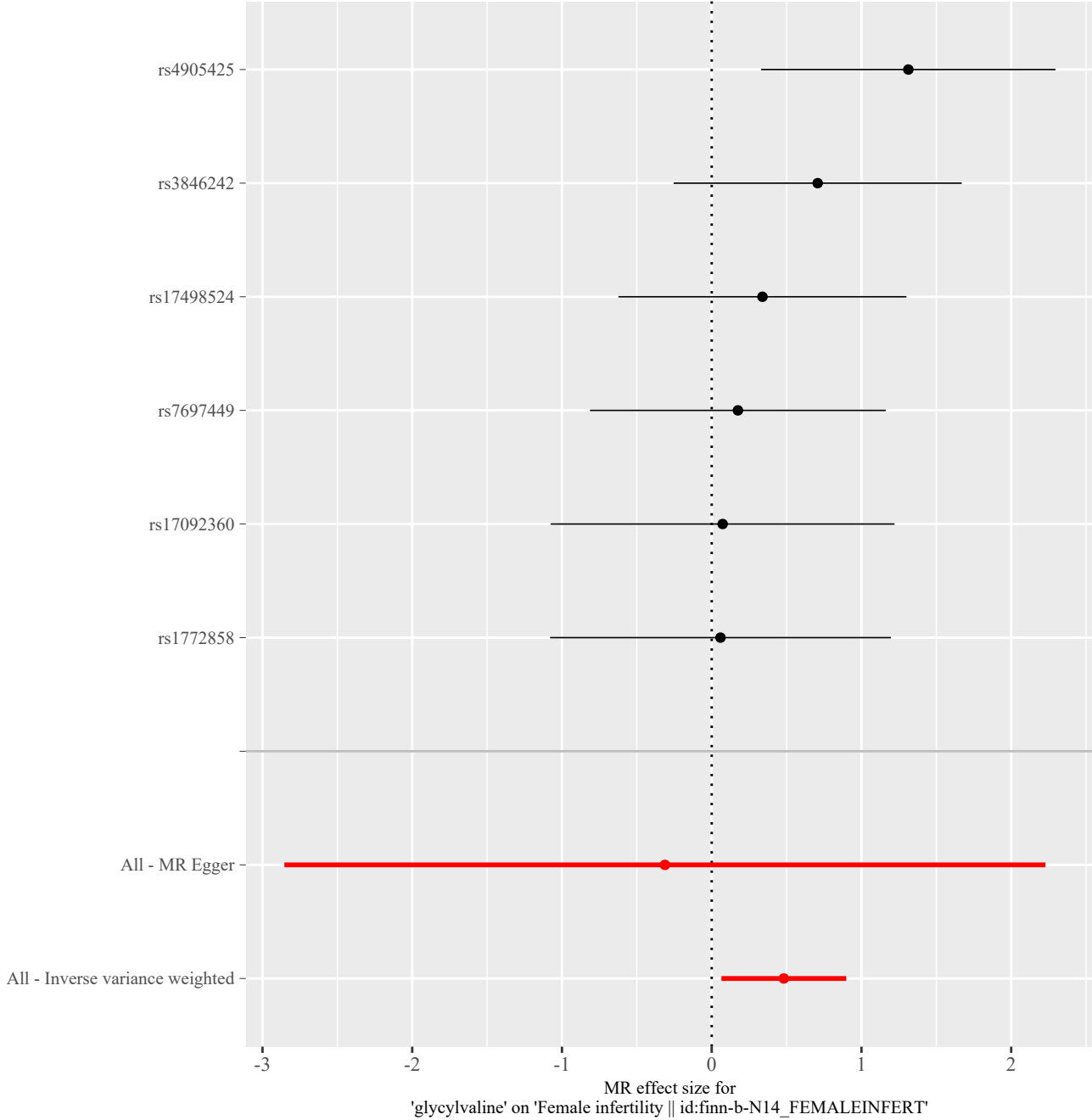

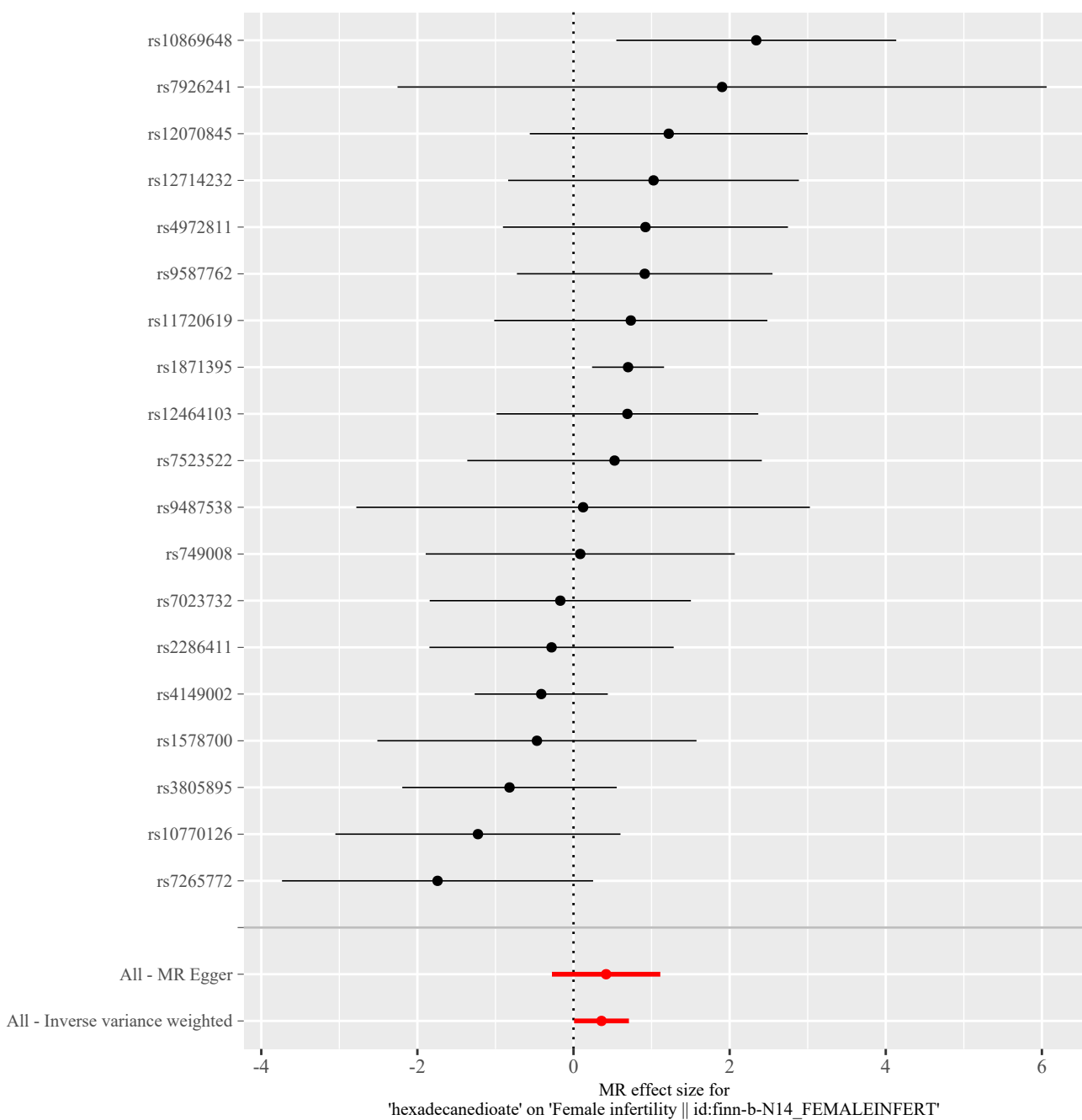

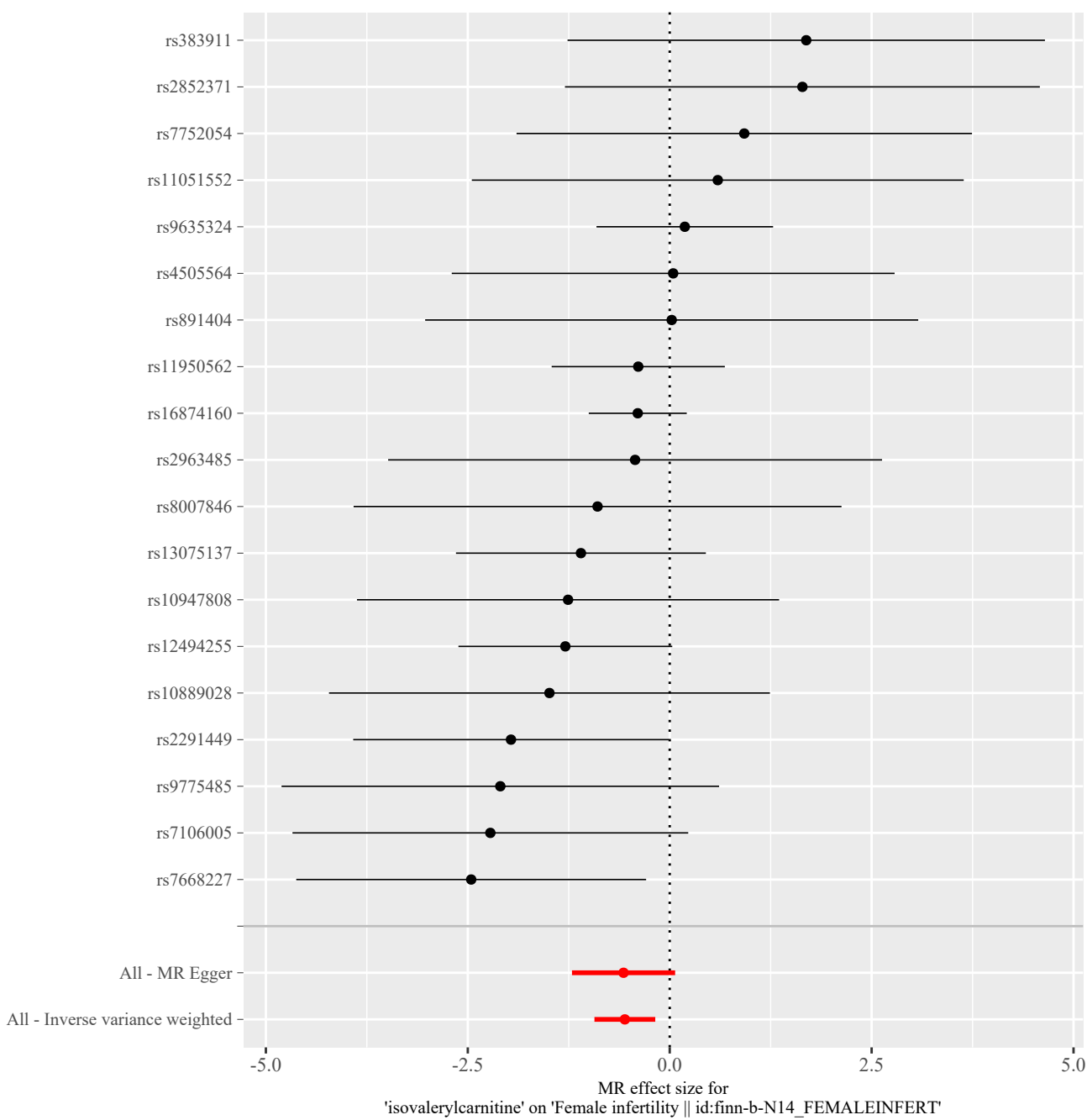

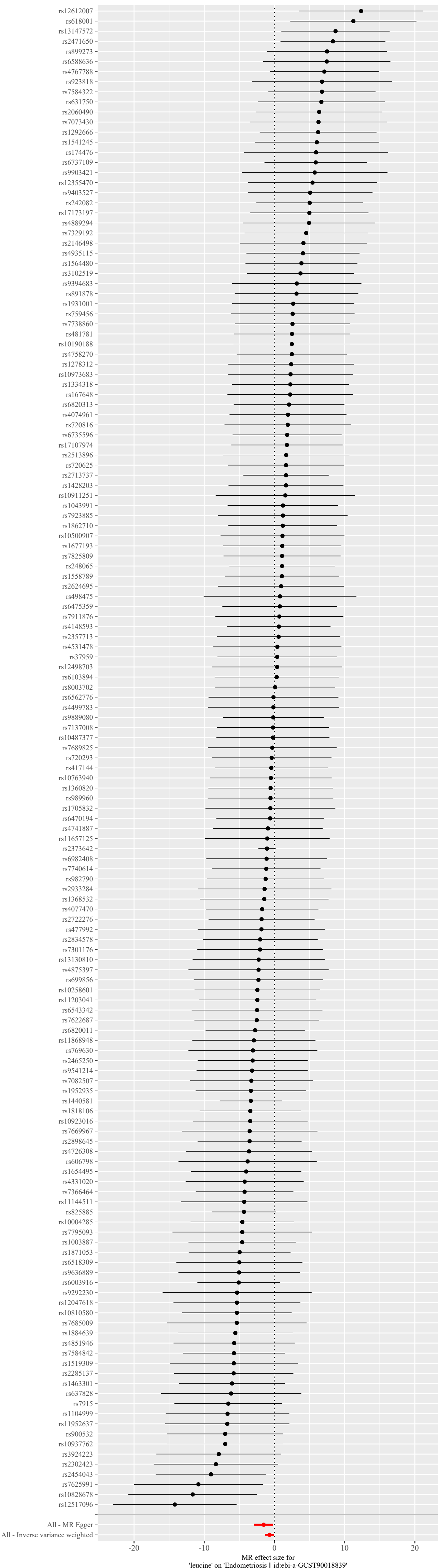

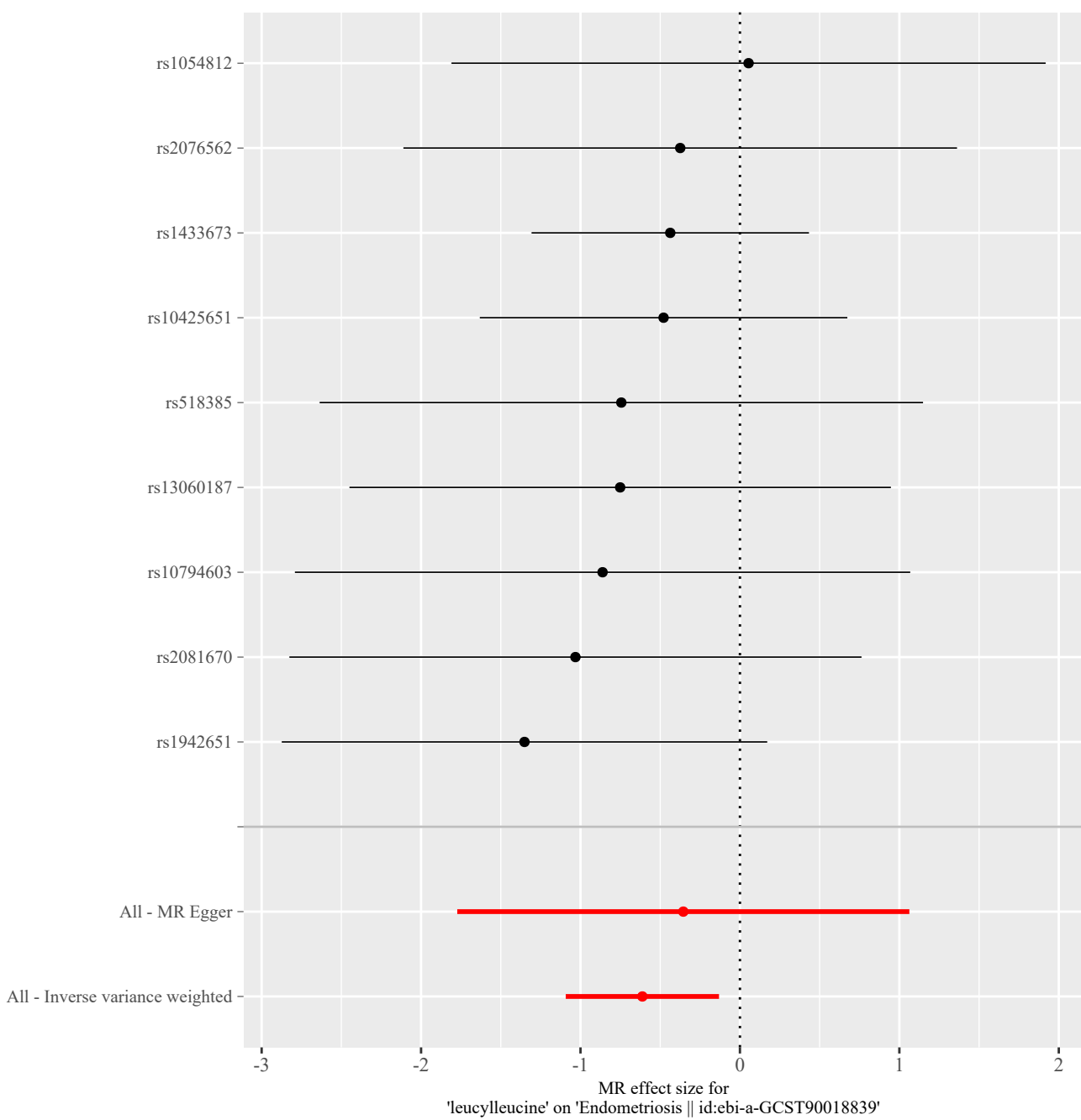

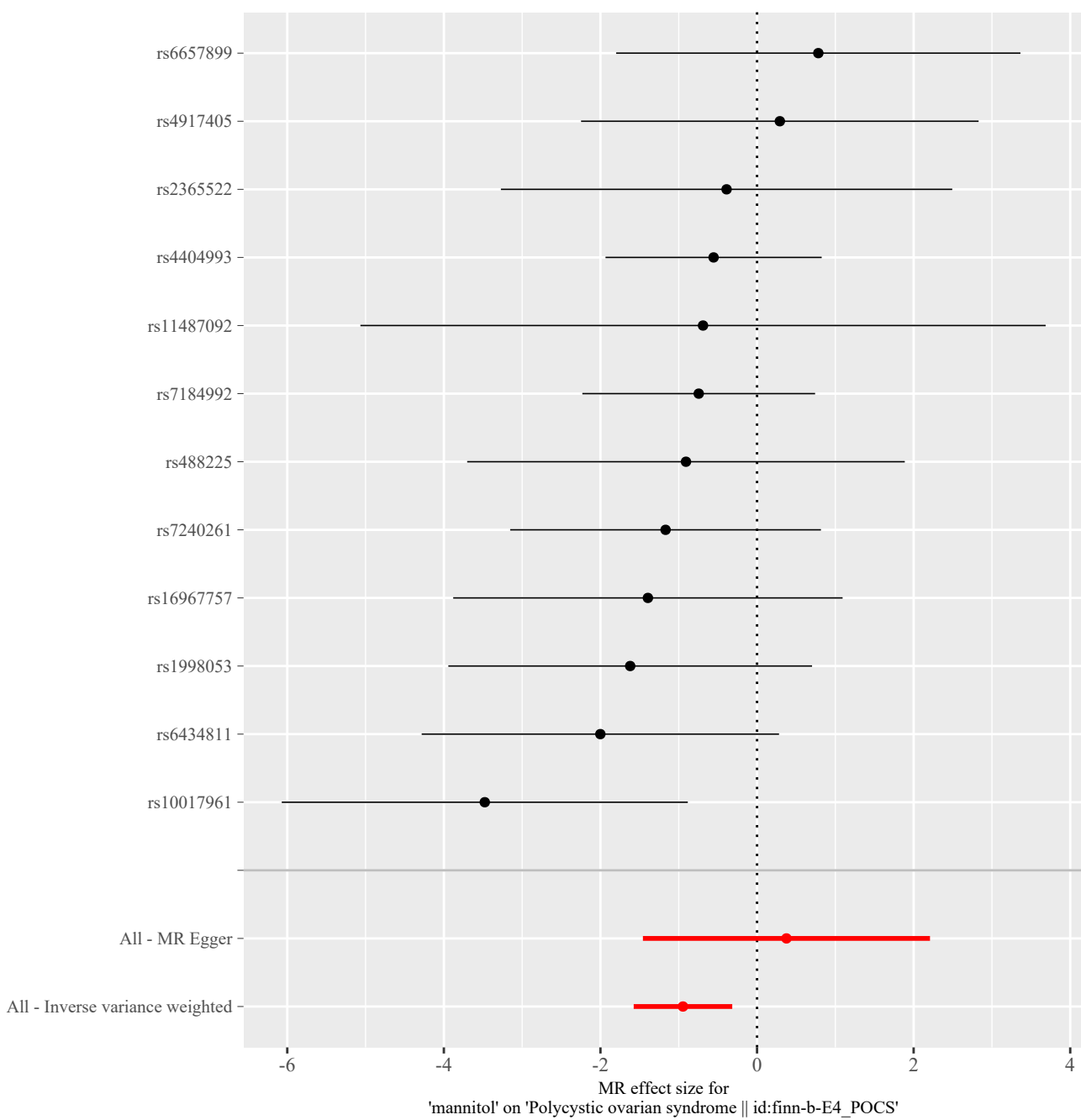

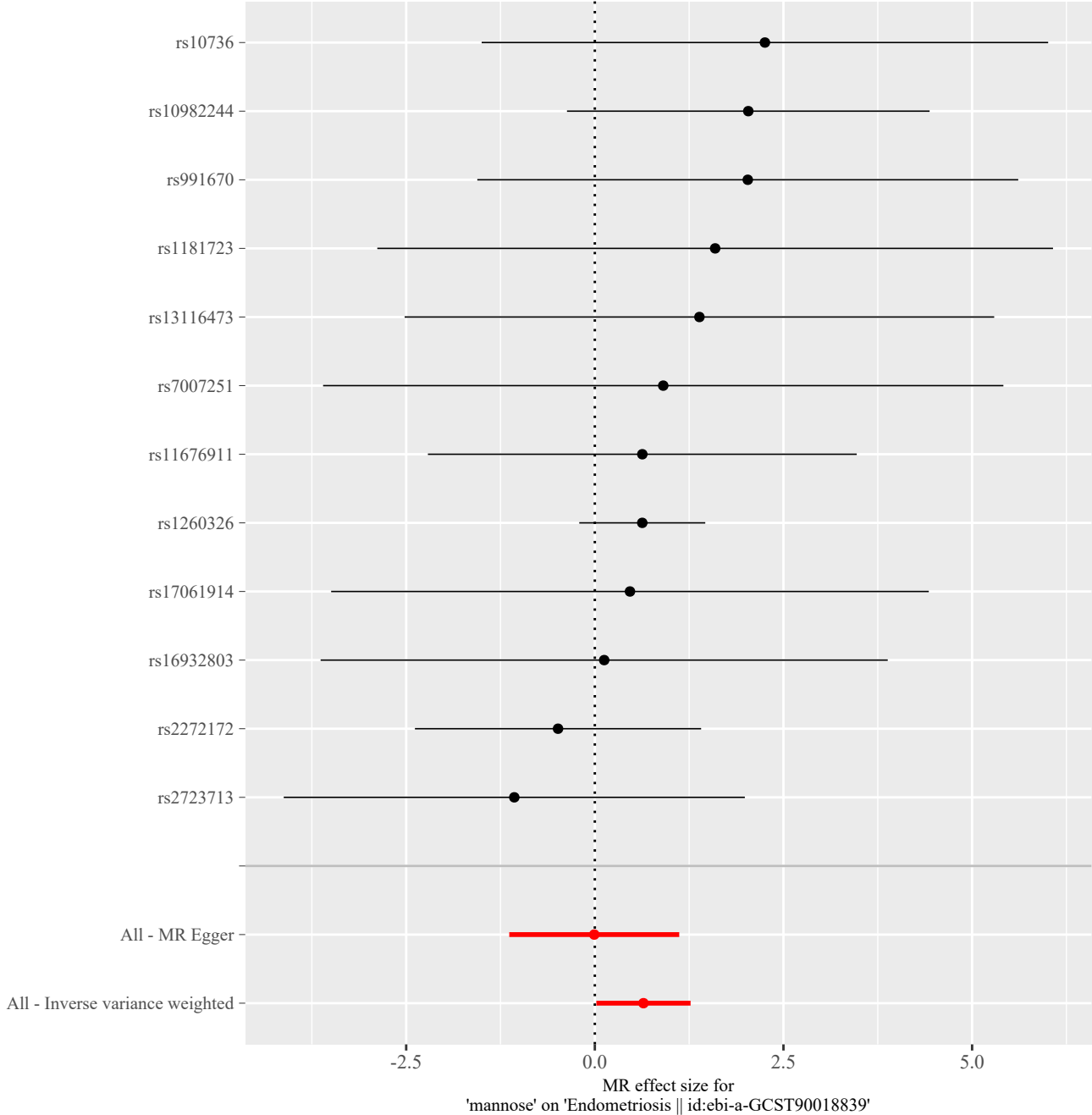

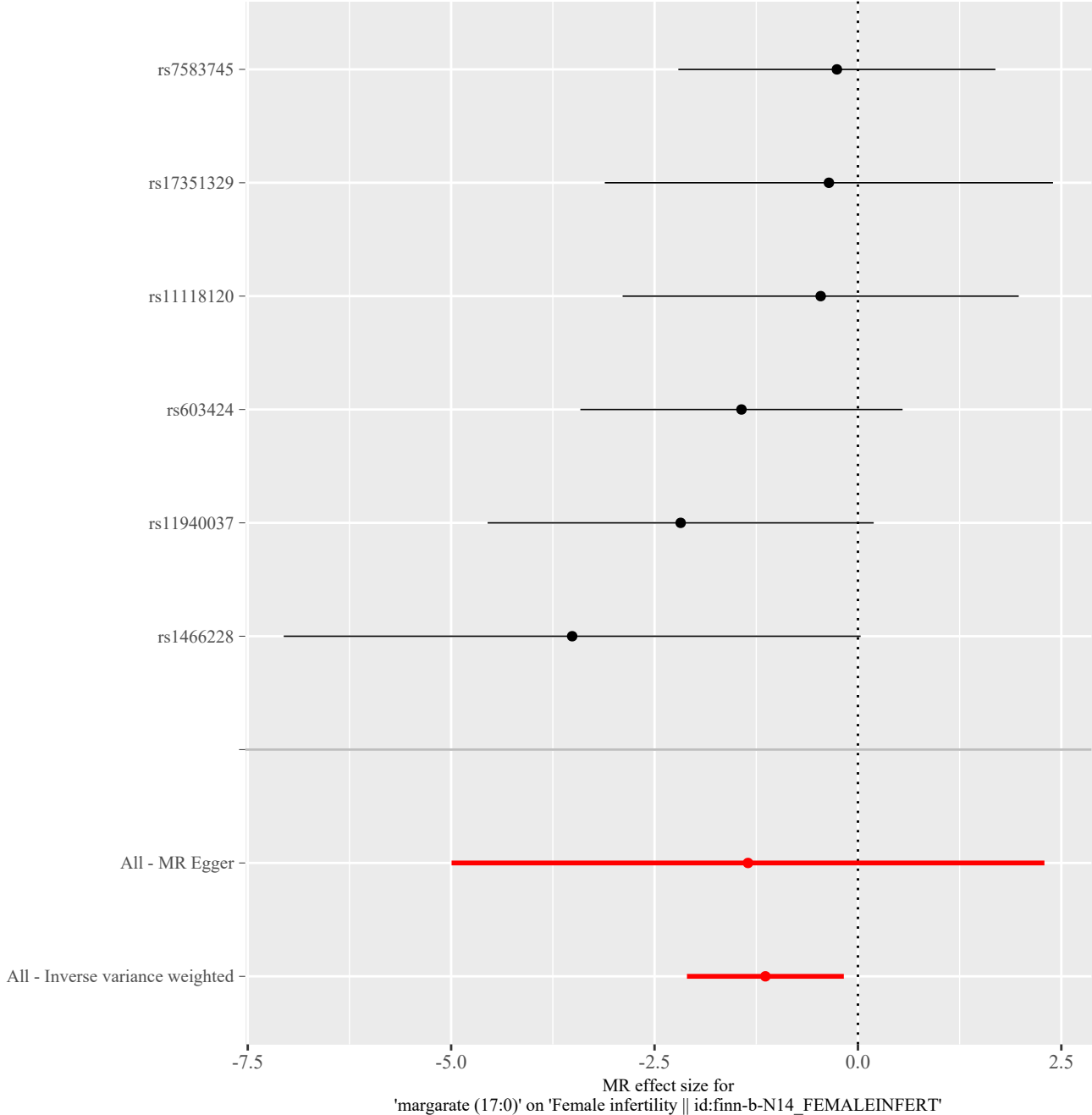

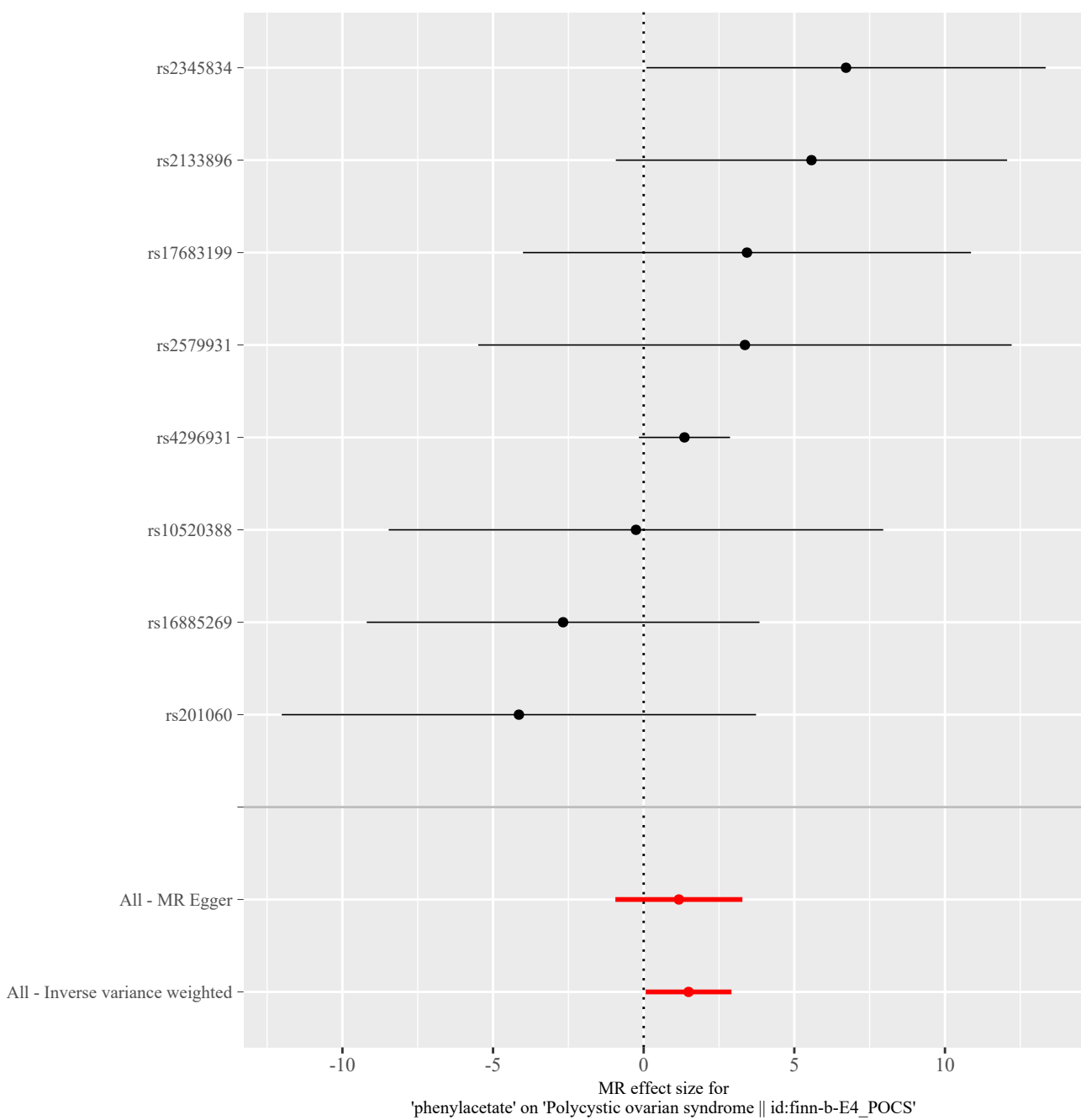

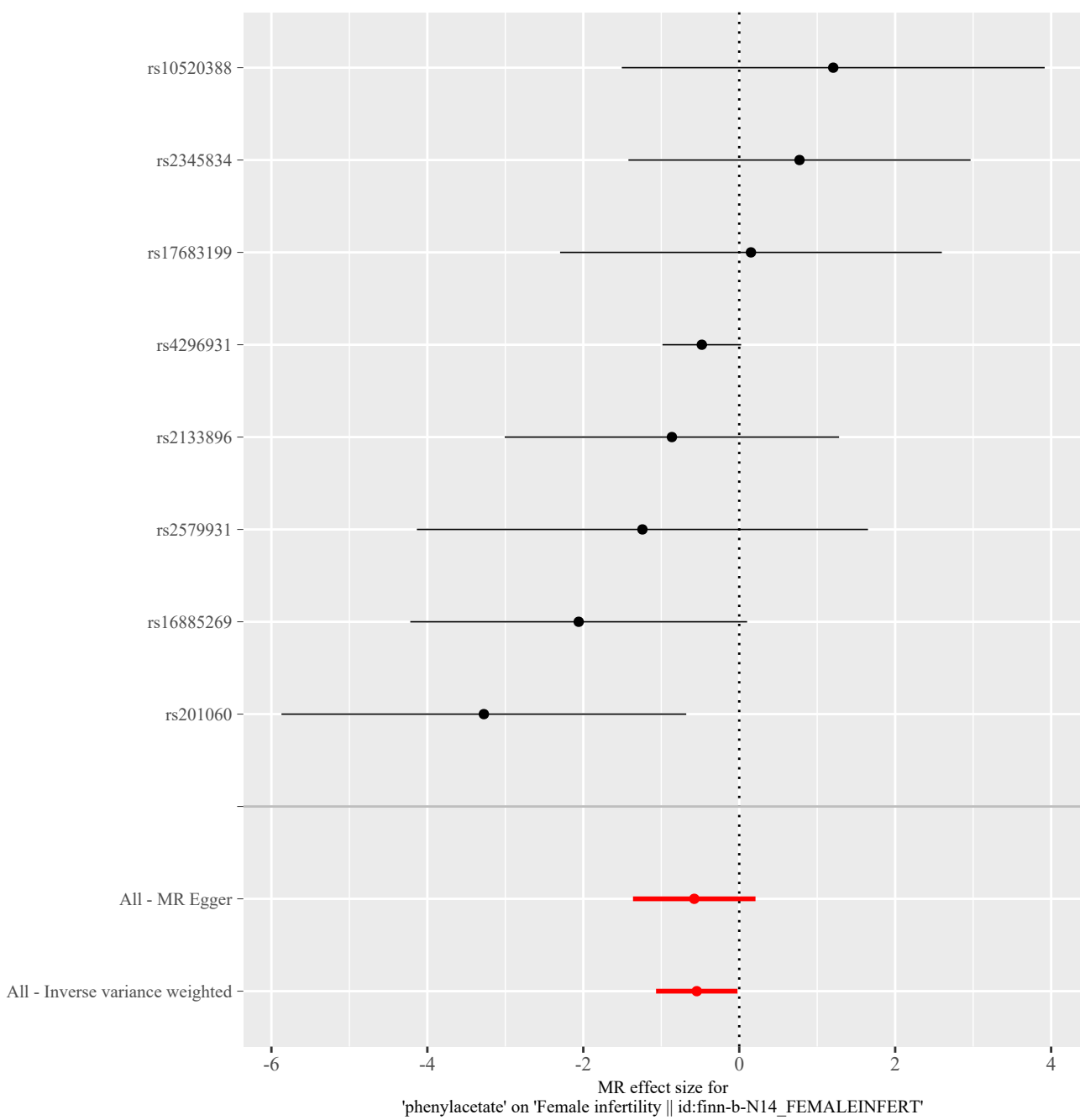

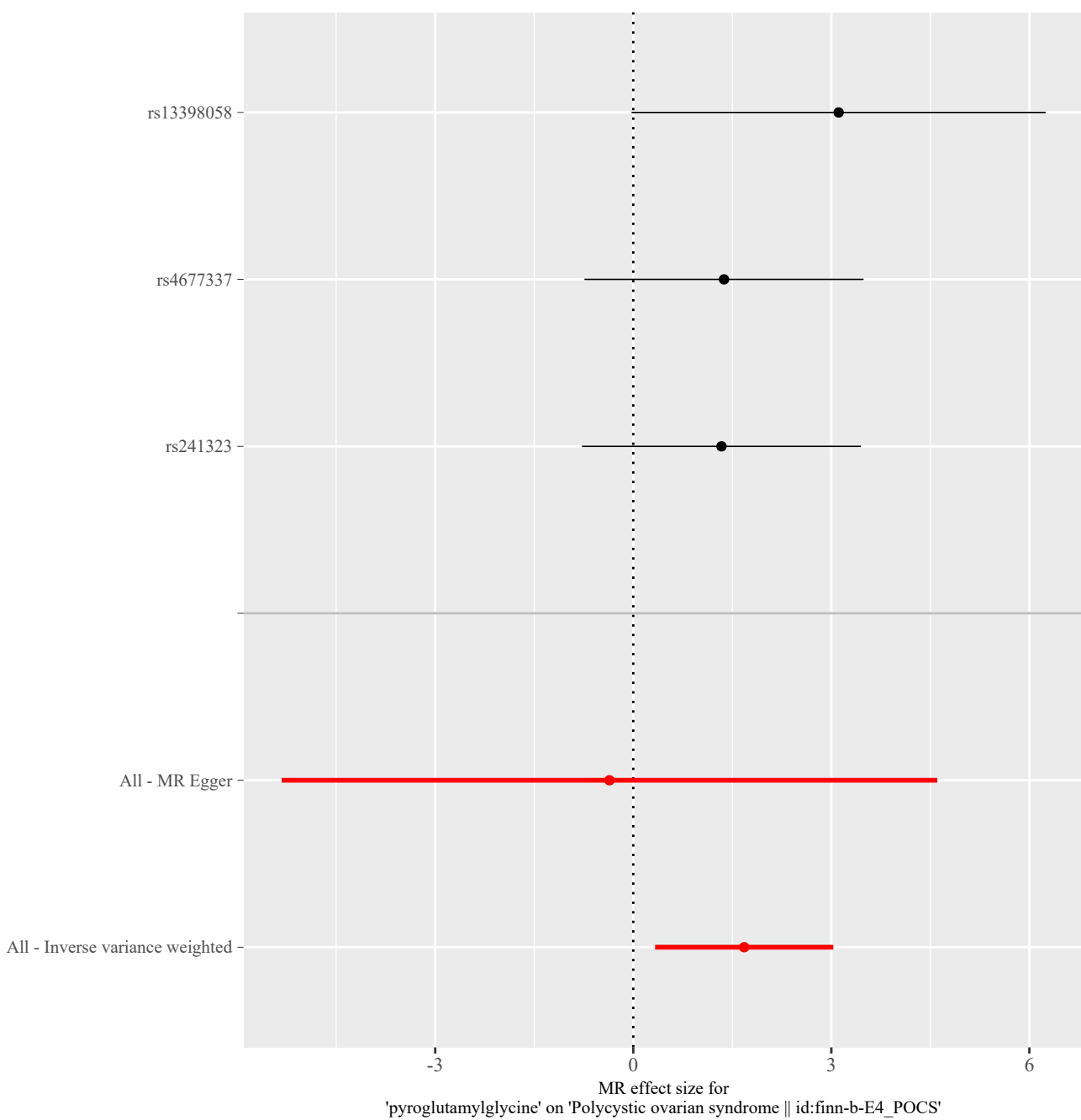

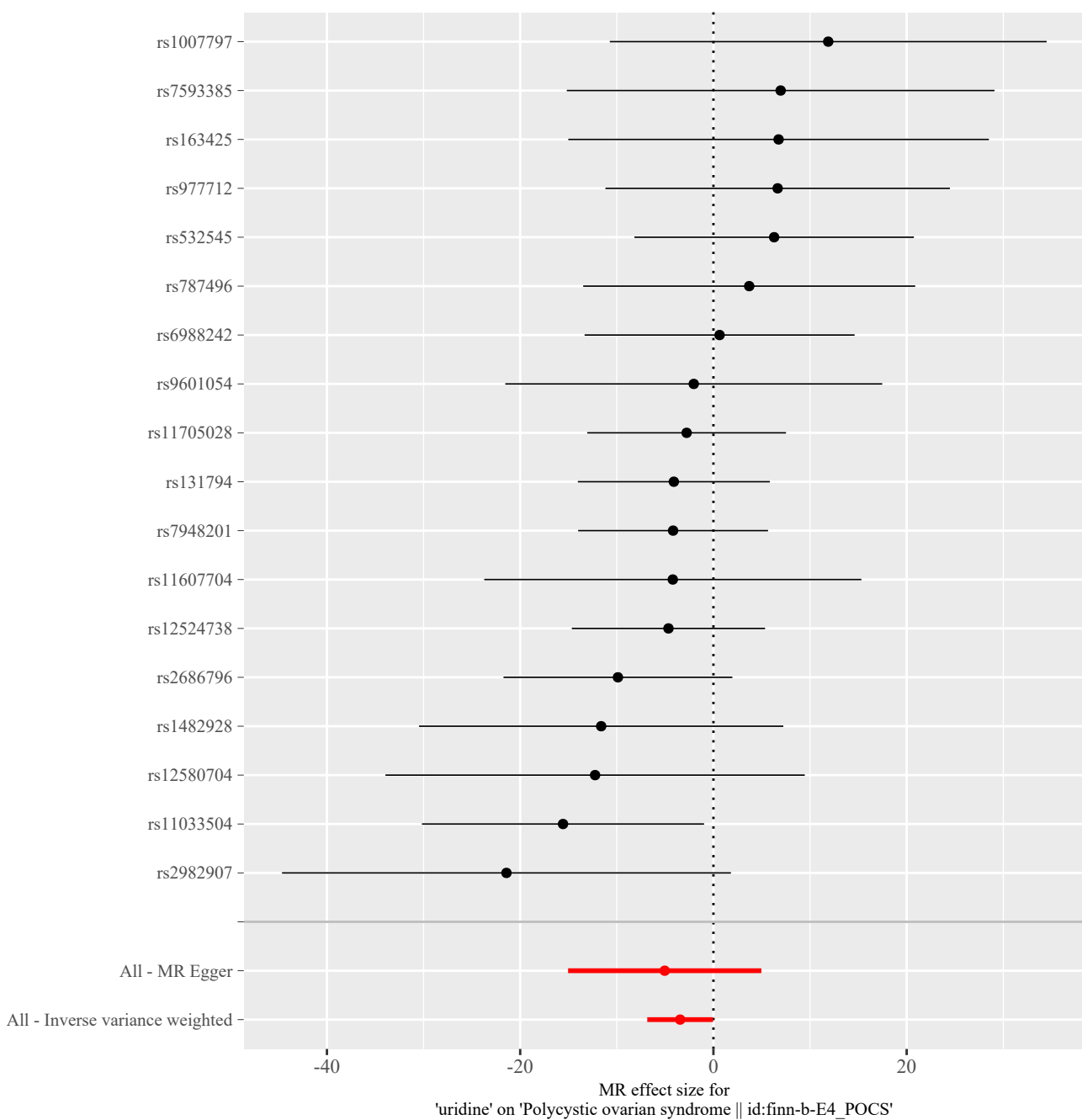

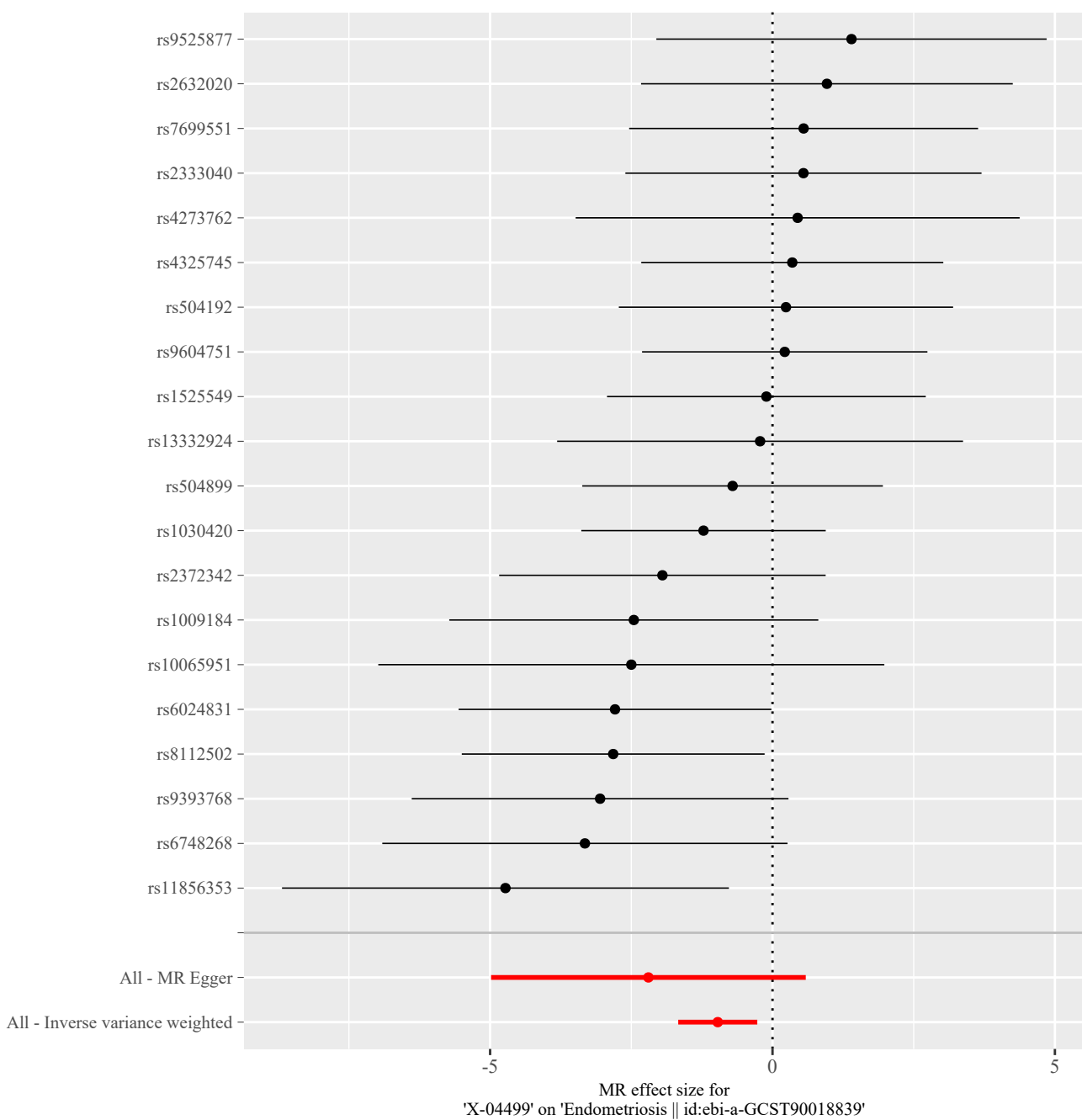

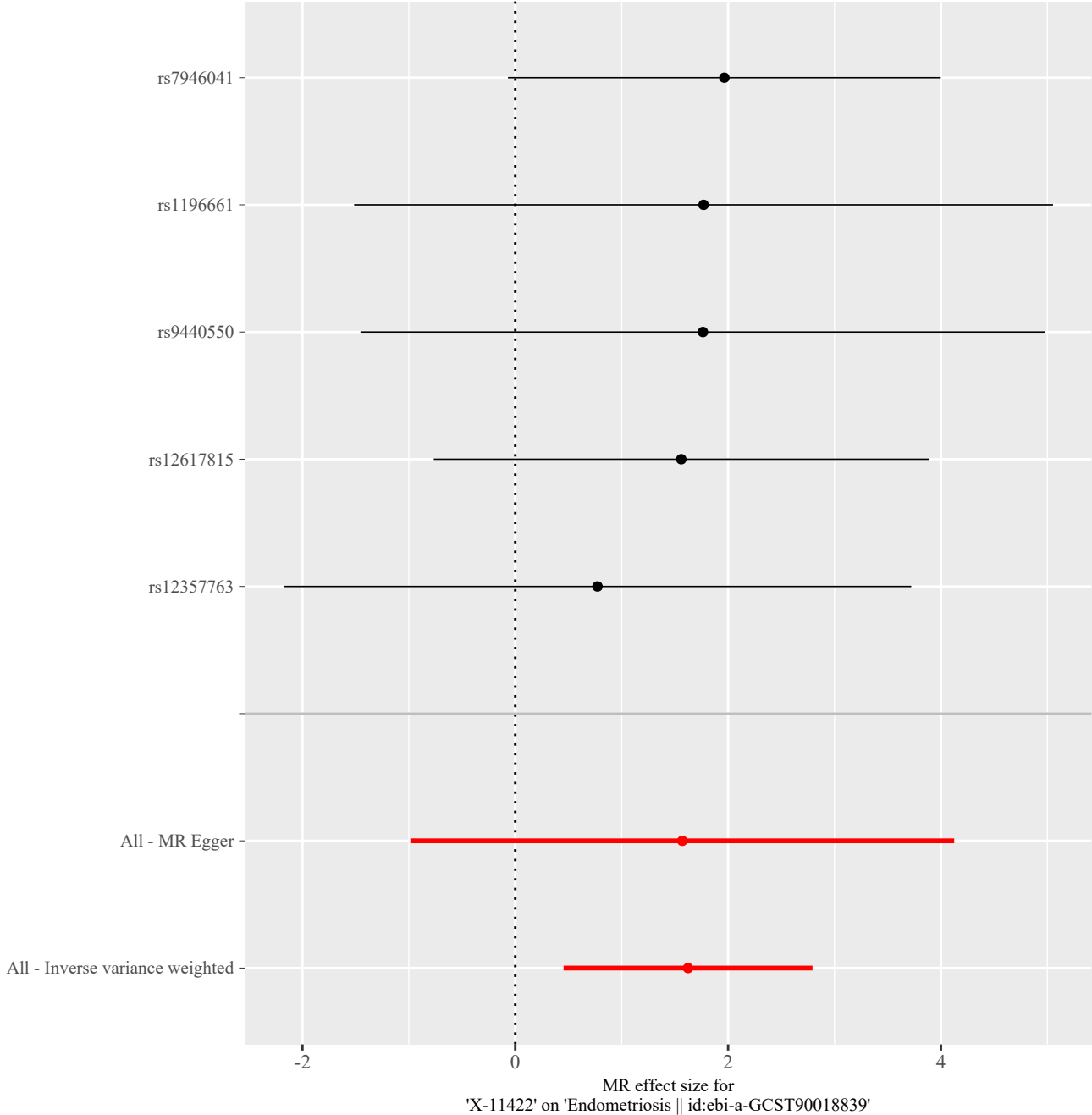

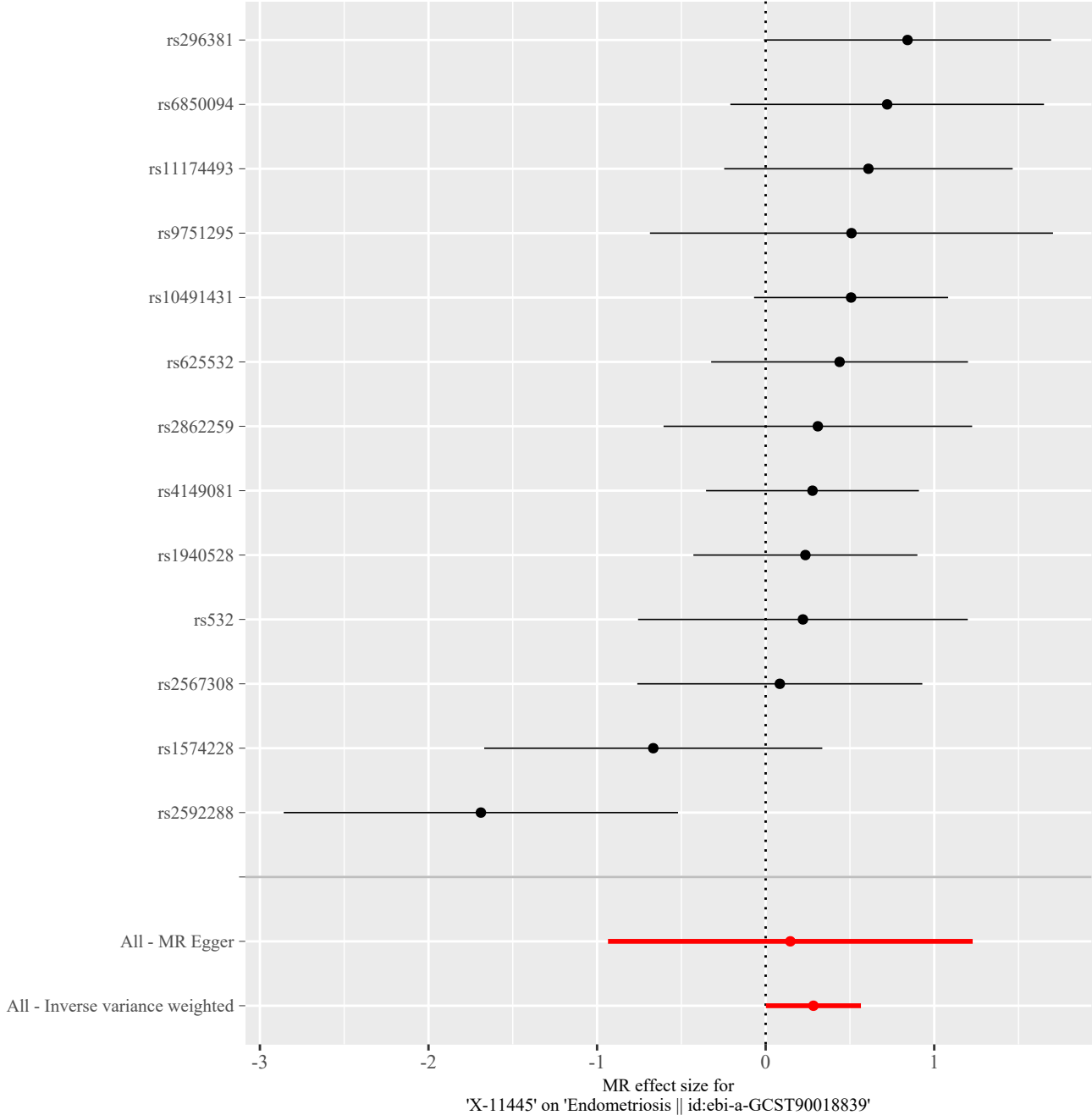

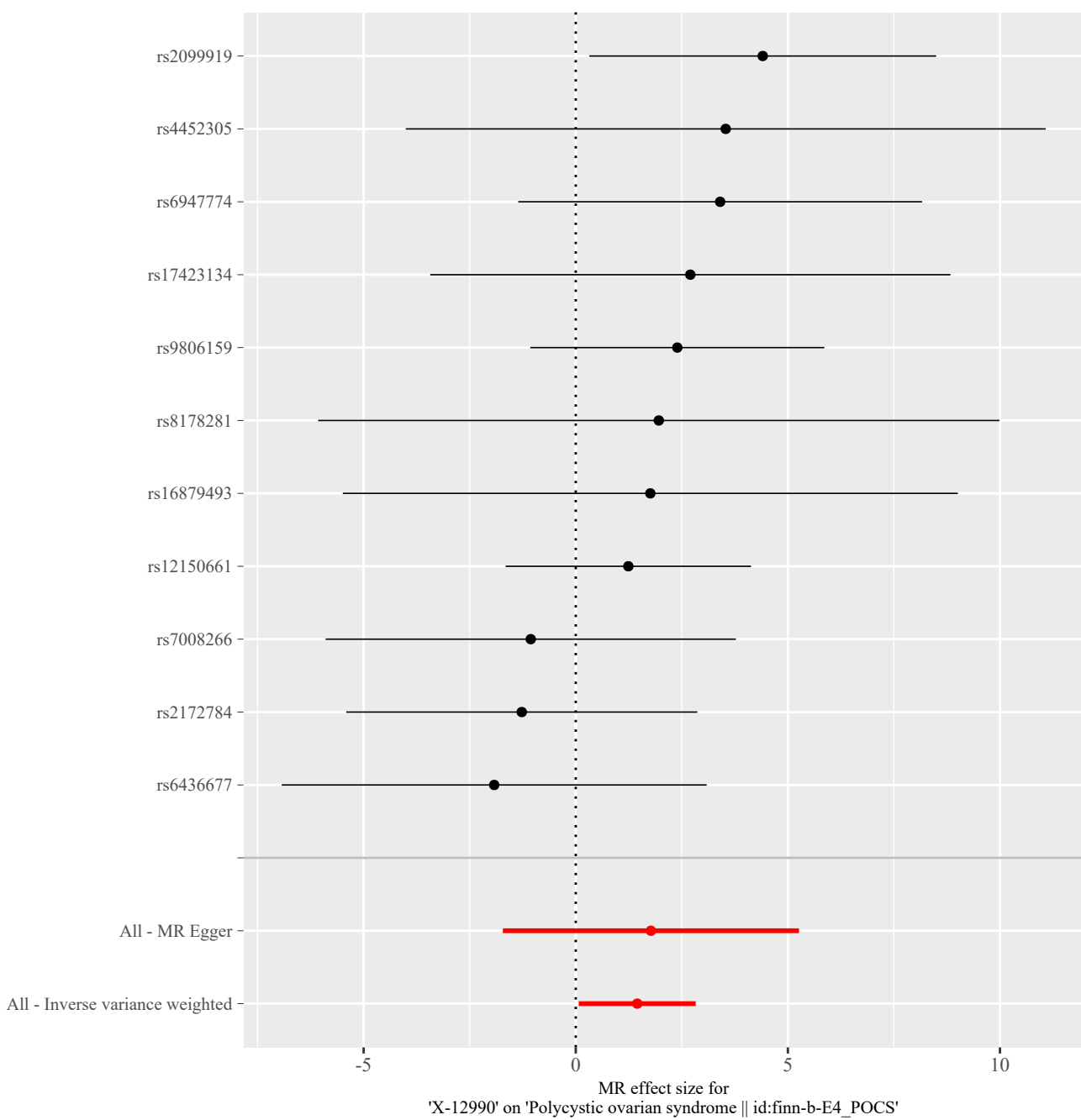

Supplement: Supplementary Figure 3 — Forest plot. The forest plot aggregates the effect sizes and confidence intervals for each metabolite’s impact on reproductive health outcomes, facilitating a meta-analytic view of their significance and heterogeneity. [file Image3.pdf]
