# Supplementary material for: Investigating the metabolomic pathways in female reproductive endocrine disorders: a Mendelian randomization study
Source: Front Endocrinol (Lausanne). 2024 Oct 31;15:1438079. doi: 10.3389/fendo.2024.1438079 (PMC11560792; doi:10.3389/fendo.2024.1438079)
Supplement: Supplementary Figure 4 — Leave-one-out plot. The leave-one-out plot examines the influence of each individual instrumental variable on the overall MR results, ensuring the robustness and reliability of the associations with female infertility, polycystic ovary syndrome, and endometriosis. [file Image4.pdf]

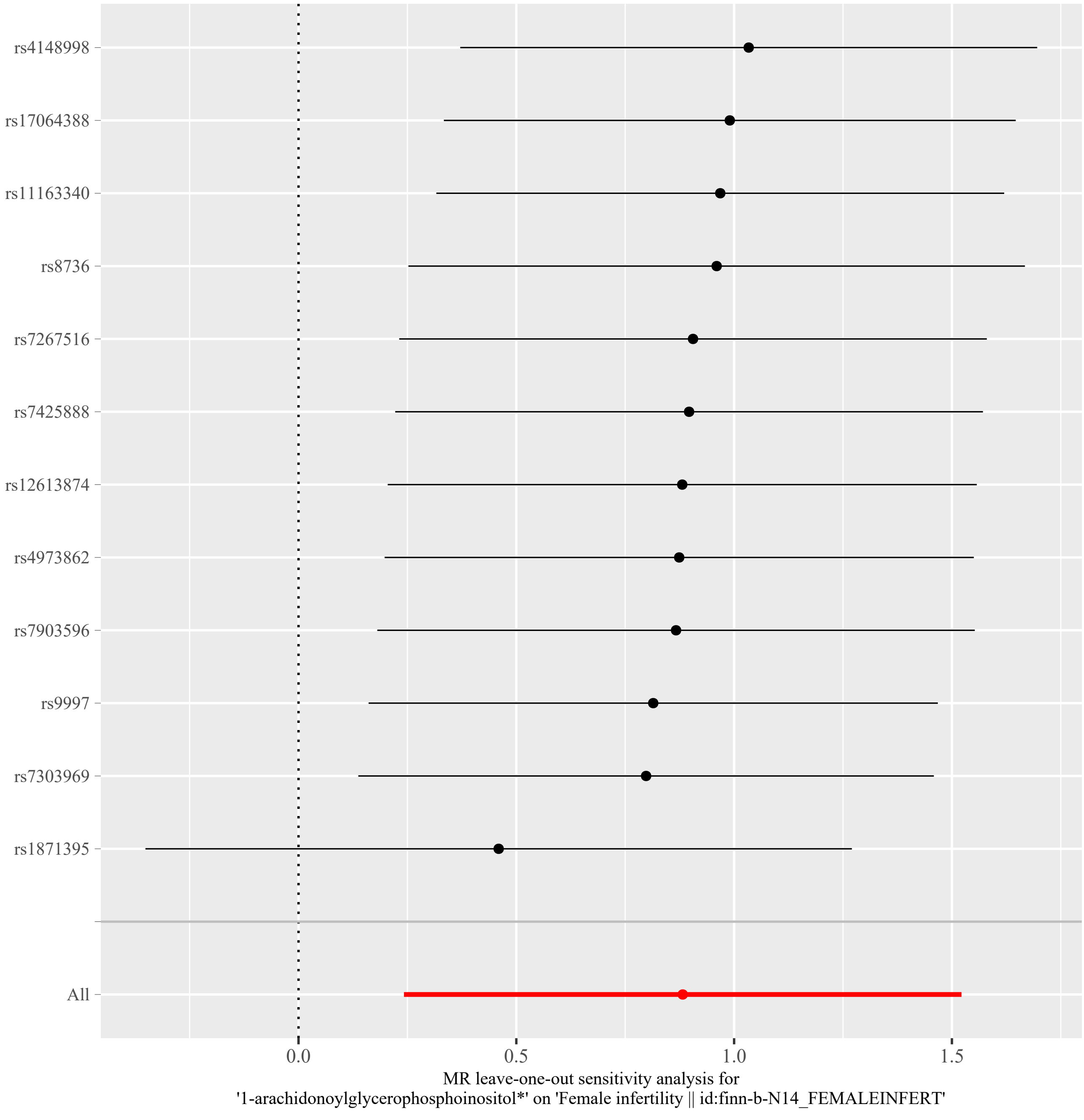
rs4148998
rs17064388
rs11163340
rs8736
rs7267516
rs7425888
rs12613874
rs4973862
rs7903596
rs9997
rs7303969
rs1871395
All
0.0
0.5
1.0
1.5
MR leave-one-out sensitivity analysis for
'1-arachidonoylglycerophosphoinositol*' on 'Female infertility || id:finn-b-N14_FEMALEINFERT'

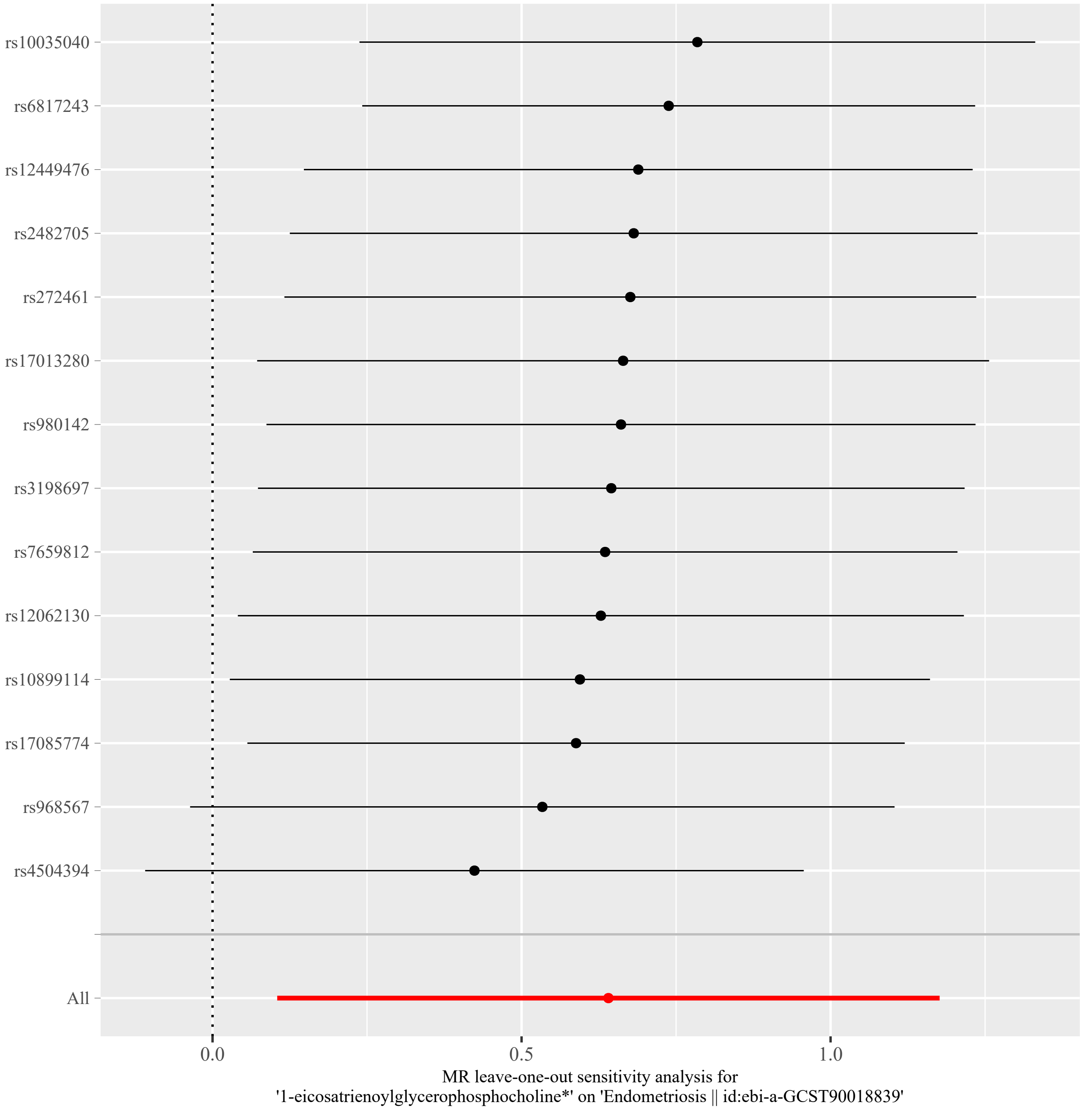

rs10035040
rs6817243
rs12449476
rs2482705
rs272461
rs17013280
rs980142
rs3198697
rs7659812
rs12062130
rs10899114
rs17085774
rs968567
rs4504394
All
0.0
0.5
1.0
MR leave-one-out sensitivity analysis for
'1-eicosatrienoylglycerophosphocholine*' on 'Endometriosis || id:ebi-a-GCST90018839'

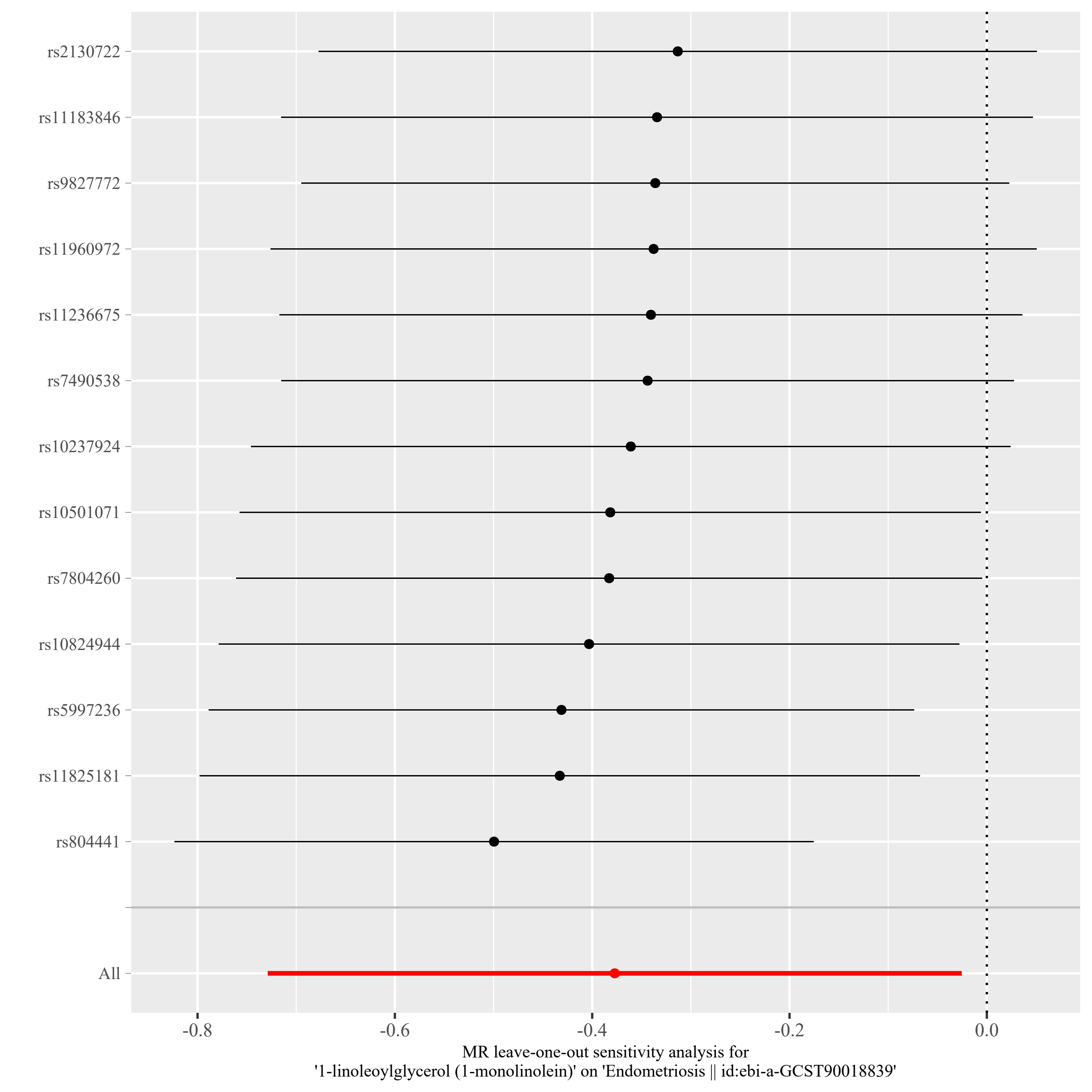
rs2130722
rs11183846
rs9827772
rs11960972
rs11236675
rs7490538
rs10237924
rs10501071
rs7804260
rs10824944
rs5997236
rs11825181
rs804441
All
-0.8
-0.6
-0.4
-0.2
0.0
MR leave-one-out sensitivity analysis for
'1-linoleoylglycerol (1-monolinolein)' on 'Endometriosis || id:ebi-a-GCST90018839'

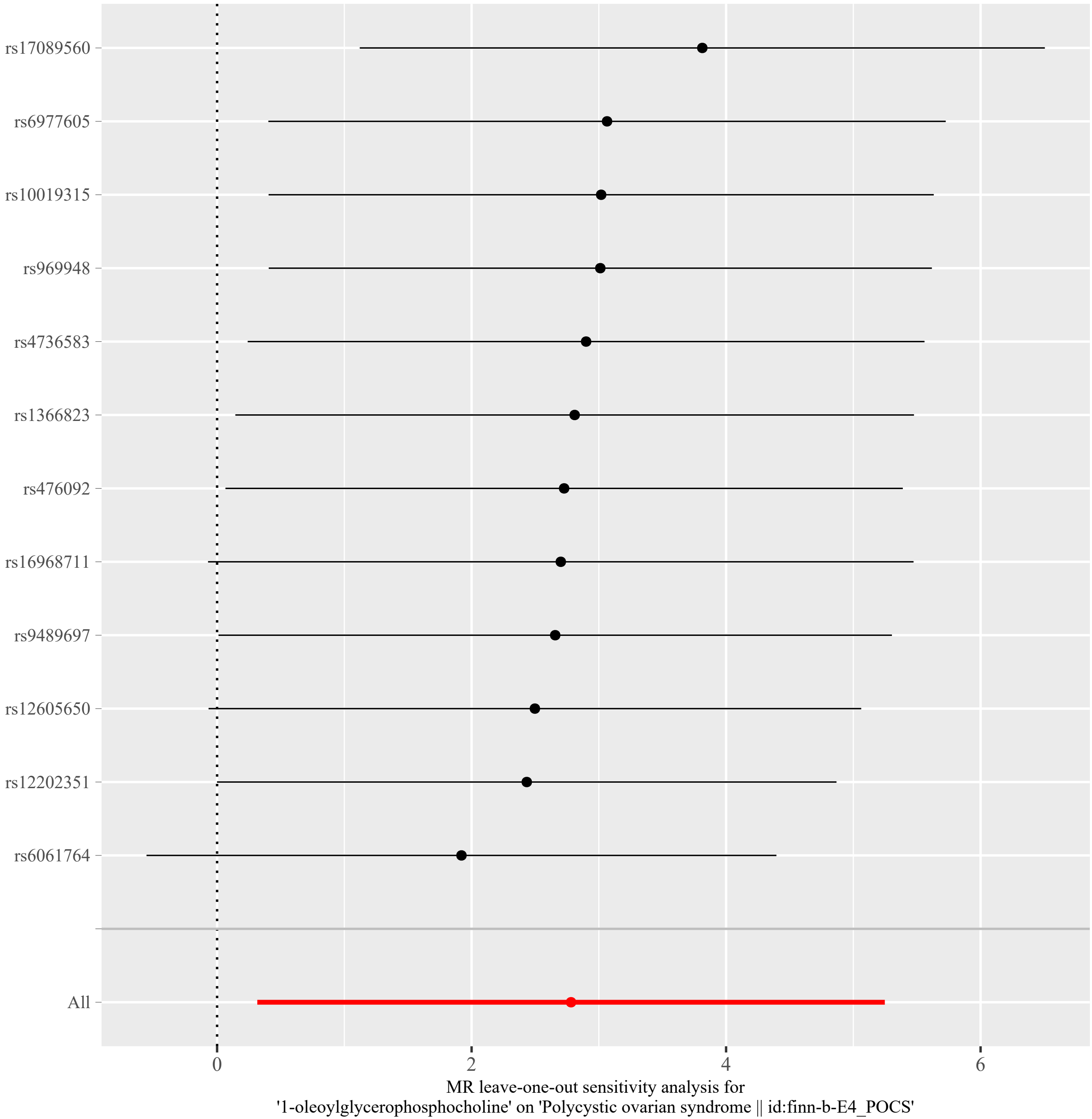
rs17089560
rs6977605
rs10019315
rs969948
rs4736583
rs1366823
rs476092
rs16968711
rs9489697
rs12605650
rs12202351
rs6061764
All
0
2
4
6
MR leave-one-out sensitivity analysis for
'1-oleoylglycerophosphocholine' on 'Polycystic ovarian syndrome || id:finn-b-E4_POCS'

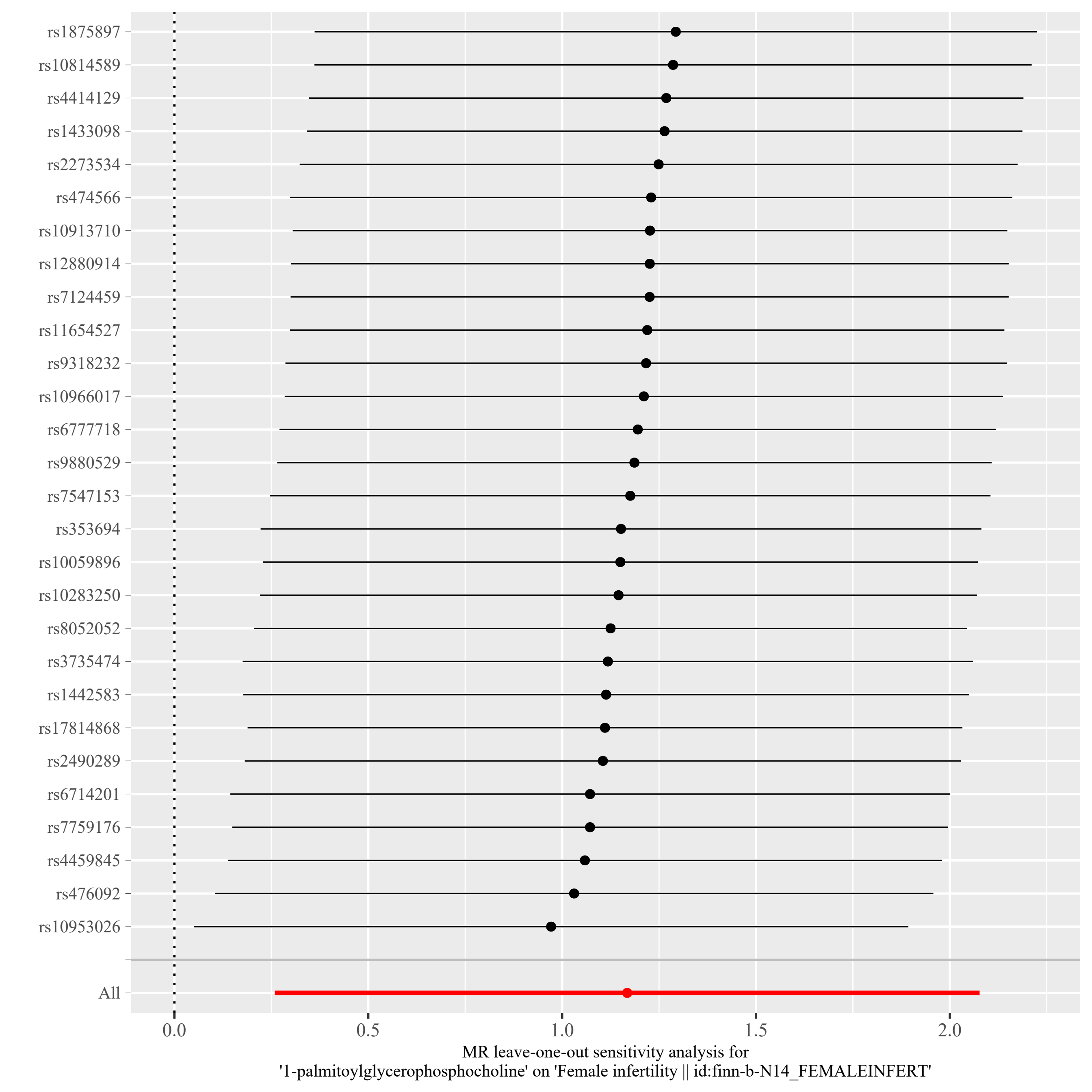
rs1875897
rs10814589
rs4414129
rs1433098
rs2273534
rs474566
rs10913710
rs12880914
rs7124459
rs11654527
rs9318232
rs10966017
rs6777718
rs9880529
rs7547153
rs353694
rs10059896
rs10283250
rs8052052
rs3735474
rs1442583
rs17814868
rs2490289
rs6714201
rs7759176
rs4459845
rs476092
rs10953026
All
0.0
0.5
1.0
1.5
2.0
MR leave-one-out sensitivity analysis for
'1-palmitoylglycerophosphocholine' on 'Female infertility || id:finn-b-N14_FEMALEINFERT'

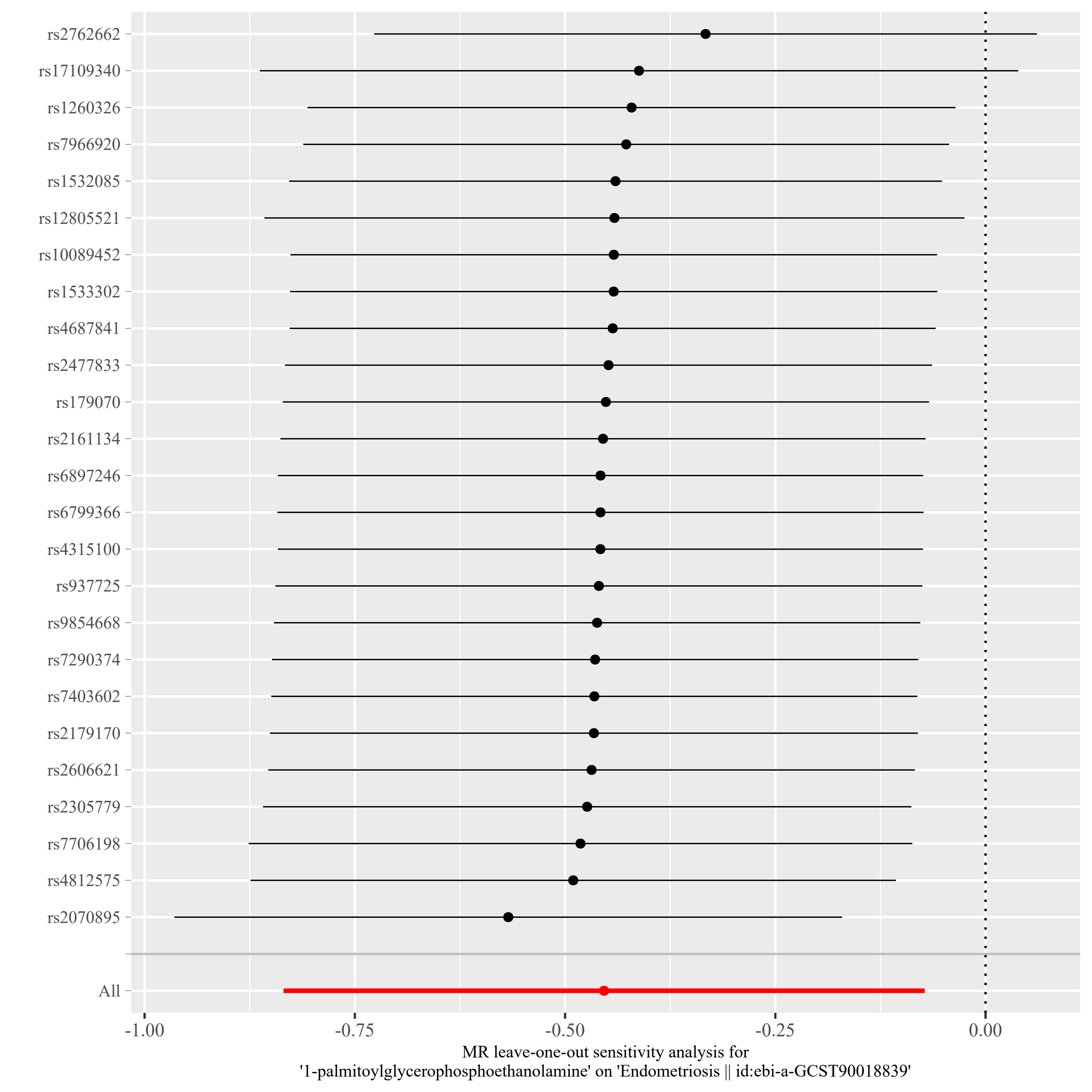
rs2762662
rs17109340
rs1260326
rs7966920
rs1532085
rs12805521
rs10089452
rs1533302
rs4687841
rs2477833
rs179070
rs2161134
rs6897246
rs6799366
rs4315100
rs937725
rs9854668
rs7290374
rs7403602
rs2179170
rs2606621
rs2305779
rs7706198
rs4812575
rs2070895
All
-1.00
-0.75
-0.50
-0.25
0.00
MR leave-one-out sensitivity analysis for
'1-palmitoylglycerophosphoethanolamine' on 'Endometriosis || id:ebi-a-GCST90018839'

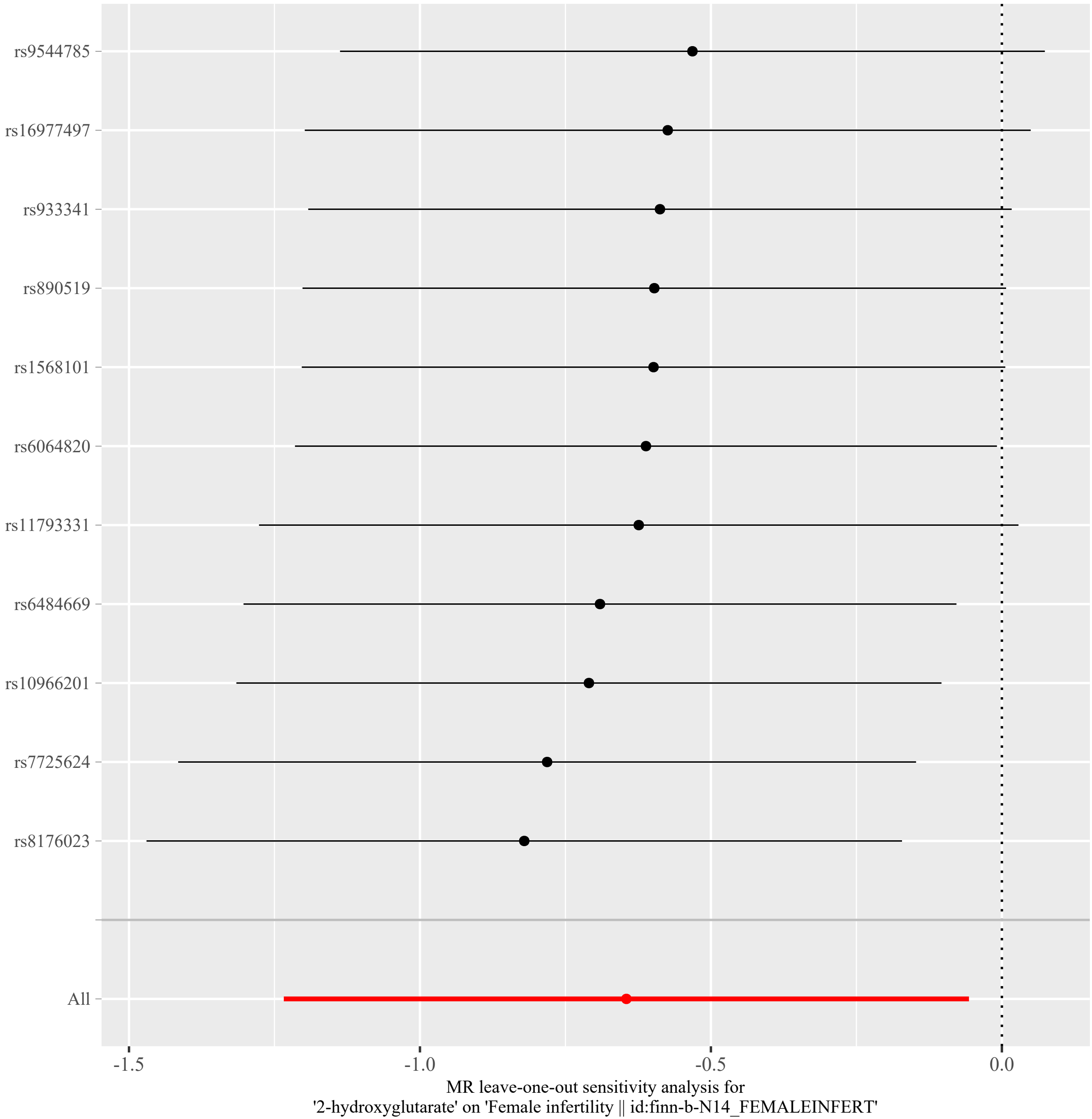
rs9544785
rs16977497
rs933341
rs890519
rs1568101
rs6064820
rs11793331
rs6484669
rs10966201
rs7725624
rs8176023
All
-1.5
-1.0
-0.5
0.0
MR leave-one-out sensitivity analysis for
'2-hydroxyglutarate' on 'Female infertility || id:finn-b-N14_FEMALEINFERT'

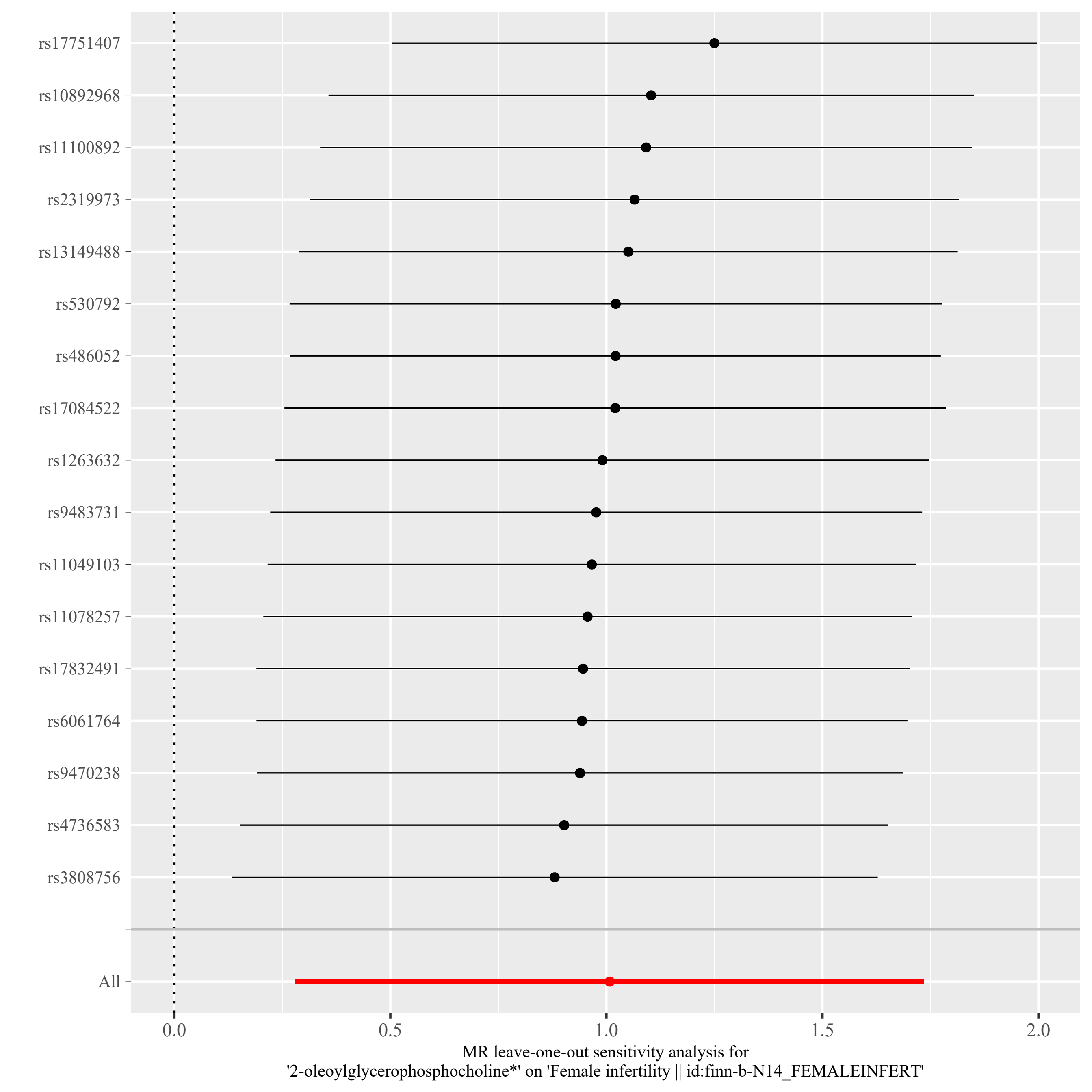
rs17751407
rs10892968
rs11100892
rs2319973
rs13149488
rs530792
rs486052
rs17084522
rs1263632
rs9483731
rs11049103
rs11078257
rs17832491
rs6061764
rs9470238
rs4736583
rs3808756
All
0.0
0.5
1.0
1.5
2.0
MR leave-one-out sensitivity analysis for
'2-oleoylglycerophosphocholine*' on 'Female infertility || id:finn-b-N14_FEMALEINFERT'

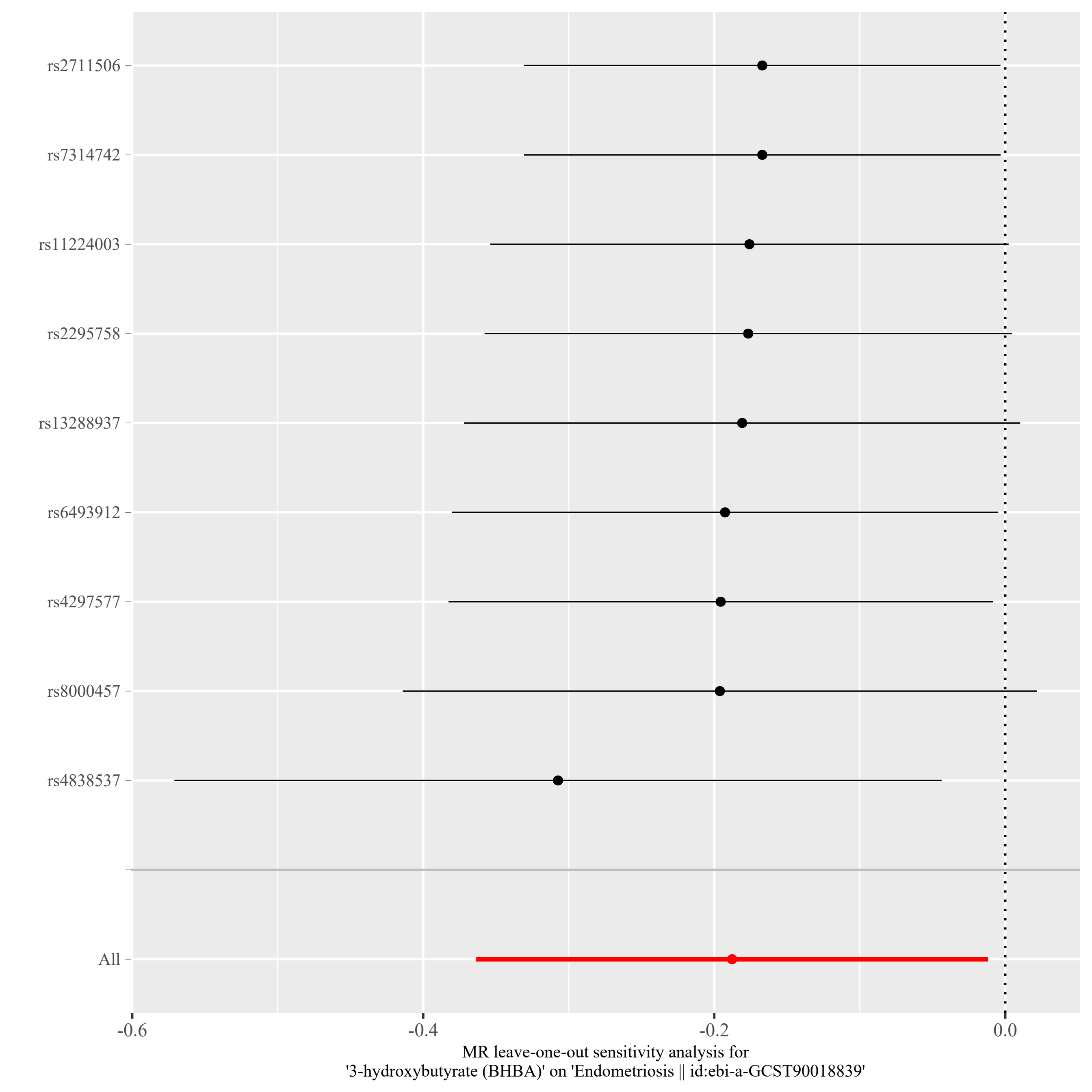
rs2711506
rs7314742
rs11224003
rs2295758
rs13288937
rs6493912
rs4297577
rs8000457
rs4838537
All
-0.6
-0.4
-0.2
0.0
MR leave-one-out sensitivity analysis for
'3-hydroxybutyrate (BHBA)' on 'Endometriosis || id:ebi-a-GCST90018839'

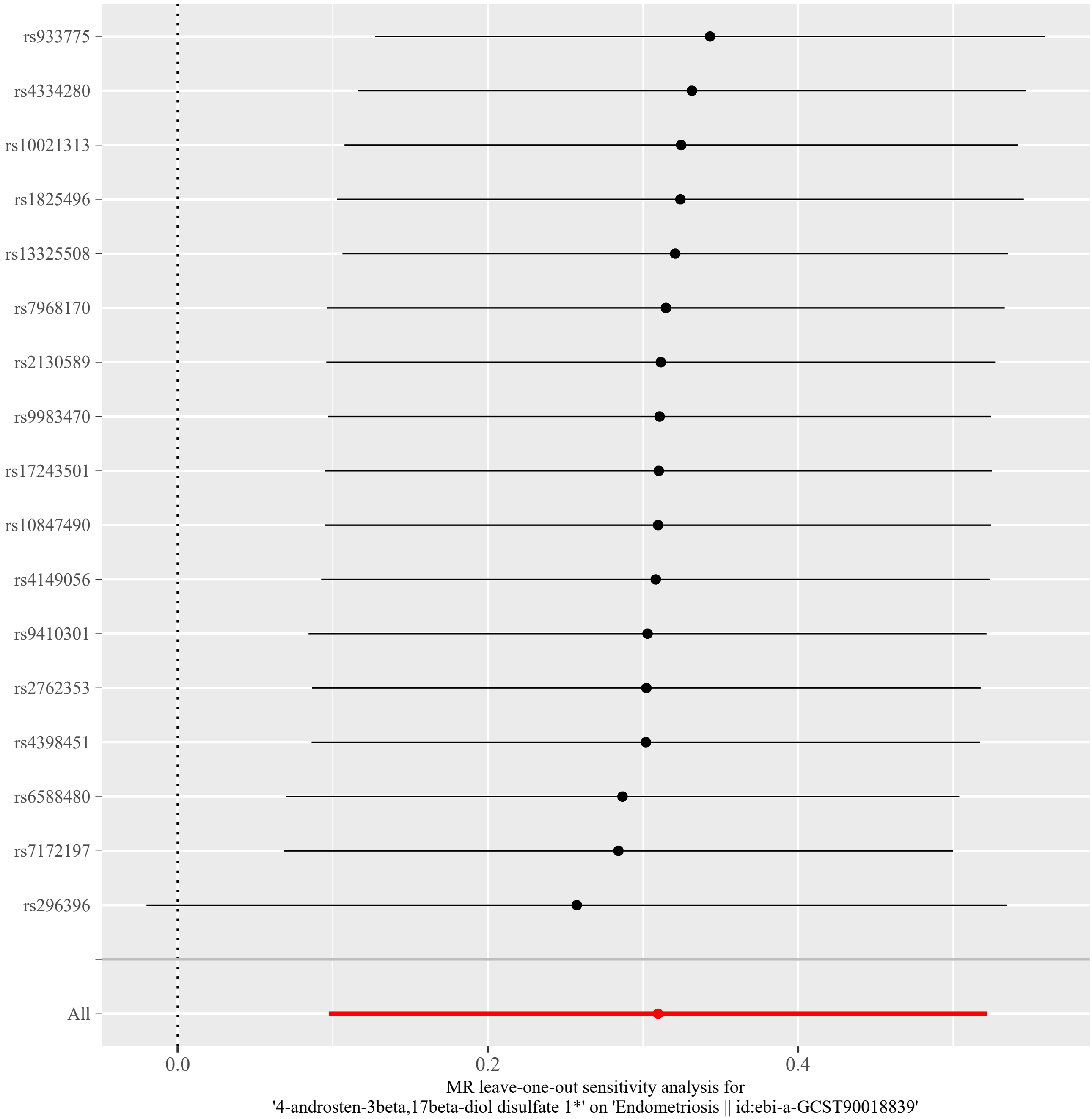
rs933775
rs4334280
rs10021313
rs1825496
rs13325508
rs7968170
rs2130589
rs9983470
rs17243501
rs10847490
rs4149056
rs9410301
rs2762353
rs4398451
rs6588480
rs7172197
rs296396
All
0.0
0.2
0.4
MR leave-one-out sensitivity analysis for
'4-androsten-3beta,17beta-diol disulfate 1*' on 'Endometriosis || id:ebi-a-GCST90018839'

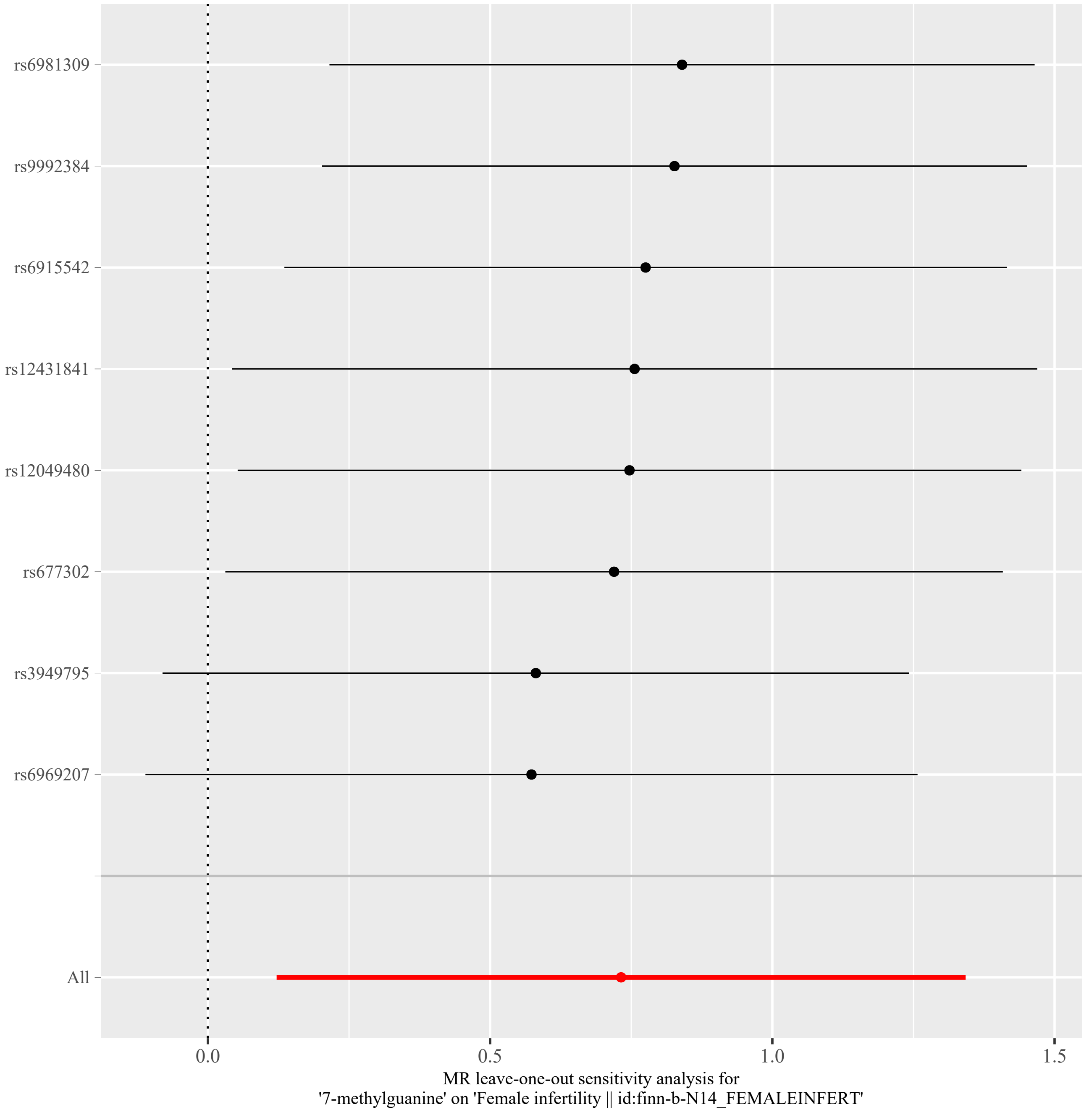
rs6981309
rs9992384
rs6915542
rs12431841
rs12049480
rs677302
rs3949795
rs6969207
All
0.0
0.5
1.0
1.5
MR leave-one-out sensitivity analysis for
'7-methylguanine' on 'Female infertility || id:finn-b-N14_FEMALEINFERT'

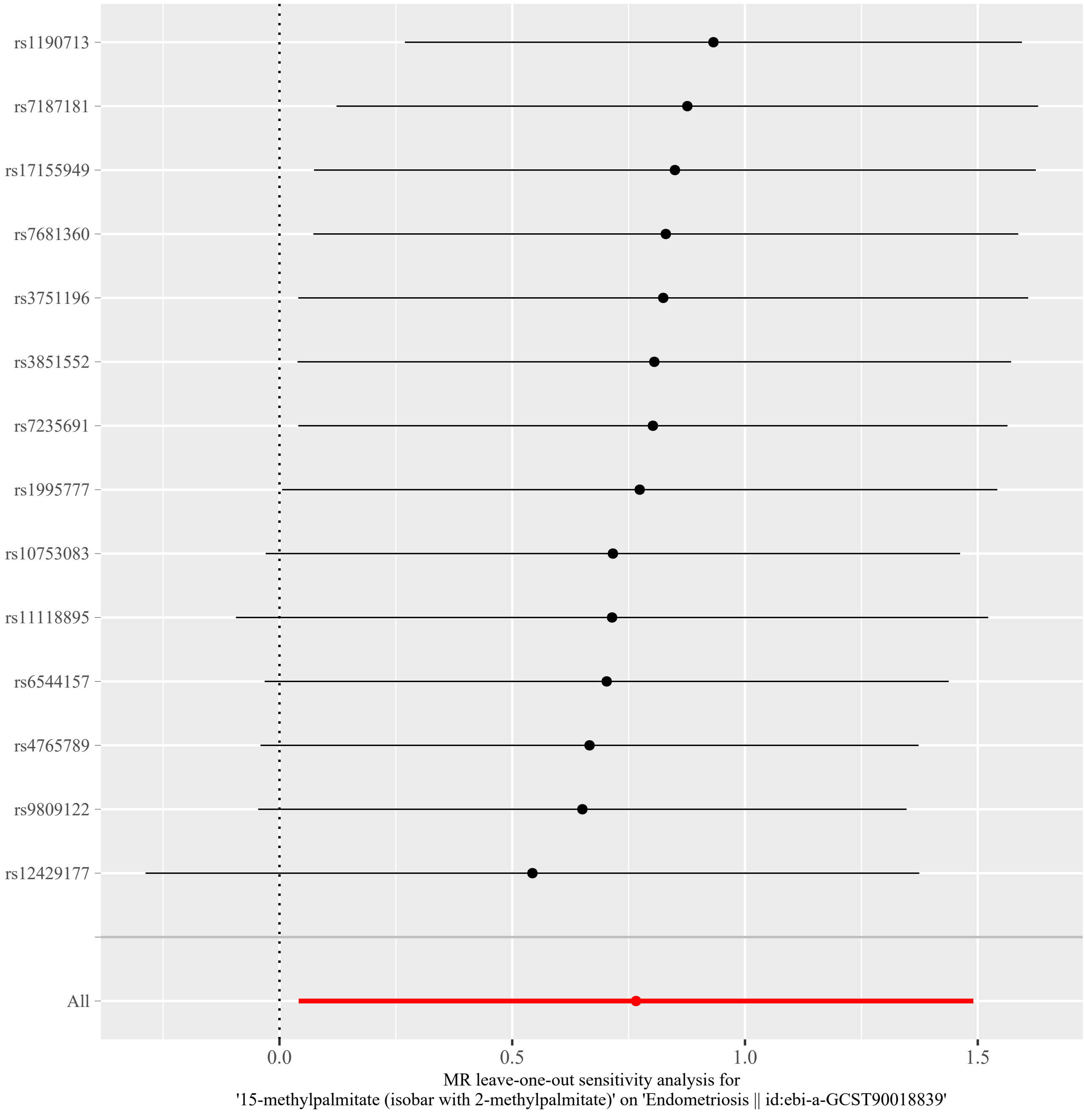
rs1190713
rs7187181
rs17155949
rs7681360
rs3751196
rs3851552
rs7235691
rs1995777
rs10753083
rs11118895
rs6544157
rs4765789
rs9809122
rs12429177
All
0.0
0.5
1.0
1.5
MR leave-one-out sensitivity analysis for
'15-methylpalmitate (isobar with 2-methylpalmitate)' on 'Endometriosis || id:ebi-a-GCST90018839'

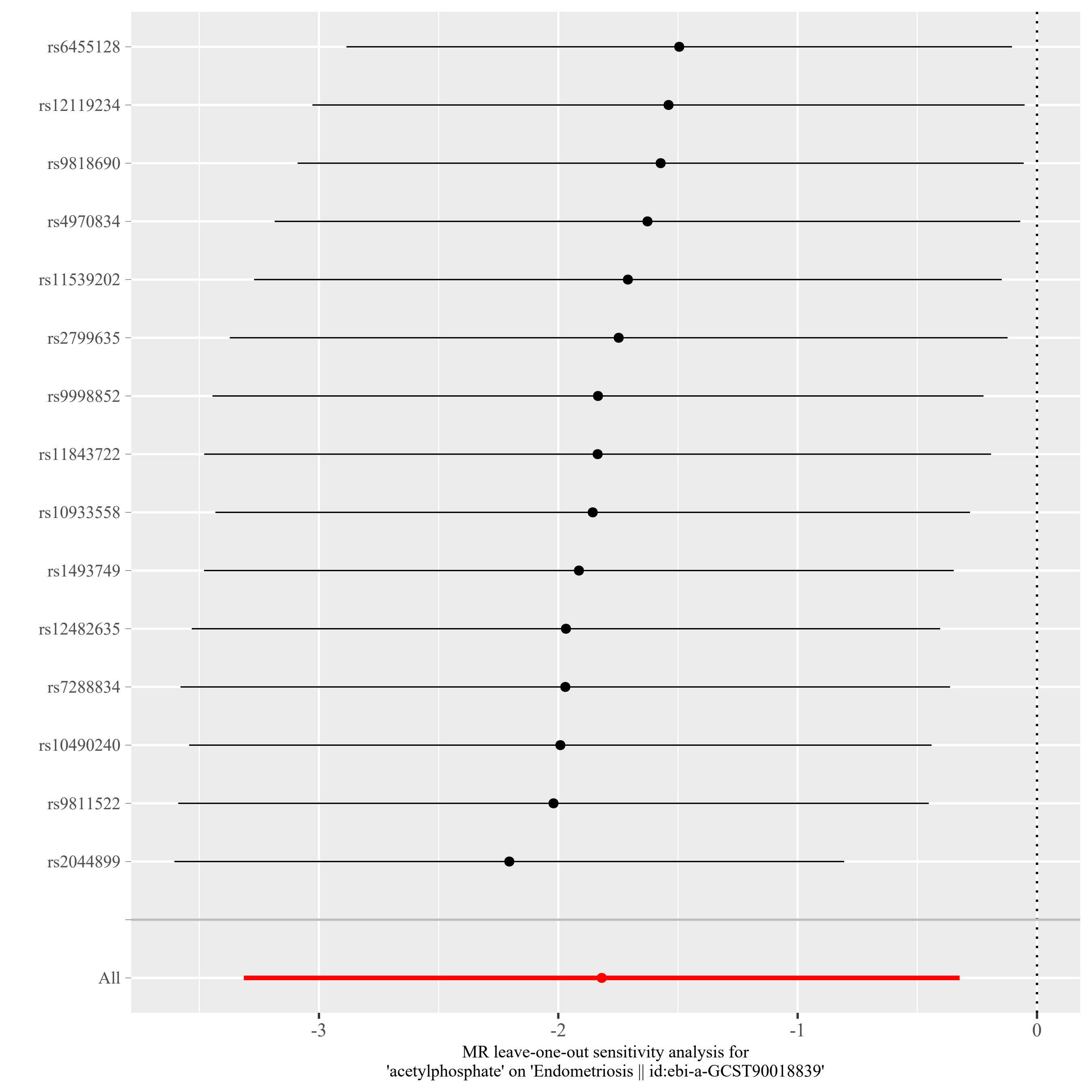
rs6455128
rs12119234
rs9818690
rs4970834
rs11539202
rs2799635
rs9998852
rs11843722
rs10933558
rs1493749
rs12482635
rs7288834
rs10490240
rs9811522
rs2044899
All
-3
-2
-1
0
MR leave-one-out sensitivity analysis for
'acetylphosphate' on 'Endometriosis || id:ebi-a-GCST90018839'

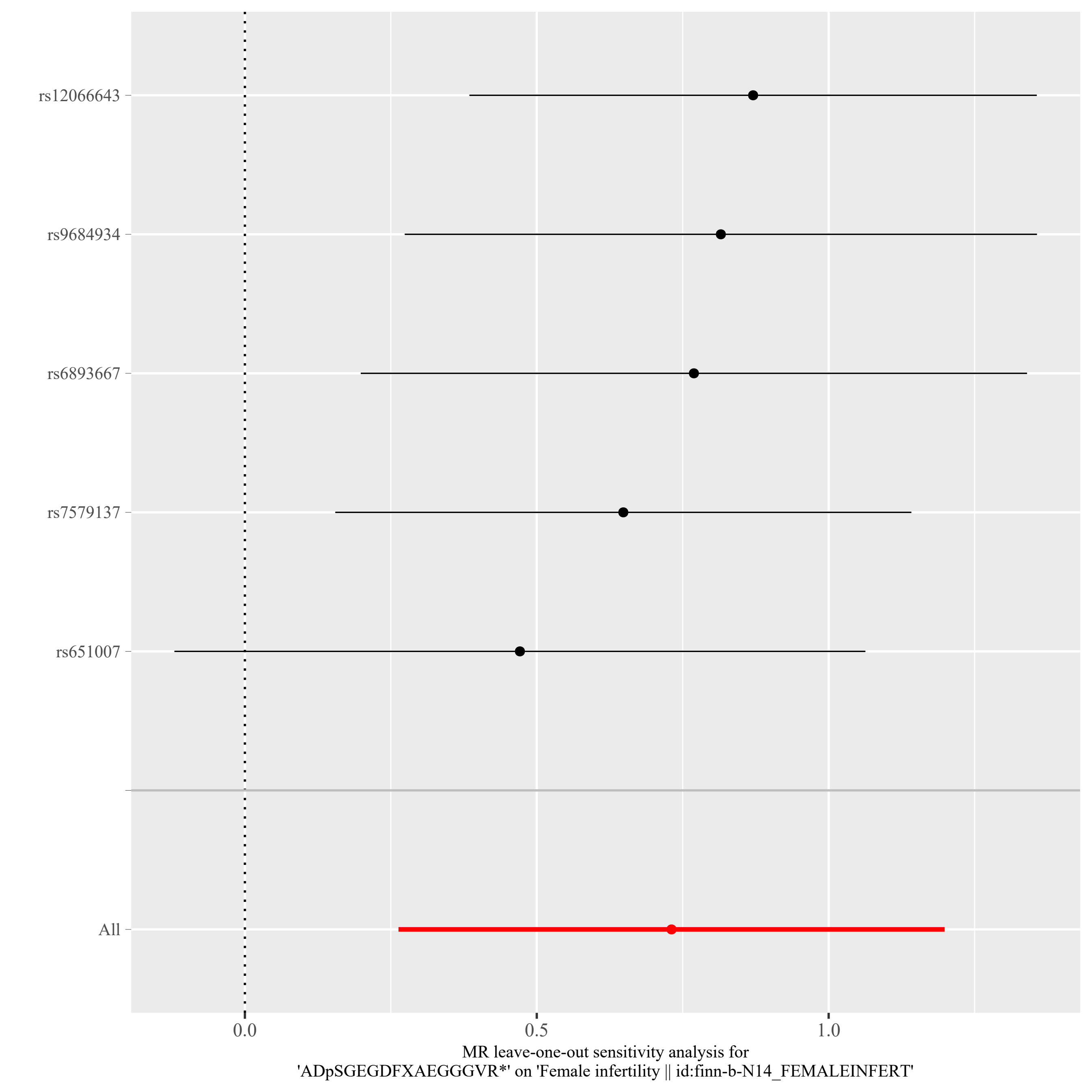
rs12066643
rs9684934
rs6893667
rs7579137
rs651007
All
0.0
0.5
1.0
MR leave-one-out sensitivity analysis for
'ADpSGEGDFXAEGGGVR*' on 'Female infertility || id:finn-b-N14_FEMALEINFERT'

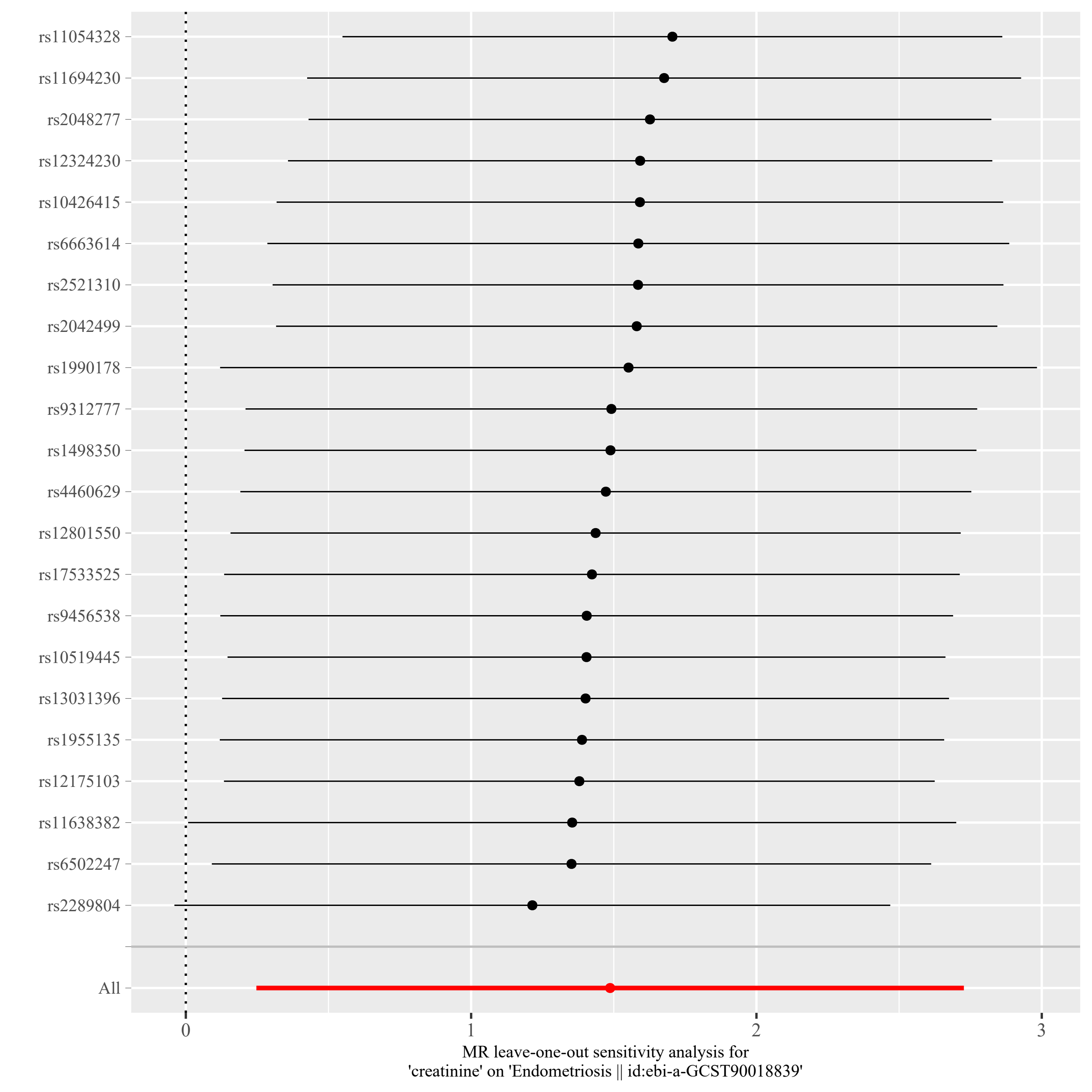
rs11054328
rs11694230
rs2048277
rs12324230
rs10426415
rs6663614
rs2521310
rs2042499
rs1990178
rs9312777
rs1498350
rs4460629
rs12801550
rs17533525
rs9456538
rs10519445
rs13031396
rs1955135
rs12175103
rs11638382
rs6502247
rs2289804
All
0
1
2
3
MR leave-one-out sensitivity analysis for
'creatinine' on 'Endometriosis || id:ebi-a-GCST90018839'

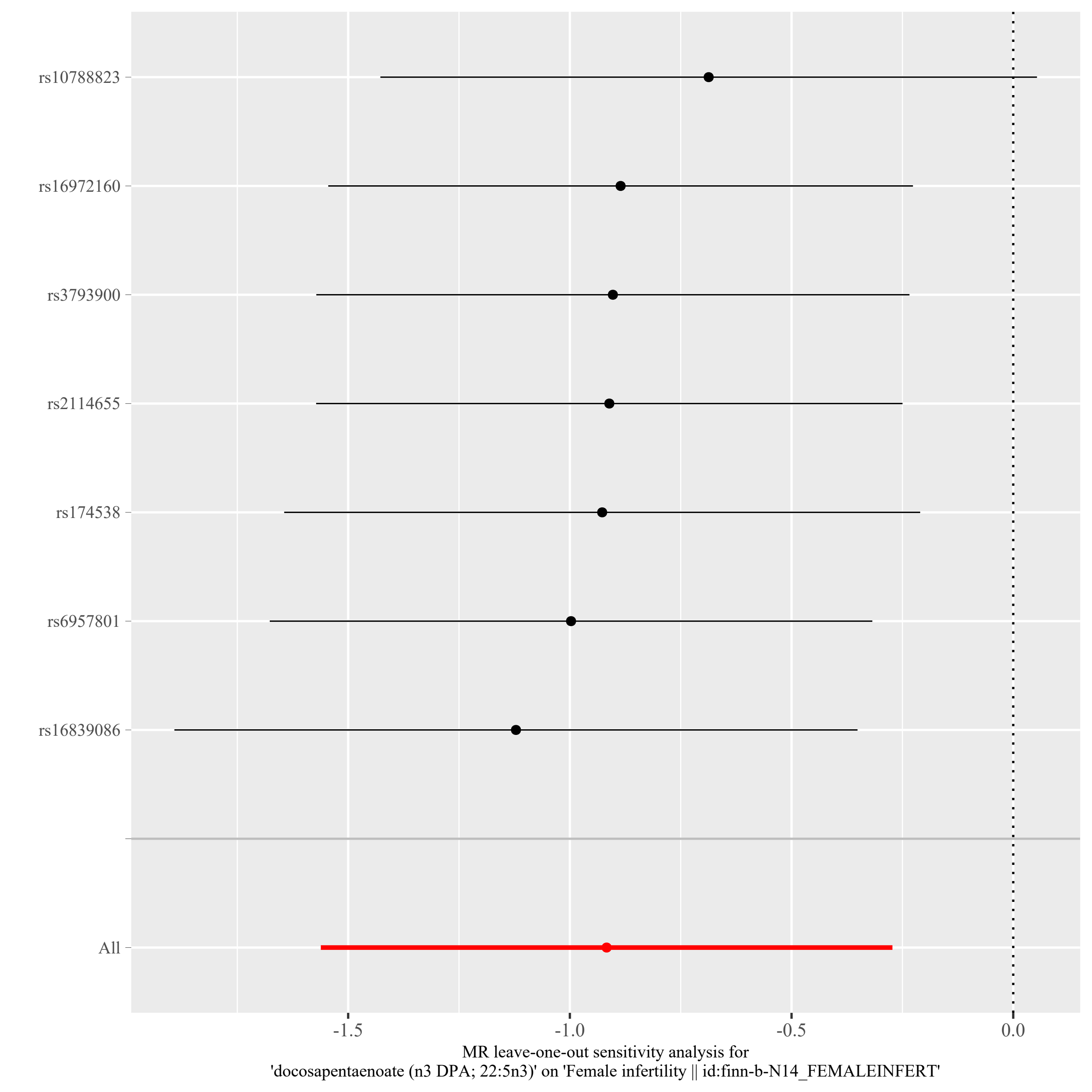
rs10788823
rs16972160
rs3793900
rs2114655
rs174538
rs6957801
rs16839086
All
-1.5
-1.0
-0.5
0.0
MR leave-one-out sensitivity analysis for
'docosapentaenoate (n3 DPA; 22:5n3)' on 'Female infertility || id:finn-b-N14_FEMALEINFERT'

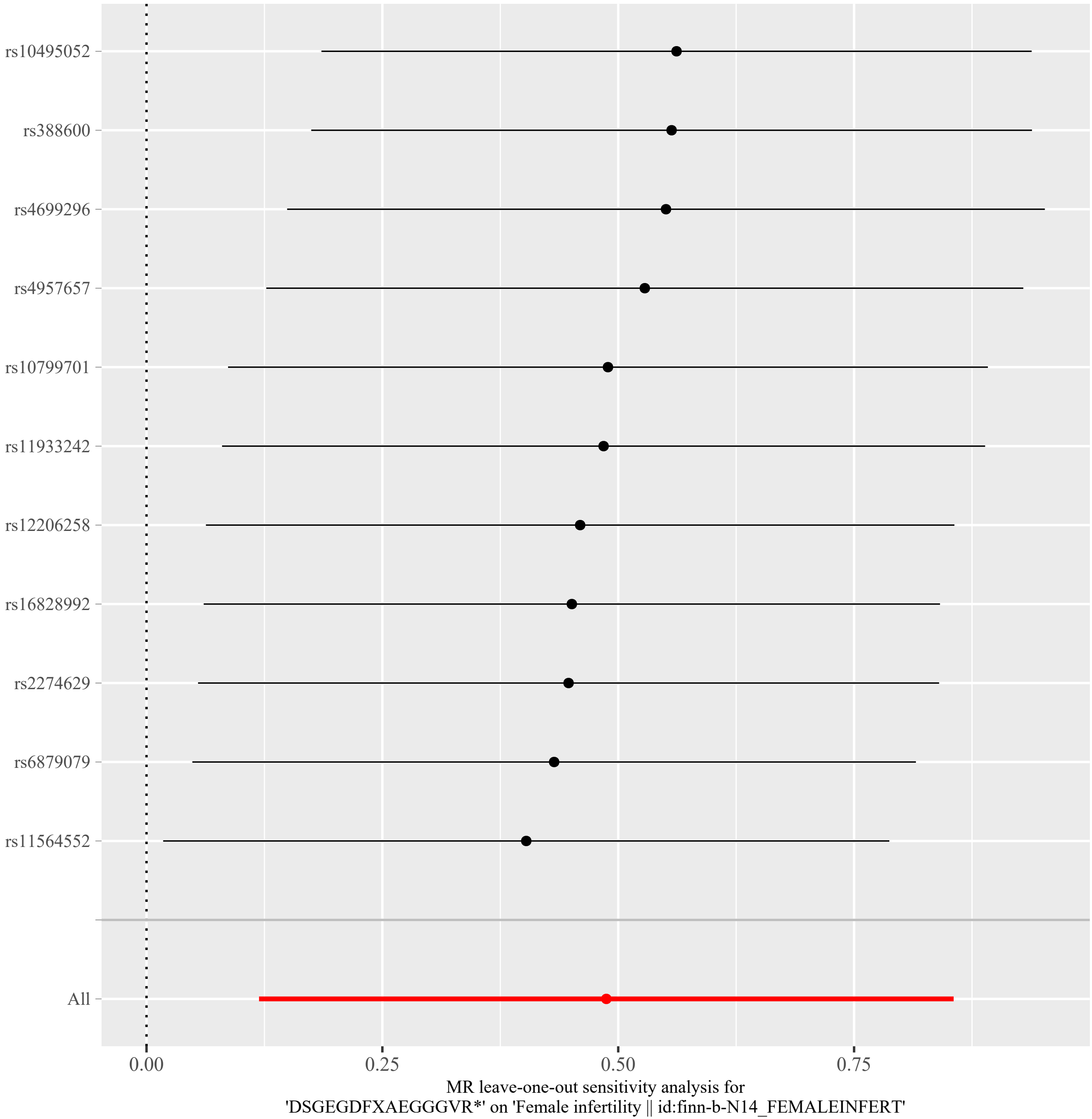
rs10495052
rs388600
rs4699296
rs4957657
rs10799701
rs11933242
rs12206258
rs16828992
rs2274629
rs6879079
rs11564552
All
0.00
0.25
0.50
0.75
MR leave-one-out sensitivity analysis for
'DSGEGDFXAEGGGVR*' on 'Female infertility || id:finn-b-N14_FEMALEINFERT'

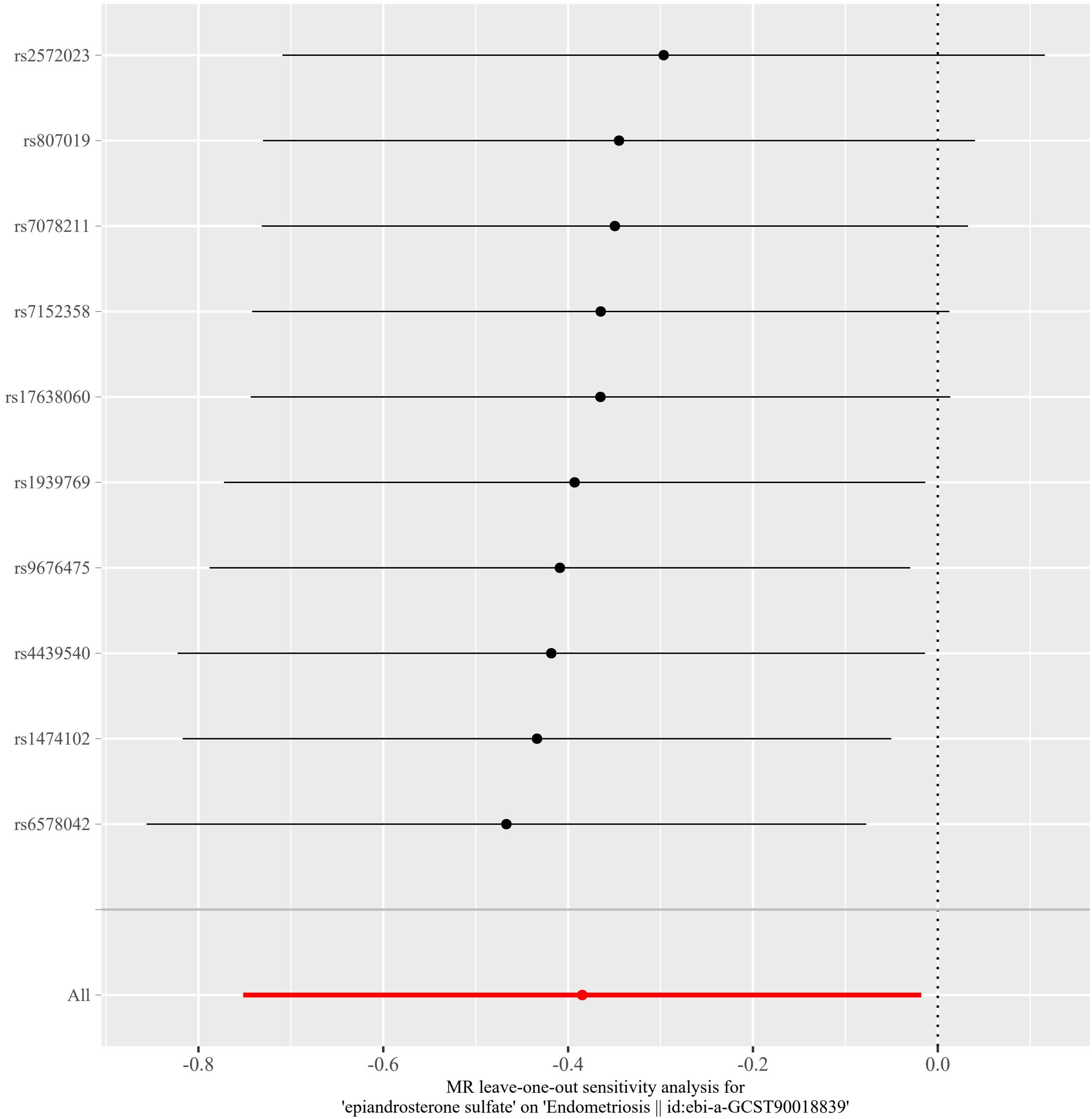
rs2572023
rs807019
rs7078211
rs7152358
rs17638060
rs1939769
rs9676475
rs4439540
rs1474102
rs6578042
All
-0.8
-0.6
-0.4
-0.2
0.0
MR leave-one-out sensitivity analysis for
'epiandrosterone sulfate' on 'Endometriosis || id:ebi-a-GCST90018839'

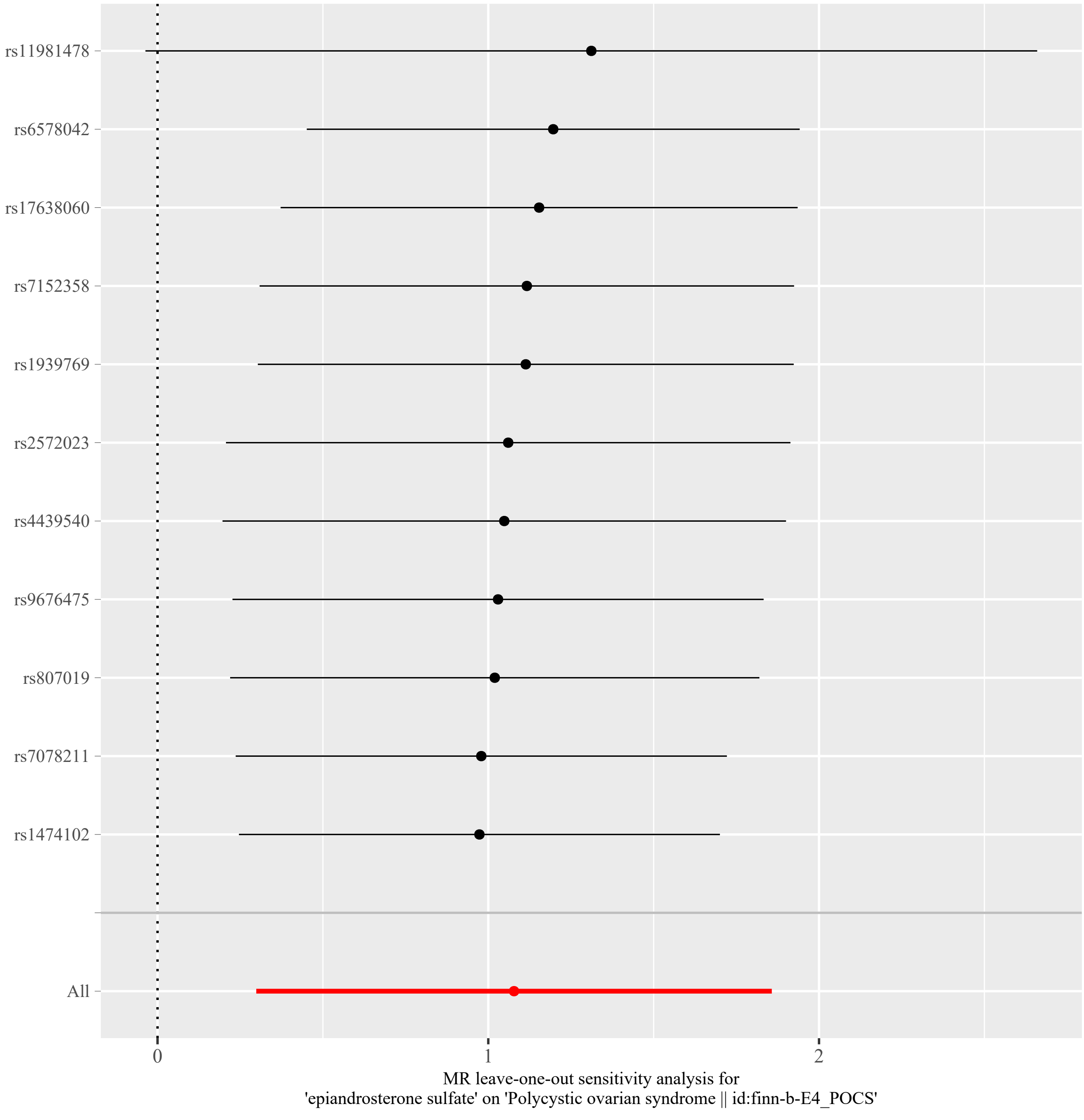
rs11981478
rs6578042
rs17638060
rs7152358
rs1939769
rs2572023
rs4439540
rs9676475
rs807019
rs7078211
rs1474102
All
0
1
2
MR leave-one-out sensitivity analysis for
'epiandrosterone sulfate' on 'Polycystic ovarian syndrome || id:finn-b-E4_POCS'

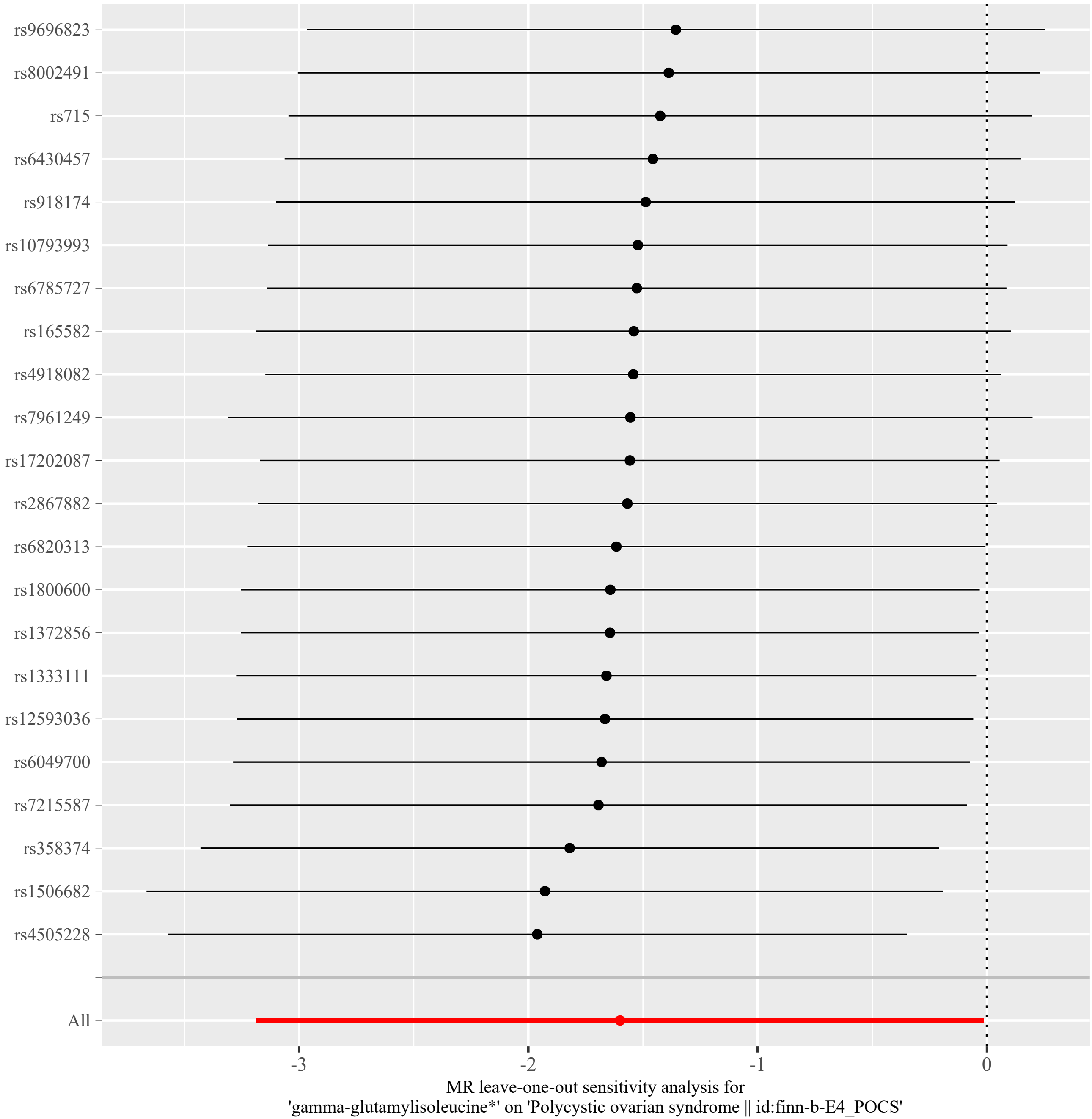
rs9696823
rs8002491
rs715
rs6430457
rs918174
rs10793993
rs6785727
rs165582
rs4918082
rs7961249
rs17202087
rs2867882
rs6820313
rs1800600
rs1372856
rs1333111
rs12593036
rs6049700
rs7215587
rs358374
rs1506682
rs4505228
All
-3
-2
-1
0
MR leave-one-out sensitivity analysis for
'gamma-glutamylisoleucine*' on 'Polycystic ovarian syndrome || id:finn-b-E4_POCS'

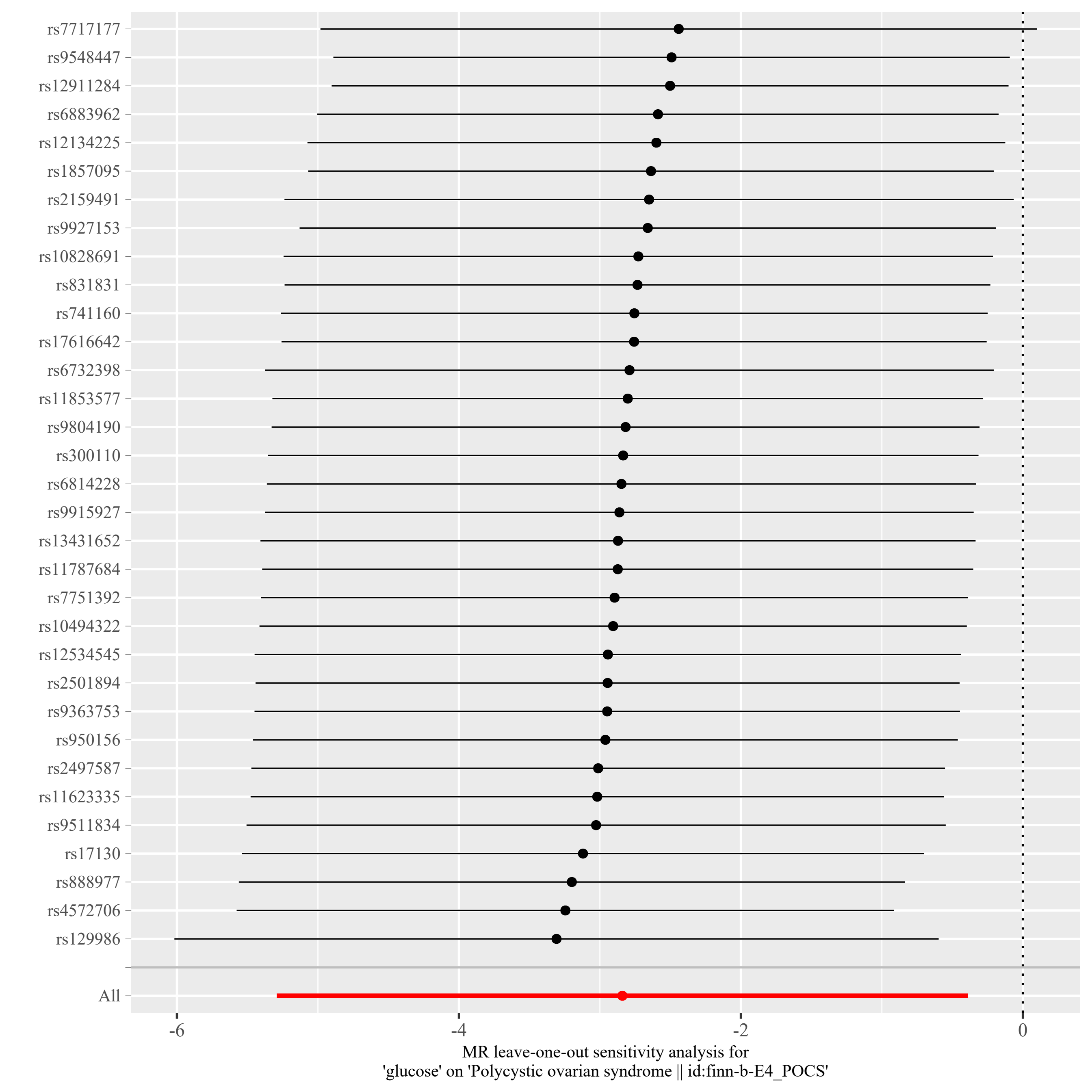
rs7717177
rs9548447
rs12911284
rs6883962
rs12134225
rs1857095
rs2159491
rs9927153
rs10828691
rs831831
rs741160
rs17616642
rs6732398
rs11853577
rs9804190
rs300110
rs6814228
rs9915927
rs13431652
rs11787684
rs7751392
rs10494322
rs12534545
rs2501894
rs9363753
rs950156
rs2497587
rs11623335
rs9511834
rs17130
rs888977
rs4572706
rs129986
All
-6
-4
-2
0
MR leave-one-out sensitivity analysis for
'glucose' on 'Polycystic ovarian syndrome || id:finn-b-E4_POCS'

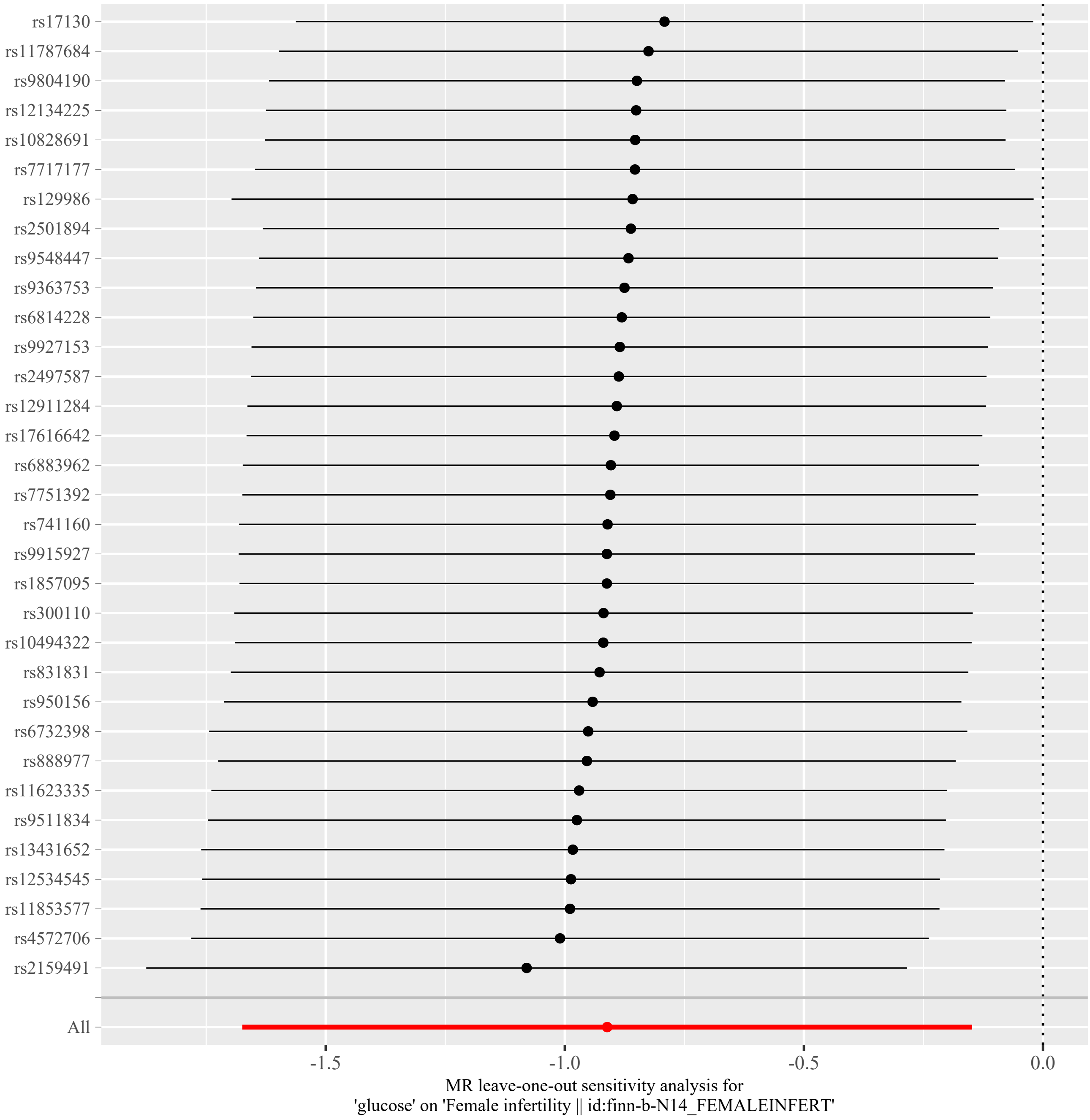

rs17130
rs11787684
rs9804190
rs12134225
rs10828691
rs7717177
rs129986
rs2501894
rs9548447
rs9363753
rs6814228
rs9927153
rs2497587
rs12911284
rs17616642
rs6883962
rs7751392
rs741160
rs9915927
rs1857095
rs300110
rs10494322
rs831831
rs950156
rs6732398
rs888977
rs11623335
rs9511834
rs13431652
rs12534545
rs11853577
rs4572706
rs2159491
All
-1.5
-1.0
-0.5
0.0
MR leave-one-out sensitivity analysis for
'glucose' on 'Female infertility || id:finn-b-N14_FEMALEINFERT'

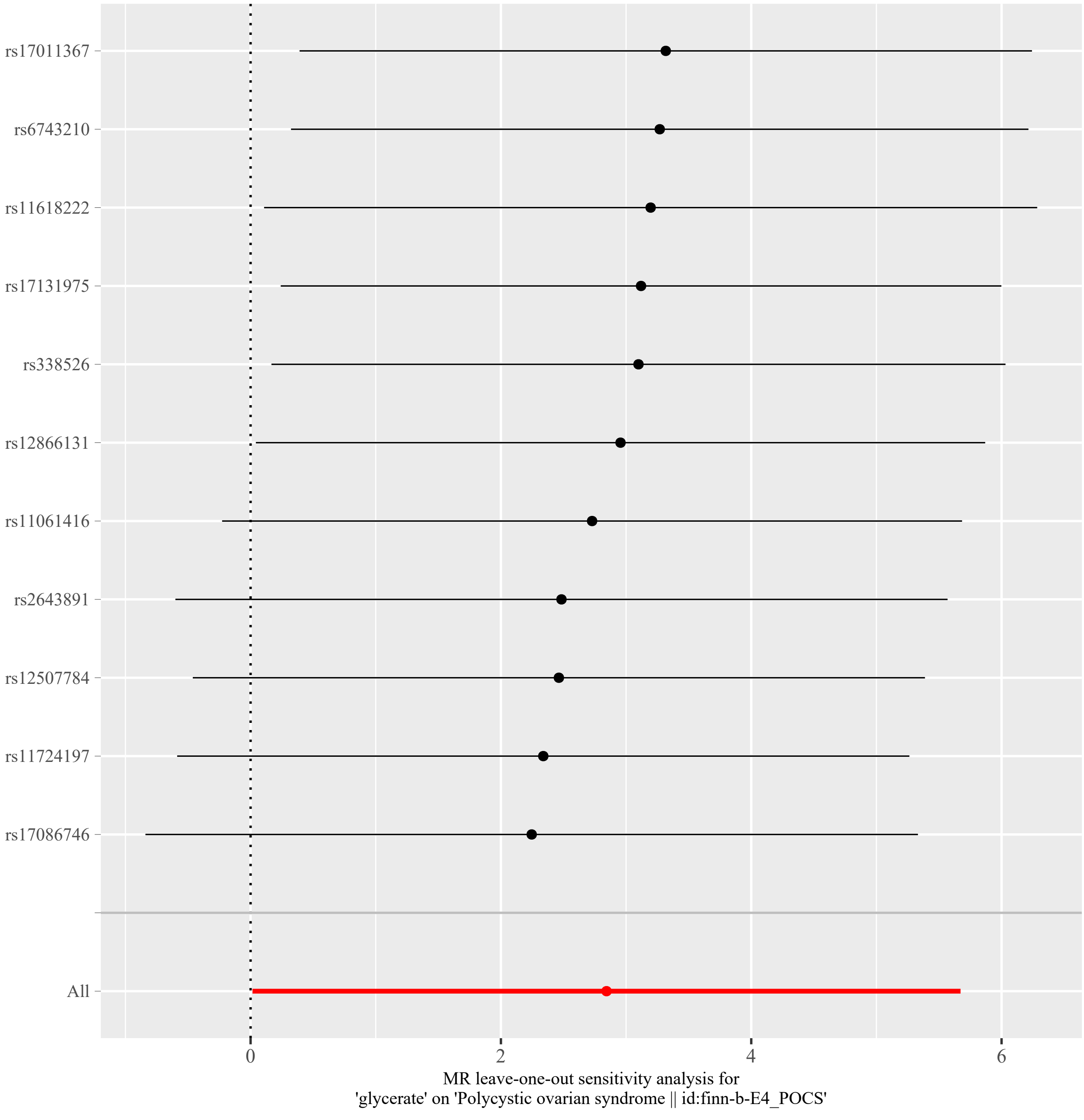
rs17011367
rs6743210
rs11618222
rs17131975
rs338526
rs12866131
rs11061416
rs2643891
rs12507784
rs11724197
rs17086746
All
0
2
4
6
MR leave-one-out sensitivity analysis for
'glycerate' on 'Polycystic ovarian syndrome || id:finn-b-E4_POCS'

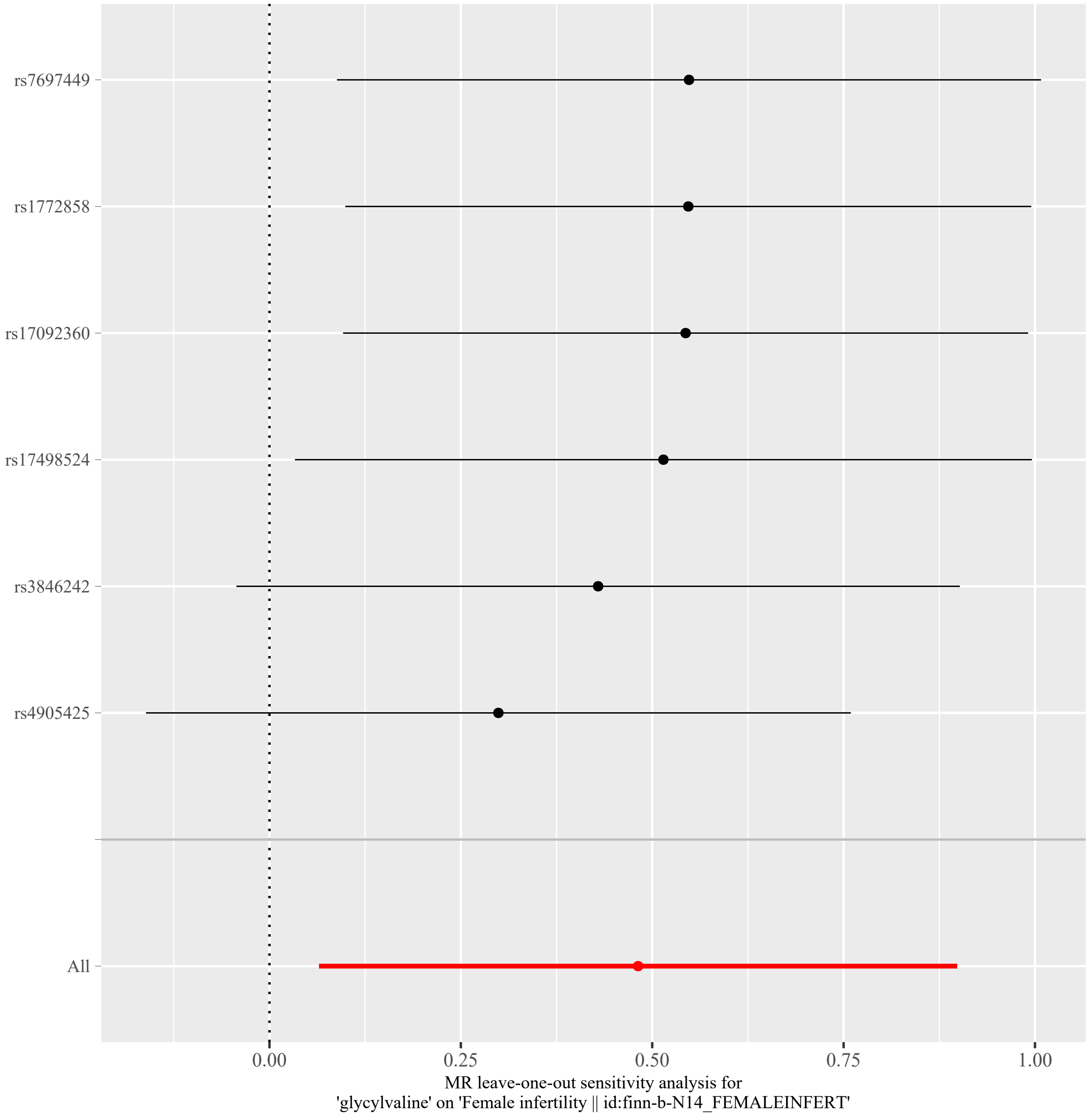
rs7697449
rs1772858
rs17092360
rs17498524
rs3846242
rs4905425
All
0.00
0.25
0.50
0.75
1.00
MR leave-one-out sensitivity analysis for
'glycylvaline' on 'Female infertility || id:finn-b-N14_FEMALEINFERT'

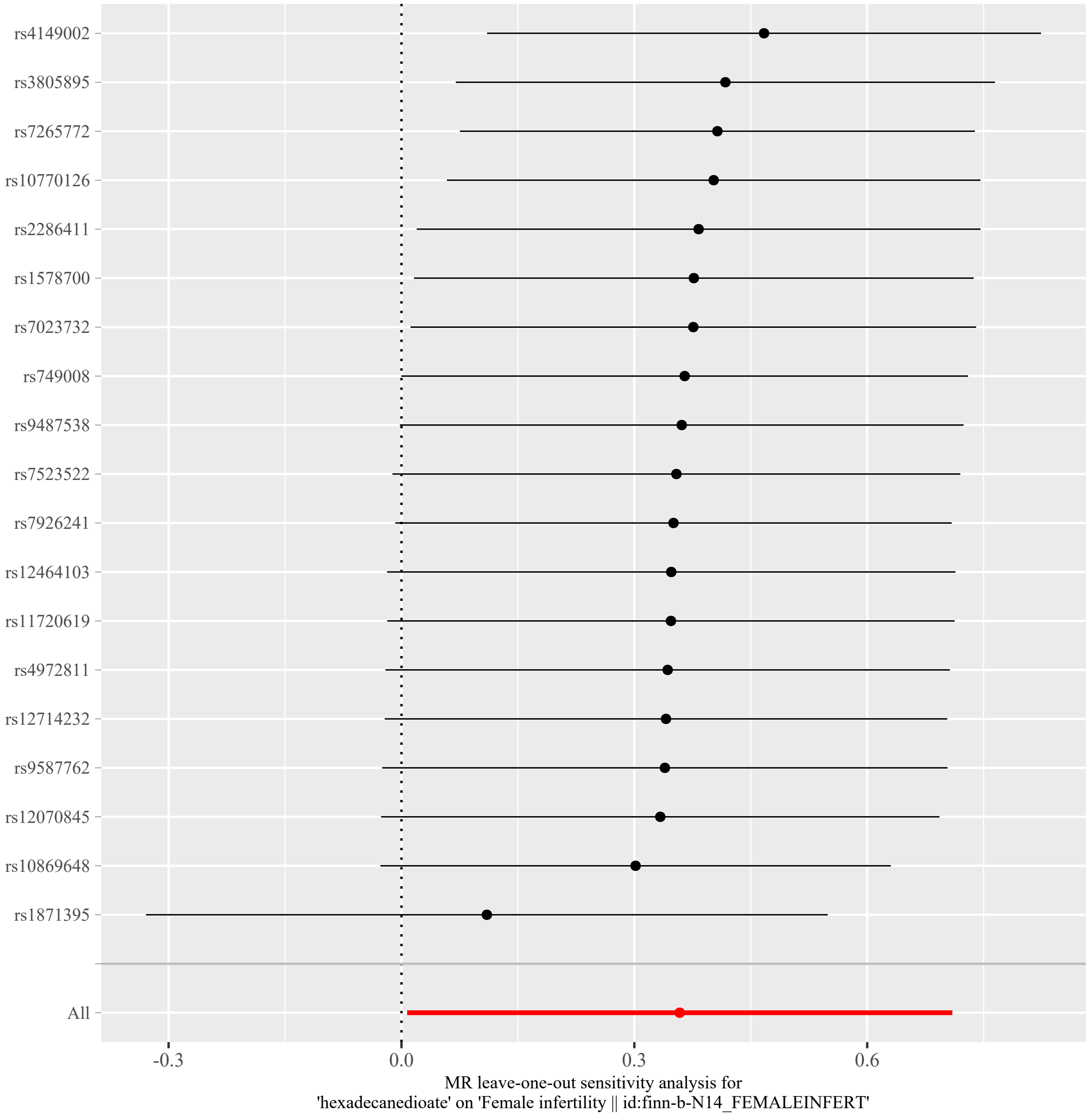
rs4149002
rs3805895
rs7265772
rs10770126
rs2286411
rs1578700
rs7023732
rs749008
rs9487538
rs7523522
rs7926241
rs12464103
rs11720619
rs4972811
rs12714232
rs9587762
rs12070845
rs10869648
rs1871395
All
-0.3
0.0
0.3
0.6
MR leave-one-out sensitivity analysis for
'hexadecanedioate' on 'Female infertility || id:finn-b-N14_FEMALEINFERT'

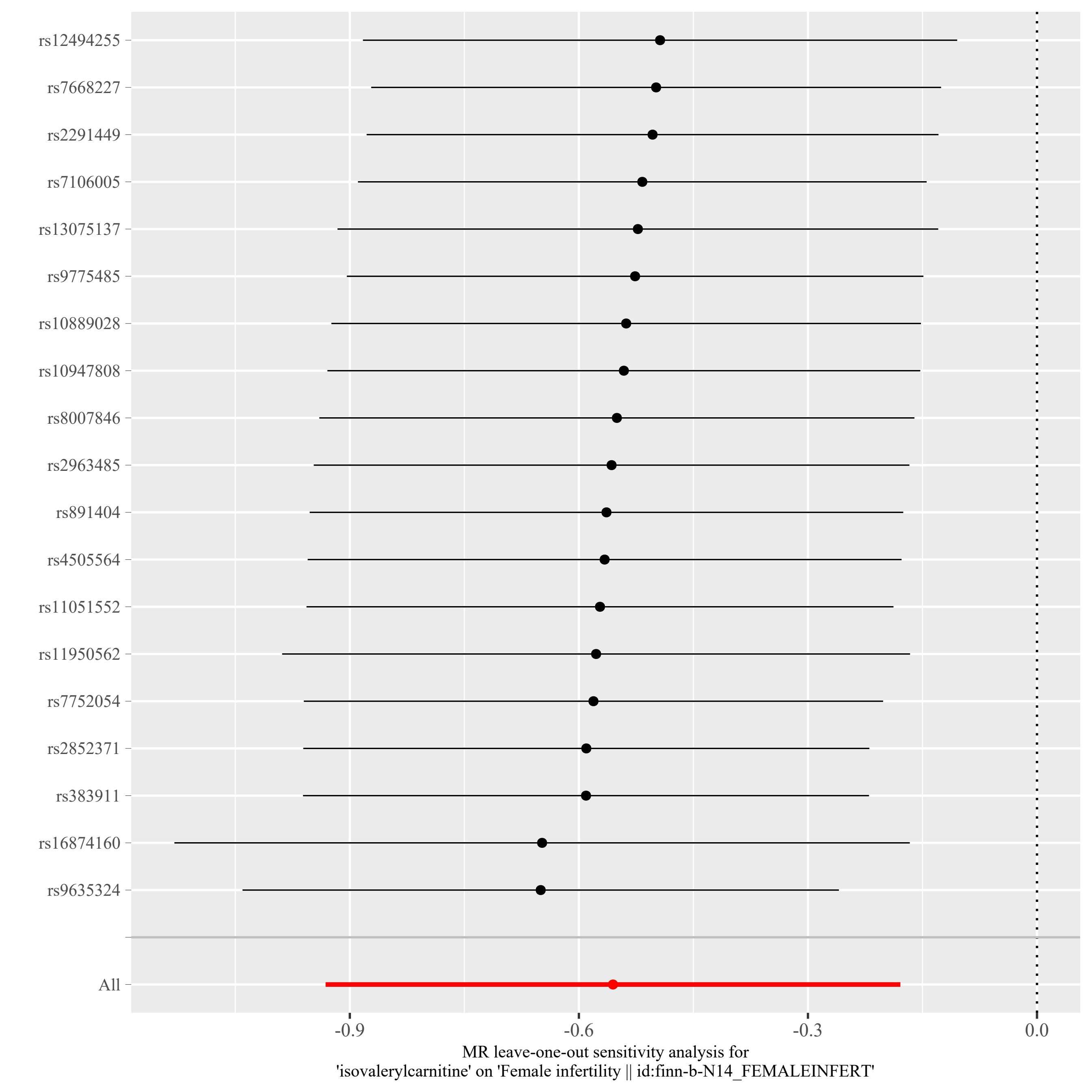
rs12494255
rs7668227
rs2291449
rs7106005
rs13075137
rs9775485
rs10889028
rs10947808
rs8007846
rs2963485
rs891404
rs4505564
rs11051552
rs11950562
rs7752054
rs2852371
rs383911
rs16874160
rs9635324
All
-0.9
-0.6
-0.3
0.0
MR leave-one-out sensitivity analysis for
'isovalerylcarnitine' on 'Female infertility || id:finn-b-N14_FEMALEINFERT'

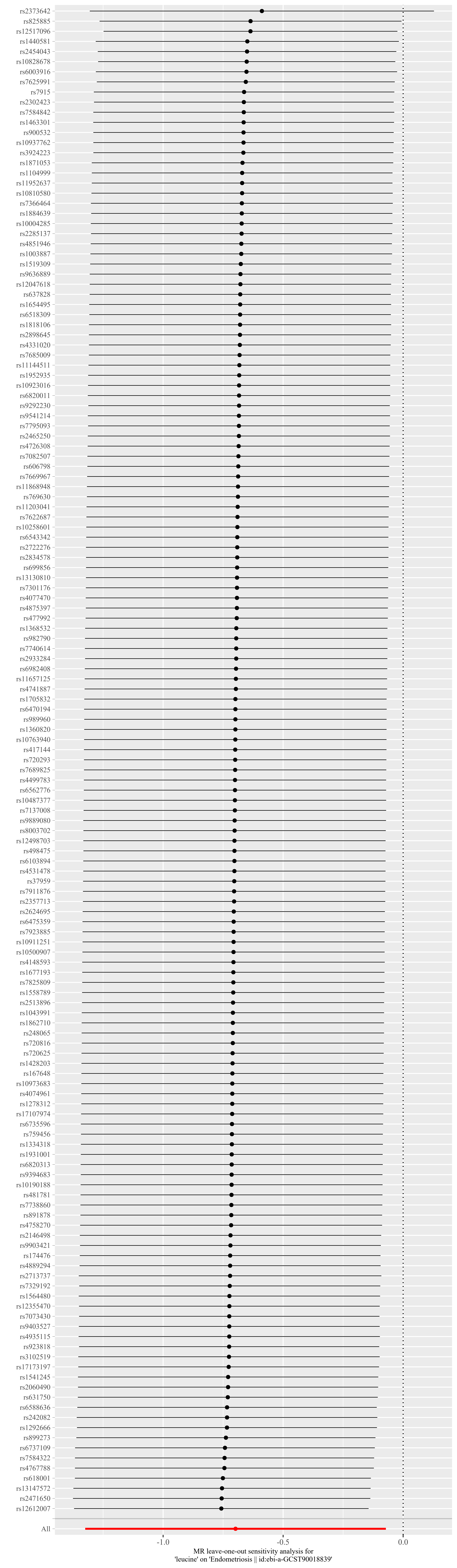
rs2373642
rs825885
rs12517096
rs1440581
rs2454043
rs10828678
rs6003916
rs7625991
rs7915
rs2302423
rs7584842
rs1463301
rs900532
rs10937762
rs3924223
rs1871053
rs1104999
rs11952637
rs10810580
rs7366464
rs1884639
rs10004285
rs2285137
rs4851946
rs1003887
rs1519309
rs9636889
rs12047618
rs637828
rs1654495
rs6518309
rs1818106
rs2898645
rs4331020
rs7685009
rs11144511
rs1952935
rs10923016
rs6820011
rs9292230
rs9541214
rs7795093
rs2465250
rs4726308
rs7082507
rs606798
rs7669967
rs11868948
rs769630
rs11203041
rs7622687
rs10258601
rs6543342
rs2722276
rs2834578
rs699856
rs13130810
rs7301176
rs4077470
rs4875397
rs477992
rs1368532
rs982790
rs7740614
rs2933284
rs6982408
rs11657125
rs4741887
rs1705832
rs6470194
rs989960
rs1360820
rs10763940
rs417144
rs720293
rs7689825
rs4499783
rs6562776
rs10487377
rs7137008
rs9889080
rs8003702
rs12498703
rs498475
rs6103894
rs4531478
rs37959
rs7911876
rs2357713
rs2624695
rs6475359
rs7923885
rs10911251
rs10500907
rs4148593
rs1677193
rs7825809
rs1558789
rs2513896
rs1043991
rs1862710
rs248065
rs720816
rs720625
rs1428203
rs167648
rs10973683
rs4074961
rs1278312
rs17107974
rs6735596
rs759456
rs1334318
rs1931001
rs6820313
rs9394683
rs10190188
rs481781
rs7738860
rs891878
rs4758270
rs2146498
rs9903421
rs174476
rs4889294
rs2713737
rs7329192
rs1564480
rs12355470
rs7073430
rs9403527
rs4935115
rs923818
rs3102519
rs17173197
rs1541245
rs2060490
rs631750
rs6588636
rs242082
rs1292666
rs899273
rs6737109
rs7584322
rs4767788
rs618001
rs13147572
rs2471650
rs12612007
All
-1.0
-0.5
0.0
MR leave-one-out sensitivity analysis for
'leucine' on 'Endometriosis || id:ebi-a-GCST90018839'

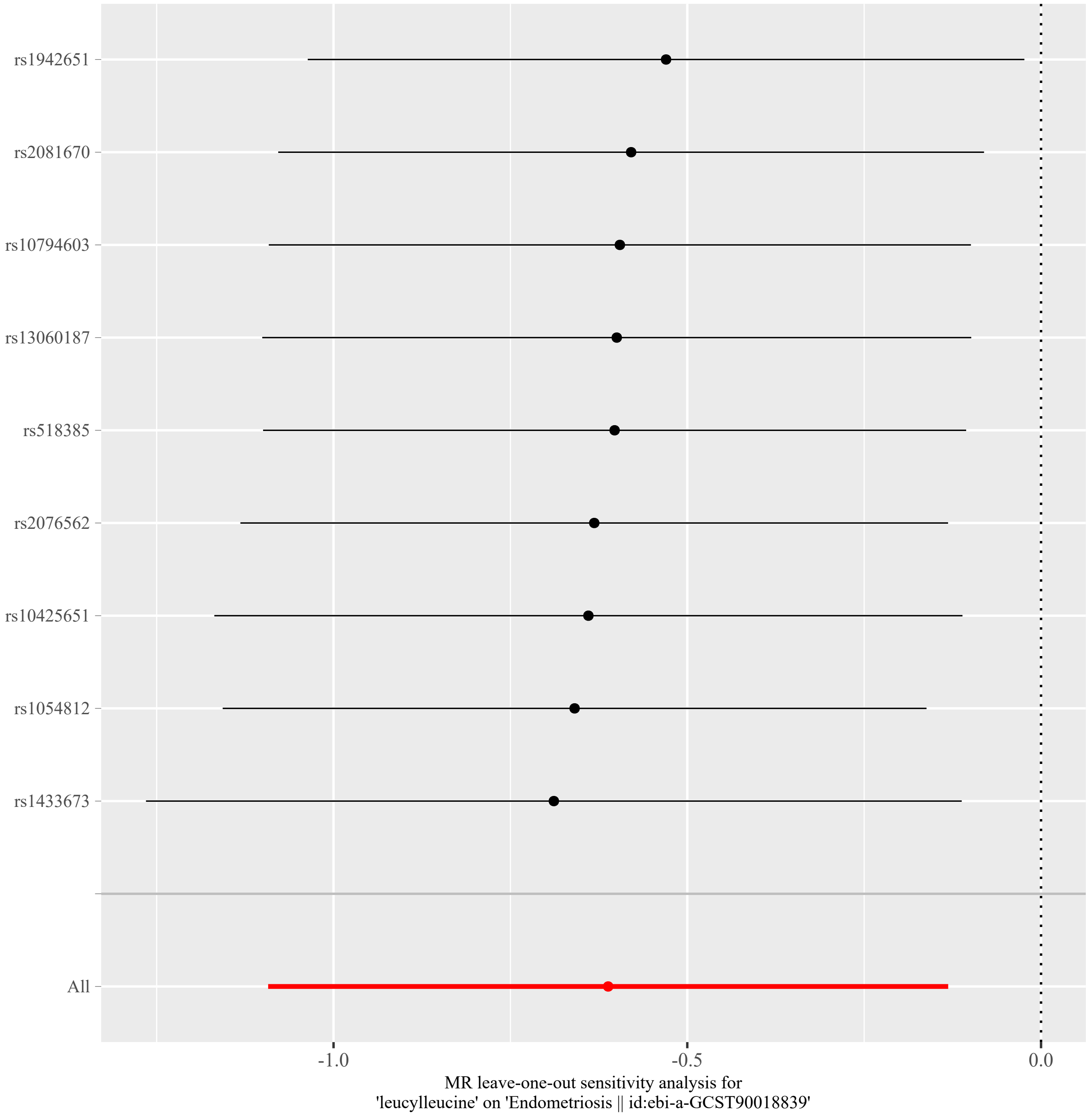

rs1942651
rs2081670
rs10794603
rs13060187
rs518385
rs2076562
rs10425651
rs1054812
rs1433673
All
-1.0
-0.5
0.0
MR leave-one-out sensitivity analysis for
'leucylleucine' on 'Endometriosis || id:ebi-a-GCST90018839'

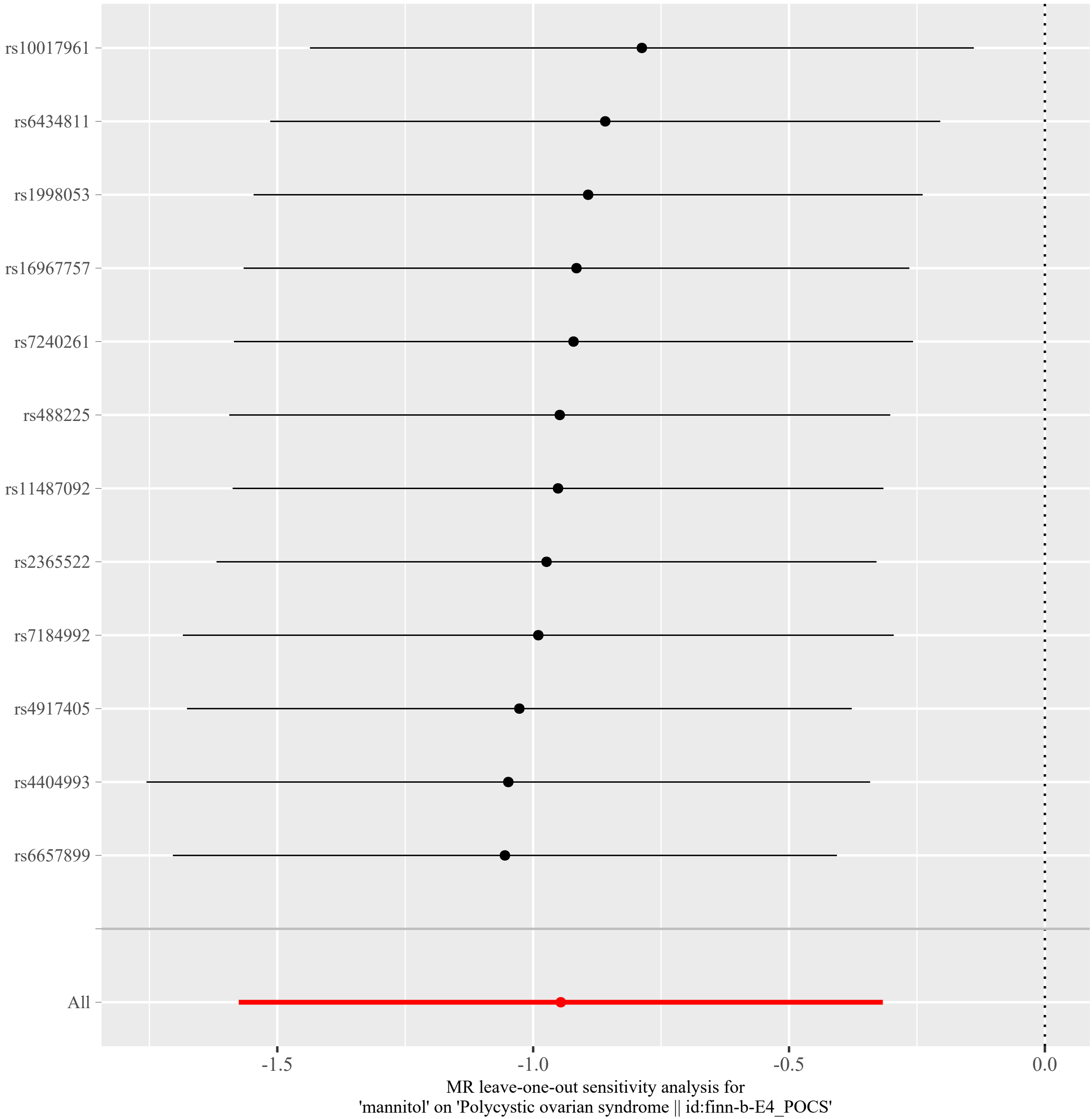
rs10017961
rs6434811
rs1998053
rs16967757
rs7240261
rs488225
rs11487092
rs2365522
rs7184992
rs4917405
rs4404993
rs6657899
All
-1.5
-1.0
-0.5
0.0
MR leave-one-out sensitivity analysis for
'mannitol' on 'Polycystic ovarian syndrome || id:finn-b-E4_POCS'

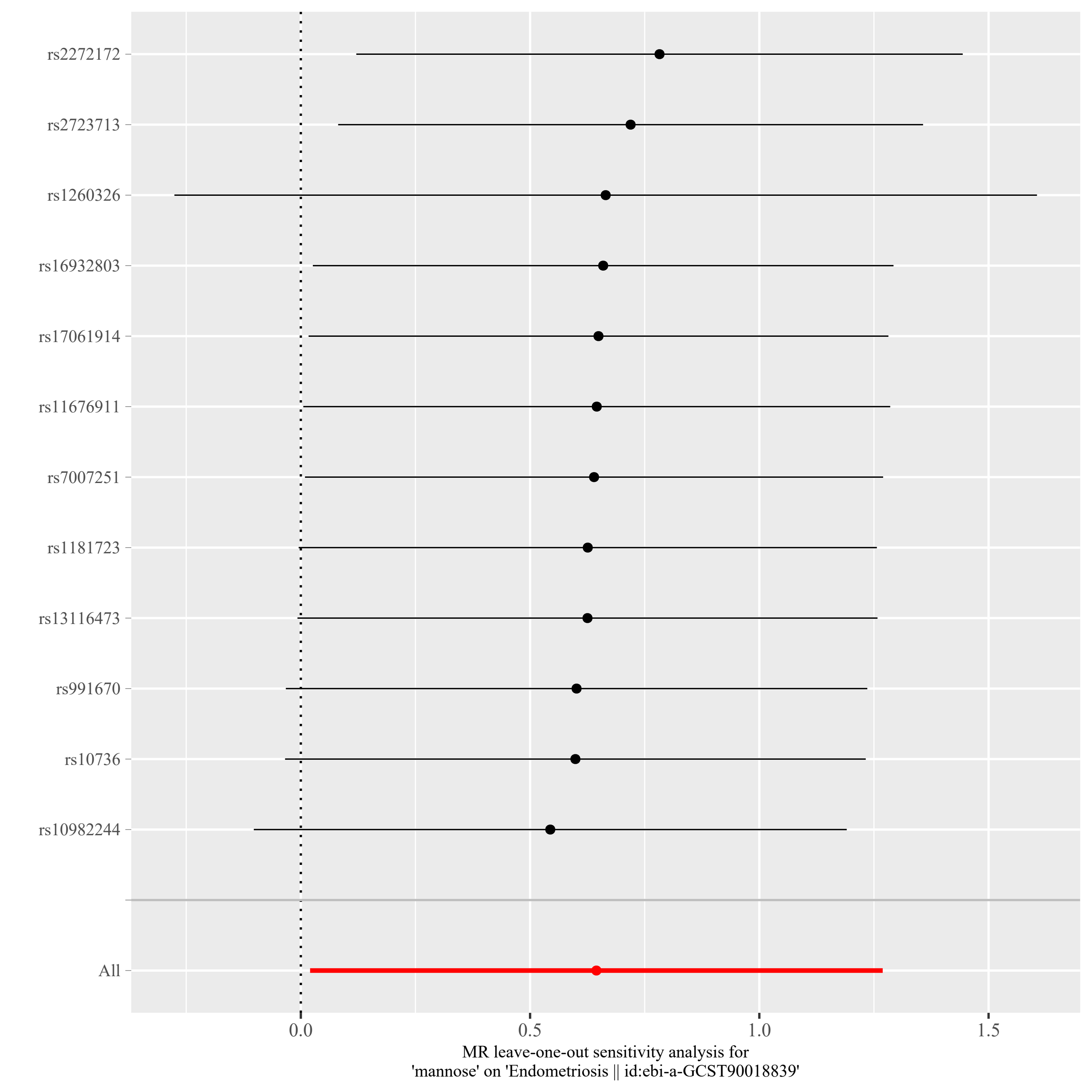
rs2272172
rs2723713
rs1260326
rs16932803
rs17061914
rs11676911
rs7007251
rs1181723
rs13116473
rs991670
rs10736
rs10982244
All
0.0
0.5
1.0
1.5
MR leave-one-out sensitivity analysis for
'mannose' on 'Endometriosis || id:ebi-a-GCST90018839'

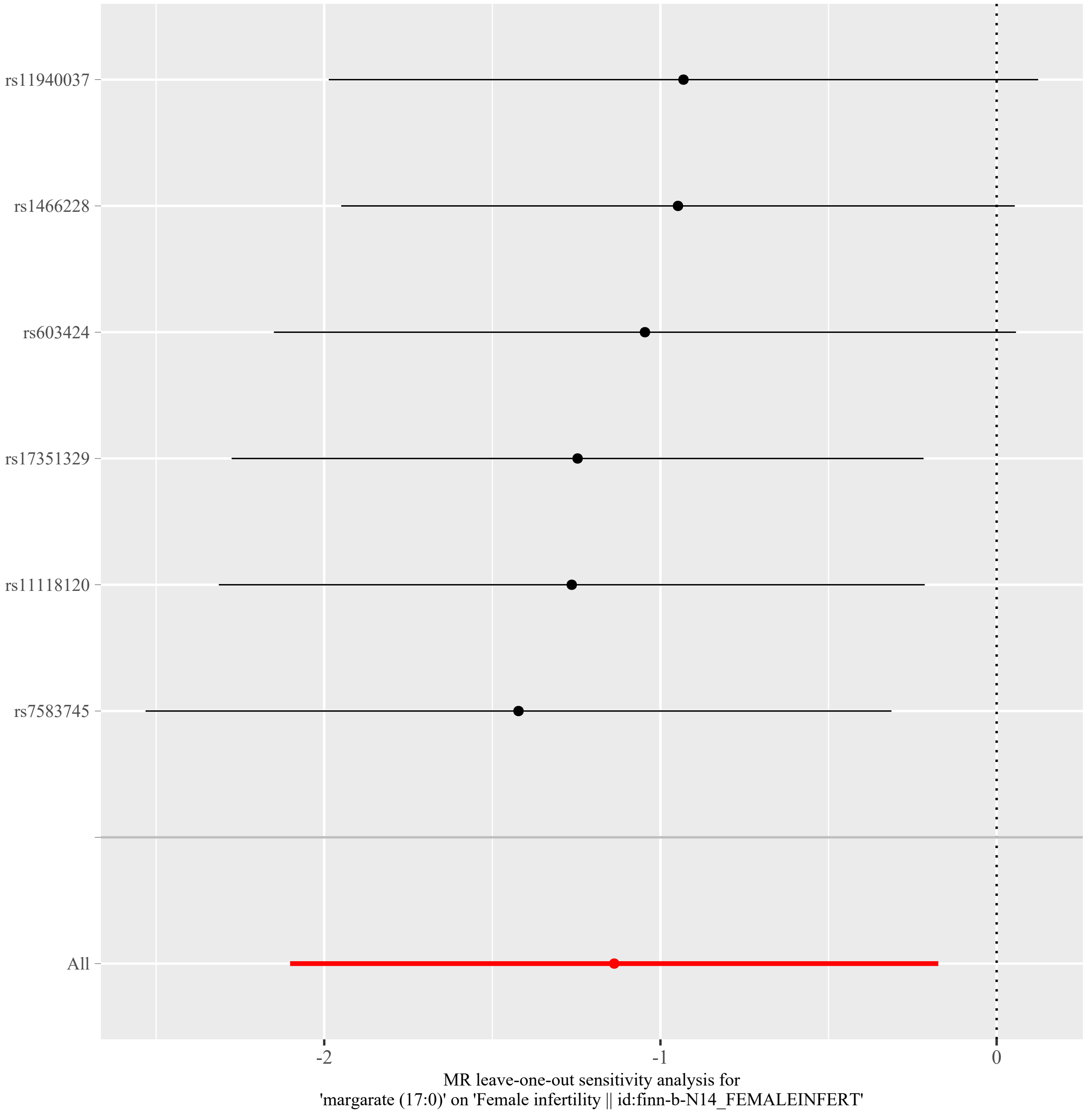
rs11940037
rs1466228
rs603424
rs17351329
rs11118120
rs7583745
All
-2
-1
0
MR leave-one-out sensitivity analysis for
'margarate (17:0)' on 'Female infertility || id:finn-b-N14_FEMALEINFERT'

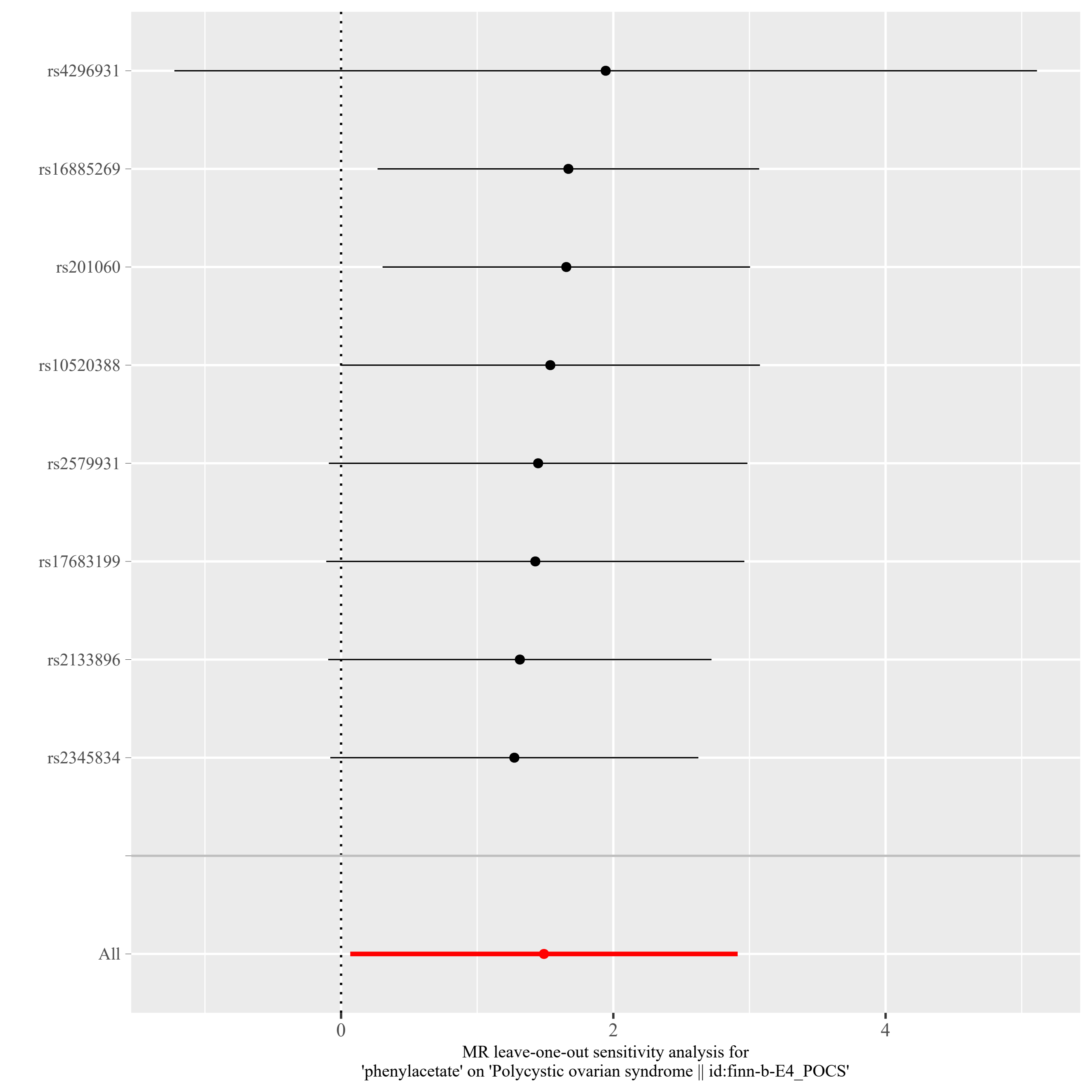
rs4296931
rs16885269
rs201060
rs10520388
rs2579931
rs17683199
rs2133896
rs2345834
All
0
2
4
MR leave-one-out sensitivity analysis for
'phenylacetate' on 'Polycystic ovarian syndrome || id:finn-b-E4_POCS'

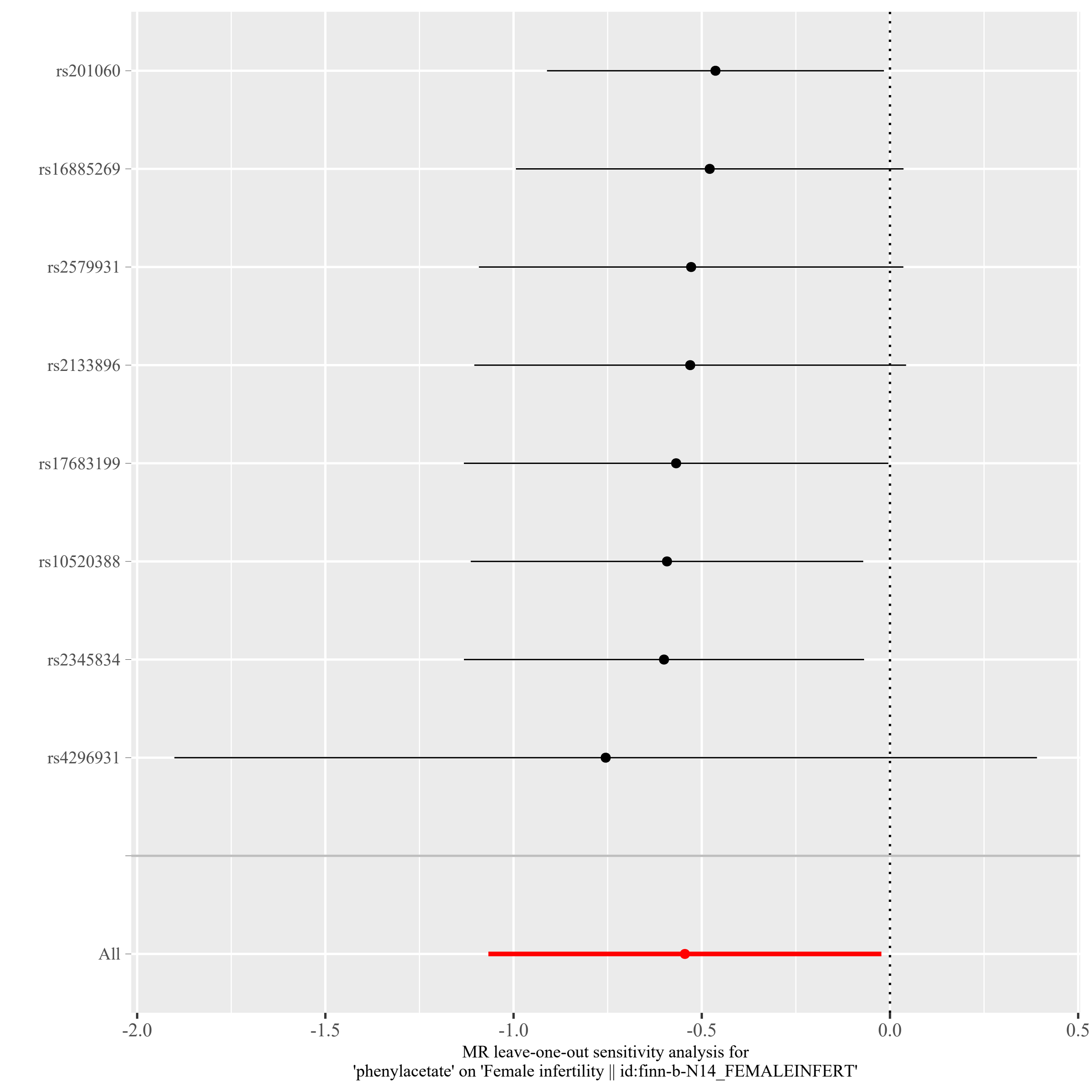

rs201060
rs16885269
rs2579931
rs2133896
rs17683199
rs10520388
rs2345834
rs4296931
All
-2.0
-1.5
-1.0
-0.5
0.0
0.5
MR leave-one-out sensitivity analysis for
'phenylacetate' on 'Female infertility || id:finn-b-N14_FEMALEINFERT'

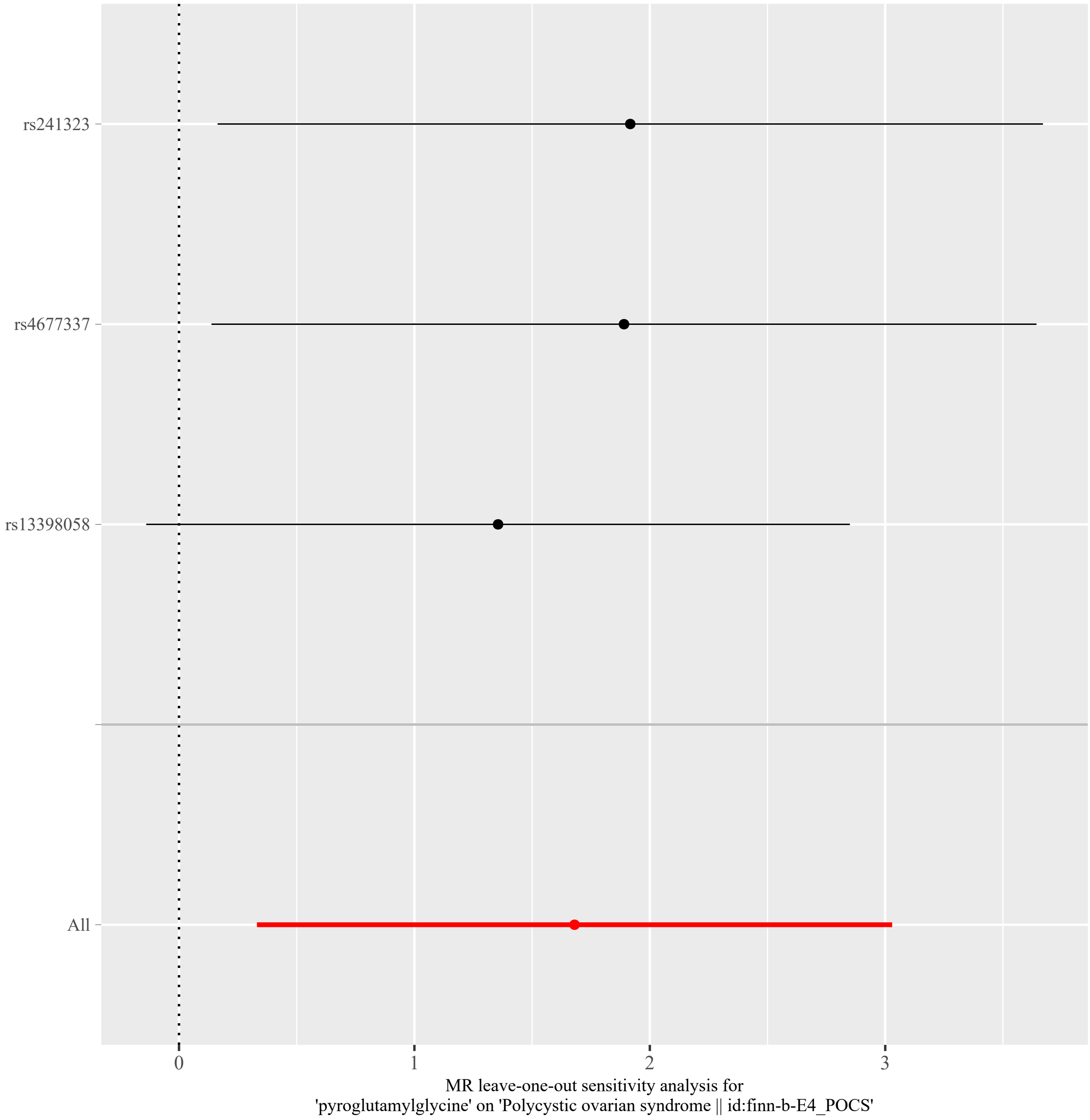
rs241323
rs4677337
rs13398058
All
0
1
2
3
MR leave-one-out sensitivity analysis for
'pyroglutamylglycine' on 'Polycystic ovarian syndrome || id:finn-b-E4_POCS'

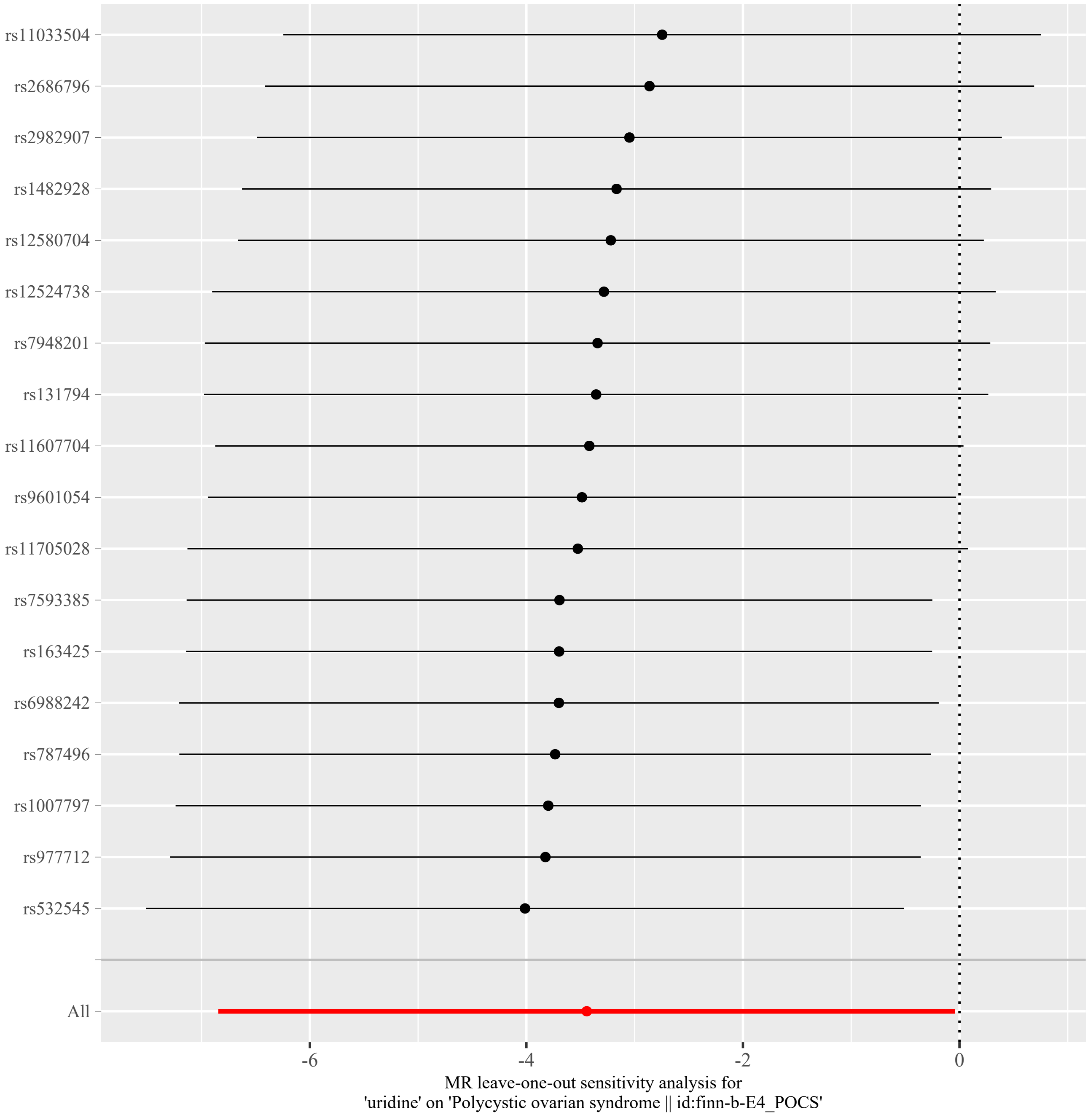
rs11033504
rs2686796
rs2982907
rs1482928
rs12580704
rs12524738
rs7948201
rs131794
rs11607704
rs9601054
rs11705028
rs7593385
rs163425
rs6988242
rs787496
rs1007797
rs977712
rs532545
All
-6
-4
-2
0
MR leave-one-out sensitivity analysis for
'uridine' on 'Polycystic ovarian syndrome || id:finn-b-E4_POCS'

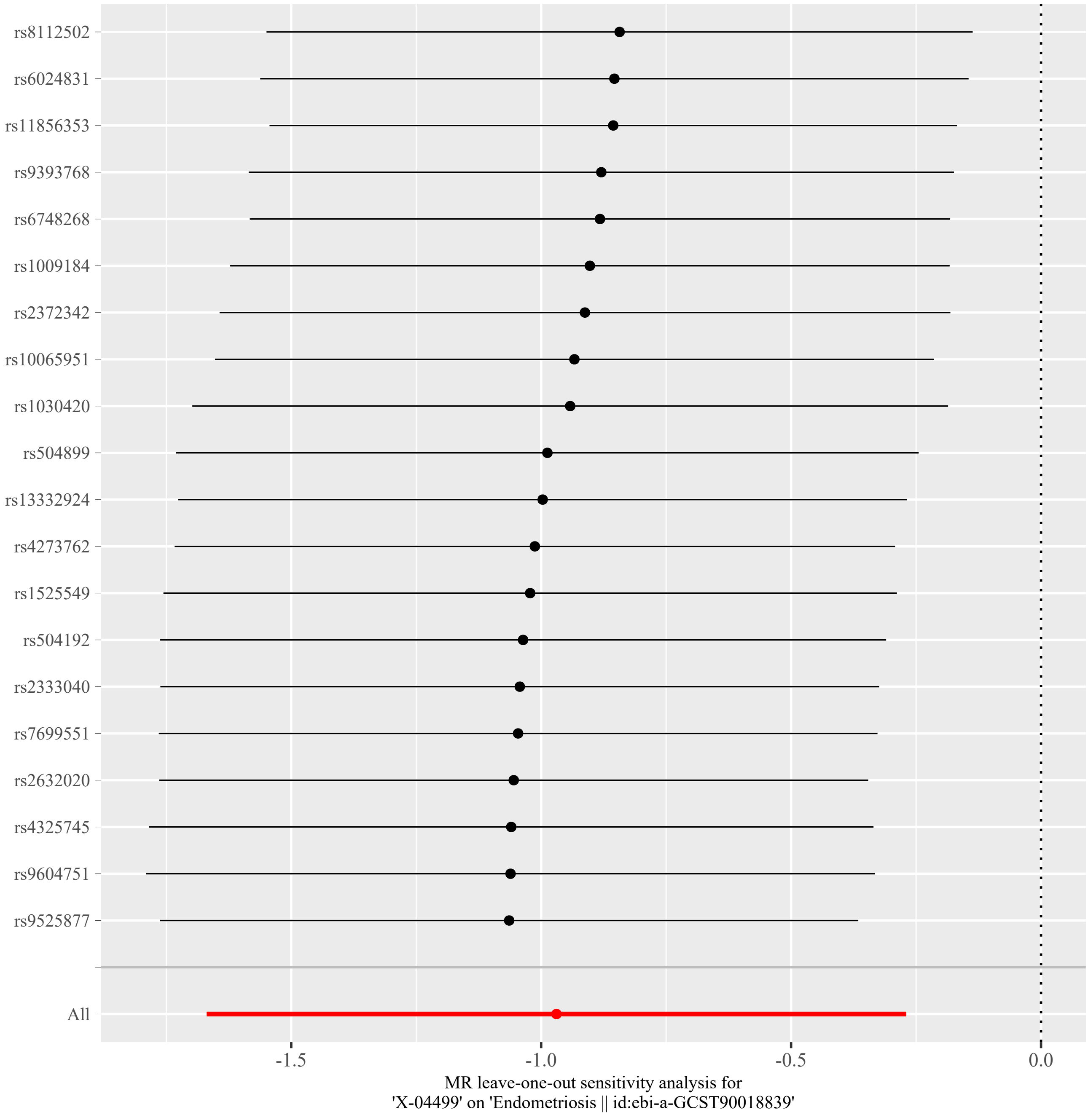
rs8112502
rs6024831
rs11856353
rs9393768
rs6748268
rs1009184
rs2372342
rs10065951
rs1030420
rs504899
rs13332924
rs4273762
rs1525549
rs504192
rs2333040
rs7699551
rs2632020
rs4325745
rs9604751
rs9525877
All
-1.5
-1.0
-0.5
0.0
MR leave-one-out sensitivity analysis for
'X-04499' on 'Endometriosis || id:ebi-a-GCST90018839'

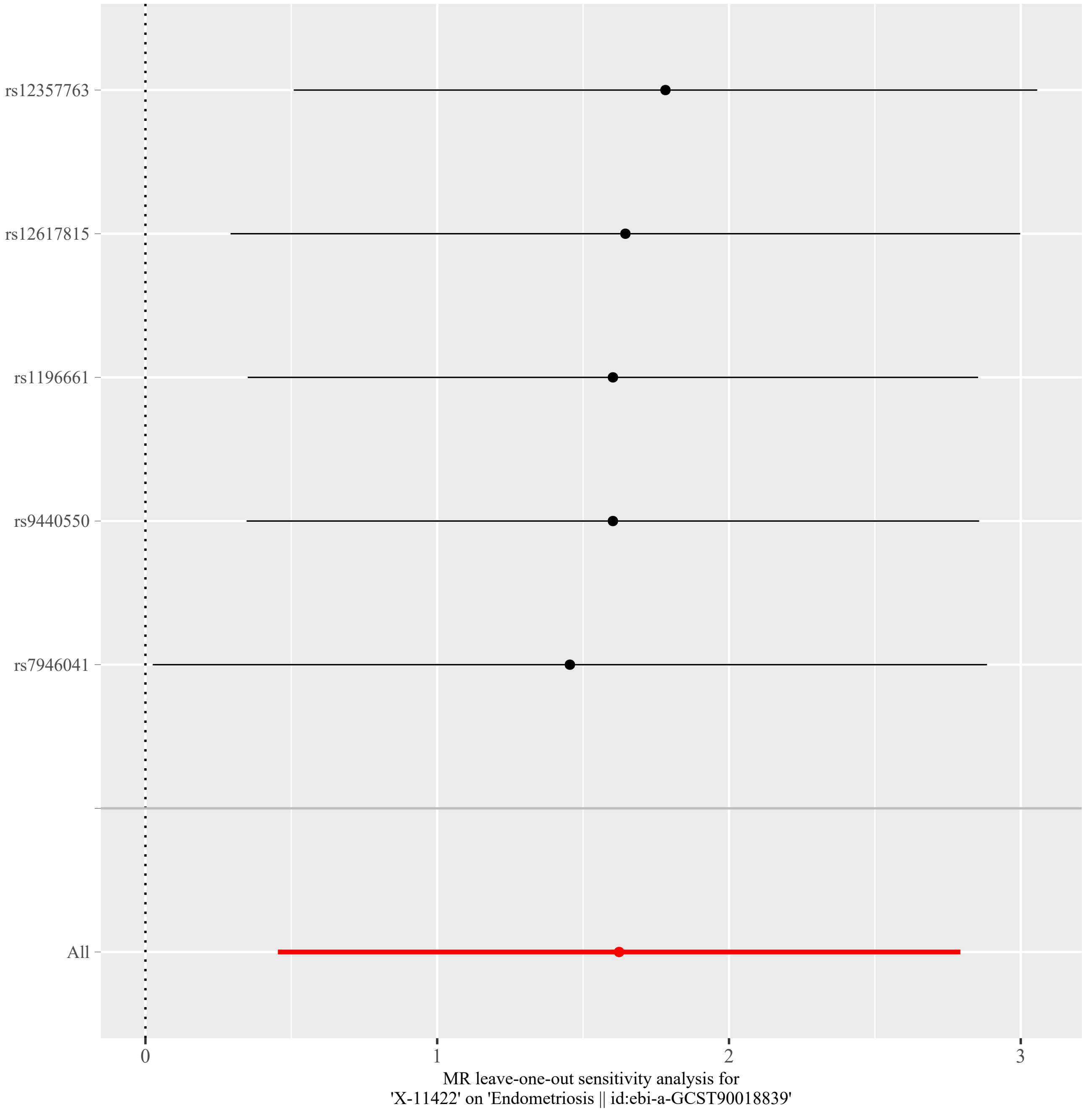
rs12357763
rs12617815
rs1196661
rs9440550
rs7946041
All
0
1
2
3
MR leave-one-out sensitivity analysis for
'X-11422' on 'Endometriosis || id:ebi-a-GCST90018839'

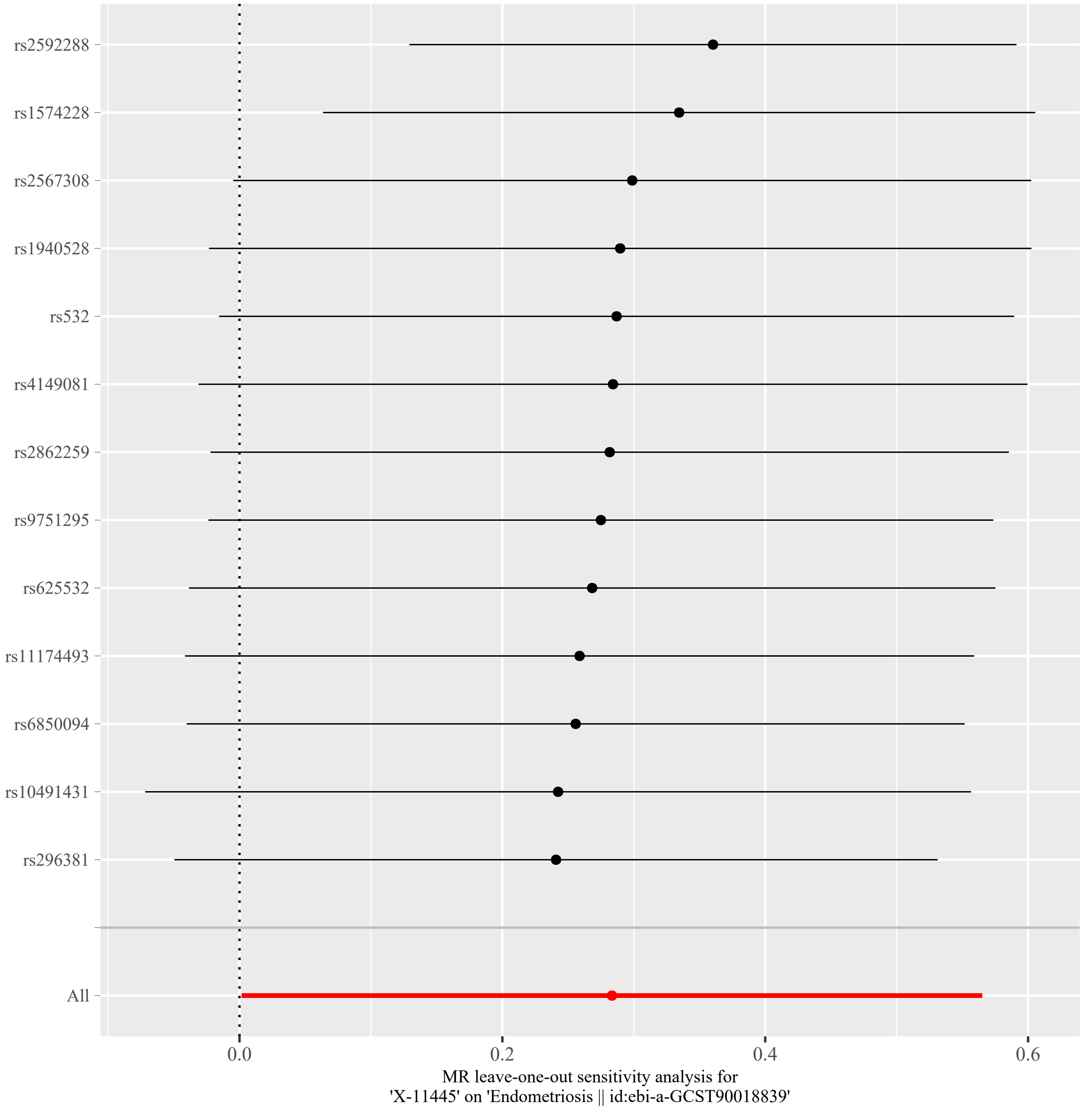

rs2592288
rs1574228
rs2567308
rs1940528
rs532
rs4149081
rs2862259
rs9751295
rs625532
rs11174493
rs6850094
rs10491431
rs296381
All
0.0
0.2
0.4
0.6
MR leave-one-out sensitivity analysis for
'X-11445' on 'Endometriosis || id:ebi-a-GCST90018839'

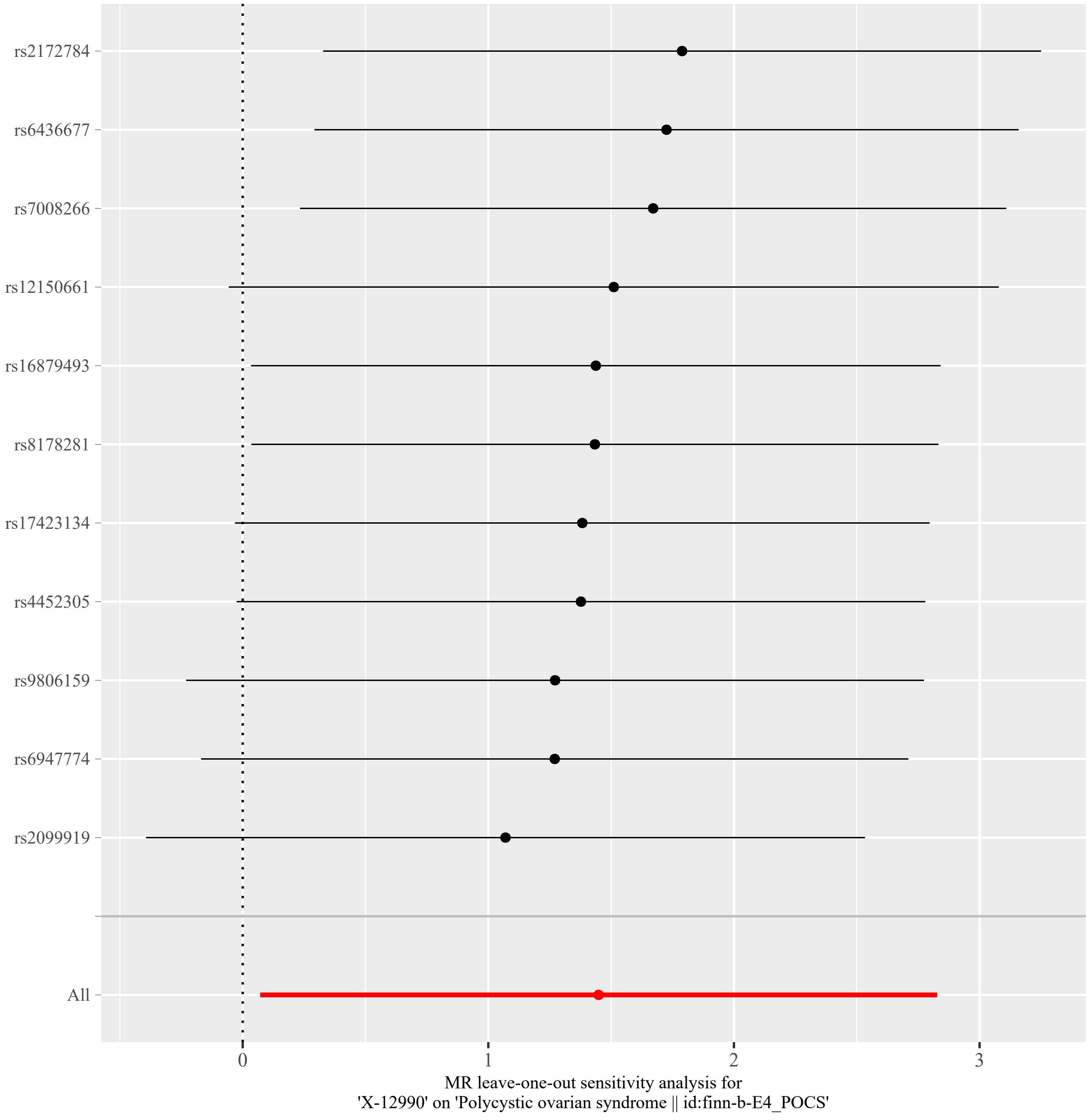

rs2172784
rs6436677
rs7008266
rs12150661
rs16879493
rs8178281
rs17423134
rs4452305
rs9806159
rs6947774
rs2099919
All
0
1
2
3
MR leave-one-out sensitivity analysis for
'X-12990' on 'Polycystic ovarian syndrome || id:finn-b-E4_POCS'
